# Author Correction: Bevacizumab, olaparib, and durvalumab in patients with relapsed ovarian cancer: a phase II clinical trial from the GINECO group

**DOI:** 10.1038/s41467-024-48915-9

**Published:** 2024-06-04

**Authors:** Gilles Freyer, Anne Floquet, Olivier Tredan, Aurore Carrot, Carole Langlois-Jacques, Jonathan Lopez, Frédéric Selle, Cyril Abdeddaim, Alexandra Leary, Coraline Dubot-Poitelon, Michel Fabbro, Laurence Gladieff, Michele Lamuraglia

**Affiliations:** 1 Department of Medical Oncology, Lyon 1 University, Lyon, France; 2 GINECO (Groupe d’Investigateurs Nationaux pour l’Etude des Cancers de l’Ovaire, Paris, France; 3 Institut de Cancérologie des HCL, Lyon, France; 4 Department of Medical Oncology - Gynecological Tumors, Institut Bergonié, Bordeaux, France; 5 Medical Oncology, Centre Léon Bérard, Lyon, France; 6 EMR 3738, UFR Lyon-Sud, Université Lyon1, Lyon, France; 7 Biostatistics and Bioinformatics Department, Hospices Civils de Lyon, Lyon, France; 8 Department of Biochemistry and Molecular Biology, Hospices Civils de Lyon, Lyon, France; 9 Department of Medical Oncology, Groupe Hospitalier Diaconesses Croix Saint-Simon, Paris, France; 10 Gynecologic Oncology Department, Centre Oscar Lambret, Lille, France; 11 Oncology Department, Institut Gustave Roussy, Villejuif, France; 12 Medical Oncology, Institut Curie Saint Cloud, Paris, France; 13 Department of Medical Oncology, Institut du Cancer de Montpellier, Montpellier, France; 14 Medical Oncology, Institut Claudius Regaud IUCT-Oncopole, Toulouse, France; 15 Medical Oncology, Institut de Cancérologie du CHUSE, Saint-Etienne, France

**Keywords:** Ovarian cancer, Ovarian cancer, Cancer therapy

Correction to: *Nature Communications* 10.1038/s41467-024-45974-w, published online 05 March 2024

The original version of the article file contained some inaccuracies in the Discussion section. The following corrections have now been made in the revised PDF and HTML versions of the article file.

The second part of the original statement “*In a small population of 44 patients, they obtained a median PFS of 22.4 months, which is encouraging; however, the number of previous treatment lines was not reported*^*27*^” was inaccurate since one of the inclusion criteria of the cited trial (ref. ^27^) was “Participant has received 2 previous courses of platinum-containing therapy”. The original sentence has now been rephrased as “*In a small population of 44 patients, they obtained a median PFS of 22.4 months, which is encouraging*^27^.”

In addition, in the original sentence “*Studies evaluating different triplet combinations are underway*^*37,38*^”, ref. ^38^ was incorrectly cited since related to the publication of the study protocol for the trial now published in ref. ^27^. The revised sentence now reads: “*Studies evaluating different triplet combinations are underway*^*37*^”. Original ref. ^38^ has been removed from the References list (and references numbering updated accordingly).

Original reference (now removed)

38. Lee, Y. J. et al. A single-arm phase II study of olaparib maintenance with pembrolizumab and bevacizumab in BRCA non-mutated patients with platinum-sensitive recurrent ovarian cancer (OPEB-01). *J. Gynecol. Oncol*. **32**, e31 (2021).

