# Supplementary material for: Bevacizumab, olaparib, and durvalumab in patients with relapsed ovarian cancer: a phase II clinical trial from the GINECO group
Source: Nat Commun. 2024 Mar 5;15:1985. doi: 10.1038/s41467-024-45974-w (PMC10914754; doi:10.1038/s41467-024-45974-w)
Supplement: Supplementary file 1 — Supplementary Information [file 41467_2024_45974_MOESM1_ESM.pdf]

Bevacizumab, Olaparib, and Durvalumab in Patients with Relapsed Ovarian Cancer: A Phase II Clinical Trial

SUPPLEMENTARY METHODS - KELIM-B Modelling

Semi-Mechanistic Kinetic-Pharmacodynamic (K-PD) Model Adjustment, and Qualification

Basic Population Modelling

CA-125 data are described as follows:

$$y_{ij} = f(X_{ij}, P_i) + \varepsilon_{ij} \quad (1)$$

Where y_{ij} corresponds to the jth observation of the ith individual, f(…) is the structural model described by the variables X_{ij} (time and dose), P_i is the vector of individual parameter for the ith individual and ε_{ij} is the residual error defined as the difference between observed data y_{ij} and individual predictions (y_{ij}). ε is assumed to be normally distributed with zero mean and variance to be estimated.

The individual parameters P_i are distributed around their typical values in the population θ (fixed effect or population parameter). Deviations from this typical value were expressed according to a log-normal distribution (random effect):

$$P_i = \theta \times e^{\eta_i} \quad (2)$$

Where θ is the typical value of the parameter P (mean in the population)-the random effect, η_i describes the inter-individual variability (IIV) which is the difference between the population value and the ith individual value. η_i values are expected to be a normal distribution with a 0 mean and ω^2 variance.

Structural Model and Model Qualification

A two-compartment model described treatment kinetics: central compartment (C1) receiving chemotherapy dosing (doses set to 1) and a transit compartment (C2) to describe the treatment lag-

time effect. The CA-125 production inhibition induced by the treatment is expressed by an indirect effect model using an E_{max} (EC50) relationship. This model was described by the following equations:

$$\begin{cases} \dfrac{dC1}{dt} = -K * C1 \\ \dfrac{dC2}{dt} = K * C1 - K * C2 \\ EFFECT(EC50) = 1 - \dfrac{C2}{C2 + EC50} \\ \dfrac{dCA125}{dt} = KPROD * EFFECT(EC50) - KELIM * CA125 \end{cases} \quad (3)$$

The initial conditions at time 0 were:

$$\begin{cases} C1(0) = 0 \\ C2(0) = 0 \\ CA125(0) = CA125_0 \end{cases} \quad (4)$$

Where K is the treatment kinetic rate constant ($days^{-1}$); KPROD is the CA-125 tumor production rate ($kU.mL^{-1}.days^{-1}$); EC_{50} is the concentration producing 50% of the maximum effect (AU); $KELIM^{TM}$ is the CA-125 elimination rate constant ($days^{-1}$); and $CA125_0$ is the estimated CA-125 at time = 0 ($kU.mL^{-1}$).

Standard errors of estimated parameters and goodness-of-fit plots (i.e., plots of observations vs predictions and of the distribution of normalized prediction errors [NPDE]) were used as major criteria. Moreover, 500 replicates of all individual CA-125 decline profiles were simulated using the final population model to perform a visual predictive check (VPC): the 10th, 50th, and 90th percentiles of the observed CA-125 values were compared with the 95% CI computed from the 500 simulated replicates. The observed and simulated median vs time profiles were visually compared.

SUPPLEMENTARY METHODS – TIS INFLAMMATORY SIGNATURE

The following genes were evaluated:

- HLA-E
- NKG7
- CD8A
- PSMB10
- HLA-DQA1
- HLA-DRB1
- CMKLR1
- CCL5
- CXCL9
- CD27
- CXCR6
- IDO1
- STAT1
- TIGIT
- LAG3
- PD-L1
- PD-L2
- CD276

https://nanostring.com/blog/tumor-inflammation-signature-gene-expression-profile-use-cases/

Supplementary Table 1. Baseline tumor inflammation signature (TIS) score in evaluable patients

	Platinum-resistant relapse (N=27)	Platinum-sensitive relapse (N=26)	Total (N=53)
**TIS**			
Mean (SD)	6.5 (1.09)	6.2 (1.26)	6.3 (1.18)
Median (range)	6.5 (4.4-8.8)	6.2 (3.9-8.9)	6.4 (3.9-8.9)

Supplementary Table 2. Baseline HRD status in evaluable patients

	Platinum-resistant relapse (N=15)	Platinum-sensitive relapse (N=15)	Total (N=30)
**HRD status**, n (%)			
Negative	7 (46.7%)	3 (20.0%)	10 (33.3%)
Positive	8 (53.3%)	12 (80.0%)	20 (66.7%)

Supplementary Table 3 Efficacy (RECIST 1.1 and clinical progression) according to prior PARP inhibitor, per investigator assessment in ITT patients, by platinum status

	Platinum-resistant relapse *N*=41				Platinum-sensitive relapse *N*=33			
	Best overall response				Best overall response			
	CR-PR (N=11)	SD-PD (N=28)	NE (N=2)	Total (N=41)	CR-PR (N=12)	SD-PD (N=20)	NE (N=1)	Total (N=33)
**Prior PARP inhibitor**, n (%)								
Yes	4 (30.8%)	8 (61.5%)	1 (7.7%)	13 (100%)	5 (29.4%)	12 (70.6%)	0 (0%)	17 (100%)
No	7 (25.0%)	20 (71.4%)	1 (3.6%)	28 (100%)	7 (43.8%)	8 (50.0%)	1 (6.3%)	16 (100%)

CR, complete response; PR, partial response; SD, stable disease, PD, progressive disease

Supplementary Figure 1. Kaplan-Meier estimate of progression-free survival according to the presence or not/missing status of a *BRCA* mutation. Number of patients at risk is shown. (Log Rank 2-sided test: Chisq=0.0739 for 1 degree of freedom). Source data are provided as a Source Data file.

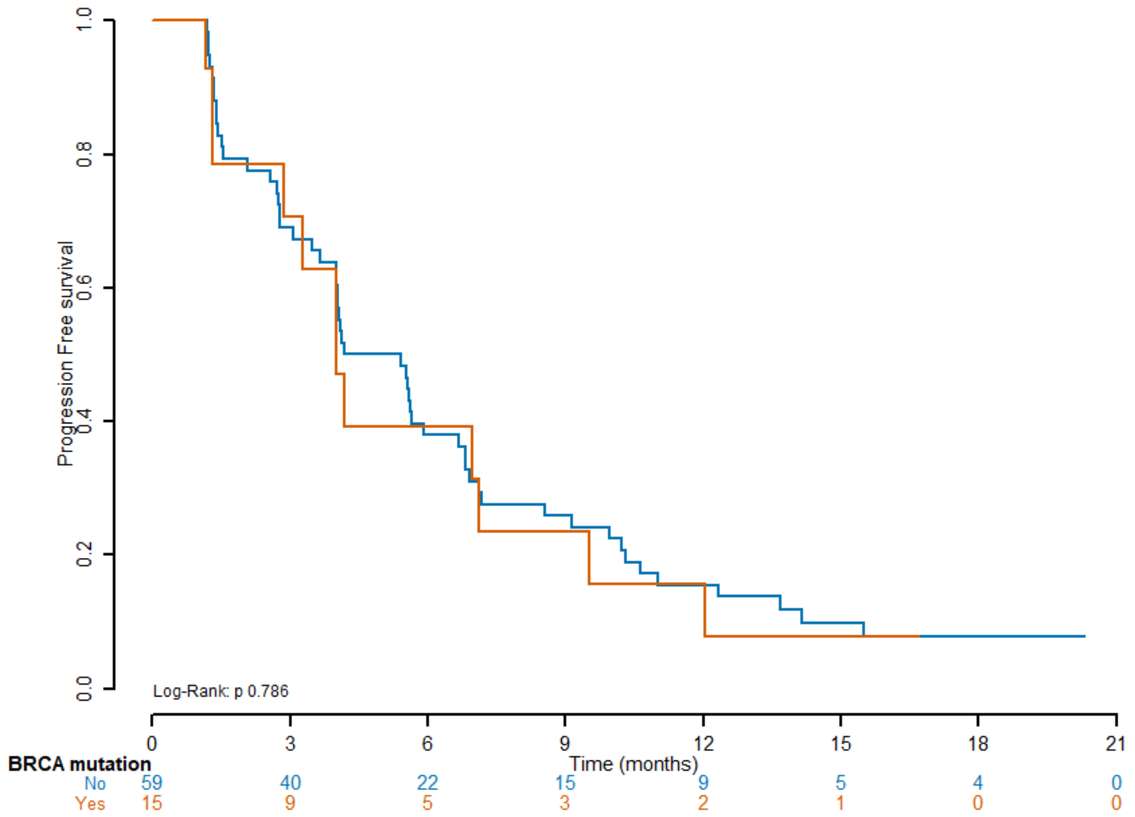

Supplementary Figure 2. Analysis of prediction of tumor response for selected biomarkers. Mean difference with 95% CI. Source data are provided as a Source Data file.

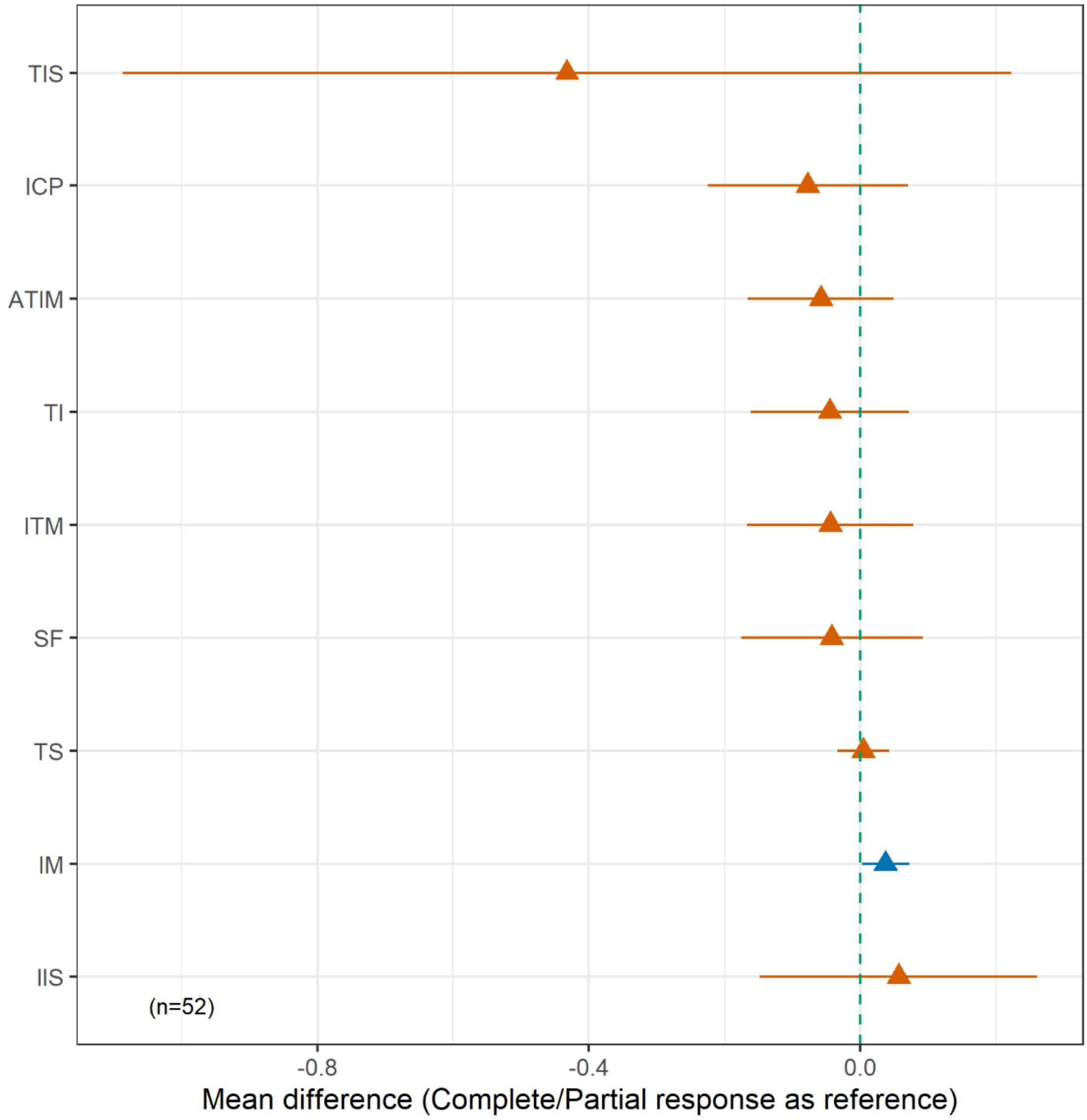

ICP=Immune Cell Population

ATIM=Anti-tumor Immune Activity

ITM=Inhibitory Tumor Mechanism

SF=Stromal Factors

TI=Tumor Immunogenicity

IIS=Inhibitory Immune Signal

TS=Tumor Sensitivity

IM=Inhibitory Metabolism

TIS=Tumor Inflammation Signature

Supplementary Figure 3. Boxplot analysis of patients with response (complete response /partial response) vs patients without response (stable disease/progressive disease) according to TIS score, in the platinum-resistant relapse (PRR) and platinum-sensitive relapse (PSR) groups. The line dividing the box is the median. The ends of the box show the upper (Q3) and lower (Q1) quartiles. The extreme line shows Q3+1.5xIQR to Q1-1.5xIQR (the highest and lowest value excluding outliers), with IQR the difference between Quartile 1 and Quartile 3. Source data are provided as a Source Data file.

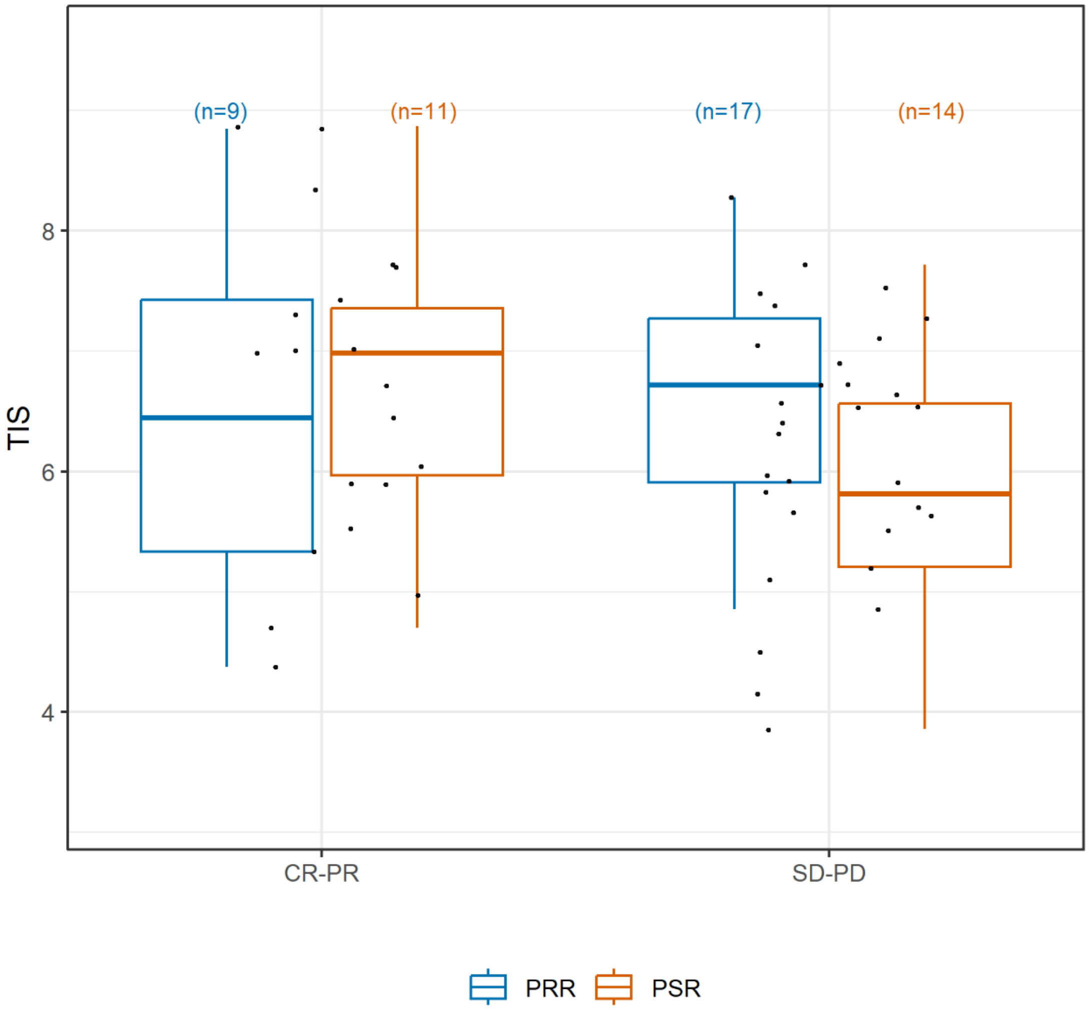

SUPPLEMENTARY NOTE 1 - BOLD PROTOCOL

A GINECO PHASE II TRIAL ASSESSING THE SAFETY AND EFFICACY OF THE BEVACIZUMAB (FKB238), OLAPARIB AND DURVALUMAB (MEDI 4736) COMBINATION IN PATIENTS WITH ADVANCED EPITHELIAL OVARIAN CANCER IN RELAPSE

BOLD PROTOCOL

(Bevacizumab (FKB238), Olaparib, and Durvalumab (MEDI 4736) in relapsing high grade carcinoma of the ovary)

GINECO- OV238		EudraCT N°	2018-002281-39
Development Phase: II		Subject:	Ovarian Carcinoma Advanced FIGO Stage IIIB – IV High Grade
**Version 5.0**	**13/01/2021**	Treatment:	Bevacizumab (FKB238), Olaparib and Durvalumab (MEDI 4736)

SCIENTIFIC COORDINATOR:	**Pr Léa PAYEN / Dr Jonathan LOPEZ**<br>Institut de Cancérologie des Hospices Civils de Lyon<br>165 Chemin du Grand Revoyet, 69495<br>PIERRE BENITE<br>E-mail: lea.payen@chu-lyon.fr; jonathan.lopez@chu-lyon.fr
COORDINATING INVESTIGATOR:	**Pr Gilles FREYER**<br>Institut de Cancérologie des Hospices Civils de Lyon<br>165 Chemin du Grand Revoyet, 69495<br>PIERRE BENITE<br>E-mail: gilles.freyer@univ-lyon1.fr
BIOSTATISTICIAN:	**Dr Catherine Mercier /**<br>**Ms Carole Langlois-Jacques**<br>Service de Biostatistique-Bioinformatique des Hospices Civils de Lyon<br>165 Chemin du Grand Revoyet, 69495<br>PIERRE BENITE<br>E-mail: catherine.mercier @chu-lyon.fr / carole.langlois-jacques@chu-lyon.fr
TRIAL MANAGER:	**Ms Laure JERBER**<br>Phone: + 33 (0)1 84 85 20 16<br>Fax: + 33(0)1 43 26 26 73<br>E-mail: ljerber@arcagy.org

**SPONSOR:**	**ARCAGY-GINECO**<br>8 rue Lamennais<br>75008 Paris

VERSION SPONSOR SIGNATURE PAGE

STUDY TITLE	**A GINECO phase II trial assessing the safety and efficacy of the Bevacizumab (FKB238), Olaparib (MEDI 4736) and Durvalumab combination in patients with advanced epithelial ovarian cancer in relapse: BOLD**
	This protocol describes BOLD trial, led by ARCAGY-GINECO.<br>It provides information about procedures for entering patients into the trial. The protocol should not be used as an aide-memoire or guide for the treatment of other patients.<br>Clinical problems related to this trial should be referred to the Principal Investigator, or the trial manager.
VERSION	**Version 5.0 dated 13th of January 2021**
SCIENTIFIC COORDINATOR	**Pr Léa PAYEN / Dr Jonathan LOPEZ**<br>Institut de Cancérologie des Hospices Civils de Lyon<br>165 Chemin du Grand Revoyet, 69495 PIERRE BENITE<br>Signatures ____________ / ____________
COORDINATING INVESTIGATOR	**Pr Gilles FREYER**<br>Institut de Cancérologie des Hospices Civils de Lyon<br>165 Chemin du Grand Revoyet, 69495 PIERRE BENITE<br>Signature ____________
BIOSTATISTICIAN	**Dr Catherine Mercier / Ms Carole Langlois-Jacques**<br>Institut de Cancérologie des Hospices Civils de Lyon<br>165 Chemin du Grand Revoyet, 69495 PIERRE BENITE<br>Signature ____________ / ____________
SPONSOR	**Mme Laure JERBER**<br>ARCAGY-GINECO<br>8 rue Lamennais<br>75008 Paris<br>FRANCE<br>Signature ____________

REVISION CHRONOLOGY PAGE

VERSION NAME	VERSION DATE	SUMMARY OF CHANGES	COUNTRY
Version 1.0	13/07/2018	First version submited	France
Version 1.1	14/08/2018	Version 1.0 modified following ANSM comments	France
Version 2.0	22/01/2019	Protocol containing amendment n°1	France
Version 3.0	16/09/2019	Protocol containing amendment n°2	France
Version 4.0	24/08/2020	Protocol containing amendment n°4	France
Version 5.0	13/01/2021	Protocol containing amendment n°5	France

PROTOCOL SUMMARY

**Study title**	*A GINECO phase II trial assessing the safety and efficacy of the Bevacizumab (FKB238), Olaparib and Durvalumab (MEDI 4736) combination in patients with advanced epithelial ovarian cancer in relapse :* BOLD
**Study Code**	BOLD (**B**evacizumab, **OL**aparib, and **D**urvalumab in relapsing high grade carcinoma of the ovary).
**EudraCT number**	2018-002281-39
**Sponsor ID**	GINECO-OV238
**Sponsor**	**ARCAGY-GINECO**<br>8 rue Lamannais<br>75008 Paris
**Participating groups**	GINECO
**Scientific coordinator**	Pr Léa PAYEN, PharmD, PhD, Department of Tumor Biology Institut de Cancérologie des Hospices civils de Lyon, France<br>Dr Jonathan LOPEZ, MD, PhD, Department of Molecular Biology, Institut de Cancérologie des Hospices civils de Lyon, France
**Coordinating Investigator**	Pr Gilles FREYER, MD, PhD, Medical Oncology Department, Institut de Cancérologie des Hospices civils de Lyon, France
**Biostatistician**	Dr Catherine Mercier, MD, PhD, Department of Bio-statistics, Institut de Cancérologie des Hospices civils de Lyon, France<br>Ms Carole Langlois-Jacques, Department of Bio-statistics, Institut de Cancérologie des Hospices civils de Lyon, France
**Indication**	Patients with high grade serous or high grade endometrioid or other high grade epithelial non mucinous ovarian tumor, with at least one previous line of platinum-taxane chemotherapy, and present with platinum resistant disease (PRR) or platinum-sensitive relapse (PSR), whatever the line of chemotherapy given at relapse.
**Rational of the study**	The lifetime risk of ovarian cancer is around 1 to 2% in developed countries (Jayson et al., 2014). While there are effective treatment options that significantly prolong survival, advanced ovarian cancer (AOC) is still mostly a fatal disease, which requires additional therapeutic options. After first-line cytoreductive surgery and chemotherapy, 70 % of patients achieving complete remission will relapse. In patients with platinum sensitive relapse, long remissions may be obtained by platinum containing chemotherapy regimens and, in some cases, by surgery. Bevacizumab is indicated in first-relapse, in patients who did not previously receive this drug. Olaparib is SoC for mBRCA ½ PSR maintenance patients. However, relapsing AOC is no longer a curable disease and iterative relapses usually occur. In patients with platinum resistant disease, non-platinum cytotoxic agents such as paclitaxel, liposomal doxorubicin, or topotecan are indicated, in combination with bevacizumab, but the overall prognosis remains poor.<br>There is clearly an unmet therapeutic need in patients with either platinum-resistant relapse or platinum-sensitive relapse.

- **Durvalumab in epithelial ovarian cancer**

Durvalumab is an antibody directed against PD-L1 r. PD-L1 is the ligand of PD-1, a receptor expressed by CD8+ cytotoxic lymphocytes. The interaction between the PD-1 receptor and its ligand leads to desactivation and apoptosis of the CD8+ lymphocyte. Conversely, anti-PD(L)-1 therapy triggers a T cell immune response (Hedge et al., 2016, Mony et al., 2015) which can induce prolonged remissions (Larkin et al., 2015).

In the tumor micro-environment, the presence of an immune infiltrate containing lymphocytes and expressing IFNy, CD8 and PD-L1 has been found to be associated with antitumor activity of the immune checkpoint inhibitors (Hedge et al., 2016). A high mutational load and mutation correction deficiencies, which provide new antigens for the immune system to react with, have also been shown to drive the immune response triggered by anti-PD(L)-1 therapies (Le et al., 2015, Rivzi et al., 2015). Upon treatment initiation, modification of the tumor immune environment can be observed and seems to correlate with subsequent response (Chen et al., 2016). All these observations provide possible strategies to potentiate and enhance immunotherapy.

Ovarian cancers have a moderate mutational load compared to cancers known to be very sensitive to anti-PD-1 agents such as melanoma or lung cancers (Martincorena et al., 2015). They do, however, have tumor-infiltrating lymphocytes and expression of PD-1 and PD-L1 in a significant proportion of cases. Thus, anti-PD(L)-1 monotherapy has shown moderate efficacy in ovarian cancer compared to highly mutated and inflamed melanoma or lung tumors. Here, we propose to combine the anti-PD-L1 antibody durvalumab with olaparib, which inhibits mutation repair, and bevacizumab, which modulates the tumor microenvironment.

- **Olaparib**

Olaparib is an inhibitor of the enzyme poly-ADP-ribosyl polymerase (PARP), an effector of the signaling of DNA damage, particularly strand breaks. Olaparib is currently approved for the treatment of relapsing, platinum-sensitive ovarian cancer in patients with either germinal or somatic BRCA mutations. Its efficacy has also been observed in non-mutated tumors, since at least 50 % of high grade serous carcinomas exhibit DNA homologous recombination deficiency (HRD) (Ledermann et al., 2014, Oza et al., 2015). This drug is well tolerated, even as a maintenance therapy on long periods of time.

The inhibition of PARP increases cellular use of the non-homologous end joining (NHEJ) pathway for the reparation of double strand breaks (Patel et al., 2011). NHEJ is a low-fidelity mechanism with a high chance of mutation after strand break repair. Therefore, olaparib is expected to increase mutational load in tumor cells, which has been shown to correlate with anti-tumor immune response (Le et al., 2015, Rivzi et al., 2015).

In addition, blocking PARP enzymatic activity blocks its release from DNA strand breaks, thereby causing a bulky and toxic buildup of PARP complexes (Murai et al., 2012); this cytotoxic mechanism might also release antigens and necrotic signals which may favor an immune response.

The potential synergy between olaparib and anti-VEGF therapy has been recently supported by the results of randomized phase 2 trial showing that the combination of the antiangiogenic cediranib (30mg daily, per os)) plus olaparib (200 mg capsules twice daily) for the treatment of recurrent platinum-sensitive HGSOC compared to olaparib alone

prolonged PFS from a median of 9.0 months up to 17.7 months (hazard ratio = 0.42, 95% CI 0.23-0.76, p=0.005) (J Liu, ASCO 2014, LBA 5500) (NCT 01116648). In addition the response rate of the combination was 80% compared to 48% for the single agent olaparib (p=0.002). However, toxicity of the combination was significant leading dose adaptation in 77% of patients (versus 24% in the single agent arm), with increased occurrence of severe hypertension, fatigue and diarrhea which are previously reported cediranib toxicity.

- **Bevacizumab**

Bevacizumab is a monoclonal antibody directed against the vascular-endothelial growth factor (VEGF), which activates tyrosine-kinase receptors that promote proliferation, differentiation and migration of cells constituting blood vessels.
Clinically, bevacizumab in combination with chemotherapy has been shown to increase PFS in phase III trials in combination with chemotherapy, in various situations such as first-line, platinum sensitive relapse and resistant relapse (Aghajanian et al., 2012, Burger et al., 2011, Perren et al., 2011, Pujade-Lauraine et al., 2014) .
Because tumor cells do not signal and migrate properly, intratumoral blood vessels have a different structure than those found elsewhere. Anti-VEGF therapies tend to normalize their structure, which correlates with pathologic response (Wallin et al., 2014). This could facilitate infiltration by antigen-specific T cells and destruction by the immune system, as observed in a xenograft model (Wallin et al., 2014). As a consequence, we hypothesize that a combination of bevacizumab and durvalumab would have synergistic efficacy.

- **Clinical data in combination**

Bevacizumab and olaparib have already been tested on 12 patients in a phase I trial at their usual doses (10 mg/kg q2w and 400 mg bid – 50 mg capsules -, respectively), and no DLTs were observed (Dean et al., 2012). The addition of an anti-VEGF small molecule, cediranib, to olaparib doubled the median PFS in a randomized phase II trial in patients with platinum sensitive relapse, with a manageable safety profile (Liu et al., 2014).
A recently reported phase I trial established the RDP2D of Durvalumab and Olaparib – 150 mg tablets –, when given in combination, at 1500 mg every 4 weeks, and 300 mg bid, respectively (Lee et al. 2017). In addition, the ENGOT/GINECO PAOLA phase III trial is currently evaluating the combination of Olaparib and Bevacizumab as first-line maintenance after platinum-paclitaxel combination, in patients with advanced high-grade serous ovarian carcinoma. Under the hypothesis of a survival benefit in favor of this combination, it would also be of interest to assess the value of adding Durvalumab in order to improve the efficacy of the overall combination.
There are no trials to date assessing anti-VEGF in combination with anti-PARP and anti-PD-L1 therapy.

Beside additive efficacy, a synergistic effect could be expected :

- Between bevacizumab and durvalumab, through normalization of blood vessel and potentiation of immunologic infiltration.
- Between olaparib and durvalumab, through cytotoxicity-mediated release of antigens and impairment of mutation repair mechanisms, thereby increasing neoantigen loads.

- Between olaparib and bevacizumab, through tumor environment modulation and signaling of DNA damage inhibition, which has already been tested with the anti-VEGF cediranib.

For those reasons we propose a phase II trial of Olaparib, Bevacizumab and Durvalumab combination, in patients with relapsing AO high grade carcinoma :

- In platinum sensitive relapse (PSR), whatever the line, in patients not amenable to frontline surgery of the relapse and previously-treated by a platinum-containing chemotherapy in first line and
  - ✓ Either didn't receive any of the tested drugs,
  - ✓ Or previously received either bevacizumab or olaparib BUT NOT the combination of both drugs.
- In platinum-resistant relapse (PRR), in previously untreated patients for their relapse, or in patients who received a maximum of 1 chemotherapy regimen in this setting and either
  - ✓ Either didn't receive any of the tested drugs,
  - ✓ Or previously received either bevacizumab or olaparib BUT NOT the combination of both drugs.

We will also translational research, including :

- Assessment of germline and somatic BRCA mutations and determination of HRD phenotype and mutational load by NGS
- Quantification of mutagenesis in simultaneous treatment on ctDNA
- Characterization of immune response in the tumor by Nanostring immuno-oncology panel on tumors

Study Objectives

Primary objective

Efficacy and tolerance of the following combination:
Durvalumab 1.12 g IV Q3W
Bevacizumab (FKB238) 15 mg/kg Day 1 Q3W
Olaparib 300 mg twice daily po (150 mg and 100mg tablets), continuously (NB : using the new tablet formulation, the 300 mg dose is considered equivalent to the 400 mg capsule dose)
The primary objective is the rate of clinical and radiological non-progressive disease, as assessed by immune-related response criteria (irRC) (Wolchok et al. 2009) :

- At 3 months in the PRR cohort
- At 6 months in the PSR cohort

Secondary objectives

The secondary objectives are

- CA 125 decline as expressed by the KELIM parameter
- Progression free survival (PFS)
- Overall survival (OS)
- Tumor response
- Toxicity as assessed by CTCAE V.5.0 scale

Translational research objectives

The translational research objectives are

- Correlate olaparib administration and durvalumab efficacy

	• Correlate HRD phenotype and response to anti-PARP therapy<br>• Correlate tumor microenvironnement, immune check point status, and durvalumab response.
**Study design**	This is a multicenter, national, open label, non-randomized, phase II trial in patients with ovarian cancer.<br>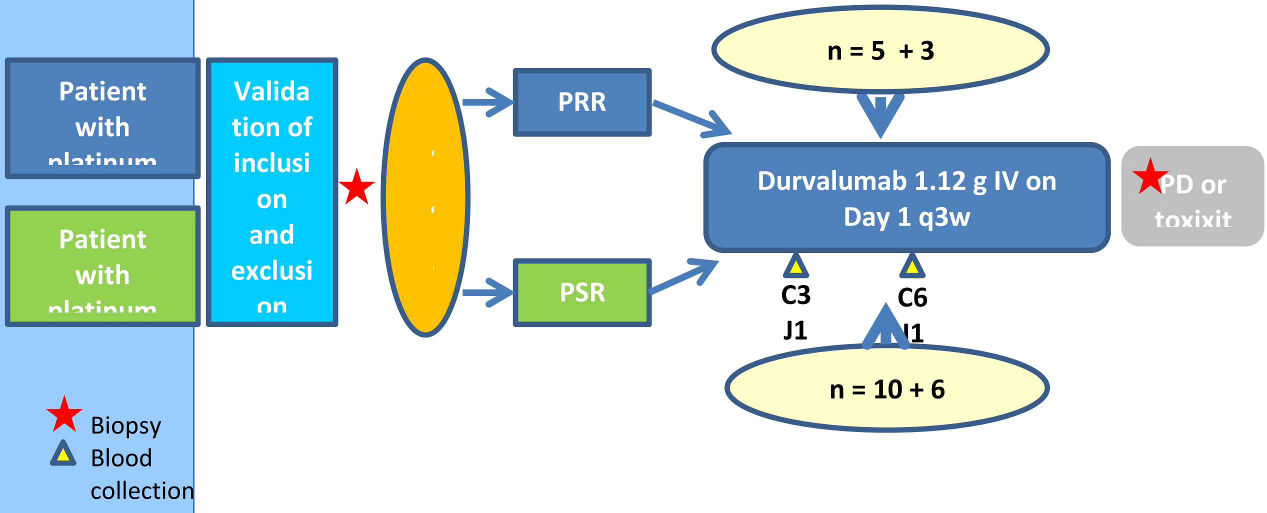<br>*Fig. 1: Design of the BOLD trial.*
**Patient and site Number**	63 patients recruited in approximately 10 sites.
**Inclusion criteria**	**<u>Patients with platinum resistant relapse</u>**<br>I-1 Female Patient must be ≥18 years of age.<br>I-2 Signed informed consent and ability to comply with treatment and follow-up.<br>I-3 Patient with :<br>Ovarian cancer, primary peritoneal cancer and/or fallopian-tube cancer, histologically confirmed (based on local histopathological findings): high grade serous or high grade endometrioid or other high grade epithelial non mucinous ovarian tumor.<br>I-4 Patient who has completed at least one line of platinum-taxane chemotherapy, and presents with platinum resistant relapse (resistant disease defined by a tumor progression less than six months after the last dose of platinum) [Note: the patient may have received one or even more line of platinum based chemotherapy].<br>I-5 Patient who didn't receive any of the tested drugs, or previously received either bevacizumab or olaparib BUT NOT the combination of both drugs.<br>I-6 At least one measurable or evaluable lesion that can be accurately assessed at baseline by computed tomography (CT) (or magnetic resonance imaging [MRI] where CT is contraindicated) and is suitable for repeated assessment as per irRECIST. The baseline scan must be obtained within 28 days of first dose.<br>I-7 Availability of a pre-treatment tumor sample (archival FFPE block or fresh biopsy if feasible) lasting of less than 3 months before inclusion into the study and performed AFTER the last chemotherapy administration.

I-8 Patient not amenable to cytoreductive surgery at the time of relapse (surgery is not allowed during the protocole treatment).

I-9 Patient must have normal organ and bone marrow function:

a. Hemoglobin ≥ 9.0 g/dL. (Transfusions is not allowed within 28 days before randomization)

b. Absolute neutrophil count (ANC) ≥ 1.5 x 10^9/L.

c. Platelet count ≥ 100 x 10^9/L. (Platelet transfusion or G-CSF administration is not allowed within 28 days before randomization)

d. Total bilirubin ≤ 1.5 x institutional upper limit of normal (ULN).

e. Aspartate aminotransferase / Serum Glutamic Oxaloacetic Transaminase (ASAT/SGOT)) and Alanine aminotransferase / Serum Glutamic Pyruvate Transaminase (ALAT/SGPT)) ≤ 2.5 x ULN, unless liver metastases are present in which case they must be ≤ 5 x ULN.

f. Creatinine clearance ≥ 60 mL/min by Cockcroft and Gault formula.

g. Patient not receiving anticoagulant medication who has an International Normalized Ratio (INR) ≤ 1.5 and an Activated ProThrombin Time (aPTT) ≤ 1.5 x ULN. The use of full-dose oral or parenteral anticoagulants is permitted as long as the INR or APTT is within therapeutic limits (according to site medical standard). If the patient is on oral anticoagulants, dose has to be stable for at least two weeks at the time of inclusion.

h. Urine dipstick for proteinuria < 2+. If urine dipstick is ≥ 2+, 24-hour urine must demonstrate < 1 g of protein in 24 hours.

i. Normal blood pressure or adequately treated and controlled hypertension (systolic BP ≤ 150 mmHg and/or diastolic BP ≤ 90 mmHg).

I-10 Expectancy of at least 12 weeks

I-11 Eastern Cooperative Oncology Group (ECOG) performance status 0-1.

I-12 Postmenopausal or evidence of non-childbearing status for women of childbearing potential prior to the first dose of study treatment (see protocol appendix 1).

I-13 As this study will include patients in France, a subject will be eligible for inclusion in this study only if either affiliated to, or a beneficiary of, a social category.

Patients with platinum sensitive relapse

I-1 Female Patient must be ≥18 years of age.

I-2 Signed informed consent and ability to comply with treatment and follow-up.

I-3 Patient with :

Ovarian cancer, primary peritoneal cancer and/or fallopian-tube cancer, histologically confirmed (based on local histopathological findings): high grade serous or high grade endometrioid or other high grade epithelial non mucinous ovarian tumor.

I-4 Patient who is in platinum-sensitive relapse, whatever the line of chemotherapy given at relapse [Note: any chemotherapy previously administered must have contained a

platinum compound]. The platinum sensitive relapse is defined by a tumor progression occurring more than six months after the last dose of platinum chemotherapy.

I-5 Patient who didn't receive any of the tested drugs, or previously received either bevacizumab or olaparib BUT NOT the combination of both drugs.

I-6 At least one measurable or evaluable lesion that can be accurately assessed at baseline by computed tomography (CT) (or magnetic resonance imaging [MRI] where CT is contraindicated) and is suitable for repeated assessment as per irRECIST. The baseline scan must be obtained within 28 days of first dose.

I-7 Availability of a pre-treatment tumor sample (archival FFPE block or fresh biopsy if feasible) lasting of less than 3 month before inclusion into the study and performed AFTER the last chemotherapy administration.

I-8 Patient not amenable to cytoreductive surgery at the time of relapse (surgery is not allowed during the protocole treatment).

I-9 Patient must have normal organ and bone marrow function:

a. Hemoglobin ≥ 9.0g/dL. (Transfusions is not allowed within 28 before randomization)

b. Absolute neutrophil count (ANC) ≥ 1.5 x 10^9/L.

c. Platelet count ≥ 100 x 10^9/L. (Platelet transfusion or G-CSF administration is not allowed within 28 days before randomization).

d. Total bilirubin ≤ 1.5 x institutional upper limit of normal (ULN).

e. Aspartate aminotransferase / Serum Glutamic Oxaloacetic Transaminase (ASAT/SGOT)) and Alanine aminotransferase / Serum Glutamic Pyruvate Transaminase (ALAT/SGPT)) ≤ 2.5 x ULN, unless liver metastases are present in which case they must be ≤ 5 x ULN.

f. Creatinine clearance ≥ 60 mL/min by Cockcroft and Gault formula.

g. Patient not receiving anticoagulant medication who has an International Normalized Ratio (INR) ≤ 1.5 and an Activated ProThrombin Time (aPTT) ≤ 1.5 x ULN. The use of full-dose oral or parenteral anticoagulants is permitted as long as the INR or APTT is within therapeutic limits (according to site medical standard). If the patient is on oral anticoagulants, dose has to be stable for at least two weeks at the time of incusion.

h. Urine dipstick for proteinuria < 2+. If urine dipstick is ≥ 2+, 24-hour urine must demonstrate < 1 g of protein in 24 hours.

i. Normal blood pressure or adequately treated and controlled hypertension (systolic BP ≤ 150 mmHg and/or diastolic BP ≤ 90 mmHg).

I-10 Expectancy of at least 12 weeks

I-11 Eastern Cooperative Oncology Group (ECOG) performance status 0-1.

I-12 Postmenopausal or evidence of non-childbearing status for women of childbearing potential prior to the first dose of study treatment.

I-13 As this study will include patients in France, a subject will be eligible for inclusion in this study only if either affiliated to, or a beneficiary of, a social category.

Exclusion criteria	**<u>Patient must not enter the study if any of the following exclusion criteria are fulfilled</u>**

E-1 Non-epithelial origin of the tumor (i.e. germ cell tumor).

E-2 Ovarian tumors of low malignant potential (e.g. borderline tumors), or mucinous carcinoma.

E-3 Carcinosarcoma (Mixed Mullerian Tumor)

E-4 Patient with synchronous primary endometrial cancer unless both of the following criteria are met:

- Stage < II,
- Less than 60 years old at the time of diagnosis of endometrial cancer with stage IA or IB grade 1 or 2, or stage IA grade III endometrial carcinoma,OR ≥ 60 years old at the time of diagnosis of endometrial cancer with stage IA grade 1 or 2 endometrioid adenocarcinoma.

Patient with serous or clear cell adenocarcinoma or carcinosarcoma of the endometrium is not eligible.

E-5 Other malignancy within the last 5 years except: adequately treated non-melanoma skin cancer, curatively treated in situ cancer of the cervix, ductal carcinoma in situ (DCIS). Patient with a history of localized malignancy diagnosed over 5 years ago may be eligible provided she completed her adjuvant systemic therapy and remains free of recurrent or metastatic disease. Patient with history of primary triple negative breast cancer may be eligible provided she completed her definitive anticancer treatment more than 3 years ago and she remains breast cancer disease free prior to start of study treatment.

E-6 Patient with myelodysplastic syndrome/acute myeloid leukemia history.

E-7 Current or prior use of immunosuppressive medication within 14 days (use 28 days if combining durvalumab with a novel agent) before the first dose of durvalumab, with the exceptions of intranasal and inhaled corticosteroids or systemic corticosteroids at physiological doses, which are not to exceed 10 mg/day of prednisone, or an equivalent corticosteroid. The following are exceptions to this criterion:

- Intranasal, inhaled, topical steroids, or local steroid injections (e.g., intra articular injection)
- Systemic corticosteroids at physiologic doses not to exceed <<10 mg/day>> of prednisone or its equivalent
- Steroids as premedication for hypersensitivity reactions (e.g., CT scan premedication)

E-8 Any unresolved toxicity CTCAE Grade ≥2 from previous anticancer therapy with the exception of alopecia, vitiligo, and the laboratory values defined in the inclusion criteria

- Patients with Grade ≥2 neuropathy will be evaluated on a case-by-case basis after consultation with the Study Physician.
- Patients with irreversible toxicity not reasonably expected to be exacerbated by treatment with durvalumab may be included only after consultation with the Study Physician.

E-9 Active or prior documented autoimmune or inflammatory disorders (including inflammatory bowel disease [e.g., colitis or Crohn's disease], diverticulitis [with the exception of diverticulosis], systemic lupus erythematosus, Sarcoidosis syndrome, or

	Wegener syndrome [granulomatosis with polyangiitis, Graves' disease, rheumatoid arthritis, hypophysitis, uveitis, etc]). The following are exceptions to this criterion:<br>• Patients with vitiligo or alopecia<br>• Patients with hypothyroidism (e.g., following Hashimoto syndrome) stable on hormone replacement<br>• Any chronic skin condition that does not require systemic therapy<br>• Patients without active disease in the last 5 years may be included but only after consultation with the study physician<br>• Uncontrolled intercurrent illness, including but not limited to, ongoing or active infection, symptomatic congestive heart failure, uncontrolled hypertension, unstable angina pectoris, cardiac arrhythmia, interstitial lung disease, serious chronic gastrointestinal conditions associated with diarrhea, or psychiatric illness/social situations that would limit compliance with study requirement, substantially increase risk of incurring AEs or compromise the ability of the patient to give written informed consent<br>• History of active primary immunodeficiency<br>• Active infection including tuberculosis (clinical evaluation that includes clinical history, physical examination and radiographic findings, and TB testing in line with local practice), hepatitis B (known positive HBV surface antigen (HBsAg) result), hepatitis C, or human immunodeficiency virus (positive HIV 1/2 antibodies). Patients with a past or resolved HBV infection (defined as the presence of hepatitis B core antibody [anti-HBc] and absence of HBsAg) are eligible. Patients positive for hepatitis C (HCV) antibody are eligible only if polymerase chain reaction is negative for HCV RNA.<br>**E-10** Patient receiving radiotherapy within 6 weeks prior to study treatment.<br>**E-11** Major surgery within 4 weeks of starting study treatment and patient must have recovered from any effects of any major surgery.<br>**E-12** Previous allogenic bone marrow transplant.<br>**E-13** Any previous treatment with Anti PD(L)-1 immunotherapy, including durvalumab<br>**E-14** Any previous treatment with a PARP inhibitor in combination with an anti-VEGF (previous treatment with PARP inhibitor alone or anti-VEGF alone is allowed).<br>**E-15** Past medical history of interstitial lung disease, drug-induced pneumonitis, radiation pneumonitis that required steroid treatment, or any evidence of clinically active interstitial lung disease<br>**E-16** Administration of other simultaneous chemotherapy drugs, any other anticancer therapy or anti-neoplastic hormonal therapy, or simultaneous radiotherapy during the trial treatment period (hormonal replacement therapy is permitted as are steroidal antiemetics).<br>**E-17** Current or recent (within 10 days prior to inclusion) chronic use of aspirin > 325 mg/day.<br>**E-18** Concomitant use of known potent CYP3A4 inhibitors such as ketoconazole, itraconazole, ritonavir, indinavir, saquinavir, telithromycin, clarithromycin and nelfinavir. The required washout period prior to starting study treatment is 2 weeks.

	**E-19** Concomitant use of known strong (e.g. phenobarbital, enzalutamide, phenytoin, rifampicin, rifabutin, rifapentine, carbamazepine, nevirapine and St John's Wort) or moderate CYP3A inducers (e.g. bosentan, efavirenz, modafinil). The required washout period prior to starting study treatment is 5 weeks for enzalutamide or phenobarbital and 3 weeks for other agents. Patients should stop using herbal remedies 7 days prior to the first dose of study medication and for the duration of the trial.<br>**E-20** Prior history of hypertensive crisis (CTCAE grade 4) or hypertensive encephalopathy.<br>**E-21** Clinically significant (e.g. active) cardiovascular disease, Previous Cerebro-Vascular Accident (CVA), Transient Ischemic Attack (TIA) or Sub- Arachnoids Hemorrhage (SAH) within 6 months prior to inclusion.<br>**E-22** History Clinically significant (e.g. active) cardiovascular disease, including:<br>• Myocardial infarction or unstable angina within ≤ 6 months of inclusion,<br>• New York Heart Association (NYHA) ≥ grade 2 congestive heart failure (CHF),<br>• Poorly controlled cardiac arrhythmia despite medication (patient with rate controlled atrial fibrillation are eligible), or any clinically significant abnormal finding on resting ECG,<br>• Peripheral vascular disease grade ≥ 3 (e.g. symptomatic and interfering with activities of daily living [ADL] requiring repair or revision) or evidence of hemorrhagic disorders within 6 months prior to treatment administration.<br>**E-23** Evidence of bleeding diathesis or significant coagulopathy (in the absence of coagulation).<br>**E-24** History or clinical suspicion of brain metastases or spinal cord compression. CT/MRI of the brain is mandatory (within 4 weeks prior to inclusion) in case of suspected brain metastases. Spinal MRI is mandatory (within 4 weeks prior to inclusion) in case of suspected spinal cord compression.<br>**E-25** Significant traumatic injury during 4 weeks prior to inclusion.<br>**E-26** Non-healing wound, active ulcer or bone fracture. Patient with granulating incisions healing by secondary intention with no evidence of facial dehiscence or infection is eligible but require 3 weekly wound examinations.<br>**E-27** History of VEGF therapy related abdominal fistula or gastrointestinal perforation or active gastrointestinal bleeding within 6 months prior to the first study treatment.<br>**E-28** Current, clinically relevant bowel obstruction, including sub-occlusive and occlusive disease.<br>**E-29** Patient with evidence of abdominal free air not explained by paracentesis or recent surgical procedure.<br>**E-30** Evidence of any other disease, metabolic dysfunction, physical examination finding or laboratory finding giving reasonable suspicion of a disease or condition that contraindicates the use of an investigational drug or puts the patient at high risk for treatment related complications.<br>**E-31** Pregnant or lactating women.<br>**E-32** Participation in another clinical study with an investigational product during her chemotherapy course immediately prior to inclusion.

	**E-33** Patient unable to swallow orally administered medication and patient with gastrointestinal disorders likely to interfere with absorption of the study medication.<br>**E-34** Patient with a known hypersensitivity to olaparib, durvalumab or bevacizumab or any of the recipients of those products.<br>**E-35** Immunocompromised patient, e.g., with known active hepatitis (i.e. Hepatitis B or C) due to risk of transmitting the infection through blood or other body fluids or patient who is known to be serologically positive for human immunodeficiency virus (HIV).
**Investigational medicinal and comparator products, dosage and mode of administration**	Patients will be treated the same way in the PRR and PSR cohorts:<br>**Durvalumab 1.12 g IV on Day 1 Q3W**<br>**Bevacizumab (FKB238) 15 mg/kg Day 1 Q3W**<br>**Olaparib 300 mg bid po, continuously**<br><br>Patients will be treated upon disease progression, unacceptable toxicity or consent withdrawal.
**Statistical considerations**	**Calculating of needed patient number and analysis of primary endpoints**<br><br>The statistical considerations are independent in the two cohorts.<br>The sample size was calculated independently in the two cohorts. The study is based on two hypotheses for each cohort.<br><br>**Platinum resistant cohort:**<br>• The null hypothesis (p0) is 50% or lower. A rate of non-progressive disease at 3 months of 50% was considered undesirable (p0 = 50%) compared with historical control in this patient population<br>• The alternative hypothesis is a rate of non-progressive disease at 3 months higher than 50%.<br>• The positive hypothesis (p1) used for sample size calculation is 75%. A non-progressive disease rate of 75% was considered to warrant further investigations<br><br>Sample size was calculated based on the exact binomial distribution. Using a one stage design and the exact binomial distribution [A'Hern, 2001] , twenty three evaluable patients must be included in trial (**n=23**). This design yields a one-sided type-1 error rate of α=5% maximum and a power of at least 80% when the true non progressive disease rate is 75%. **The total number of patients in PRR cohort might be higher than 23 to anticipate non evaluable patients who will be replaced**.<br><br>**Platinum Sensitive cohort:**<br>• The null hypothesis (p0) is 65%. A rate of non-progressive disease at 6 months of 65% was considered undesirable<br>• The alternative hypothesis is a rate of non-progressive disease at 6 months higher than 65%.<br>• The positive hypothesis (p1) used for sample size calculation is 84%. A non-progressive disease rate of 84% was considered to warrant further investigations.<br>•We again based sample size and cut-offs calculation on the exact binomial distribution. Using a one stage design and the exact binomial distribution, forty evaluable patients must be included in the trial (n=40). This design yields a one-sided type-1 error rate of α=3% maximum and a power of at least 82% when the true non progressive disease rate is 84%.

The total number of patients in PSR cohort might be higher than 40 to anticipate non evaluable patients who will be replaced.

Translational research

<u>Analysis of the objectives and methods for the translational research</u>

- **Hypothesis 1: Early increase of tumour mutational burden (TMB) following treatment by olaparib is predictive of a better outcome by increasing efficacy of durvalumab**

We will compare the absolute number of mutations by megabase of genomic DNA (TMB) at baseline, week 3 and week 6 to test whether olaparib is increasing mutational load. **Patients will be classified as TMB responders** if at W6 they are moving up from low to intermediate or from intermediate to high categories as defined by the FMI.

- **Hypothesis 2: HRD phenotype is associated with a better response to anti-PARP therapies**

Patients will be included in the study irrespective of their BRCA and HRD status. HRD phenotype will be defined using a large NGS panel and patients stratified as **HR defective or competent**.

We will **test whether HR status is associated a better PFS** using a Mann-Whitney test.

- **Hypothesis 3: Tumour microenvironment and immune checkpoint status before treatment is predictive of a better response to durvalumab.**

T cell-inflammed score (TIS) and alternative resistance mechanisms will be evaluated using IO360 gene expression panel (770 genes). TIS will be analyzed as a continuous quantitative variable or to stratify tumours as TIS low versus TIS high based on the defined cut-off. Expression of alternative genes will allow us to stratify patients into 2 groups: PD1/PD-L1 pathway only versus alternative escape mechanisms. Clinical benefit of durvalumab is believed to be compromised in the second category.

We will **test whether the TIS score is associated with a better PFS** using a Cox model and a Mann-Whitney test. Association between PFS and presence of alternative escape mechanism will also be evaluated (Mann Whitney).

Time of the study	Type of sample	Optional or mandatory
Screening	• Tumor biopsy / surgical sample obtained within 3 months before inclusion	Mandatory
	• Blood sample for ctDNA, biomarker and translational research<br>• Blood sample for initial tumor mutational load	Translational study : Mandatory
On treatment (on cycle 3, day 1)	• Blood sample for ctDNA, biomarker and translational research<br>• Blood sample for tumor mutational load after three weeks of treatment	Translational study : Mandatory
On treatment (on cycle 6, day 1)	• Blood sample for ctDNA, biomarker and translational research<br>• Blood sample for tumor mutational load after six weeks of treatment	Translational study : Mandatory
At relapse	• Tumor biopsy	Optional

Study Calendar

FPI: Q4 2018

Accrual period: 24 months

LPI: Q4 2020

Treatment duration: until progression

	**Follow-up duration:** 12 months<br>**Estimated date of last FUP:** Q2 2023<br>**Primary endpoint analysis:** Q2 2021<br>**CSR:** Q2 2024
**References**	- A'Hern RP. Sample size tables for exact single-stage phase II designs. Stat Med. 2001 Mar 30;20(6):859-66.<br>- Aghajanian, C. *et al.* OCEANS: a randomized, double-blind, placebo-controlled phase III trial of chemotherapy with or without bevacizumab in patients with platinum-sensitive recurrent epithelial ovarian, primary peritoneal, or fallopian tube cancer. *J. Clin. Oncol. Off. J. Am. Soc. Clin. Oncol.* 30, 2039–2045 (2012).<br>- Ayers M, Lunceford J, Nebozhyn M, Murphy E, Loboda A, Kaufman DR, Albright A, Cheng JD, Kang SP, Shankaran V, Piha-Paul SA, Yearley J, Seiwert TY, Ribas A, McClanahan TK. IFN-γ-related mRNA profile predicts clinical response to PD-1 blockade. J Clin Invest. 2017 Aug 1;127(8):2930-2940<br>- Burger, R. A. *et al.* Incorporation of bevacizumab in the primary treatment of ovarian cancer. *N. Engl. J. Med.* 365, 2473–2483 (2011).<br>- Chen, P.-L. *et al.* Analysis of Immune Signatures in Longitudinal Tumor Samples Yields Insight into Biomarkers of Response and Mechanisms of Resistance to Immune Checkpoint Blockade. *Cancer Discov.* 6, 827–837 (2016).<br>- Dean, E. *et al.* Phase I study to assess the safety and tolerability of olaparib in combination with bevacizumab in patients with advanced solid tumors. *Br. J. Cancer* 106, 468–474 (2012).<br>- Hegde, P. S., Karanikas, V. & Evers, S. The Where, the When, and the How of Immune Monitoring for Cancer Immunotherapies in the Era of Checkpoint Inhibition. *Clin. Cancer Res.* 22, 1865–1874 (2016).<br>- Jayson, G. C., Kohn, E. C., Kitchener, H. C. & Ledermann, J. A. Ovarian cancer. *The Lancet* 384, 1376–1388 (2014).<br>- Larkin, J. *et al.* Combined Nivolumab and Ipilimumab or Monotherapy in Untreated Melanoma. *N. Engl. J. Med.* 373, 23–34 (2015).<br>- Le, D. T. *et al.* PD-1 Blockade in Tumors with Mismatch-Repair Deficiency. *N. Engl. J. Med.* 372, 2509–2520 (2015).<br>- Ledermann, J. *et al.* Olaparib maintenance therapy in patients with platinum-sensitive relapsed serous ovarian cancer: a preplanned retrospective analysis of outcomes by BRCA status in a randomised phase 2 trial. *Lancet Oncol.* 15, 852–861 (2014).<br>- Lee JM, Cimino-Mathews A, Peer CJ, Zimmer A, Lipkowitz S, Annunziata CM, Cao L, Harrell MI, Swisher EM, Houston N, Botesteanu DA, Taube JM, Thompson E, Ogurtsova A, Xu H, Nguyen J, Ho TW, Figg WD, Kohn EC. Safety and Clinical Activity of the Programmed Death-Ligand 1 Inhibitor Durvalumab in Combination With Poly (ADP-Ribose) Polymerase Inhibitor Olaparib or Vascular Endothelial Growth Factor Receptor 1-3 Inhibitor Cediranib in Women's Cancers: A Dose-Escalation, Phase I Study. J Clin Oncol. 2017 Jul 1;35(19):2193-2202.<br>- Liu, J. F. *et al.* Combination cediranib and olaparib versus olaparib alone for women with recurrent platinum-sensitive ovarian cancer: a randomised phase 2 study. *Lancet Oncol.* 15, 1207–1214 (2014).<br>- Martincorena, I. & Campbell, P. J. Somatic mutation in cancer and normal cells. *Science* 349, 1483–1489 (2015).<br>- Mony, J. T. *et al.* Anti-PD-L1 prolongs survival and triggers T cell but not humoral anti-tumor immune responses in a human MUC1-expressing preclinical ovarian cancer model. *Cancer Immunol. Immunother. CII* 64, 1095–1108 (2015).<br>- Murai, J. *et al.* Differential trapping of PARP1 and PARP2 by clinical PARP inhibitors. *Cancer Res.* 72, 5588–5599 (2012).

- Oza, A. M. *et al.* Olaparib combined with chemotherapy for recurrent platinum-sensitive ovarian cancer: a randomised phase 2 trial. *Lancet Oncol.* 16, 87–97 (2015).
- Patel, A. G., Sarkaria, J. N. & Kaufmann, S. H. Nonhomologous end joining drives poly(ADP-ribose) polymerase (PARP) inhibitor lethality in homologous recombination-deficient cells. *Proc. Natl. Acad. Sci. U. S. A.* 108, 3406–3411 (2011).
- Perren, T. J. *et al.* A phase 3 trial of bevacizumab in ovarian cancer. *N. Engl. J. Med.* 365, 2484–2496 (2011).
- Pujade-Lauraine, E. *et al.* Bevacizumab combined with chemotherapy for platinum-resistant recurrent ovarian cancer: The AURELIA open-label randomized phase III trial. *J. Clin. Oncol. Off. J. Am. Soc. Clin. Oncol.* 32, 1302–1308 (2014).
- Rizvi, N. A. *et al.* Cancer immunology. Mutational landscape determines sensitivity to PD-1 blockade in non-small cell lung cancer. *Science* 348, 124–128 (2015).
- Wallin, J. J. *et al.* Atezolizumab in combination with bevacizumab enhances antigen-specific T-cell migration in metastatic renal cell carcinoma. *Nat. Commun.* 7, 12624 (2016).
- Wolchok, J. D. *et al.* Guidelines for the evaluation of immune therapy activity in solid tumors: immune-related response criteria. *Clin. Cancer Res. Off. J. Am. Assoc. Cancer Res.* 15, 7412–7420 (2009).

TABLE OF CONTENTS

REVISION CHRONOLOGY PAGE 4
PROTOCOL SUMMARY 5
TABLE OF CONTENTS 19
LIST OF ABBREVIATIONS AND DEFINITIONs OF TERMS 24
1. BACKGROUND 25
1.1. Background 25
1.1.1. Epidemiology 25
1.1.2. Standard therapy for ovarian advanced cancer 25
1.1.3. Rationale for new approaches in advanced ovarian cancer 25
1.2. Immunotherapy for ovarian cancer 26
1.2.1. OC is an immunogenic tumor: evidence 26
1.2.2. The PD-L1/PD1 immunosuppressive pathway in Ovarian Cancer 26
1.2.3. Immune checkpoint inhibitors: anti-PD1 & anti-PD-L1 in Ovarian Cancer 27
1.3. Bevacizumab in monotherapy 27
1.3.1. Mechanism of action 28
1.3.2. Pre-clinical experience 28
1.3.3. Clinical experience 28
1.4. Olaparib in monotherapy 30
1.4.1. Mechanism of action 30
1.4.2. Pre-clinical experience 30
1.4.3. Clinical experience 30
1.5. Durvalumab in monotherapy 34
1.5.1. Mechanism of action 35
1.5.2. Pre-clinical experience 36
1.5.3. Clinical experience 36
1.6. Use of bevacizumab, olaparib or durvalumab in combination 36
1.7. Rationale for BOLD design 36
1.7.1. Rationale for combining bevacizumab, olaparib and durvalumab 37
1.7.2. Rationale for the study design 39
1.7.3. Rationale for population 39
1.7.4. Rationale for dose and formulation 39
1.8. Benefit/risk assessment and ethical assessment 42
1.8.1. Olaparib benefit/risk in monotherapy and combination therapy 42
1.8.2. Durvalumab benefit/risk 43
2. STUDY OBJECTIVES 45
2.1. Primary objective 45
2.2. Secondary objectives 45
2.3. Translational research objectives 45

3. STUDY PLAN AND PROCEDURES 46
3.1. Study design 46
3.2. Flow chart 47
3.3. Patient screening and study schedule 49
4. SUBJECT SELECTION CRITERIA 50
4.1. Inclusion criteria 50
4.2. Exclusion criteria 52
4.3. Child-bearing potential definition and birth control methods 55
4.4. Restriction for herbal medication 55
5. STUDY TREATMENT 56
5.1. Identity of investigational products 56
5.2. Formulations, packaging and handling 56
5.2.1. Olaparib 56
5.2.2. FKB238 56
5.2.3. Durvalumab 57
5.3. Labelling 57
5.4. Storage 57
5.4.1. Olaparib 57
5.4.2. FKB238 57
5.4.3. Durvalumab 57
5.5. Dosage and administration 58
5.5.1. Olaparib 58
5.5.2. FKB238 58
5.5.3. Durvalumab 59
5.6. Product accountability 59
5.7. Management of toxicities and dose modification 59
5.7.1. General comments 60
5.7.2. FKB238 60
5.7.3. Olaparib 61
5.7.4. Durvalumab 65
5.8. Management of investigational product overdose 93
5.9. Treatment completion 93
6. CONCOMITANT AND POST-STUDY TREATMENT(S) 93
6.1. Permitted therapy 93
6.2. Medication that may not be administered 93
6.3. Blood donation 94
6.4. Subsequent therapies for cancer 94
7. PERMANENT DISCONTINUATION FROM STUDY TREATMENT 95
7.1. Permanent discontinuation of Investigational Product 95
7.2. Treatment after discontinuation of study treatment 96

8. STUDY CONDUCT 97
8.1. Patient enrolment 97
8.2. Procedures for inclusion and initiation of investigational product 97
8.3. Procedures for handling patients incorrectly enrolled or initiated on investigational product 97
8.4. Study treatment management 97
8.4.1. Role of the patient 97
8.4.2. Role of the site study staff for the study treatment 98
8.5. Patient withdrawal from study treatment and from study 98
8.6. Study and site discontinuation 98
9. COLLECTION OF STUDY VARIABLES 100
9.1. Recording of data 100
9.2. Data collection at enrolment and follow-up 100
9.3. Enrolment / Screening procedures 100
9.3.1. Assessments and procedures prior to inclusion 100
9.3.2. Tests to be repeated prior treatment start 101
9.3.3. Tests to be done prior treatment start 101
9.4. Assessments during study treatment 101
9.4.1. Assessment during treatment period 101
9.4.2. Assessment every 6 weeks (each 2 cycle ±7 days) until progression or unacceptable toxicity .. 101
9.5. Assessments for treatment discontinuation 102
9.5.1. Treatment discontinuation visit 102
9.5.2. Safety follow-up visit (30 days after last dose administration of study treatment) 102
9.6. Follow up visits 102
9.7. Survival 103
10. STUDY ASSESSMENTS AND PROCEDURES 104
10.1. Tumor assessments 104
10.1.1. Tumor assessment scheduled 104
10.1.2. Imaging modalities 104
10.1.3. Tumor evaluation 104
10.2. Disease specific tumor marker (CA-125) 105
10.3. Physical examination 105
10.4. Vital signs and blood pressure 105
10.5. Laboratory safety assessments 105
10.5.1. Full haematology assessment 106
10.5.2. Coagulation 106
10.5.3. Biochemistry assessment 106
10.5.4. Serum or urine pregnancy test 106
10.5.5. Urinalysis 106
10.6. ECG 107

11. Safety ... 108
11.1. Definition ... 108
11.1.1. Definition of adverse events (AE) ... 108
11.1.2. Definition of serious adverse events (SAE) ... 108
11.1.3. Individual case safety report (ICSR) ... 108
11.1.4. Adverse events of specific interest ... 108
11.1.5. New fact ... 110
11.1.6. Should Not be considered as AE/SAE ... 110
11.2. INVESTIGATOR RESPONSIBILITIES ... 110
- Recording of all AEs (serious or not) ... 110
- Assessment of all cases (AE's seriousness, causality assessment to IMP or non-IMP) ... 110
- Notification of serious AE, AESI and new facts to sponsor within timelines ... 111
- Follow-up of queries within timelines ... 111
11.3. Recording of adverse events ... 111
11.3.1. Time period for collection of adverse events ... 111
11.3.2. Variables ... 111
11.3.3. Intensity of AEs ... 112
11.3.4. Causality assessment ... 113
11.3.5. Adverse event due to lack of efficacy or to worsening disease ... 114
11.3.6. Overdose ... 114
11.3.7. Pregnancy ... 115
11.3.8. Deaths ... 115
11.4. Reporting of serious adverse events ... 115
11.4.1. Initial notification ... 115
11.4.2. SAE follow up ... 116
12. TRANSLATIONAL RESEARCH PROGRAM ... 117
12.1. Rational and objectives ... 117
12.2. Samples for biomarker analysis ... 117
12.2.1. Archival tumor samples ... 117
12.2.2. Tumor sample at disease progression ... 117
12.2.3. Blood samples ... 117
12.2.4. Samples flow shart ... 117
12.3. Handling, Labelling and storage of biological samples before centralisation ... 118
12.4. Shipment of samples for centralisation ... 118
12.5. Translational research analysis ... 118
12.6. Samples Future Use ... 119
13. ETHICAL AND REGULATORY REQUIREMENTS ... 120
13.1. Ethical conduct of the study ... 120
13.2. Patient data protection ... 120

13.3. Ethical and regulatory review 120
13.4. Informed consent 121
13.5. Changes to the protocol and informed consent form 121
13.6. Audit and inspections 121
14. STUDY MANAGEMENT 122
14.1. Pre-study activities 122
14.2. Training of study site personnel 122
14.3. Monitoring of the study 122
14.4. Study agreements 122
14.5. Study timetable 123
15. DATA MANAGEMENT 124
16. STATISTICAL METHODS AND SAMPLE SIZE DETERMINATION 125
16.1. Statistical considerations 125
16.1.1. Sample size determination 125
16.1.2. Decision rule 125
16.2. Definition of population 126
16.2.1. Intent-to-Treat Population 126
16.2.2. Per Protocol Population 126
16.2.3. Population analysis 126
16.3. Outcome measures 126
16.3.1. Primary outcome measure 126
16.3.2. Other secondary outcome measures 126
16.4. Statistical Analyses 126
16.4.1. Primary Outcome 127
16.4.2. secondary outcomes 127
16.4.3. Exploratory analyses 128
17. STUDY COMMITTEES 130
17.1. Independant Data Monitoring Committee (IDMC) 130
17.2. Steering Committee 130
18. PUBLICATIONS 131
19. MEDICAL EMERGENCIES AND CONTACTS 132
20. List of references 133
21. Appendices 140

LIST OF ABBREVIATIONS AND DEFINITIONS OF TERMS

The following abbreviations and special terms are used in this clinical study protocol:

LIST OF ABBREVIATION OR SPECIAL TERM	EXPLANATION
AE	Adverse Event
AESI	Adverse Event of Specific Interest
ARCAGY	Association de Recherche sur les CAncers dont GYnécologiques
Baseline	Refers to the most recent assessment of any variable prior to dosing with study treatment
CA-125	Cancer Antigen – 125
CRF	Case Report Form (electronic/paper)
CRO	Clinical Research Organization
CT	Computed Tomography
CTC / CTCAE	Common Terminology Criteria for Adverse Event
EC	Ethics Committee, synonymous to Institutional Review Board (IRB) and Independent Ethics Committee (IEC)
GCP	Good Clinical Practice
GINECO	Groupe d'Investigateurs Nationaux pour l'Étude des Cancers Ovariens et du sein
GMP	Good Manufacturing Practice
GINECO	Groupe d'Investigateurs Nationaux pour l'Étude des Cancers Ovariens et du sein
GMP	Good Manufacturing Practice
HCC	Hepatocellular Carcinoma
ITT	Intent To Treat
Ir RECIST	Immune-related Response Criteria Derived from RECIST v.1.1
MAP	Managed Access Program
MedDRA	Medical Dictionary for Regulatory Activities
MRI	Magnetic Resonance Imaging
NE	Not Evaluable
NED	No Evidence of Disease
NTL	Non-Target Lesions
OC	Ovarian Cancer
ORR	Objective Response Rates
OS	Overall Survival
PD	Progressive Disease
PFS	Progression Free Survival
PI	Principal Investigator
PP	Per Protocol
PRR	Platinum resistant relapse
PSR	Platinum sensitive relapse
QoL	Quality of Life
SAP	Statistical Analysis Plan

_

1. BACKGROUND

1.1. Background

1.1.1. Epidemiology

Ovarian cancer (OC) is the 7th most common cancer in women and is responsible of 152.000 deaths worldwide annually. The lifetime risk of developing ovarian cancer is 1-2% and the risk of dying of this disease is around 1%, with a five-year survival rate of less than 45%. The overall incidence worldwide has been estimated at 5/100000, increasing with age and peaking in the late 70s (1-2bis). In more than 70% of the patients, the diagnosis of ovarian cancer occurs at late stages and the survival rate is less than30%. In the few cases of localized tumor at the time of the diagnosis, the survival rate at 5 years increases up to 90%. More than 90% of ovarian cancers originate from epithelial cells, 5% from stromal cells and less than 5% have a germ cells origin. The incidence of OC increases with age and is most prevalent in the eighth decade of life. More than 70% of the patients are diagnosed with advanced disease and less than 40% of women with OC are cured.

1.1.2. Standard therapy for ovarian advanced cancer

The initial treatment of choice for ovarian cancer is surgery, followed by adjuvant chemotherapy. However, in the cases where the patients do not fit for surgery or in cases where the tumor is unresectable (stage FIGO IIIC-IV), a neo-adjuvant chemotherapy is proposed and a surgical tumor debulking is done after the first 3-4 cycles of chemotherapy.

First line of adjuvant chemotherapy as well as neo-adjuvant chemotherapy includes a combination of cisplatin or carboplatin (AUC 5-6) and paclitaxel intravenously, every 3 weeks. Although surgery and chemotherapy can be curative in patients with early stage disease, most of the patients are diagnosed at an advanced stage of and they will relapse. The median PFS of ovarian cancer is 18 months. The first relapse is usually platinum sensitive and the treatment of choice is still a combination of platinum and paclitaxel, or gemcitabine, or doxorubicin and this until patients become platinum resistant. The resistance is defined by a progression within the 6 months after the end of the last platinum-containing regimen. In this case, the chance of response to a platinum containing regimen is less than 15% (3).

Relapsing ovarian cancer is no longer a curative disease in most cases and there is a need in developing new therapeutic strategies and in improving massive molecular screening in order to select the most appropriate treatment especially for patients that are not responding anymore to platinum containing regimens.

1.1.3. Rationale for new approaches in advanced ovarian cancer

At the time of relapse, prediction of chemotherapy efficacy and patient's survival are dependent of the length of the platinum-free interval, defined as the interval between the date of the last administration of platinum and the date of relapse. Early relapse (or platinum-resistant relapse) and late relapse (or platinum-sensitive relapse) occur between one month and 6 months and over 6 months from the last dose of platinum, respectively.

Whereas single non-platinum agent is considered as standard in platinum-resistant disease, standard chemotherapy for platinum-sensitive relapse (PSR) consists of carboplatin combinations either with gemcitabine, paclitaxel (ICON4) or pegylated liposomal doxorubicin (PLD) (4). Also in PSR, several biologic therapies, when sequentially added to carboplatin-based chemotherapy, have been shown to delay disease

progression compared to chemotherapy alone. Olaparib, the first in class anti-PARP agent, has been recently registered in the European Union (EU) in the PSR setting, but only for a subpopulation of patients with germinal or somatic BRCA mutation who have a platinum-sensitive relapse of a high grade serous OC which is still responding to a platinum-based chemotherapy (5). In contrast, bevacizumab has been registered in EU for patients with PSR whatever the BRCA status and the histology when patients are treated with the carboplatin-gemcitabin regimen. Bevacizumab is an anti-VEGF monoclonal antibody targeting blood vessels of the malignant tumor microenvironment and was found to be particularly successful in the treatment strategy of OC.

1.2. Immunotherapy for ovarian cancer

1.2.1. OC is an immunogenic tumor: evidence

There is evidence that OC is an immunogenic tumor that can be recognized by the host system. In peripheral blood, ascites and tumor of approximately half of OC patients can be detected spontaneous antitumor immune response by antibodies and oligoclonal T-cells which recognize autologous tumor-associated antigens (TAAs) (6-8). Some TAAS have been studied in OC, such as New York-esophageal-1 (NY-ESO-1), p53, HER2/neu, survivin, sperm surface protein Sp17, folate receptor-alpha, melanoma associated antigen 3 (MAGE 3) and human telomerase reverse transcriptase (hTRT) (9). OC, however, exhibit an extreme degree of heterogeneity of TAAs with an average of 60 private nonsynonymous mutations per tumor which are rarely shared among different tumors.

Within the tumor, spontaneous antitumor immune response has been demonstrated but only in approximately 55% of the patients with OC in the form of intraepithelial tumor-infiltrating lymphocytes (TILs) (10). The presence of epithelial tumor-infiltrating T cells has been repeatedly associated with a prolonged survival among ovarian cancer patients (11). If the immune system is able to target OC tumors and influence survival, ovarian cancer cells can also exploit several mechanisms to evade immunologic elimination. Immune evasion mechanisms includes the recruitment of immunosuppressive cells such as regulatory T cells (12-13), tumor-associated macrophages (TAMs) (14), or immature dendritic cells which have been correlated with poor survival in this patient population. The immunosuppressive environment is further augmented in OC by the expression of T cell inhibitory receptors on tumor cells and immune cells.

1.2.2. The PD-L1/PD1 immunosuppressive pathway in Ovarian Cancer

T-cell activation requires a primary signal through T-cell receptor (TCR) and cognate antigen complex with Major Histocompatibility Complex (MHC), and a secondary signal, such as costimulatory CD28. This activation is regulated by inhibitory receptors, such as cytotoxic T lymphocyte-associated protein 4 (CTLA-4) and programmed death-1 (PD-1). The interaction of PD-1 with its ligand PD-L1 highly suppresses anti-tumor cytotoxic T-cells. PD-1 receptor molecules can be expressed on CD8+ and CD4+ T-cells (including Tregs), whereas programmed death ligand 1 (PD-L1) is expressed on activated T-cells, tumor-infiltrating macrophages & fibroblasts and cancer cells.

Many human tumors have been found to overexpress PD-L1, which acts to suppress anti-tumor immunity. PD-1 is an inhibitory receptor expressed on T cells following T-cell activation, which is sustained in states of chronic stimulation, such as in chronic infection or cancer (15-16). Ligation of PD-L1 with PD-1 inhibits T-cell proliferation, cytokine production, and cytolytic activity, leading to the functional inactivation or exhaustion of T cells. B7.1 is a molecule expressed on antigen-presenting cells and activated T cells. PD-L1 binding to B7.1 on T cells and antigen-presenting cells can mediate the downregulation of immune responses, including

inhibition of T-cell activation and cytokine production (17-19).

Aberrant expression of PD-L1 on tumor cells has also been reported to impede anti-tumor immunity, resulting in immune evasion (20).

Though data remains scarce, high IHC PD-L1 expression (score 2 & 3) has been detected in 68% of ovarian cancer patients (n=70) and that expression of PD-L1 had a strong prognostic value (21). The authors found also that the density of intraepithelial CD8+ T cells was inversely correlated to expression of PD-L1 by tumors, suggesting that the expression of PD-L1 on tumor cells may inhibit invasion of tumor epithelium by CD8+ T cells.

In addition, PD-1 expression at the surface of intra-tumoral CD4+ FOXP3+ Tregs was found to show the highest levels in ovarian cancer (around 20% of the cells) compared to other tumor types, including melanoma, renal cell cancer or hepatoma (22). Thus Targeting PD-1/PD-L1 pathway may inhibit Treg expression, one of the major component of ovarian cancer immunosuppression. Also Curiel et al showed that myeloid dendritic cells (MDCs) from ovarian cancer express PD-1 and that blockade of PD-1 enhanced MDC-mediated T-cell activation, including upregulation of IL-2 andinterferon-gamma, and down regulation of IL-10, which resulted in enhanced T-cell immunity against autologous ovarian human tumors into NOD-SCID mice. (23) Together with the aforementioned data on immune infiltration, these data provide rationale for a therapeutic PD-1/PD-L1 pathway blockade in ovarian cancer.

1.2.3. Immune checkpoint inhibitors: anti-PD1 & anti-PD-L1 in Ovarian Cancer

Interruption of the PD-L1/PD-1 pathway represents an attractive strategy to reinvigorate tumor-specific T-cell immunity. Recently, monoclonal antibodies (mAbs) targeting checkpoint immune molecules expressed at the surface of immune cells such as CTLA4, PD-1 and PD-L1 have been developed. These mAbs targeting checkpoint immune molecules have shown activity first in metastatic melanoma (24-27). More recently, they also have demonstrated significant efficacy in other types of cancers such as lung (28), renal cell or bladder Cancer (29-30).

In ovarian carcinoma patients, the anti-PD1 compound nivolumab has been reported to achieve 3 objective responses out of 13 (23%) heavily pre-treated patients (Hamanishi J, ASCO 2014). Response was prolonged over 1 year in 2 out of the 3 responders (30bis).

Similarly, the anti-PD1 pembrozilumab achieved 3 confirmed responses (11.5% [(95% CI, 2.4-30.2]) in 26 patients treated in a phase IB study and 3 additional patients had a tumor reduction of at least 30%. Most common AEs were fatigue (42.3%), anemia (30.8%), and decreased appetite (30.8%). Drug-related AEs occurred in 69.2% of pts (grade ≥ 3, 1/26 pts).

The anti-PD-L1 avelumab has reported a 10.7% objective response and a 44% stabilization rate in 75 patients with ovarian cancer in relapse (31). In this study, confirmed or unconfirmed responses (n=11) tend to be more frequently observed in patients with low burden of tumor, limited number of prior lines of chemotherapy and in the setting of platinum-sensitivity. Toxicity was minimal. Considering all grades, fatigue was observed in 16% of the patients, chills in 12%, nausea in 10.7%, diarrhea in 10.7%, rash in 8% and hypothyroidism in 5.3%. Only 6 patients experienced treatment-related CTCAE grade 3 toxicity with none occuring in more than one patient (peripheral edema, localized edema, tumor pain, arthritis, myositis, increased level of lipase, CPK, glycemia and anemia).

1.3. Bevacizumab in monotherapy

Ovarian cancer is among the tumors expressing the highest levels of hypoxia-inducible factor-1 alpha (HIF-1) and vascular endothelial growth factor VEGF. In humans a direct relationship has been demonstrated between the expression of biomarkers for angiogenesis such as VEGF and VEGF-R, in tumor samples and serum, and the behavior of epithelial ovarian carcinomas. VEGF is highly expressed in the ascites of women with advanced ovarian cancer and several studies demonstrated a direct relationship between VEGF levels and prognosis, especially when measured on ascites, since the peritoneal cavity represents the site of the most frequent and aggressive spread of the ovarian cancer (32-33).VEGF levels in ascites were inversely correlated with platinum sensibility and lower levels of VEGF were associated to an increase in PFS and OS (33).

1.3.1. Mechanism of action

Bevacizumab is a monoclonal antibody specifically designed to bind and inhibit circulating VEGF. In first-line treatment following optimal surgical debulking 2 phase III trials, GOG0218 (34) and ICON7 (35) demonstrated that bevacizumab in combination with standard chemotherapy (carboplatin and paclitaxel), followed by the continued use of bevacizumab alone, significantly increased progression free survival (PFS) compared to chemotherapy alone. Since 2011 bevacizumab has been approved in EU when combined with carboplatin and paclitaxel and is indicated for the front-line treatment of adult patients with advanced (International Federation of Gynecology and Obstetrics [FIGO] stages III B, III C and IV) epithelial ovarian, fallopian tube, or primary peritoneal cancer.

1.3.2. Pre-clinical experience

The pre-clinical experience is fully described in the current version of the bevacizumab Investigator Brochure.

In 1993, Ferrara et al. reported that a murine anti-human VEGF monoclonal antibody called A.4.6.1 exerted a potent inhibitory effect on the growth of several tumor cell lines in nude mice, while the antibody had no effect on the tumor cells in vitro (36). Subsequently, many other tumor cell lines were shown to be inhibited in vivo by the same anti-VEGF monoclonal antibody (37-40). Tumor growth inhibition has been demonstrated by numerous laboratories using other anti-VEGF antibodies or several anti-VEGF approaches, including a retrovirus-delivered dominant negative Flk-1 mutant (41), small molecule inhibitors of VEGFR-2 signaling (41-42), antisense oligonucleotides targeting VEGF (43), and soluble VEGF receptors (44).

Bevacizumab inhibits the growth of human tumor cell lines in nude mice, achieving a maximal inhibition at the dose of 1–2 mg/kg twice weekly (45). Half-maximal inhibition required 0.1–0.5 mg/kg doses. The magnitude of the inhibition is inversely related to the content of stromal-derived mouse VEGF within the tumor xenograft. In tumors with high human/mouse VEGF ratio, the inhibition can exceed 90% (46).

Safety evaluation studies of bevacizumab were conducted in Macaca fascicularis (cynomolgous monkey)(47). Bevacizumab is expected to be pharmacologically active in this species, considering the complete identity between human and cynomolgous VEGF isoforms at the protein level (48). Following administration of bevacizumab, young adult cynomolgus monkeys exhibited a physeal dysplasia characterized by increase in hypertrophied chondrocytes and inhibition of vascular invasion of the growth plate, very similar to the growth plate lesion observed in mice treated with Flt(1-3)-IgG (49). Other expected effects of prolonged bevacizumab administration were suppression of angiogenesis in the female reproductive tract, resulting in decreased ovarian and uterine weights and an absence of corpora lutea (50).

1.3.3. Clinical experience

The assessment of safety and efficacy of bevacizumab added to standard chemotherapy in first-line treatment

following optimal surgical debulking was derived from the 2 phase III trials, GOG0218 6 (51) and ICON7 7 (52). These trials were conducted in women with newly diagnosed OC and demonstrated that front-line bevacizumab in combination with standard chemotherapy (carboplatin and paclitaxel), followed by the continued use of bevacizumab alone, significantly increased progression free survival (PFS) compared to chemotherapy alone.

The GOG0218 study involved 1,873 women with AOC who were randomized between 3 arms of treatment: the control arm with chemotherapy alone, the bevacizumab-initiation arm in which bevacizumab was administered concomitantly to chemotherapy and the bevacizumab-throughout arm in which patients received bevacizumab (15mg/kg q21d) in combination with chemotherapy (paclitaxel and carboplatin), and pursed bevacizumab alone for a total duration of up to 15 months. In the bevacizumab-throughout arm (n=623), patients had a median PFS of 14.1 months compared to 10.3 months in women who received chemotherapy alone (hazard ratio = 0.72, p<0.0001). Disease progression in the GOG0218 was measured using both levels of CA-125 (GCIC criteria) and imagery (RECIST criteria): a sensitivity analysis was conducted including only disease progressions determined by radiographs/scans and excluding progressions based on CA-125 alone. In this analysis, women who continued bevacizumab had a median PFS of 18.0 months compared to 12.0 months in women who received chemotherapy alone (hazard ratio = 0.64, p<0.001).

In the ICON7 trial, 1,528 women with FIGO stage I-IV OC were randomized to receive chemotherapy (paclitaxel and carboplatin) alone or in combination with bevacizumab (7.5mg/kg q21d) followed by bevacizumab alone for a total duration of up to 12 months. The median PFS in the bevacizumab arm was 19 months compared to 17.3 months in women who received chemotherapy alone (hazard ratio = 0.79, p = 0.004).

The assessment of safety and efficacy of bevacizumab added to standard chemotherapy has also been explored in patients with relapsing ovarian cancer. In 2014, bevacizumab plus chemotherapy was approved in EU and US in combination with paclitaxel, pegylated liposomal doxorubicin (PLD), or topotecan, for the treatment of patients with platinum-resistant recurrent ovarian cancer. This approval was based on results of the AURELIA study, which randomized patients to physician's choice of chemotherapy (PLD, paclitaxel, or topotecan) with or without bevacizumab, and included patients with more than one prior line of platinum based chemotherapy. This study demonstrated improvement in median progression-free survival (PFS) from 3.4 months to 6.7 months (Hazard Ratio [HR=0.48], 95% confidence interval [CI]: 0.38 to 0.60, p<0.001). No statistically significant improvement in OS was seen. (53). Moreover, in a subsequent analysis based on each individual chemotherapy cohort, the combination of paclitaxel and Bevacizumab gave the best ORR by RECIST (difference Bevacizumab plus chemotherapy versus chemotherapy alone of 23.1% in the paclitaxel group compared to 17% in the topotecan cohort and 13.7% in the PDL cohort) (54).

A total of 3 trials have been run to assess the combination of bevacizumab with standard carboplatin-based combinations in patients with platinum-sensitive relapse (PSR). The OCEANS trial was conducted in 484 patients with PSR, no prior bevacizumab therapy and measurable disease. Patients were randomly assigned to receive every 3 weeks either carboplatin (AUC4, d1) with gemcitabine (1000mg/m², d1 1 8) or the same chemotherapy plus bevacizumab (15 mg/kg q3 wk) until progression or unacceptable toxicity. Patients treated in the bevacizumab arm had significantly prolonged PFS (12.4 vs 8.4 months) with a HR for relapse of 0.48 (95% CI: 0.39-0.60, , p<0.0001). Objective response rate (ORR) was increased from 57.4% up to 78.5% (p<0.0001) in the bevacizumab arm. There was no significant difference in overall survival (OS). As for toxicity, safety data were consistent with bevacizumab profile with an increase of grade>3 hypertension (17 vs <1%), proteinuria (9 vs 1%), non-CNS bleeding (6 vs 1%) and arterial thromboembolic events 3 vs 1%), without

significant increase in hematotoxicity or bowel perforation. These results from OCEANS trial led to the approval of bevacizumab in EU for patients with first PSR relapse and without previous exposure to anti-angiogenic agents in front line. (55).

Since 2011 bevacizumab has been approved in Europe in combination with carboplatin and paclitaxel and indicated for the front-line treatment of adult patients with advanced (International Federation of Gynecology and Obstetrics [FIGO] stages III B, III C and IV) epithelial ovarian, fallopian tube, or primary peritoneal cancer.

However, despite the progress brought by bevacizumab, the prognostic of patients with AOC remains poor with a median PFS of 14-19 months and a median Overall Survival (OS) at 5 years of 30-35%.

1.4. Olaparib in monotherapy

Olaparib (AZD2281, KU-0059436) is a potent Polyadenosine 5'diphosphoribose [poly (ADP ribose)] polymerization (PARP) inhibitor (PARP-1, -2 and -3) that is being developed as an oral therapy, both as a monotherapy (including maintenance) and for combination with chemotherapy and other anti-cancer agents.

1.4.1. Mechanism of action

The mechanism of action for olaparib results from the trapping of inactive PARP onto the single-strand breaks preventing their repair (56-57). Persistence of SSBs during DNA replication results in their conversion into the more serious DNA DSBs that would normally be repaired by HR repair. Olaparib has been shown to inhibit selected tumor cell lines in vitro and in xenograft and primary explants models as well as in genetic BRCA knock-out models, either as a stand-alone treatment or in combination with established chemotherapies.

1.4.2. Pre-clinical experience

The pre-clinical experience is fully described in the current version of the olaparib Investigator Brochure.

Olaparib has been tested in a standard range of safety pharmacology studies e.g., dog cardiovascular and respiratory function tests, and the rat Irwin test. There were no noticeable effects on the cardiovascular or respiratory parameters in the anaesthetized dog or any behavioral, autonomic or motor effects in the rat at the doses studied. Rodent and dog toxicology studies have indicated that the primary target organ of toxicity is the bone marrow with recovery seen following withdrawal of olaparib. *Ex vivo* studies have confirmed that olaparib is cytotoxic to human bone marrow cells. Olaparib was not mutagenic in the Ames test but was clastogenic in the Chinese hamster ovary (CHO) chromosome aberration test *in vitro*. When dosed orally, olaparib also induced micronuclei in the bone marrow of rats. This profile is consistent with the potential for genotoxicity in man. Reproductive toxicology data indicate that olaparib can have adverse effects on embryofoetal survival and development at dose levels that do not induce significant maternal toxicity. Further information can be found in the current version of the olaparib IB.

1.4.3. Clinical experience

1.4.3.1. Olaparib efficacy: D0810C00019 and D0810C00041 randomized trials

1.4.3.1.1. D0810C00019 study ("Study 19")

The pivotal study D0810C00019 is a randomized, double-blind, placebo-controlled phase II study to evaluate maintenance treatment with olaparib (capsule formulation) in patients with platinum-sensitive relapsed HGSOC who had received ≥2 previous platinum regimens and were in partial or complete response following their last platinum-containing regimen. The primary endpoint was investigator-assessed PFS. In total, 265 patients were randomized to olaparib 400 mg twice daily (136) or placebo (129). The primary analysis was carried out following 153 PFS events and demonstrated that maintenance treatment with olaparib led to a

significant PFS improvement versus placebo [hazard ratio = 0.35 (95% confidence interval (CI) 0.25 - 0.49); p<0.00001] (58). This phase II study D0810C00019 demonstrated the efficacy of olaparib maintenance in platinum sensitive OC relapse.

1.4.3.1.2. D0810C00041 study

Study D0810C00041 (Clinical-trial.gov NCT01081951), is open-label, randomized Phase II in patients with platinum sensitive advanced high grade serous ovarian cancer (59). The primary end point was PFS assessed by an Independent Center Review. In total, 162 patients were randomized to olaparib 200mg twice daily (D1-10) plus placlitaxel 175mg/m² and carboplatin AUC 4 followed by olaparib maintenance (400mg twice daily) versus placlitaxel 175mg/m² and carboplatin AUC 6. The primary analysis was carried out following 102 PFS events and demonstrated that addition of olaparib led to a significant PFS improvement [hazard ratio = 0.51; 97% CI 0.34-0.77; p=0.0012]. This phase II study D0810C00041 was another confirmation of olaparib activity evidence in HGOC.

1.4.3.2. Olaparib safety

1.4.3.2.1. Olaparib exposure in clinical trials

As of 20 August 2014, approximately 2866 patients with ovarian, breast, pancreatic, gastric and a variety of other solid tumours are estimated to have received treatment with olaparib across the dose range 10 mg twice daily to 600 mg twice daily in AstraZeneca-sponsored, investigator-sponsored and collaborative group studies. An analysis of data from 12 monotherapy studies in 975 patients estimated that 16.1% (157/975) of patients had been exposed to olaparib (capsule) for > 12 months, 7.8% [76/975] for > 18 months and 4.2% for > 24 months at the time of database closure for the respective studies. From the available data to date, there is no evidence of any unexpected toxicity following long-term olaparib (capsule) monotherapy exposure.

1.4.3.2.2. Adverse events (D0810C00019 study)

The following AEs in the D0810C00019 study were the most common in olaparib treated patients and were reported at a ≥ 10% greater frequency in the olaparib 400 mg twice daily group compared with the placebo group:

- Nausea (70.6% and 35.9% in the olaparib 400 mg twice daily and placebo groups, respectively)
- Fatigue (52.2% versus 39.1%)
- Vomiting (33.8% versus 14.1%)
- Anaemia (21.3% versus 5.5%)

In both treatment groups, the nausea, vomiting, and fatigue were generally mild to moderate (CTCAE grade 1/2), intermittent and manageable on continued treatment. The majority of the CTCAE grade 3/4 hemoglobin decreases were managed through temporary reduction or cessation of olaparib treatment and through blood transfusions.

Eight patients overall had at least 1 AE leading to permanent discontinuation of study treatment. In the olaparib group, 2 AEs that led to permanent discontinuation of study treatment were considered by the investigator to be serious: CTCAE grade 4 small bowel obstruction considered unrelated to olaparib treatment and CTCAE grade 5 hemorrhagic stroke considered related to olaparib treatment. The majority of AEs leading to discontinuation of study treatment resolved after treatment was stopped.

A higher percentage of patients had an AE leading to an interruption or a dose reduction in the olaparib group compared with the placebo group. The AEs that led to interruption or a dose reduction in the olaparib group were generally events known to be associated with olaparib. Vomiting was the most common AE leading to

dose interruption in the olaparib group; abdominal pain, fatigue and small intestinal obstruction was the most common AE leading to dose interruption in the placebo group. Nausea and fatigue were the most common AEs leading to dose reduction in the olaparib group; anemia, vomiting and gastritis (occurring in 1 patient) led to dose reduction in the placebo group.

1.4.3.2.3. Myelodysplastic syndrom /acute myeloid leukemia occurrence in olaparib clinical trials

Development of secondary myelodysplastic syndrome (MDS)/therapy-related acute myeloid leukemia (t-AML) is an AE of interest, as it may be related to products that affect DNA repair mechanisms. As of 20 August 2014, 21 reports of MDS/AML have been received out of 2,866 olaparib treated patients, giving a cumulative reporting rate of 0.7%. Across the clinical study program, MDS has also been reported for 2 patients who did not receive olaparib: one patient that received placebo in Study 19 (0.8% [1/128]) and one patient treated with pegylated liposomal doxorubicin as the comparator in Study 12 (3.1% [1/32]), giving a similar reporting rate. Of the 21 cases reported across the development program, 14 have been reported in monotherapy studies and 7 in combination studies. Sixteen of the patients had a gBRCA mutation. In 13 cases, the diagnosis was MDS without a report of AML. There were 8 cases of AML. The median age of onset was 63 years and all but 3 patients had ovarian cancer. Eight patients had a history of previous cancer. The mean time from diagnosis of current cancer to onset of MDS or AML was 62 months. All patients had associated history features that may have contributed to the development of MDS/AML. All had received chemotherapy with DNA damaging agents, including platinum, taxanes and anthracyclines. Many patients received multiple treatment regimens over multiple years and 7 patients had also received radiotherapy. Four patients were treated with olaparib for less than 6 months, 5 patients were treated for between 6 months and 1 year, 4 patients were treated for between 1 and 2 years, and 8 patients were treated for more than 2 years. In the majority of cases, MDS occurred while on treatment with olaparib but in 4 cases the onset of MDS was more than 5 months after olaparib was discontinued. Epidemiological studies from the literature have indicated a higher risk of therapy related AML in ovarian cancer populations, particularly those receiving alkylating agents and pelvic irradiation with a wide range of incidence rates. In two recent studies using the US SEER database, Vay et al identified 98 cases of t-AML among 63,359 epithelial ovarian cancer cases, with an overall incidence of 0.17% (60), while another SEER-based study in registries representing 9.5% of the US population for years 1975-2008 reported 72 t AML cases among 23,180 ovarian cancer patients (incidence of 0.31%) (61). The SEER data need to be reviewed with caution because MDS is not collected in cancer registries as it is considered pre-malignant and the data from the early episodes are confounded by a higher contemporaneous use of Melphalan, which is no longer prescribed.

While non-clinical data suggest bone marrow progenitor cell populations are reduced temporarily following olaparib treatment, there is no evidence to date linking olaparib treatment to the generation of abnormal bone marrow precursors. Moreover, preclinical data suggest potential benefit with PARP inhibitors in MDS/AML and clinical trials are now underway to assess this effect 19 (62).

1.4.3.2.4. New primary malignancies in olaparib clinical trials

Similar to MDS/AML, the development of new primary malignancies is an adverse event of interest that may be related to products that affect DNA repair mechanisms, and of relevance to patients with germline BRCA mutations, who are at risk of developing other cancers. As of 20 August 2014, 21 of the 2,866 patients who had received olaparib had reported 23 events of a new primary malignancy (other than MDS/AML), giving a cumulative incidence of 0.73% for new primary malignancies. Ten of the events were non-melanoma skin cancers. The remaining events were: breast cancer (n=3), intraductal proliferative breast lesion, lung cancer

(n=2), gastric cancer, plasma cell myeloma, malignant melanoma, precursor T lymphoblastic lymphoma/leukemia, colon cancer, tongue neoplasm and malignant muscle neoplasm (preexisting before olaparib treatment). In addition, one patient in the placebo arm of the double blind Study reported a new primary malignancy event of bladder cancer (1/128, [0.78%]). All patients had already previously received various chemotherapy agents including multiple cycles of DNA damaging platinum containing chemotherapies, taxanes, anthracyclines and other alkylating and DNA damaging agents. Four patients were reported to have had prior radiotherapy. Nineteen patients had a documented breast cancer gene mutation (BRCA 1 or 2). Seven patients had an earlier diagnosis of a previous cancer (ovarian, cervix, breast, peritoneal) prior to their cancer under investigation in the olaparib study. New primary malignancies are monitored actively in the phase III trial both during study treatment and during the long term survival follow up.

1.4.3.3. From capsules to tablets

The initial formulation of olaparib in studies D0810C00019 and D0810C00041 consisted of 8 capsules twice daily delivering 400 mg twice daily.

A more patient friendly tablet formulation (2 tablets twice daily) has been developed. The tablet dose of olaparib that will be investigated in this study is 300 mg twice daily. This tablet dose has been chosen based on data from an ongoing study, D0810C00024. Since it has been shown that the capsule and tablet formulations are not bioequivalent, a formulation switch based on bioequivalence has not been possible. Olaparib, when given via the tablet formulation has a tmax typically between 0.5 and 2 hours and mean terminal half-life of approximately 12 to 15 hours. Based on the average single dose t½, it would be expected that steady state exposure would be achieved within approximately 3 days of commencing dosing with olaparib. It is metabolized primarily by the CYP3A4 enzyme and is excreted through the urine (35% to 50%) and feces (12% to 60%). The tablet dose of 300 mg twice daily is considered to have similar efficacy in terms of tumor shrinkage in BRCA mutated ovarian cancer patients to the 400 mg twice daily capsule together with an acceptable tolerability profile. The tolerability profile of the 300 mg twice daily tablet dose in study D0810C00024 was considered similar to the 400 mg twice daily capsule formulation. The most common adverse events were consistent with the known safety profile of olaparib, namely low grade nausea, vomiting, fatigue and anemia. Further information is provided in the IB.

The results of the food effect study have shown that food (high fat meal) slows the rate of absorption (delays tmax), decreases peak exposures to drug (Cmax) by approximately 21%, but does not affect the extent of absorption (AUC). However, light snack can still be used to reduce nausea/vomiting.

1.4.3.4. Development of olaparib in BRCA 1/2 mutated patients

1.4.3.4.1. Activity of olaparib in BRCA 1/2 mutated patients in the D0810C00019 study

Among the 265 patients included in the D0810C00019 study, 254 (95.8%) were tested for either germline or somatic BRCA mutation and 136 of them (53.5%) were found to have a deleterious BRCA mutation.

The magnitude of the olaparib PFS benefit observed in D0810C00019 study appeared to be even greater in the population of patients with germline (gBRCA1/ 2) or somatic (sBRCA1/ 2) mutation [hazard ratio = 0.18 ; 95% CI 0.10-0.31, median : 11.2 vs 4.3 month ; p< 0.0001] than in the non mutated population (63).

Regarding the overall survival, BRCA mutated patients treated with olaparib had a 3 months median OS prolongation compared to placebo, but this benefit was not significant.

The findings from study D0810C00019 are supported by clinical data from over 350 additional patients with

BRCAm ovarian cancer in six other olaparib trials demonstrating consistent response rates (olaparib IB).

1.4.3.4.2. Safety of olaparib in BRCA 1/2 mutated patients in clinical study

The tolerability profile of olaparib is well characterized and suitable for long-term dosing until disease progression in patients with relapsed platinum sensitive ovarian cancer who carry gBRCAm. The common AEs of olaparib in these patients are similar to the global population and include nausea, vomiting, fatigue and anemia, mainly of low grade. Long-term tolerability to olaparib maintenance therapy has been demonstrated, with 45%, 25% and 17% of the patients in the gBRCA subgroup in study 19 in the olaparib arm remaining on treatment at 1 year, 2 years and 3 years, respectively. Most patients remained on treatment until disease progression, with only a small number of patients permanently discontinuing study treatment due to AEs (9.4% with olaparib vs. 0% with placebo in the gBRCA subgroup). MDS/AML are considered AEs of special interest as they may be related to agents that affect DNA repair, including chemotherapy. These events have been seen in less than 1% of patients who received olaparib. These events will be actively monitored in the ongoing phase III studies, including prompted follow-up.

1.4.3.4.3. Ongoing phase III trials in BRCA mutated patients

The SOLO2 phase III trial investigated the efficacy of olaparib (tablet formulation) administered as maintenance therapy for BRCA 1/ 2 mutated patients with platinum sensitive relapse. Between Sept 3, 2013, and Nov 21, 2014, the investigators enrolled 295 eligible patients who were randomly assigned to receive olaparib (n=196) or placebo (n=99). One patient in the olaparib group was randomised in error and did not receive study treatment. Investigator-assessed median progression-free survival was significantly longer with olaparib (19·1 months [95% CI 16·3–25·7]) than with placebo (5·5 months [5·2–5·8]; hazard ratio [HR] 0·30 [95% CI 0·22–0·41], p<0·0001). The most common adverse events of grade 3 or worse severity were anaemia (38 [19%] of 195 patients in the olaparib group *vs* two [2%] of 99 patients in the placebo group), fatigue or asthenia (eight [4%] *vs* two [2%]), and neutropenia (ten [5%] *vs* four [4%]). Serious adverse events were experienced by 35 (18%) patients in the olaparib group and eight (8%) patients in the placebo group. The most common in the olaparib group were anaemia (seven [4%] patients), abdominal pain (three [2%] patients), and intestinal obstruction (three [2%] patients). The most common in the placebo group were constipation (two [2%] patients) and intestinal obstruction (two [2%] patients). One (1%) patient in the olaparib group had a treatment-related adverse event (acute myeloid leukaemia) with an outcome of death. In march 2018, the CHMP recommended the use of olaparib, whatever the BRCA status (see the CHMP document in appendix 1).

The SOLO1 (study terminated, results pending) phase III trial investigated the efficacy of olaparib (tablet formulation) administered as maintenance therapy in the first line setting for the treatment of newly diagnosed HGOC with advanced BRCA mutation. A total of 344 patients should be accrued.

The primary assessment of PFS will be based on independent central review of objective radiological findings as per the RECIST 1.1 guidelines. A number of secondary endpoints will provide further support for the clinical benefit of olaparib in this patient population, and will include OS, time from inclusion to progression by RECIST v1.1 or CA-125, time from inclusion to second progression (PFS2; see details in the study plan), time from inclusion to first subsequent therapy or death (TFST), time from inclusion to second subsequent therapy or death (TSST), time from inclusion to study treatment discontinuation or death (TDT), and patient reported outcome (PRO) measures.

1.5. Durvalumab in monotherapy

Immune responses directed against tumors are one of the body's natural defenses against the growth and proliferation of cancer cells. However, over time and under pressure from immune attack, cancers develop strategies to evade immune-mediated killing allowing them to develop unchecked. One such mechanism involves upregulation of surface proteins that deliver inhibitory signals to cytotoxic T cells. PD-L1 is one such protein, and is upregulated in a broad range of cancers with a high frequency, with up to 88% expression in some indications. In a number of these cancers, including lung (64) (Mu et al 2011), renal (65-67), pancreatic (68-70), and ovarian cancer (71), tumor cell expression of PD-L1 is associated with reduced survival and an unfavorable prognosis. Along with PD-L1 expression data generated internally, these survival data suggest that an antibody targeting PD-L1 has the potential to affect multiple solid tumor types.

PD-L1 is part of a complex system of receptors and ligands that are involved in controlling T-cell activation. PD-L1 acts at multiple sites in the body to help regulate normal immune responses and is utilized by tumors to help evade detection and elimination by the host immune system tumor response. In the lymph nodes, PD-L1 on antigen-presenting cells binds to anti-programmed cell death 1 (PD-1) or cluster of differentiation (CD)80 on activated T cells and delivers an inhibitory signal to the T cell (72-73). This results in reduced T-cell activation and fewer activated T cells in circulation. In the tumor microenvironment, PD-L1 expressed on tumor cells binds to PD-1 and CD80 on activated T cells reaching the tumor. This delivers an inhibitory signal to those T cells, preventing them from killing target cancer cells and protecting the tumor from immune elimination (74).

PD-L1 is part of a complex system of receptors and ligands that are involved in controlling T-cell activation. PD-L1 acts at multiple sites in the body to help regulate normal immune responses and is utilized by tumors to help evade detection and elimination by the host immune system tumor response. In the lymph nodes, PD-L1 on antigen-presenting cells binds to anti-programmed cell death 1 (PD-1) or cluster of differentiation (CD)80 on activated T cells and delivers an inhibitory signal to the T cell (75-76). This results in reduced T-cell activation and fewer activated T cells in circulation. In the tumor microenvironment, PD-L1 expressed on tumor cells binds to PD-1 and CD80 on activated T cells reaching the tumor.

This delivers an inhibitory signal to those T cells, preventing them from killing target cancer cells and protecting the tumor from immune elimination (74).

Durvalumab is an FDA-approved immunotherapy for cancer, developed by Medimmune/AstraZeneca. It is a human immunoglobulin G1 kappa (IgG1κ) monoclonal antibody that blocks the interaction of programmed cell death ligand 1 (PD-L1) with the PD-1 and CD80 (B7.1) molecules. Durvalumab is approved for the treatment of patients with locally advanced or metastatic urothelial carcinoma who either have disease progression during or following platinum-containing chemotherapy or have disease progression within 12 months of neoadjuvant or adjuvant treatment with platinum- containing chemotherapy. Recently, Durvalumab has been approved by FDA in NSCLC cancer patient (see FDA documents can be found trough the following links:
https://www.accessdata.fda.gov/drugsatfda_docs/appletter/2018/761069Orig1s002ltr.pdf and,
https://www.accessdata.fda.gov/drugsatfda_docs/label/2018/761069s002lbl.pdf.)
Durvalumab is known as a checkpoint inhibitor drug.

1.5.1. Mechanism of action

Nonclinical data with durvalumab suggest that targeting PD-L1 with a biologic agent could be an effective antitumor therapy. Durvalumab is a human monoclonal antibody (mAb) that selectively binds human PD-L1 with high affinity and blocks its ability to bind to PD-1 and CD80. PD-L1 is part of a complex system of receptors and ligands that are involved in controlling T-cell activation. PD-L1 acts at multiple sites in the body to help regulate normal immune responses by delivering inhibitory signals to T cells through the PD-1 and CD80 receptors. Blockade of PD-L1 with durvalumab relieved PD-L1-mediated suppression of human T-cell activation in vitro.

1.5.2. Pre-clinical experience

The pre-clinical experience is fully described in the current version of the durvalumab Investigator Brochure.

In a xenograft model, durvalumab inhibited human tumor growth via a T-cell-dependent mechanism. Moreover, an anti-mouse PD-L1 antibody demonstrated improved survival in a syngeneic tumor model when given as monotherapy and resulted in complete tumor regression in >50% of treated mice when given in combination with chemotherapy. Combination therapy (dual targeting of PD-L1 and CTLA-4) resulted in tumor regression in a mouse model of colorectal cancer. In another combination therapy study, dual targeting of PD-1 and PD-L1 in a syngeneic model β of sarcoma in mice demonstrated statistically significant mean tumor growth delay relative to the control group.

1.5.3. Clinical experience

As of 14 October 2017, 167 clinical studies of durvalumab are ongoing across various tumor indications. Encouraging clinical activity of durvalumab combined with acceptable and manageable safety has been demonstrated across the monotherapy and combination therapy studies. These data provide a sound rationale for further development of durvalumab in advanced solid tumors and hematologic malignancies. Currently, durvalumab is being tested in multiple phase II in monotherapy and in combination trial.

1.6. Use of bevacizumab, olaparib or durvalumab in combination

Bevacizumab and olaparib have already been tested on 12 patients in a phase I trial at their usual doses (10 mg/kg q2w and 400 mg bid – 50 mg capsules -, respectively), and no DLTs were observed (77). The addition of an anti-VEGF small molecule, cediranib, to olaparib doubled the median PFS in a randomized phase II trial in patients with platinum sensitive relapse, with a manageable safety profile(78-79).

A recently reported phase I trial established the RDP2D of Durvalumab and Olaparib – 150 mg tablets –, when given in combination, at 1500 mg every 4 weeks, and 300 mg bid, respectively (80). In addition, the ENGOT/GINECO PAOLA phase III trial is currently evaluating the combination of Olaparib and Bevacizumab as first-line maintenance after platinum-paclitaxel combination, in patients with advanced high-grade serous ovarian carcinoma. Under the hypothesis of a survival benefit in favor of this combination, it would also be of interest to assess the value of adding Durvalumab in order to improve the efficacy of the overall combination.

There are no trials to date assessing anti-VEGF + anti-PARP + anti-PD-1 therapy.

Beside additive efficacy, a synergistic effect could be expected:

- Between bevacizumab and durvalumab, through normalization of blood vessel and potentiation of immunologic infiltration.
- Between olaparib and durvalumab, through cytotoxicity-mediated release of antigens and impairment of mutation repair mechanisms, thereby increasing neoantigen loads.
- Between olaparib and bevacizumab, as already observed with the anti-VEGF cediranib.

1.7. Rationale for BOLD design

The proposed Phase II study is designed to investigate the efficacy and clinical benefit of bevacizumab, olaparib

and durvalumab combination in patients with relapsing advanced high grade ovarian cancer treated with standard treatment strategy

- In platinum sensitive relapse (PSR), whatever the line, in patients previously-treated by a platinum-containing chemotherapy
- In platinum-resistant relapse (PRR), in previously untreated patients for their relapse, or in patients who received a maximum of 1 chemotherapy regimen in this setting

This hypothesis is based upon:

The benefit of olaparib demonstrated for patient with HGOC in the phase II study D0810C00019,

The recent approbation of EMEA for the use of bevacizumab, in combination with carboplatin and paclitaxel and then alone in maintenance, for the first-line treatment (FIGO stages IIIB, IIIC and IV) epithelial ovarian cancer, fallopian tube, or primary peritoneal cancer.

1.7.1. Rationale for combining bevacizumab, olaparib and durvalumab

1.7.1.1. Rationale for combining olaparib and bevacizumab

There are several data suggesting that olaparib and bevacizumab may be synergistic. One mechanism of bevacizumab resistance is hypoxia induction as a response to vessel regression caused by the anti-angiogenic agent, resulting in an increase of DNA damage and genetic instability (81). This observation that tumor cells exposed to chronic hypoxia acquire defects in HR and increased sensitivity to PARP inhibition (82) is an example of 'contextual synthetic lethality' in which hypoxia-induced repair-deficient tumor cells can be targeted by disrupting backup pathways. Therefore, the premise of combining olaparib and bevacizumab is based on the rationale that direct targeting of PARP by olaparib and indirect sensitization to olaparib by acquisition of HR defects by bevacizumab will be therapeutically beneficial. The potential synergy between olaparib and anti-VEGF therapy has been recently supported by the results of randomized phase 2 trial showing that the combination of the antiangiogenic cediranib (30mg daily, per os)) plus olaparib (200 mg capsules twice daily) for the treatment of recurrent platinum-sensitive HGSOC compared to olaparib alone prolonged PFS from a median of 9.0 months up to 17.7 months (hazard ratio = 0.42, 95% CI 0.23-0.76, p=0.005) (J Liu, ASCO 2014, LBA 5500) (NCT 01116648).In addition the response rate of the combination was 80% compared to 48% for the single agent olaparib (p=0.002). However, toxicity of the combination was significant leading dose adaptation in 77% of patients (versus 24% in the single agent arm), with increased occurrence of severe hypertension, fatigue and diarrhea which are previously reported cediranib toxicity. Knowing the favorable toxicity profile of bevacizumab, it might be expected that the combination of olaparib with bevacizumab will have a more tolerable toxicity profile.

Indeed, a recent phase I study established the safety, tolerability and clinical pharmacokinetics of olaparib alone and in combination with bevacizumab. Patients with advanced solid tumors received increasing doses of continuous oral olaparib (100, 200 and 400 mg twice daily. capsule formulation) in combination with bevacizumab (10 mg /kg intravenous q2w). 12 patients were enrolled and received treatment. The most common adverse events (AEs) related to olaparib were grade 1/2 nausea and fatigue. No hematological AEs were reported. No serious AEs related to treatment or dose-limiting toxicities (DLTs) were reported. Three patients discontinued due to AEs, two patients discontinued both olaparib and bevacizumab and one patient discontinued olaparib. Five patients received combination treatment for over 6 months. There was no evidence that bevacizumab did affected olaparib metabolism. The authors have concluded the combination of olaparib 400 mg twice daily (capsule formulation) with bevacizumab 10 mg /kg q2w was generally well tolerated with no DLT and could be considered for future clinical investigation (83).

As of 14 October 2017, 4 clinical studies of bevacizumab in combination with olaparib are ongoing for patients with advanced solid tumor. The French PAOLA study (GINECO) included 900 patients in first-line and 50 % of them received the combination without any major signal of toxicity.

1.7.1.2. Rationale for combining olaparib and durvalumab

Treatment options for patients with ovarian cancer who have already received platinum-based chemotherapy, are limited and frequently require consideration of investigational agents. Molecular selection criteria with the use of an approved cancer therapy (olaparib) targeting DNA damage repair mechanisms will be used to provide a rational therapeutic option, and this agent will be combined with an immunotherapeutic agent to broaden the therapeutic effect of this regimen, based in part on the separate nonoverlapping mechanisms of action of the component agents and on the considerations outlined below.

Inhibition of PARP in sensitive tumor cells, for example those carrying mutations in the BRCA1 or BRCA2 genes, results in accumulating levels of DNA damage and genomic instability, ultimately resulting in cell death (84). Accumulating DNA damage has the potential to modify the immunogenicity of tumors through a number of keymechanisms:

- Triggering of intracellular signalling events that result in the activation of nuclear factor kappa B (NFκB) and interferon regulatory factor 7 (IRF7). These transcriptional regulators result in the increased production of cytokines and chemokines that have the potential to promote antitumor immunity, such as type I IFNs(85) (85).
- Upregulation of surface receptors such as major histocompatibility complex (MHC), ligands for natural-killer group 2, member D (NKG2D) and inducible T-cell costimulatory ligand (ICOSL), which render tumor cells more visible to detection by cytotoxic T cells (86).
- Death of tumor cells and release of antigen, which may help to promote antigen presentation and immune priming (87).

In agreement with this hypothesis, olaparib was associated with a significant improvement in PFS as a maintenance treatment in ovarian cancer (88), and recent analyses suggests this may translate into a survival advantage (89). These effects would be expected to help promote an effective antitumor immune response. In keeping with this hypothesis, several tumor types with genetic defects expected to lead to increased DNA damage show evidence of enhanced immune recognition. For example, BRCAm tumor cells are associated with higher levels of tumor infiltrating lymphocytes and secreting lymphocyte attractants (eg, C-X-C motif ligand [CXCL] 10) and immune suppressive ligands such as PD-L1 (90).

Based on this basic biology, the hypothesis to be tested in this study is that increased DNA damage triggered through PARP inhibition will result in enhanced antitumor immunity that can be further enhanced through combination with an immune checkpoint inhibitor in advanced cancers. This hypothesis is supported by published studies in mouse models of cancer, demonstrating that administration of a PARPi to sensitive tumor types results in increased T-cell infiltration and activation within tumors (91-92). It is also anticipated that durvalumab will prolong the duration of response to olaparib in patients with DNA repair deficient cancers.

As of 14 October 2017, 7 clinical studies of olaparib in combination with durvalumab are ongoing across various tumor indications.

In the study CNIO-BR-008 (Abrogation of chronic monoclonal antibody treatment-induced T-cell exhaustion with durvalumab in advanced HER-2 negative breast cancer: a Pilot Proof-of-concept Trial), Quintela M. and collegues wish to study the efficacy of bevacizumab in combination with durvalumab.

When progression to bevacizumab-alone maintenance treatment occurs, patients will enter the trial and will start receiving durvalumab 750 mg (equivalent to 10 mg/kg Q2W) IV infusion, if ≥ 30 kg, every 2 weeks together

with bevacizumab 15mg/kg IV infusion every 3 weeks. The patients undergo a tumor biopsy before the first dose of durvalumab, and after one month of combined treatment - the blood sampling continue on a monthly basis. The treatment continues until disease progression or for a maximum of 50 weeks. This study is ongoing.

1.7.2. Rationale for the study design

The study design will allow patients to receive proposed bevacizumab biosimilar in combination with olaparib and durvalumab treatment up to disease progression as per normal clinical practice after the enrolment. In this study there is no standard treatment. The use of bevacizumab in combination with olaparib and durvalumab may provide further benefit to patients in terms of prolongation of the progression free interval, increasing the interval between lines of chemotherapy, delaying further hospitalization and the cumulative toxicities associated with chemotherapy.
The primary endpoint will be progression free survival based on investigators' review of objective radiological findings as per the immune-related response criteria (93). A number of secondary endpoints will provide further support for the pharmacokinetic analysis of olaparib and durvalumab in this patient population.

1.7.3. Rationale for population

Only patients with advanced (FIGO stage IIIb-IV) high grade serous or endometrioïd OC will be selected in this trial. From the data of the Cancer Genome Atlas, about half of the patients with HGSOC are expected to have deficient HR.

The D0810C00019 study has shown that olaparib is highly effective in BRCAm patients [hazard ratio = 0.18; 95% CI 0.10-0.31, median: 11.2 vs 4.3 months; p< 0.0001], but also in HGSOC without germline or somatic BRCA mutation [hazard ratio = 0.54; 95% CI 0.34-0.85; p< 0.0075].

One of the secondary objectives of the study will be to correlate a HRD signature with olaparib efficacy. In the NOVA study (94), Mirza et al showed that the PARP inhibitor niraparib was efficacious in all patients with HGSOC in platinum-sensistive relapse, irrespective of their HRD status. However, patients with BRCA mutations derived the maximal benefit, followed by patients with HRD phenotype, and the patients without any abnormality in DNA-repair had the lowest benefit in term of PFS. In march 2018, the CHMP recommended the use of olaparib, whatever the BRCA status (see the CHMP document in appendix 1).

FIGO stages IIIb-IV are the ones where bevacizumab is indicated in first line treatment of OC according to EMA label.

Patients with platinum-sensitive relapse are those who are expected benefit the most from anti-PD-L1 checkpoint inhibitor treatment (95).

In addition, the population with platinum-sensitive relapse is known to be enriched in patients with germinal and/or tumor BRCA mutation as a consequence of the hypersensitivity of BRCA-mutated ovarian cancer cells to platinum therapy (96). And women with BRCA abnormalities are more likely to have CD8 + TILs compared to those with sporadic cancers (97). The enhanced immunogenicity of BRCA1− tumors has been attributed to a significant enrichment of genes involved in immune response which is linked to the genetic instability arising from impaired DNA repair as a direct result of BRCA dysfunction (98).

1.7.4. Rationale for dose and formulation

1.7.4.1. Olaparib

Use in monotherapy

The recommended olaparib monotherapy tablet dose is 300 mg bid.

Use in combination

Dose selection for this study is based on the results of an independent study, the National Cancer Institute (NCI) Study ESR-14-10366 (hereafter referred to as the "NCI study"). The pertinent portion of this study assessed patients in a standard 3+3 design according to the dose escalation scheme outlined below (Table 1). Olaparib was administered according to the dosing schedule used in monotherapy, and durvalumab was studied at a fixed dose of 10 mg/kg every 2 weeks (Q2W) for the first 2 cohorts. Later in the study, the durvalumab dosing schedule was changed to a fixed dose every 4 weeks (Q4W) schedule. Data from this study forms the basis for the dose selection in the current protocol.

Seven patients have been enrolled into 3 cohorts, as of 20 October 2015:

Dose level	Olaparib dose mg[a]	MEDI4736 dose	Ovarian Cancer, n	Other, n
1	200	10 mg/kg Q2W	2	TNBC (1)
2	300	10 mg/kg Q2W	3	
3	300	fixed dose (1500 mg Q4W)	2	TNBC (1)

Q2W=every 2 weeks; Q4W=every 4 weeks; TNBC=triple negative breast cancer.

a Administered as tablet bid continuous

Table 1 Enrollment in NCI Study ESR1-14-10366, as of 20 October 2015

Enrollment to Cohort 2 is complete and these patients have completed the safety evaluation period. As shown in Table 2, administration of the MEDI4736/olaparib combination is well tolerated, with the majority of events observed being grade 1 and only occasional instances of grade 2 severity. No instances of grade 3-4 toxicity, and no instances of dose-limiting toxicity under the above-cited criteria, have been observed.

Dose level	AE type[a]	Grade 1	Grade 2	Grade 3-4
1	ALC low	2	1	
1	Creatinine high	1		
1	Fatigue	3		
1	Headache	2		
1	Nausea	1		
1	Neuropathy middle right finger	1		
1	Oral sensitivity	1		
1	Platelets low	1		
1	Proteinuria	1		
1	Queasiness	1		
1	Skin rash	1		
1	Vertigo	1		
2	Abdominal pain	1		
2	ALC low		1	
2	Anemia		1	

Dose level	AE type[a]	Grade 1	Grade 2	Grade 3-4
2	Cramping pubic pain		1	
2	Creatinine high	1		
2	Diarrhea	1[b]		
2	Fatigue	3		
2	GERD	1		
2	Headache	1		
2	Hematuria	1		
2	Nausea	1	1	
2	Platelets low	1		
2	Stomatitis	1		
2	WBC low	1		
	Total	29	5	

ALC=absolute lymphocyte count; GERD=gastroesophageal reflux disease; WBC=white blood cell.

a Of attribution ≥ 'possible' for either durvalumab, olaparib or durvalumab and olaparib

b One additional instance of diarrhea was noted, which was attributed to "other"; relationship to the combination treatment, durvalumab, olaparib, and disease was considered unlikely.

Table 2 Summary of Adverse Events Observed in NCI Study ESR-14-10366, as of 20 October 2015

On the basis of these data, and given that the combination of olaparib and bevacizumab has been shown well tolerated and effective, the dose of olaparib to be used in this study will be 300 mg bid (tablets), in combination with bevacizumab and durvalumab.

1.7.4.2. Durvalumab

A population PK model was developed for durvalumab using monotherapy data from study CD-ON-MEDI4736-1108 (hereafter referred to as Study 1108) (phase I study; N=292; doses=0.1 to 10 mg/kg Q2W or 15 mg/kg every 3 weeks; solid tumors). Population PK analysis indicated only a minor impact of body weight (WT) on the PK of durvalumab (coefficient of ≤0.5). The impact of body WT-based (10 mg/kg Q2W) and fixed dosing (750 mg Q2W) of durvalumab was evaluated by comparing predicted steady-state PK concentrations (5th, median, and 95th percentiles) using the population PK model. A fixed dose of 750 mg was selected to approximate 10 mg/kg (based on a median body WT of approximately 75 kg). A total of 1000 patients were simulated using a body WT distribution of 40 to 120 kg. Simulation results demonstrated that body WT-based and fixed dosing regimens yield similar median steady state PK concentrations with slightly less overall between-patient variability with the fixed dosing regimen.

Similar findings have been reported by others (99-102). Wang and colleagues investigated 12 mAbs and found that fixed and body size-based dosing perform similarly, with fixed dosing being better for 7 of 12 antibodies. In addition, they investigated 18 therapeutic proteins and peptides and showed that fixed dosing performed better for 12 of 18 in terms of reducing the between-patient variability in PK/pharmacodynamics parameters (103).

A fixed dosing approach is preferred by the prescribing community due to ease of use and reduced dosing errors. Given the expectation of similar PK exposure and variability, AstraZeneca considered it feasible to switch to fixed dosing regimens.

Based on an average body WT of 75 kg, it is planned that a fixed dose of 1.12 g Q3W ±2 days durvalumab

(equivalent to 15 mg/kg Q3W) will be administered via intravenous (IV) infusion, in combination with olaparib and bevacizumab. This choice is based on:
- the long half-life of Durvalumab : 17 days, which makes the 1.25gQ4W and 1.12gQ3W equivalent in term of plasma exposure;
- Our wish to preserve patients' quality of life, allowing a single and concomitant administration of Durvalumab and Bevacizumab every 3 weeks.

1.7.4.3. FKB238

FKB238 is under development as a proposed biosimilar product to Avastin (bevacizumab). From the first in human clinical study of FKB238, no SAEs were observed. All the observed treatment-emergent adverse drug reactions were mild or moderate. Data obtained during a range of initial characterisation and nonclinical studies with FKB238 to date do not indicate an additional potential impact on patient safety compared to Avastin. Therefore, it could be expected that similar class effects and a safety profile to Avastin (bevacizumab) will be observed. However, due to the very limited patient exposure to FKB238, it should be noted that unexpected safety issues may arise during clinical development. Additional details are available in the FKB238 Investigator's Brochure.

1.8. Benefit/risk assessment and ethical assessment

1.8.1. Olaparib benefit/risk in monotherapy and combination therapy

In AstraZeneca-sponsored studies and managed access programs (MAP), a total of 4264 patients have received olaparib either as monotherapy (n=3264) or in combination with chemotherapy or other anti-cancer agents (n=1000; including studies where patients received monotherapy and combination therapy sequentially). Many of these combination studies are ongoing.
Olaparib has been given as either monotherapy (an estimated 2327 patients) or in combination with other chemotherapy/anticancer agents (an estimated 1535 patients). Many of these combination studies are ongoing. An estimated 2343 patients have received the capsule formulation of olaparib. Since 2013, most new clinical studies have utilized the tablet formulation which was designed to deliver the therapeutic dose of olaparib in fewer dose units than for the capsule. In march 2018, the CHMP recommended the use of olaparib, whatever the BRCA status (see the CHMP document in appendix 1).
An estimated 3300 patients have received the tablet formulation and 27 patients have received both formulations of olaparib. Approximately 1500 patients have received comparator or placebo across the olaparib development program. Olaparib has demonstrated statistically significant improvement in PFS as a maintenance monotherapy in a phase II study in platinum-sensitive ovarian cancer and statistically significant improvement in PFS in combination with carboplatin and paclitaxel in a phase II study in patients with platinum-sensitive relapsed serous ovarian cancer. Olaparib plus paclitaxel has demonstrated statistically significant improvement in OS, but not PFS, compared with paclitaxel alone in a phase II study in patients with recurrent/metastatic gastric cancer (104). Whilst this did not translate into a statistically significant benefit in OS in a Phase III study (the GOLD study), there was a trend towards a survival advantage in the overall population. Based on this, the inclusion criteria will include all gastric cancer patients rather than only patients with ATM-negative status. In addition, olaparib has demonstrated antitumor activity in non-comparative studies in patients with gBRCA mutated cancers including ovarian, breast, pancreatic, and prostatic.
Olaparib is currently being studied in a phase II study in patients with SCLC. The olaparib capsule formulation was registered for use in the EU and US in December 2014, and Korea in August 2015, as a treatment for

advanced recurrent BRCAm ovarian cancers.
As of 31 March 2015 it is estimated that >750 patients have received commercial material. The recommended olaparib monotherapy capsule dose is 400 mg bid. The recommended olaparib monotherapy tablet dose is 300 mg bid.
Olaparib monotherapy appears to be generally well tolerated across studies up to and including these doses and from the available data to date, there is no evidence of any unexpected toxicity following long-term olaparib (capsule) monotherapy exposure.
An analysis of data from 13 AstraZeneca-sponsored monotherapy studies in 1006 patients with ovarian cancer (634/1006 [63%]) and other non-ovarian solid tumors (372/1006 [37%]) who received olaparib capsule at a range of doses, estimated that 16.0% (161/1006) of patients had been exposed to olaparib (capsule) for >12 months, 8.3% (84/1006) for >18 months and 4.1% for >24 months at the time of database closure for the respective studies. Twenty-one patients (2.1%) had received ≥48 months of olaparib exposure. As of 31 March 2015, 117 patients (117/883 [13%]) from 7 completed AstraZeneca-sponsored monotherapy studies had continued on olaparib treatment post-study closure because they continued to derive treatment benefit. Approximately half (63/117 [54%]) of these patients have received >24 months of olaparib capsule treatment, of which 39 (33%) have received >36 months of treatment and of the 39 patients, 16 (14%) have received >4 years treatment.
Data from phase I dose escalation studies of olaparib in combination with various chemotherapy agents indicated an increase in bone marrow toxicity (anemia, neutropenia, thrombocytopenia) greater than expected if the agents had been administered alone. However, tolerable regimens of a combination of olaparib with carboplatin/paclitaxel, paclitaxel alone, and liposomal doxorubicin have been established, supporting further studies in the combination setting. Administration of olaparib in combination with dacarbazine, topotecan, gemcitabine, cisplatin, carboplatin + paclitaxel or paclitaxel resulted in a lower maximum tolerated dose compared with administration as a monotherapy.
Toxicities considered to be associated with administration of olaparib include hematological effects (anemia, neutropenia, lymphopenia, thrombocytopenia, mean corpuscular volume elevation), decreased appetite, nausea and vomiting, diarrhea, dyspepsia, stomatitis, upper abdominal pain, dysgeusia, fatigue (including asthenia), increase in blood creatinine, headache, and dizziness.
In a small number of patients, pneumonitis, myelodysplastic syndrome (MDS)/acute myeloid leukemia (AML) and new primary malignancies have been reported. Evidence from the whole development program for olaparib does not support a conclusion that there is a causal relationship between olaparib and these events. Pneumonitis and new primary malignancies are important potential risks for olaparib and are being kept under close pharmacosurveillance. Myelodysplastic syndrome (MDS)/acute myeloid leukemia (AML) are important identified risk for Olaparib and are being kept under close pharmacosurveillance.

1.8.2. Durvalumab benefit/risk

Durvalumab has been given to humans as part of ongoing studies as a single drug or in combination with other drugs. To date durvalumab has been given to more than 1800 patients as part of ongoing studies either as monotherapy or in combination with other anti-cancer agents.

The majority of the safety and efficacy data currently available for durvalumab are based on the first-in-human, single-agent study (Study 1108) in patients with advanced solid tumors. Overall, 456 of 694 patients treated with durvalumab 10 mg/kg Q2W were evaluable for response. Evaluable patients were defined as having ≥24 weeks follow-up, measurable disease at baseline, and ≥1 follow-up scan or discontinued because

of disease progression or death without any follow-up scan. In PD-L1 unselected patients, the overall response rate (ORR), based on investigator's assessment per RECIST 1.1, ranged from 0% in uveal melanoma to 20.0% in bladder cancer, and DCR at 24 weeks ranged from 4.2% in triple-negative breast cancer to 39.1% in advanced cutaneous melanoma. PD-L1 status was known for 383 of the 456 response evaluable patients. Across the PD-L1-positive tumors, ORR was highest for bladder cancer, advanced cutaneous melanoma, and hepatocellular carcinoma (33.3% each); non-small cell lung cancer (NSCLC; 26.7%); and squamous cell carcinoma of the head and neck (SCCHN; 18.2%). Moreover, in the PD-L1-positive subset, DCR at 24 weeks was highest (>10%) in advanced cutaneous melanoma (66.7%), NSCLC (36.0%), hepatocellular carcinoma and bladder cancer (33.3% each), and SCCHN (18.2%). The recent "PACIFIC" trial shows a favorable efficacy/safety profile in NSCLC cancer patient (105).

Monoclonal antibodies directed against immune checkpoint proteins, such as PD-L1, aim to boost endogenous immune responses directed against tumor cells. By stimulating the immune system however, there is the potential for adverse effects on other tissues. Most adverse drug reactions seen with the immune checkpoint inhibitor class of agents are thought to be due to the effects of inflammatory cells on specific tissues. Potential risks are events with a potential inflammatory mechanism and which may require more frequent monitoring and/or unique interventions such as immunosuppressants and/or endocrine replacement therapy. Identified risks with durvalumab are diarrhea, increases in transaminases, pneumonitis and colitis, pemphigoid, myasthenia gravis, immune thrombocytopenia. Potential risks include endocrinopathies (hypo- and hyper-thyroidism, hypophysitis and adrenal insufficiency) hepatitis/hepatotoxicity, neurotoxicities, nephritis, pancreatitis, dermatitis, infusion-related reactions, anaphylaxis, hypersensitivity or allergic reactions, and immune complex disease. Further information on these risks can be found in the current version of the durvalumab IB.

In monotherapy clinical studies AEs (all grades) reported very commonly (≥ 10% of patients) are fatigue, nausea, decreased appetite, dyspnea, cough, constipation, diarrhea, vomiting, back pain, pyrexia, abdominal pain, anemia, arthralgia, peripheral edema, headache, rash, and pruritus. Approximately 10% of patients experienced an AE that resulted in permanent discontinuation of durvalumab and approximately 3.5% of patients experienced an SAE that was considered to be related to durvalumab by the study investigator.

The majority of treatment-related AEs were manageable with dose delays, symptomatic treatment, and in the case of events suspected to have an immune basis, the use of established treatment guidelines for immune-mediated toxicity. A detailed summary of durvalumab monotherapy AE data can be found in the current version of the durvalumab IB.

2. STUDY OBJECTIVES

2.1. Primary objective

The primary objective of this study is to determine the efficacy and tolerance of combining bevacizumab (FKB238)with olaparib and durvalumab in patients with relapse of high grade epithelial ovarian, fallopian tube, or peritoneal cancer (patients with platinum sensitive relapse OR patients with platinum resistant relapse).

- Bevacizumab 15 mg/kg Day 1 Q3W
- Olaparib 300 mg bid po, continuously, starting at D1
- Durvalumab 1.12 g IV on Day 1 Q3W

The primary outcome measure is the rate of clinical and radiological non-progressive disease, based on investigator assessment and using the immune-related response criteria (irRECIST, (106)):

- At 3 months in the PRR cohort
- At 6 months in the PSR cohort

2.2. Secondary objectives

The secondary objectives of this study are to determine:

1. CA 125 decline as expressed by the KELIM parameter
2. Progression free survival (PFS)
3. Overall survival (OS)
4. Tumor response
5. Toxicity as assessed by CTCAE V5.0 scale

2.3. Translational research objectives

The translational research objectives of this study are:

- To correlate olaparib administration and durvalumab efficacy
- To correlate HRD phenotype and response to anti-PARP therapy
- To correlate tumor microenvironnement, immune check point status, and durvalumab response.

3. STUDY PLAN AND PROCEDURES

3.1. Study design

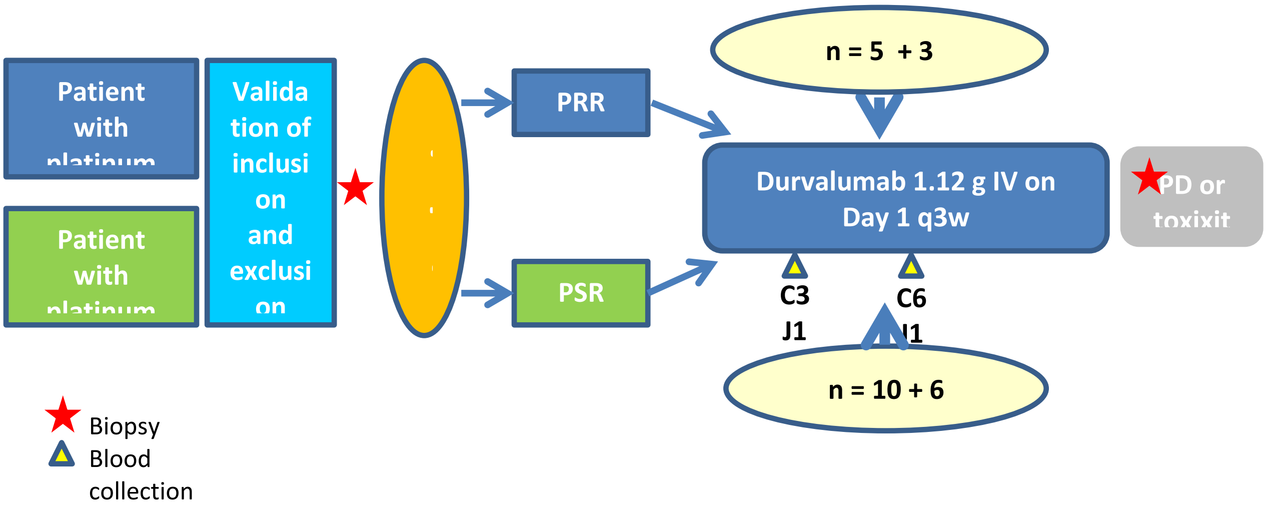

This is a multi-centre, open, non-randomized Phase II trial assessing the safety of the Bevacizumab, Olaparib and Durvalumab combination in patients with advanced epithelial ovarian cancer in relapse.

Approximately 63 patients will be included (23 patients with platinum resisant disease, 40 with platinum sensitive disease) using an Interactive Voice Response System /Interactive web system (IVR/IWR system) as specified below:

Patients will be treated the same way in the PRR and PSR cohorts:

Durvalumab 1.12 g intravenous on Day 1 every 3 weeks
Bevacizumab 15 mg/kg Day 1 every 3 weeks
Olaparib tablets 300mg twice by day per os, continuously

Before inclusion in the study:
A tumor biopsy / surgical sample should have been obtained within 3 months before inclusion and at least 3 months since completion of the last treatment and sent to the central lab.
A blood sample will be collected before treatment, at week 3 and week 6.

Inclusion in the study:
The maximum time relapsed between inclusion and the beginning of treatment is 14 days.

Following inclusion, patients will attend visits according the following flow chart (see section 3.2).

If one cohort reached the number of patient before the other one the recruitment in this cohort will be closed. In such case the other cohort will remain open for recruitment.

Data on tolerance will be collected for assessment of the safety. For the primary endpoint, disease progression will be assessed by investigators per immune-related response criteria (irRECIST) and toxicity will be assessed by the investigators per CTCAE V5.0 scale.

Patients will be monitored until disease progression, intolerable toxicity, elective withdrawal from the study, or study completion or termination.
Patients who discontinue study treatment for reasons other than disease progression (e.g., toxicity) should

continue to undergo scheduled tumor assessments until death, disease progression per iRECIST v1.1, withdrawal of consent, or until the study closes, whichever occurs first. In addition, information regarding use of anti-neoplastic agents for OC after treatment termination will be obtained during the survival followup.

3.2. Flow chart

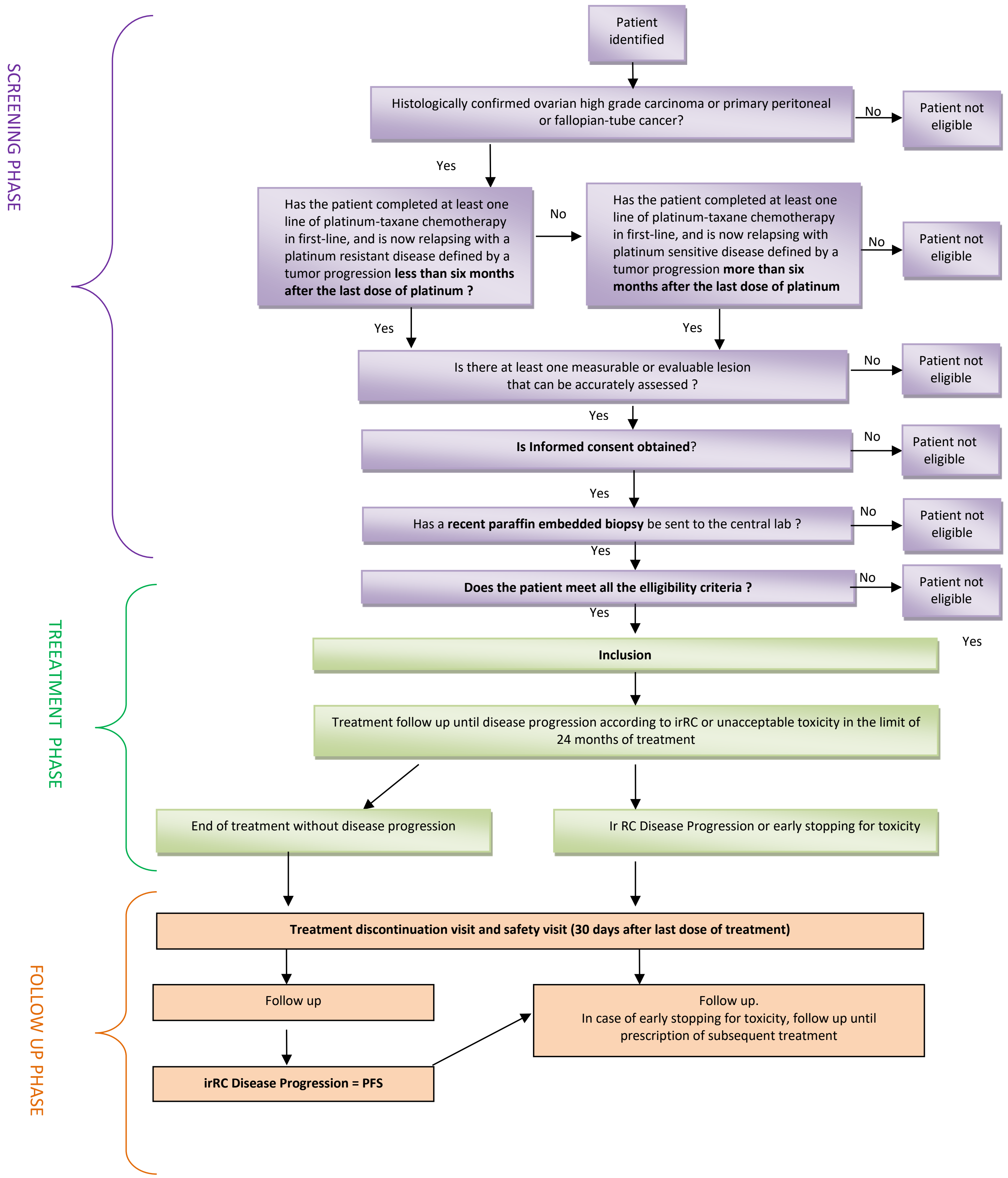

*In case of lost to follow up, overall survival data will be obtained from hospital records and / or public death registries where available.

3.3. Patient screening and study schedule

	VISIT									
**Study period**	**Inclusion**	**C1**	**C1**	**C1**	**C2**	**Cn**	**Assessment visit**	**End of treatment (EoT) visit**	**Safety follow-up visit**	**Follow up after progression or toxicity**
**Timelines**		**D1**	**D8**	**D15**	**D1**	**D1**	**Each 6 weeks ±1 week (2 cycles) until progression**	**Just after last TTT administration**	**30 days ±7 after last TTT administration**	**Each 3 months during 12 months**
**Informed consent**[1]	☑									
**Inclusion / Exclusion criteria**	☑									
**Medical history**	☑									
**Physical examination, Vital signs, Performance status**	☑	☑	☑	☑	☑	☑	☑	☑	☑	☑
**Haematology**[a]	☑[5]	☑	☑	☑	☑	☑	☑	☑	☑	
**Coagulation**[b]	☑	☑								
**Biochemistry**[c]	☑[5]	☑	☑	☑	☑	☑	☑	☑	☑	
**Hormonology**[d]	☑[5]	☑	☑	☑	☑	☑	☑	☑	☑	
**Urinalysis (dipstick)**	☑[5]	☑	☑	☑	☑	☑	☑	☑	☑	
**Pregnancy test**[2]	☑[5]						☑		☑	☑
**ECG**	☑								☑	
**CT scan (Thoracic-Abdominal-Pelvic)**[3]	☑[4]						☑		☑	
**CA-125**	☑[5]						☑	☑	☑	☑
**Study treatment : Bevacizumab, 15mg/Kg IV**[9]		☑			☑	☑				
**Study treatment : Olaparib, 300mg bid po**[10]				☑						
**Study treatment : Durvalumab, 1.12g IV**[9]		☑			☑	☑				
**Adverse events/concomitant treatments**	☑	☑	☑	☑	☑	☑	☑	☑	☑	☑
**Translational Research: biopsy**	☑[6]							☑[7]		
**Translational Research: blood sample**	☑				☑	☑[8]				

1: Informed consent has to be signed any time before any study procedure.
2: Except for women without female genital tract.
3: MRI of the abdomen and pelvis and a chest X-ray can be performed instead of CT scan. The same imaging modality (CT or MRI) has to be used throughout the trial. If an unscheduled CT-scan or MRI of the abdomen and pelvis has been performed within six weeks of a scheduled image, it does not need to be repeated.
4: Imaging has to be done within 28 days of first dose
5:Test to be repeated within 7 days prior the treatment start.
6: Within 3 months before inclusion and performed after the last previous line chemotherapy administration
7: Tumor biopsy at relapse optional
8: blood sample at C3D1 and C6D1
9: Every 3 weeks (D21Cx = D1Cx+1)
10: Continuously
a:Hemoglobin, platelets, white blood cell, neutrophil, lymphcyts, absolute neutrophils count
b:Coagulation: aPTT, INR
c: Biochemistry: Albumin, Total bilirubin, Lactic dehydrogenase [LDH], Aspartate [AST] and Alanine transaminase [ALT], Alkaline Phosphatase [ALP], Sodium, Potassium, Calcium, Gamma Glutamyl transferase (GGT), Creatinin, Creatinin clearance, Glucose
d: Thyroid stimulating hormone (TSH), Free thyroxine (FT4)

4. SUBJECT SELECTION CRITERIA

The patient population should be selected without bias. Investigator(s) should keep a record, the patient screening log. Each patient should meet all of the inclusion criteria and none of the exclusion criteria for this study. Under no circumstances can there be exception to this rule. All patients must provide informed consent prior to any study specific procedures.

4.1. Inclusion criteria

Patients will be eligible for study participation if they respect all the following criteria:

I-1 Female Patient must be ≥18 years of age.

I-2 Signed informed consent and ability to comply with treatment and follow-up.

I-3 Patient with :

Ovarian cancer, primary peritoneal cancer and/or fallopian-tube cancer, histologically confirmed (based on local histopathological findings): high grade serous or high grade endometrioid or other high grade epithelial non mucinous ovarian tumor.

I-4 Patient who has completed at least one line of platinum-taxane chemotherapy, and presents with platinum resistant relapse (resistant disease defined by a tumor progression less than six months after the last dose of platinum) [Note: the patient may have received one or even more line of platinum based chemotherapy].

I-5 Patient who didn't receive any of the tested drugs, or previously received either bevacizumab or olaparib BUT NOT the combination of both drugs.

I-6 At least one measurable or evaluable lesion that can be accurately assessed at baseline by computed tomography (CT) (or magnetic resonance imaging [MRI] where CT is contraindicated) and is suitable for repeated assessment as per irRECIST. The baseline scan must be obtained within 28 days of first dose.

I-7 Availability of a pre-treatment tumor sample (archival FFPE block or fresh biopsy if feasible) lasting of less than 3 months before inclusion into the study and performed AFTER the last chemotherapy administration.

I-8 Patient not amenable to cytoreductive surgery at the time of relapse (surgery is not allowed during the protocole treatment).

I-9 Patient must have normal organ and bone marrow function:

a. Hemoglobin ≥ 9.0g/dL. (Transfusions is not allowed within 28 days before randomization)

b. Absolute neutrophil count (ANC) ≥ 1.5 x 10^9/L.

c. Platelet count ≥ 100 x 10^9/L. (Platelet transfusion or G-CSF administration is not allowed within 28 days before randomization)

d. Total bilirubin ≤ 1.5 x institutional upper limit of normal (ULN).

e. Aspartate aminotransferase / Serum Glutamic Oxaloacetic Transaminase (ASAT/SGOT)) and Alanine aminotransferase / Serum Glutamic Pyruvate Transaminase (ALAT/SGPT)) ≤ 2.5 x ULN, unless liver metastases are present in which case they must be ≤ 5 x ULN.

f. Creatinine clearance ≥ 60 mL/min by Cockcroft and Gault formula.

g. Patient not receiving anticoagulant medication who has an International Normalized Ratio (INR) ≤ 1.5 and an Activated ProThrombin Time (aPTT) ≤ 1.5 x ULN. The use of full-dose oral or parenteral anticoagulants is permitted as long as the INR or APTT is within therapeutic limits (according to site medical standard). If the patient is on oral anticoagulants, dose has to be stable for at least two weeks at the time of inclusion.

h. Urine dipstick for proteinuria < 2+. If urine dipstick is ≥ 2+, 24-hour urine must demonstrate < 1 g of protein in 24 hours.

i. Normal blood pressure or adequately treated and controlled hypertension (systolic BP ≤ 150 mmHg and/or diastolic BP ≤ 90 mmHg).

I-10 Expectancy of at least 12 weeks

I-11 Eastern Cooperative Oncology Group (ECOG) performance status 0-1.

I-12 Postmenopausal or evidence of non-childbearing status for women of childbearing potential prior to the first dose of study treatment (see protocol 2).

I-13 As this study will include patients in France, a subject will be eligible for inclusion in this study only if either affiliated to, or a beneficiary of, a social category.

Patients with platinum sensitive relapse

I-1 Female Patient must be ≥18 years of age.

I-2 Signed informed consent and ability to comply with treatment and follow-up.

I-3 Patient with :

Ovarian cancer, primary peritoneal cancer and/or fallopian-tube cancer, histologically confirmed (based on local histopathological findings): high grade serous or high grade endometrioid or other high grade epithelial non mucinous ovarian tumor.

I-4 Patient who is in platinum-sensitive relapse, whatever the line of chemotherapy given at relapse [Note: any chemotherapy previously administered must have contained a platinum compound]. The platinum sensitive relapse is defined by a tumor progression occurring more than six months after the last dose of platinum chemotherapy.

I-5 Patient who didn't receive any of the tested drugs, or previously received either bevacizumab or olaparib BUT NOT the combination of both drugs.

I-6 At least one measurable or evaluable lesion that can be accurately assessed at baseline by computed tomography (CT) (or magnetic resonance imaging [MRI] where CT is contraindicated) and is suitable for repeated assessment as per irRECIST. The baseline scan must be obtained within 28 days of first dose.

I-7 Availability of a pre-treatment tumor sample (archival FFPE block or fresh biopsy if feasible) lasting of less than 3 months before inclusion into the study and performed AFTER the last chemotherapy administration.

I-8 Patient not amenable to cytoreductive surgery at the time of relapse (surgery is not allowed during the protocole treatment).

I-9 Patient must have normal organ and bone marrow function:

a. Hemoglobin ≥ 9.0g/dL. (Transfusions is not allowed within 28 before randomization)

b. Absolute neutrophil count (ANC) ≥ 1.5 x 10^9/L.

c. Platelet count ≥ 100 x 10^9/L. (Platelet transfusion or G-CSF administration is not allowed within 28 days before randomization)

d. Total bilirubin ≤ 1.5 x institutional upper limit of normal (ULN).

e. Aspartate aminotransferase / Serum Glutamic Oxaloacetic Transaminase (ASAT/SGOT)) and Alanine aminotransferase / Serum Glutamic Pyruvate Transaminase (ALAT/SGPT)) ≤ 2.5 x ULN, unless liver metastases are present in which case they must be ≤ 5 x ULN.

f. Creatinine clearance ≥ 60 mL/min by Cockcroft and Gault formula.

g. Patient not receiving anticoagulant medication who has an International Normalized Ratio (INR) ≤ 1.5 and an Activated ProThrombin Time (aPTT) ≤ 1.5 x ULN. The use of full-dose oral or parenteral anticoagulants is permitted as long as the INR or APTT is within therapeutic limits (according to site medical standard). If the patient is on oral anticoagulants, dose has to be stable for at least two weeks at the time of incusion.

h. Urine dipstick for proteinuria < 2+. If urine dipstick is ≥ 2+, 24-hour urine must demonstrate < 1 g of protein in 24 hours.

i. Normal blood pressure or adequately treated and controlled hypertension (systolic BP ≤ 150 mmHg and/or diastolic BP ≤ 90 mmHg).

I-10 Expectancy of at least 12 weeks

I-11 Eastern Cooperative Oncology Group (ECOG) performance status 0-1.

I-12 Postmenopausal or evidence of non-childbearing status for women of childbearing potential prior to the first dose of study treatment.

I-13 As this study will include patients in France, a subject will be eligible for inclusion in this study only if either affiliated to, or a beneficiary of, a social category.

4.2. Exclusion criteria

Patient must not enter the study if any of the following exclusion criteria are fulfilled

E-1 Non-epithelial origin of the tumor (i.e. germ cell tumor).

E-2 Ovarian tumors of low malignant potential (e.g. borderline tumors), or mucinous carcinoma.

E-3 Carcinosarcoma (Mixed Mullerian Tumor)

E-4 Patient with synchronous primary endometrial cancer unless both of the following criteria are met:

- Stage < II,
- Less than 60 years old at the time of diagnosis of endometrial cancer with stage IA or IB grade 1 or 2, or stage IA grade III endometrial carcinoma,OR ≥ 60 years old at the time of diagnosis of endometrial cancer with stage IA grade 1 or 2 endometrioid adenocarcinoma.

Patient with serous or clear cell adenocarcinoma or carcinosarcoma of the endometrium is not eligible.

E-5 Other malignancy within the last 5 years except: adequately treated non-melanoma skin cancer, curatively treated in situ cancer of the cervix, ductal carcinoma in situ (DCIS). Patient with a history of localized malignancy diagnosed over 5 years ago may be eligible provided she completed her adjuvant systemic therapy and remains free of recurrent or metastatic disease.

Patient with history of primary triple negative breast cancer may be eligible provided she completed her definitive anticancer treatment more than 3 years ago and she remains breast cancer disease free prior to start of study treatment.

E-6 Patient with myelodysplastic syndrome/acute myeloid leukemia history.

E-7 Current or prior use of immunosuppressive medication within 14 days (use 28 days if combining durvalumab with a novel agent) before the first dose of durvalumab, with the exceptions of intranasal and inhaled corticosteroids or systemic corticosteroids at physiological doses, which are not to exceed 10 mg/day of prednisone, or an equivalent corticosteroid. The following are exceptions to this criterion:

- Intranasal, inhaled, topical steroids, or local steroid injections (e.g., intra articular injection)
- Systemic corticosteroids at physiologic doses not to exceed <<10 mg/day>> of prednisone or its equivalent
- Steroids as premedication for hypersensitivity reactions (e.g., CT scan premedication)

E-8 Any unresolved toxicity NCI CTCAE Grade ≥2 from previous anticancer therapy with the exception of alopecia, vitiligo, and the laboratory values defined in the inclusion criteria

- Patients with Grade ≥2 neuropathy will be evaluated on a case-by-case basis after consultation with the Study Physician.
- Patients with irreversible toxicity not reasonably expected to be exacerbated by treatment with durvalumab may be included only after consultation with the Study Physician.

E-9 Active or prior documented autoimmune or inflammatory disorders (including inflammatory bowel disease [e.g., colitis or Crohn's disease], diverticulitis [with the exception of diverticulosis], systemic lupus erythematosus, Sarcoidosis syndrome, or Wegener syndrome [granulomatosis with polyangiitis, Graves' disease, rheumatoid arthritis, hypophysitis, uveitis, etc]). The following are exceptions to this criterion:

- Patients with vitiligo or alopecia
- Patients with hypothyroidism (e.g., following Hashimoto syndrome) stable on hormone replacement
- Any chronic skin condition that does not require systemic therapy
- Patients without active disease in the last 5 years may be included but only after consultation with the study physician
- Uncontrolled intercurrent illness, including but not limited to, ongoing or active infection, symptomatic congestive heart failure, uncontrolled hypertension, unstable angina pectoris, cardiac arrhythmia, interstitial lung disease, serious chronic gastrointestinal conditions associated with diarrhea, or psychiatric illness/social situations that would limit compliance with study requirement, substantially increase risk of incurring AEs or compromise the ability of the patient to give written informed consent
- History of active primary immunodeficiency
- Active infection including tuberculosis (clinical evaluation that includes clinical history, physical examination and radiographic findings, and TB testing in line with local practice), hepatitis B (known positive HBV surface antigen (HBsAg) result), hepatitis C, or human immunodeficiency virus (positive HIV 1/2 antibodies). Patients with a past or resolved HBV infection (defined as the presence of hepatitis B core antibody [anti-HBc] and absence of HBsAg) are eligible. Patients positive for hepatitis C (HCV) antibody are eligible only if polymerase chain reaction is negative for HCV RNA.

E-10 Patient receiving radiotherapy within 6 weeks prior to study treatment.

E-11 Major surgery within 4 weeks of starting study treatment and patient must have recovered from any effects of any major surgery.

E-12 Previous allogenic bone marrow transplant.

E-13 Any previous treatment with Anti PD(L)-1 immunotherapy, including durvalumab

E-14 Any previous treatment with a PARP inhibitor in combination with an anti-VEGF (previous treatment with PARP inhibitor alone or anti-VEGF alone is allowed).

E-15 Past medical history of interstitial lung disease, drug-induced pneumonitis, radiation pneumonitis that required steroid treatment, or any evidence of clinically active interstitial lung disease

E-16 Administration of other simultaneous chemotherapy drugs, any other anticancer therapy or anti-neoplastic hormonal therapy, or simultaneous radiotherapy during the trial treatment period (hormonal replacement therapy is permitted as are steroidal antiemetics).

E-17 Current or recent (within 10 days prior to inclusion) chronic use of aspirin > 325 mg/day.

E-18 Concomitant use of known potent CYP3A4 inhibitors such as ketoconazole, itraconazole, ritonavir, indinavir, saquinavir, telithromycin, clarithromycin and nelfinavir. The required washout period prior to starting study treatment is 2 weeks.

E-19 Concomitant use of known strong (e.g. phenobarbital, enzalutamide, phenytoin, rifampicin, rifabutin, rifapentine, carbamazepine, nevirapine and St John's Wort) or moderate CYP3A inducers (e.g. bosentan, efavirenz, modafinil). The required washout period prior to starting study treatment is 5 weeks for enzalutamide or phenobarbital and 3 weeks for other agents. Patients should stop using herbal remedies 7 days prior to the first dose of study medication and for the duration of the trial.

E-20 Prior history of hypertensive crisis (CTCAE grade 4) or hypertensive encephalopathy.

E-21 Clinically significant (e.g. active) cardiovascular disease, Previous Cerebro-Vascular Accident (CVA), Transient Ischemic Attack (TIA) or Sub- Arachnoids Hemorrhage (SAH) within 6 months prior to inclusion.

E-22 History Clinically significant (e.g. active) cardiovascular disease, including:

- Myocardial infarction or unstable angina within ≤ 6 months of inclusion,
- New York Heart Association (NYHA) ≥ grade 2 congestive heart failure (CHF),
- Poorly controlled cardiac arrhythmia despite medication (patient with rate controlled atrial fibrillation are eligible), or any clinically significant abnormal finding on resting ECG,
- Peripheral vascular disease grade ≥ 3 (e.g. symptomatic and interfering with activities of daily living [ADL] requiring repair or revision) or evidence of hemorrhagic disorders within 6 months prior to treatment administration.

E-23 Evidence of bleeding diathesis or significant coagulopathy (in the absence of coagulation).

E-24 History or clinical suspicion of brain metastases or spinal cord compression. CT/MRI of the brain is mandatory (within 4 weeks prior to inclusion) in case of suspected brain metastases. Spinal MRI is mandatory (within 4 weeks prior to inclusion) in case of suspected spinal cord compression.

E-25 Significant traumatic injury during 4 weeks prior to inclusion.

E-26 Non-healing wound, active ulcer or bone fracture. Patient with granulating incisions healing by secondary intention with no evidence of facial dehiscence or infection is eligible but require 3 weekly wound examinations.

E-27 History of VEGF therapy related abdominal fistula or gastrointestinal perforation or active gastrointestinal bleeding within 6 months prior to the first study treatment.

E-28 Current, clinically relevant bowel obstruction, including sub-occlusive and occlusive disease.

E-29 Patient with evidence of abdominal free air not explained by paracentesis or recent surgical procedure.

E-30 Evidence of any other disease, metabolic dysfunction, physical examination finding or laboratory finding giving reasonable suspicion of a disease or condition that contraindicates the use of an investigational drug or puts the patient at high risk for treatment related complications.

E-31 Pregnant or lactating women.

E-32 Participation in another clinical study with an investigational product during her chemotherapy course immediately prior to inclusion.

E-33 Patient unable to swallow orally administered medication and patient with gastrointestinal disorders likely to interfere with absorption of the study medication.

E-34 Patient with a known hypersensitivity to olaparib, durvalumab or bevacizumab or any of the recipients of those products.

E-35 Immunocompromised patient, e.g., with known active hepatitis (i.e. Hepatitis B or C) due to risk of transmitting the infection through blood or other body fluids or patient who is known to be serologically positive for human immunodeficiency virus (HIV).

4.3. Child-bearing potential definition and birth control methods

The following restrictions must be applied while the patient is receiving bevacizumab, olaparib and durvalumab and for the specified times before and after: patients of childbearing potential and their partners, who are sexually active, must agree to the use of two highly effective forms of contraception throughout their participation in the study and for 6 months after last dose of treatment (study drug or chemotherapy) (see appendix 2).

4.4. Restriction for herbal medication

Herbal remedies (and any form of complementary medicine including and enhance food and treatment), should be avoided thought the study.

5. STUDY TREATMENT

5.1. Identity of investigational products

Olaparib/Durvalumab/proposed biosimilar bevacizumab (FKB238) will be the investigational medicinal product (IMP) of the study as defined in the Directive 2001/20 EC of the European Parliament*.

**According to Directive 2001/20 EC of the European Parliament an investigational medicinal product is a pharmaceutical form of an active substance or placebo being tested or used as a reference in a clinical trial, including products already with a marketing authorization but used or assembled (formulated or packaged) in a way different from the authorized form, or when used for an unauthorized indication or when used to gain further information about the authorized form.*

INVESTIGATIONAL PRODUCTS	DOSAGE FORM AND STRENGTH
Olaparib	Tablet – 150 mg and 100 mg
FKB238	Glass vial 25mg/mL - 100 mg (4 mL)
Durvalumab	Glass vial with 500 mg (nominal) of durvalumab (10.0 mL) solution for infusion after dilution.

Table 3 : IMP description.

Descriptive information for Olaparib, Durvalumab and FKB238 can be found in the current version of investigator brochures. Sponsor will supply Olaparib, Durvalumab and FKB238 to all sites.

5.2. Formulations, packaging and handling

5.2.1. Olaparib

For all centers, olaparib will be packed in high density polyethylene (HDPE) bottles with child-resistant closures. The study treatment will be dispensed to patients. Each dosing container will contain sufficient medication for at least each treatment period plus coverage. Multiple bottles of study treatment may be required for dispensing in order to make up the desired dose.
Patients will be administered the study treatment tablets orally at a dose of 300 mg twice daily.
Study treatment is available as a green film-coated tablet containing 150 mg or 100 mg of olaparib. Tablets are to be taken orally, twice a day. Doses of study treatment should be taken at the same times each day approximately 12 hours apart. All doses should be taken with approximately 240 mL of water. The study treatment tablets should be swallowed whole and not chewed, crushed, dissolved or divided. Study treatment tablets can be taken with a light meal/snack (e.g., two pieces of toast or a couple of biscuits).

5.2.2. FKB238

FKB238 is a clear to slightly opalescent, colourless to pale brown, sterile, pH 5.5 solution for IV infusion in a glass vial. Each vial contains 100 mg FKB238 in 4 mL at concentration of 25 mg/mL.
The necessary amount of FKB238 should be withdrawn and diluted in a total volume of 100 mL of 0.9% sodium chloride for injection. **DO NOT ADMINISTER OR MIX WITH DEXTROSE SOLUTION.**

FKB238 contains monosodium glutamate, sorbitol and polysorbate 80 as excipients. Although the

excipients proposed for the formulation of FKB238 DP are not the same as those used to formulate Avastin, they are in common use and have a well-documented safety profile.

5.2.3. Durvalumab

Durvalumab will be supplied as a 500 mg vial for solution for infusion. The solution contains 50 mg/mL Durvalumab, 26 mM histidine/histidine hydrochloride, 275 mM trehalose dihydrate, and 0.02% (weight/volume) polysorbate 80; it has a pH of 6.0. The solution will be diluted with 0.9% (w/v) saline or 5% (w/v) dextrose for iv infusion.

5.3. Labelling

Labels will be prepared in accordance with Good Manufacturing Practice (GMP) and local regulatory guidelines. The labels will fulfill GMP requirements for labeling. Label text will be translated into local language.

Specific dosing instructions will not be included on the label.

The patient emergency contact details will not be on the label but can be found in the informed consent. For emergency purposes the patient must be in possession of the emergency contact details at all times.

5.4. Storage

All study drugs should be kept in a secure place under appropriate storage conditions and must be used within the individually assigned expiry date specified on the label. Discard any unused portion of drug remaining.

5.4.1. Olaparib

The product should be stored in the pack provided and used according to the instructions on the label. For additional details, refer to the corresponding Olaparib investigator's brochure.

5.4.2. FKB238

Upon receipt of the FKB238, vials are to be refrigerated at 2-8°C (36-46°F) and should remain refrigerated until use. Vials should be protected from light. Do not freeze, do not shake. Vials are for single use only. Vials used for one patient may not be used for any other patient. For additional details, refer to the corresponding FKB238 investigator's brochure.

5.4.3. Durvalumab

Unopened vials of durvalumab liquid Drug Product must be stored at 2°C to 8°C (36°F to 46°F) and must not be frozen. Durvalumab should be kept in original packaging until use to prevent prolonged light exposure.

Total in-use storage time from needle puncture of Durvalumab vial to the start of administration should not exceed 4 hours at room temperature or 24 hours at 2°C to 8°C (36°F to 46°F). If the in-use storage time exceeds these limits, a new dose must be prepared from new vials. Durvalumab does not contain preservatives and any unused portion must be discarded. For additional details, refer to the corresponding biosimilar Durvalumab investigator's brochure.

5.5. Dosage and administration

For all randomized patients, the 3 drugs must be started concomitantly.

Olaparib will be administered first, followed by bevacizumab and then by Durvalumab.

Patient should continue to receive study treatment until progression disease as assessed by the investigator, or unacceptable toxicity, whichever is earlier, and as long as in the investigator's opinion he is benefiting from treatment and she does not meet any other discontinuation criteria as outlined in section 5.9, and in a limit of 24 months.

In case of stopping due to a single drug toxicity, the other drugs may be continued according to the investigator decision.

In case of treatment delay due to toxicity, the next course will be administred according to section 5.7.

A maximum delay of 4 weeks will be allowed.

Once patient has been discontinued from study treatment, other treatment options will be at the discretion of the investigator.

5.5.1. Olaparib

Patients will be administered the study treatment tablets orally at a dose of 300 mg twice daily.

Tablets are to be taken orally, twice every day. Doses of study treatment should be taken at the same times each day approximately 12 hours apart. All doses should be taken with approximately 240 mL of water. The study treatment tablets should be swallowed whole and not chewed, crushed, dissolved or divided. Study treatment tablets can be taken with a light meal/snack (e.g., two pieces of toast or a couple of biscuits).

If vomiting occurs shortly after the study treatment tablets are swallowed, the dose should only be replaced if all of the intact tablets can be seen and counted. Recommendations for handling nausea/vomiting are specified in section 5.7.

Should any patient enrolled on the study misses a scheduled dose for whatever reason (e.g., as a result of forgetting to take the tablets or vomiting), the patient will be allowed to take the scheduled dose up to a maximum of 2 hours after that scheduled dose time. If greater than 2 hours after the scheduled dose time, the missed dose is not to be taken and the patient should take the allotted dose at the next scheduled time.

5.5.2. FKB238

Patients will be administered the study treatment intravenous at the dose of 15mg/kg once daily every 3 weeks +/- 2 days.
The initial dose of FKB238 will be delivered over 90 (±15) minutes. If the first infusion is tolerated without infusion-associated adverse events (fever and/or chills), the second infusion may be delivered over 60 (±10) minutes. If the 60-minutes infusion is well tolerated, all subsequent infusions may be delivered over 30 (±10) minutes.

Treatment with FKB238 will start on Day 1 following confirmation of eligibility, after the olaparib administration, and will continue on a Q3W schedule.

If a patient experiences an infusion-associated adverse event, she may be premedicated for the next FKB238 infusion; however, the infusion time may not be decreased for the subsequent infusion. If the next infusion is well tolerated with premedication, the subsequent infusion time may then be decreased by 30 minutes as long as the patient continues to be premedicated. If a patient experiences a second episode of an infusion-associated adverse event with the 60-minute infusion, all subsequent doses should be given over 90 (±15) minutes. Similarly, if a patient experiences a second episode of an infusion-associated adverse event with the 30-minute infusion, all subsequent doses should be given over 60 (±10) minutes.

5.5.3. Durvalumab

Durvalumab (1.12 g) will be administered via IV infusion Q3W ±2 days. Treatment with Durvalumab will commence on Day 1 following confirmation of eligibility and will continue on a Q3W schedule. Durvalumab should be given after the FKB238 infusion and at least 1 hour after the patient has taken their olaparib morning dose. The final durvalumab (MEDI4736) concentration in the IV bag must be between 1 and 20 mg/mL, and the dose should be delivered through an IV administration set with a 0.2- or 0.22-µm in-line filter. Following preparation of Durvalumab, the entire contents of the IV bag should be administered as an IV infusion over approximately 60 minutes (±5 minutes). In the event that there are interruptions during infusion, the total allowed infusion time should not exceed 8 hours at room temperature. A 1-hour observation period is recommended after the first infusion of Durvalumab. If no clinically significant infusion reactions are observed during or after the first cycle, subsequent infusion observation periods can be at the Investigator's discretion (suggested 30 minutes after each v infusion). Durvalumab should not be co-administered with other drugs through the same infusion line.

5.6. Product accountability

In accordance with local regulatory requirements, the principal investigator, or designated site staff, is responsible for maintaining accountability records throughout the course of the study.

Study drug accountability, based on the number of received vials per product as unit, should be maintained by each site and should document the amount of IP received from sponsor, administrated to study subjects, and returned to sponsor or destroyed at site when applicable.

All dispensing and accountability records will be available for Sponsor review.

The pharmacist will dispense study treatment for each patient according to the Investigator's prescription and to the study protocol.

Any used, unused and expired study medication must be destroyed, upon authorization of the sponsor, according to local regulations and procedures. A copy of the destruction form must be filed in the Investigator and/or Pharmacy site files SF/PSF.

The medication provided for this trial is to be used only as indicated in this protocol and only for the patients entered in this study.

5.7. Management of toxicities and dose modification

In general, the toxicity profiles of FKB238, olaparib and durvalumab are nonoverlapping, therefore, management of toxicities will be per the individual guidelines of the respective agents.

Pneumonitis is considered to be the most important potential exception. The management guidelines for pneumonitis (see Section 5.7.4.2) integrate the guidance provided for these 3 agents.

5.7.1. General comments

The following general guidance should be followed for management of toxicities.

- Treat each of the toxicities with maximum supportive care (including holding the agent suspected of causing the toxicity if required).

- If the symptoms promptly resolve with supportive care, consideration should be given to continuing the same dose of the assigned IP along with appropriate continuing supportive care. If medically appropriate, dose modifications are permitted.

- All dose modifications should be documented with clear reasoning and documentation of the approach taken.

5.7.2. FKB238

Management of FKB238 toxicity during the experimental phase of the trial will be done according to FKB238 or Avastin SmPC. In brief, no dose reduction of FKB238 is foreseen for an individual patient. Skipped doses or termination of treatment will be based on the observed toxicities. If any weight change of more than 10% or more than 10 kg is observed, the treatment dosage should be modified accordingly. Other than in cases of significant weight change, no other dose modifications are allowed for FKB238. Missed doses will not be administered subsequently.

In cases of toxicity, please refer to the current version of the FKB238 Investigatior's brochure for guidance on how to manage toxicities that may lead to either interruption or permanently discontinuation such as:

- Hypertension.
- Proteinuria.
- Thrombosis/embolism.
- Hemorrhage.
- Congestive heart failure.
- Surgery.
- Wound healing complications in addition to any other grading FKB238 related toxicity (grade 3 or 4).

FKB238 should be temporarily withheld in the event of:

- Grade 4 febrile neutropenia.
- Grade 4 thrombocytopenia (regardless of the relationship to treatment), since these conditions are predisposing factors for an increased bleeding tendency.
- Grade 4 anemia.

5.7.2.1. Dose interruption of FKB238

If FKB238 cycles are temporarily interrupted for toxicity related to FKB238, olaparib and durvalumab will be continued, unless the toxicity of the FKB238 prevents the taking of olaparib or durvalumab or increase the risk of olaparib or durvalumab toxicity. In this case, both treatments will be interrupted until toxicity resolution (or CTCAE grade 1).

For patients who experience any infusion-associated symptoms believed to be due to FKB238 the infusion rate should be slowed to ≤ 50% or interrupted to allow resolution of symptoms. If the infusion rate is interrupted, it may be resumed at ≤ 50% of the rate prior to the reaction, at lease one hour after the patient's symptoms have adequately resolved and increased in 50% increments up to the full rate if well tolerated. Infusions may be restarted at the full rate during the next cycle.

5.7.2.2. Toxicities that required FKB238 permanently discontinuation

In addition, FKB238 treatment should be permanently discontinued in patients experiencing any of the following events:

- Reversible Posterior Leucoencephalopathy Syndrome (RPLS).
- Grade 3/4 hemorrhagic/bleeding events.
- Grade 3/4 left ventricular dysfunction (CHF).
- Grade 4 venous thromboembolism.
- Grade 4 hypertension (hypertensive crisis).
- Grade 4 non-gastrointestinal fistula.
- Grade 4 proteinuria (nephrotic syndrome).
- Grade 4 diarrhea or colitis
- Any grade of CNS bleeding.
- Any grade of arterial thromboembolism.
- Any grade of gastrointestinal perforation.
- Any grade of tracheo-esophageal fistula.
- Necrotizing fasciitis

5.7.3. Olaparib

Any toxicity observed during the course of the study that is believed to be due to olaparib could be managed by interruption of the dose of study treatment or dose reductions. Repeated dose interruptions are allowed, as required, for a maximum of 4 weeks on each occasion. If the interruption is any longer, the study team must be informed. Study treatment can be dose reduced to 250 mg bid as a first step and to 200 mg bid as a second step (Table 3). If the reduced dose of 200 mg bid is not tolerable, no further dose reduction is allowed and study treatment should be discontinued.
Once dose is reduced, escalation is not permitted.

General dose reduction guidance for olaparib is provided in Table 5, 6 and 7 below.

Initial dose	Following re-challenge post-interruption: dose reduction 1	Dose reduction 2
**300 mg twice daily**	250 mg twice daily	200 mg twice daily

Dose reduction level	Olaparib dose (mg) Twice a day		Tablets (dosage 150mg or 100mg)	
	Morning	evening	morning	evening
**Initial dose**	300mg	300mg	150mg + 150mg	150mg + 150mg
**First dose reduction**	250mg	250mg	150mg + 100mg	150mg + 100mg
**Second dose reduction**	200mg	200mg	100mg + 100mg	100mg + 100mg

Table 5 Dose reductions for study treatment

Specific guidance for the management of individual toxicities believed to be due to olaparib are provided in the following tables.

5.7.3.1. Management of hematological toxicity

Management of anemia

The management of anemia is presented in Table 6.

Hemoglobin level	Action to be taken
**Hb <10 but ≥8 g/dL (CTCAE grade 2)**	Give appropriate supportive treatment and investigate causality.<br>Investigator judgement to continue olaparib, continue olaparib with supportive treatment (eg, transfusion) or interrupt dose for a maximum of 4 weeks.<br>If repeat Hb <10 but ≥8 g/dL, dose interrupt (for max of 4 weeks) until Hb ≥10 g/dl and upon recovery dose reduction to 250 mg twice daily as a first step and to 200 mg twice daily as a second step may be considered.
**Hb <8 g/dL**	Give appropriate supportive treatment (eg, transfusion) and investigate causality.<br>Interrupt olaparib for a maximum of 4 weeks until improved to Hb ≥10 g/dL.<br>Upon recovery dose reduce to 250 mg twice daily as a first step and to 200 mg twice daily as a second step in the case of repeat Hb decrease.

CTCAE=Common Terminology Criteria for Adverse Event; Hb=hemoglobin

Table 6 Management of anemia

Common treatable causes of anemia (eg, iron, vitamin B12 or folate deficiencies and hypothyroidism) should be investigated and appropriately managed. In some cases, management of anemia may require blood transfusions. For cases were patients develop prolonged hematological toxicity (≥2 week interruption/delay in study treatment due to CTCAE grade 3 or higher anemia and/or development of blood transfusion dependence), refer to the section on Management of prolonged hematological toxicities while on study treatment for further information.

Management of neutropenia, leukopenia and thrombocytopenia

The management of neutropenia, leucopenia, and thrombocytopenia is presented in Table 7.

Toxicity	Study treatment dose adjustment

**CTCAE grade 1 to 2**	Investigator judgment to continue treatment or if dose interruption, this should be for a maximum of 4 weeks; appropriate supportive treatment and causality investigation.
**CTCAE grade 3 to 4**	Dose interruption until recovered to CTCAE grade 1 or better for a maximum of 4 weeks. If repeat CTCAE grade 3-4 occurrence, dose reduce olaparib to 250 mg bid as a first step and 200 mg bid as a second step.

bid=twice daily; CTCAE=Common Terminology Criteria for Adverse Events
Table 7 Management of neutropenia, leukopenia, and thrombocytopenia

Adverse event of neutropenia and leukopenia should be managed as deemed appropriate by the investigator with close follow-up and interruption of study drug if CTCAE grade 3 or worse neutropenia occurs.
Primary prophylaxis with granulocyte colony-stimulating factor (G-CSF) is not recommended, however, if a patient develops febrile neutropenia, study treatment should be interrupted and appropriate management including G-CSF should be given according to local hospital guidelines. Please note that G-CSF should not be used within at least 24 h of the last dose of study treatment unless absolutely necessary. GCSF should not be used in this case.

Platelet transfusions, if indicated, should be done according to local hospital guidelines.
Study treatment can be interrupted for CTCAE grade 1/2 neutropenia or thrombocytopenia as per investigator's judgment. In case of CTCAE grade 3/4 neutropenia or thrombocytopenia, study treatment should be interrupted for a maximum of 4 weeks. Study treatment can be restarted at the same dose if an AE of neutropenia or leukopenia has been recovered up to CTCAE grade 1 or better. Any subsequent interruptions will require study treatment dose reductions to 250 mg bid as a first step and to 200 mg bid as a second step.

Management of prolonged hematological toxicities while on study treatment
If a patient develops prolonged hematological toxicity such as:

- ≥2 week interruption/delay in study treatment due to CTCAE grade 3 or worse anemia and/or development of blood transfusion dependence
- ≥2 week interruption/delay in study treatment due to CTCAE grade 3 or worse neutropenia (ANC $<1\times10^9$/L)
- ≥2 week interruption/delay in study treatment due to CTCAE grade 3 or worse thrombocytopenia and/or development of platelet transfusion dependence (platelets $<50\times10^9$/L)

Check weekly differential blood counts including reticulocytes and peripheral blood smear. If any blood parameters remain clinically abnormal after 4 weeks of dose interruption, the patient should be referred to hematologist for further investigations. Bone marrow analysis and/or blood cytogenetic analysis should be considered at this stage according to standard hematological practice. Study treatment should be discontinued if blood counts do not recover to CTCAE grade 1 or better within 4 weeks of dose interruption.

Development of a confirmed MDS or other clonal blood disorder should be reported as an SAE and full reports must be provided by the investigator (see section 11.3). Olaparib treatment should be discontinued if patient's diagnosis of MDS and/or AML is confirmed.

5.7.3.2. Management of non-hematological toxicity

Repeat dose interruptions are allowed as required, for a maximum of 4 weeks on each occasion. If the interruption is any longer than this the study monitor must be informed.
Where toxicity reoccurs following re-challenge with study treatment, and where further dose interruptions are considered inadequate for management of toxicity, then the patient should be considered for dose reduction or must permanently discontinue study treatment.
Study treatment can be dose reduced to 250 mg bid as a first step and to 200 mg bid as a second step.
Treatment must be interrupted if any CTCAE grade 3 or 4 AE occurs, which the investigator considers to be related to administration of study treatment.

Management of new or worsening pulmonary symptoms

As pneumonitis is an AESI for durvalumab, please refer to Section 5.7.4.1.

Management of nausea and vomiting

Events of nausea and vomiting are known to be associated with olaparib treatment. In Study D0810C00019, nausea was reported in 71% of the olaparib-treated patients and 36% in the placebo-treated patients and vomiting was reported in 34% of the olaparib-treated patients and 14% in the placebo-treated patients. These events are generally mild to moderate (CTCAE grade 1 or 2) severity, intermittent and manageable on continued treatment. The first onset generally occurs in the first month of treatment for nausea and within the first 6 months of treatment for vomiting. For nausea, the incidence generally plateaus at around 9 months, and for vomiting at around 6 to 7 months.
No routine prophylactic anti-emetic treatment is required at the start of study treatment, however, patients should receive appropriate anti-emetic treatment at the first onset of nausea or vomiting and as required thereafter, in accordance with local treatment practice guidelines.
Alternatively, olaparib tablets can be taken with a light meal/snack (ie, 2 pieces of toast or a couple of biscuits).
As per international guidance on anti-emetic use in cancer patients (ESMO, National Comprehensive Cancer Network), generally a single-agent anti-emetic should be considered eg, dopamine receptor antagonist, antihistamines or dexamethasone.

**Nausea and vomiting**	
CTCAE grade > 1	First occurrence:<br>a) Introduce antiemetics (ex. Metoclopramide 10 mg may be given half hour before study treatment olaparib/placebo tablets).<br>*Nota Bene: nausea may be relieved in some patients when olaparib is taken with a snack*<br><br>b) If antiemetics are not enough sufficient to control nausea, interrupt temporarily study treatment (olaparib/placebo).<br>c) When nausea have subsided, restart study treatment at the same dose.<br>Subsequent:

	a) If the patient still complains of significant nausea despite antiemetics and previous study treatment interruption, re-interrupt study treatment.<br>b) When nausea have subsided, restart treatment at reduced dose:<br>- 250 mg twice a day as a first step, and<br>- 200 mg twice a day as a second step.

Interruptions for intercurrent non-toxicity related events

Study treatment dose interruption for conditions other than toxicity resolution should be kept as short as possible. If a patient cannot restart study treatment within 4 weeks for resolution of intercurrent conditions not related to disease progression or toxicity, the case should be discussed with the study coordinator and the DSMB.

All dose reductions and interruptions (including any missed doses), and the reasons for the reductions/interruptions are to be recorded in the eCRF.

No stoppage of study treatment is required for any needle biopsy procedure.

Study treatment should be discontinued for a minimum of 3 days before a patient undergoes radiation treatment. Study treatment should be restarted within 4 weeks as long as any bone marrow toxicity has recovered.

Because the AEs related to olaparib may include asthenia, fatigue and dizziness, patients should be advised to use caution while driving or using machinery if these symptoms occur.

Dose reductions for study treatment

Initial Dose	Following re-challenge post interruption: Dose reduction 1	Dose reduction 2
**300mg twice daily**	**250mg twice daily**	**200mg twice daily**

Renal Impairment

If subsequent to study entry and while still on study therapy, a patient's estimated CrCl falls below the threshold for study inclusion (≥51 ml/min), retesting should be performed promptly.

A dose reduction is recommended for patients who develop moderate renal impairment (calculated creatinine clearance by Cockcroft-Gault equation of between 31 and 50 ml/min) for any reason during the course of the study: the dose of olaparib should be reduced to 200mg BD.

Because the CrCl determination is only an estimate of renal function, in instances where the CrCl falls to between 31 and 50 mL/min, the investigator should use his or her discretion in determining whether a dose change or discontinuation of therapy is warranted.

Olaparib has not been studied in patients with severe renal impairment (creatinine clearance ≤ 30 ml/min) or end-stage renal disease; if patients develop severe impairment or end stage disease is it recommended that olaparib be discontinued.

5.7.4. Durvalumab

Following the first dose of IP, subsequent administration of durvalumab can be modified. All toxicities will be graded according to CTCAE V 5.0 Dose reductions are not permitted. Dose modifications of durvalumab may be required in the event of treatment-related toxicity. In case of doubt, the Investigator should consult with the Study Physician.

As with any antibody, allergic reactions to dose administration are possible. Appropriate drugs and medical equipment to treat acute anaphylactic reactions must be immediately available, and study personnel must be trained to recognize and treat anaphylaxis. The study site must have immediate access to emergency resuscitation teams and equipment in addition to the ability to admit patients to an intensive care unit if necessary.

5.7.4.1. Pneumonitis

If new or worsening pulmonary symptoms (eg, dyspnea or cough) or radiological abnormalities occur in the absence of a clear diagnosis, an interruption in study treatment dosing is recommended and further diagnostic workup should be performed to exclude pneumonitis. The differential diagnosis should include the possibility of both immune-related and non-immune related processes. Adverse events of pneumonitis are of interest for AstraZeneca as pneumonitis has been observed with use of anti-PD-1 mAbs (although not with anti-PD-L1 mAbs), and instances of pneumonitis have been reported in patients undergoing olaparib treatment.

Initial work-up should consider the inclusion of a clinical evaluation, high-resolution CT scan, ruling out infection, pulse oximetry, and other appropriate laboratory workup. Pulmonary consultation is highly recommended. Guidelines for the management of patients with irAEs including pneumonitis are provided in Table 8. Following investigation, if no evidence of abnormality is observed on CT imaging and symptoms resolve, then study treatment can be restarted, if deemed appropriate by the Investigator. If significant pulmonary abnormalities are identified, these need to be discussed with the Study Physician.

5.7.4.2. Infusion reactions

Acetaminophen and/or an antihistamine (eg, diphenhydramine) or equivalent medications per institutional standard may be administered prior to infusion at the discretion of the Investigator for primary prophylaxis against infusion-related reactions. In the event of grade ≤2 infusion-related reaction, the infusion rate of IP may be decreased by 50% or interrupted until resolution of the event (up to 4 hours) and re-initiated at 50% of the initial rate until completion of the infusion. In patients experiencing grade ≤2 infusion-related reaction, subsequent infusions may be administered at 50% of the initial rate. If a patient experiences an infusion-related reaction, acetaminophen and/or an antihistamine (eg, diphenhydramine) and/or corticosteroid or equivalent medications per institutional standard may be administered prior to subsequent infusions at the discretion of the investigator for secondary prophylaxis of infusion-related reactions. If the infusion-related reaction is grade 3 or higher in severity, treatment with IP will be discontinued.

AEs of infusion reactions (also termed infusion-related reactions) are of special interest to AstraZeneca and are defined, for the purpose of this protocol, as all AEs occurring from the start of IP infusion up to 48 hours after the infusion start time. For all infusion reactions, the eCRF should be completed as instructed in Section 14.3, and all SAEs should be reported (see section 11.3).

5.7.4.3. Hypersensitivity reactions

Hypersensitivity reactions as well as infusion-related reactions have been reported with anti-PD-L1 and anti-PD-1 therapy. As with the administration of any foreign protein and/or other biologic agents, reactions following the infusion of mAbs can be caused by various mechanisms, including acute

anaphylactic (immunoglobulin E-mediated) and anaphylactoid reactions against the mAbs and serum sickness. Acute allergic reactions may occur, may be severe, and may result in death. Acute allergic reactions may include hypotension, dyspnea, cyanosis, respiratory failure, urticaria, pruritus, angioedema, hypotonia, arthralgia, bronchospasm, wheeze, cough, dizziness, fatigue, headache, hypertension, myalgia, vomiting, and unresponsiveness. Guidelines for the management of patients with hypersensitivity (including anaphylactic reaction) and infusion-related reactions are provided in Table 8.

5.7.4.4. Hepatic function abnormalities (hepatotoxicity)

Hepatic function abnormality is defined as any increase in ALT or AST to greater than 3×ULN and concurrent increase in total bilirubin to be greater than 2×ULN. Concurrent findings are those that derive from a single blood draw or from separate blood draws taken within 8 days of each other. Follow-up investigations and inquiries will be initiated promptly by the investigational site to determine whether the findings are reproducible and/or whether there is objective evidence that clearly supports causation by a disease (eg, cholelithiasis and bile duct obstruction with distended gallbladder) or an agent other than the IP. Guidelines for management of patients with hepatic function abnormality are provided in Table 8.

5.7.4.5. Endocrine disorders

Immune-mediated endocrinopathies include hypophysitis, adrenal insufficiency, and hyper- and hypothyroidism. Guidelines for the management of patients with immune-mediated endocrine events are provided in table 8.

5.7.4.6. Pancreatic disorders

Immune-mediated pancreatitis includes autoimmune pancreatitis, and lipase and amylase elevation. Guidelines for the management of patients with immune-mediated pancreatic disorders are provided in Table 8.

5.7.4.7. Neurotoxicity

Immune-mediated nervous system events include encephalitis, peripheral motor and sensory neuropathies, Guillain-Barré, and myasthenia gravis. Guidelines for the management of patients with immune-mediated neurotoxic events are provided in Table 8.

5.7.4.8. Immune-related adverse events

An irAE is defined as a clinically significant adverse event of unknown etiology of any organ that is associated with drug exposure and is consistent with an immune-mediated mechanism.

Serologic, immunologic, and histologic (biopsy) data should be used to support an irAE diagnosis. Appropriate efforts should be made to rule out neoplastic, infectious, metabolic, toxin, or other etiologic causes of the irAE.

Based on the mechanism of action of durvalumab leading to T-cell activation and proliferation, there is a possibility of observing irAEs during the conduct of this study. Potential irAEs may be similar to those seen with the use of ipilimumab, BMS-936558 (anti PD-1 mAb), and BMS-936559 (anti-PD-L1 mAb) and may include immune-mediated enterocolitis, dermatitis, hepatitis (hepatotoxicity), pneumonitis, and endocrinopathies. These AEs are inflammatory in nature and can affect any organ. Patients should be monitored for signs and symptoms of irAEs. In the absence of an alternate etiology (eg, infection or PD), an immune-related etiology should be considered for signs or symptoms of

enterocolitis, dermatitis, pneumonitis, hepatitis, and endocrinopathy. In addition to the dose modification guidelines provided in Table 8, it is recommended that irAEs are managed according to the general treatment guidelines outlined for ipilimumab. These guidelines recommend the following:

1. Patients should be evaluated to identify any alternative etiology.
2. In the absence of a clear alternative etiology, all events of an inflammatory nature should be considered immune related.
3. Symptomatic and topical therapy should be considered for low-grade events.
4. Systemic corticosteroids should be considered for a persistent low-grade event or for a severe event.
5. More potent immunosuppressives should be considered for events not responding to systemic steroids (eg, infliximab for non-hepatic events or or mycophenolate).

If the investigator has any questions in regards to an AE being an irAE, the investigator should immediately contact the Study Physician.

Specific Immune-Mediated Reactions			
**Adverse Events**	**Severity Grade of the Event**	**Dose Modifications**	**Toxicity Management**
**Pneumonitis/Interstitial Lung Disease (ILD)**	**Any Grade** (Refer to NCI CTCAE applicable version in study protocol for defining the CTC grade/severity)	**General Guidance**	**For Any Grade:**<br>– Monitor patients for signs and symptoms of pneumonitis or ILD (new onset or worsening shortness of breath or cough). Patients should be evaluated with imaging and pulmonary function tests, including other diagnostic procedures as described below.<br>– Suspected pneumonitis should be confirmed with radiographic imaging and other infectious and disease-related aetiologies excluded, and managed as described below.<br>– Initial work-up may include clinical evaluation, monitoring of oxygenation via pulse oximetry (resting and exertion), laboratory work-up, and high- resolution CT scan.<br>– Consider Pulmonary and Infectious Diseases consults.
	**Grade 1**	No dose modifications required. However, consider holding study drug/study regimen dose as clinically appropriate and during diagnostic work-up for other etiologies.	**For Grade 1**<br>– Monitor and closely follow up in 2 to 4 days for clinical symptoms, pulse oximetry (resting and exertion), and laboratory work-up and then as clinically indicated.<br>–
	**Grade 2**	Hold study drug/study regimen dose until Grade 2 resolution to Grade ≤1.<br>• If toxicity worsens, then treat as Grade 3 or Grade 4.<br>• If toxicity improves to Grade ≤1, then the decision to reinitiate study drug/study regimen will be based upon treating physician's clinical judgment and after completion of steroid taper.	**For Grade 2**<br>– Monitor symptoms daily and consider hospitalization.<br>– Promptly start systemic steroids (e.g., prednisone 1 to 2 mg/kg/day PO or IV equivalent).<br>Reimage as clinically indicated, consider chest CT with contrast and repeat in 3-4 weeks<br>– If no improvement within 2 to 3 days, additional workup should be considered and prompt treatment with IV methylprednisolone 2 to 4 mg/kg/day started<br>– If no improvement within 2 to 3 days despite IV methylprednisolone at 2 to 4 mg/kg/day, promptly start immunosuppressive therapy such as TNF inhibitors (e.g., infliximab at 5 mg/kg IV once, may be repeated at 2 and 6 weeks after initial dose at the discretion of the treating provider). Caution: It is important to rule out sepsis and refer to infliximab label for general guidance before using infliximab.<br>– Consider, as necessary, discussing with study physician.
	**Grade 3 or 4**	**Permanently discontinue study drug/study regimen.**	**For Grade 3 or 4**

Specific Immune-Mediated Reactions			
**Adverse Events**	**Severity Grade of the Event**	**Dose Modifications**	**Toxicity Management**
			– Promptly initiate empiric IV methylprednisolone 1 to 4 mg/kg/day or equivalent.<br>– Obtain Pulmonary and Infectious diseases Consult; consider, discussing with study physician as needed.<br>– Hospitalize the patient.<br>– Supportive care (e.g., oxygen).<br>– If no improvement within 2 to 3 days, additional workup should be considered and prompt treatment with additional immunosuppressive therapy such as TNF inhibitors (e.g., infliximab at 5 mg/kg IV, may be repeated at 2 and 6 weeks after initial dose at the discretion of the treating provider . Caution: rule out sepsis and refer to infliximab label for general guidance before using infliximab.<br>–
**Diarrhea/Colitis**	**Any Grade**	**General Guidance**	**For Any Grade:**<br>– Monitor for symptoms that may be related to diarrhea/enterocolitis (abdominal pain, cramping, or changes in bowel habits such as increased frequency over baseline or blood in stool) or related to bowel perforation (such as sepsis, peritoneal signs, and ileus).<br>– WHEN SYMPTOMS OR EVALUATAION INDICATE A PERFORATION IS SUSPECTED, CONSULT A SURGEON EXPERIENCED IN ABDOMINAL SURGERY IMMEDIATELY WITHOUT DELAY.<br>– PERMANENTLY DISCONTINUE STUDY DRUG FOR ANY GRADE OF INTESTINAL PERFORATIONPatients should be thoroughly evaluated to rule out any alternative etiology (e.g., disease progression, other medications, or infections), including testing for clostridium difficile toxin, etc.<br>– Steroids should be considered in the absence of clear alternative etiology, even for low-grade events, in order to prevent potential progression to higher grade event including intestinal perforation.<br>– Use analgesics carefully; they can mask symptoms of perforation and peritonitis.
	**Grade 1**	No dose modifications.	**For Grade 1:**<br>– Monitor closely for worsening symptoms.<br>– Consider symptomatic treatment, including hydration, electrolyte replacement, dietary changes (e.g., American Dietetic Association colitis diet), loperamide, and

Specific Immune-Mediated Reactions			
**Adverse Events**	**Severity Grade of the Event**	**Dose Modifications**	**Toxicity Management**
			other supportive care measures. Use probiotics as per treating physician's clinical judgment.<br>– If symptoms persist, consider checking lactoferrin; if positive, treat as Grade 2 below. If negative and no infection, continue Grade 1 management.
	**Grade 2**	Hold study drug/study regimen until resolution to Grade ≤1<br>• If toxicity worsens, then treat as Grade 3 or Grade 4.<br>• If toxicity improves to Grade ≤1, then study drug/study regimen can be resumed after completion of steroid taper.	**For Grade 2:**<br>– Consider symptomatic treatment, including hydration, electrolyte replacement, dietary changes (e.g., American Dietetic Association colitis diet), and loperamide and/or budesonide.<br>– Promptly start prednisone 1 to 2 mg/kg/day PO or IV equivalent.<br>– If event is not responsive within 2 to 3 days or worsens despite prednisone at 1 to 2 mg/kg/day PO or IV equivalent, consult a GI specialist for consideration of further workup, such as imaging and/or colonoscopy, to confirm colitis and rule out perforation.<br>– If still no improvement within 2 to 3 days despite 1 to 2 mg/kg IV methylprednisolone, promptly start immunosuppressant agent such as infliximab at 5 mg/kg IV, may be repeated at 2 and 6 weeks after initial dose at the discretion of the treating provider. **Caution:** it is important to rule out bowel perforation and refer to infliximab label for general guidance before using infliximab.<br>– Consider, as necessary, discussing with study physician if no resolution to Grade ≤1 in 3 to 4 days.<br>–
	**Grade 3 or 4**	**Grade 3**<br><br>• For patient treated with PDL-1 inhibitors, hold study drug/study regimen until resolution to Grade ≤1; study drug/study regimen can be resumed after completion of steroid taper. Permanently discontinue study	**For Grade 3 or 4:**<br>– Promptly initiate empiric IV methylprednisolone 1 to 2 mg/kg/day or equivalent.<br>– Monitor stool frequency and volume and maintain hydration.<br>– Urgent GI consult and imaging and/or colonoscopy as appropriate.<br>– If still no improvement within 2 to 3 days, promptly addfurther immunosuppressants (e.g., infliximab at 5 mg/kg IV, may be repeated at 2 and 6 weeks after initial dose at the discretion of the treating provider). **Caution**: Ensure GI consult to rule out bowel perforation and refer to infliximab label for general guidance before using infliximab.

Specific Immune-Mediated Reactions			
**Adverse Events**	**Severity Grade of the Event**	**Dose Modifications**	**Toxicity Management**
		drug/study regimen for Grade 3 if toxicity does not improve to Grade ≤1 within 14 days.<br>• Permanently discontinue study drug for 1) Grade 3 colitis in patients treated with CTLA-4 inhibitors or 2) Any grade of intestinal perforation in any patient treated with ICI.<br>**Grade 4**<br>**Permanently discontinue study drug/study regimen.**	– **If perforation is suspected, consult a surgeon experienced in abdominal surgery immediately without any delay.**<br>–
**Hepatitis (elevated LFTs)**<br>Infliximab should not be used for management of immune-related hepatitis.<br>**PLEASE SEE the area immediately below this section to find guidance for management of "Hepatitis (elevated LFTS)" in HCC patients**	**Any Grade**<br>(Refer to NCI CTCAE applicable version in study protocol for defining the CTC grade/severity)	**General Guidance**	**For Any Grade:**<br>– Monitor and evaluate liver function test: AST, ALT, ALP, and TB.<br>– Evaluate for alternative etiologies (e.g., viral hepatitis, disease progression, concomitant medications).
	**Grade 1**	• No dose modifications.<br>• If it worsens, then treat as Grade 2 event.	**For Grade 1:**<br>– Continue LFT monitoring per protocol.
	**Grade 2**	• Hold study drug/study regimen dose until Grade 2 resolution to Grade ≤1.<br>• If toxicity worsens, then treat as Grade 3 or Grade 4.<br>• If toxicity improves to Grade ≤1 or baseline, resume study drug/study	**For Grade 2:**<br>– Regular and frequent checking of LFTs (e.g., every 1 to 2 days) until LFTs elevations improve or resolve.<br>– If no resolution to Grade ≤1 in 1 to 2 days, consider, discussing with study physician as needed.<br>– If event is persistent (>2 to 3 days) or worsens, promptly start prednisone 1 to 2 mg/kg/day PO or IV equivalent.

Specific Immune-Mediated Reactions			
**Adverse Events**	**Severity Grade of the Event**	**Dose Modifications**	**Toxicity Management**
		regimen after completion of steroid taper.<br>• Permanently discontinue study drug/study regimen for any case meeting Hy's law criteria (AST and/or ALT >3 × ULN + bilirubin >2 × ULN without initial findings of cholestasis (i.e., elevated alkaline P04) and in the absence of an alternative cause[b].	–
	**Grade 3 or 4**<br><br>(Grade 4: AST or ALT >20×ULN and/or TB >10×ULN)	**For Grade 3:**<br>For elevations in transaminases ≤8 × ULN, or elevations in bilirubin ≤5 × ULN:<br>• Hold study drug/study regimen dose until resolution to Grade ≤1 or baseline<br>• Resume study drug/study regimen if elevations downgrade to Grade ≤1 or baseline within 14 days and after completion of steroid taper.<br>• Permanently discontinue study drug/study regimen if the elevations do not downgrade to Grade ≤1 or baseline within 14 days<br>For elevations in transaminases >8 × ULN or elevations in bilirubin >5 × ULN, discontinue study drug/study regimen.<br><br>**For Grade 4:**<br>**Permanently discontinue study drug/study regimen.**	**For Grade 3 or 4:**<br>– Promptly initiate empiric IV methylprednisolone at 1 to 4 mg/kg/day or equivalent.<br>– If still no improvement within 2 to 3 days despite 1 to 2 mg/kg/day methylprednisolone IV or equivalent, promptly start treatment with an immunosuppressant therapy (i.e., mycophenolate mofetil 0.5 – 1 g every 12 hours then taper in consultation with hepatology consult). Discuss with study physician if mycophenolate is not available. **Infliximab should NOT be used.**<br>– Perform hepatology consult, abdominal workup, and imaging as appropriate.

Specific Immune-Mediated Reactions			
**Adverse Events**	**Severity Grade of the Event**	**Dose Modifications**	**Toxicity Management**
**Hepatitis**<br>**(elevated LFTs)**<br>Infliximab should not be used for management of immune-related hepatitis.<br><br>**THIS area is guidance *only* for management of "Hepatitis (elevated LFTs)" in HCC patients**	**Any Grade(Any Elevations of AST,**<br>**ALT, or TB as**<br>**Described Below)**	**General Guidance**	**For Any Grade (any Elevations Described):**<br>– Monitor and evaluate liver function test: AST, ALT, ALP, and TB.<br>– Evaluate for alternative etiologies (e.g., viral hepatitis, disease progression, concomitant medications, worsening of liver cirrhosis [e.g., portal vein thrombosis]).<br>– For HBV+ patients: evaluate quantitative HBV viral load, quantitative HBsAg, or HBeAg<br>– For HCV+ patients: evaluate quantitative HCV viral load<br>– Consider consulting Hepatology or Infectious diseases specialist regarding changing or starting antiviral HBV medications if HBV viral load is >2000 IU/ml<br>– Consider consulting Hepatology or Infectious diseases specialist regarding changing or starting antiviral HCV medications if HCV viral load has increased by ≥2-fold<br>– For HCV+ with HBcAb+: Evaluate for both HBV and HCV as above
See instructions at bottom of this section if transaminase rise is not isolated but (at any time) occurs in setting of either **increasing bilirubin or signs of DILI/liver decompensation**	**Grade 1**Isolated AST or ALT >ULN and ≤5.0×ULN, whether normal or elevated at baseline	• No dose modifications.<br>• If ALT/AST elevations represents significant worsening based on investigator assessment, then treat as Grade 2 event.<br>For all grades, see instructions at bottom of shaded area if transaminase rise is not isolated but (at any time) occurs in setting of either **increasing bilirubin or signs of DILI/liver decompensation**	

Specific Immune-Mediated Reactions			
**Adverse Events**	**Severity Grade of the Event**	**Dose Modifications**	**Toxicity Management**
	**Grade 2**<br>(Isolated AST or ALT >5.0×ULN and ≤8.0×ULN, if normal at baseline)<br><br>(Isolated AST or ALT >2.0×baseline and ≤12.5×ULN, if elevated >ULN at baseline)	• Hold study drug/study regimen dose until Grade 2 resolution to Grade ≤1 or baseline.<br>• If toxicity worsens, then treat as Grade 3 or Grade 4.<br>If toxicity improves to Grade ≤1 or baseline, resume study drug/study regimen after completion of steroid taper.<br><br>Permanently discontinue study drug/study regimen for any case meeting Hy's law criteria, in the absence of any alternative cause[b].	**For Grade 2:**<br>– Regular and frequent checking of LFTs (e.g., every 1 to 3 days) until elevations of these are improving or resolved.<br>– Recommend consult hepatologist; consider abdominal ultrasound, including Doppler assessment of liver perfusion.<br>– Consider, as necessary, discussing with study physician.<br>– If event is persistent (>2 to 3 days) or worsens, and investigator suspects toxicity to be imAE, start prednisone 1 to 2 mg/kg/day PO or IV equivalent.<br>– If still no improvement within 2 to 3 days despite 1 to 2 mg/kg/day of prednisone PO or IV equivalent, consider additional workup If still no improvement within 2 to 3 days despite 1 to 2 mg/kg/day of IV methylprednisolone, consider additional abdominal workup (including liver biopsy) and imaging (i.e., liver ultrasound), and consider starting immunosuppressants (i.e., mycophenolate mofetil 0.5 – 1 g every 12 hours then taper in consultation with hepatology consult).[a] Discuss with study physician if mycophenolate mofetil is not available. **Infliximab should NOT be used.**
	**Grade 3**<br>(Isolated AST or ALT >8.0×ULN and ≤20.0×ULN, if normal at baseline)<br><br>(Isolated AST or ALT >12.5×ULN and ≤20.0×ULN, if elevated >ULN at baseline)	• Hold study drug/study regimen dose until resolution to Grade ≤1 or baseline<br>• Resume study drug/study regimen if elevations downgrade to Grade ≤1 or baseline within 14 days and after completion of steroid taper.<br>• Permanently discontinue study drug/study regimen if the elevations do not downgrade to Grade ≤1 or baseline within 14 days	**For Grade 3:**<br>– Regular and frequent checking of LFTs (e.g., every 1-2 days) until elevations of these are improving or resolved.<br>– Consult hepatologist (unless investigator is hepatologist); obtain abdominal ultrasound, including Doppler assessment of liver perfusion; and consider liver biopsy.<br>– Considerdiscussing with study physician as needed.<br>– If investigator suspects toxicity to be immune-mediated, promptly initiate empiric IV methylprednisolone at 1 to 2 mg/kg/day or equivalent.<br>– If no improvement within 2 to 3 days despite 1 to 4 mg/kg/day methylprednisolone IV or equivalent, obtain liver biopsy (if it has not been done already) and promptly start treatment with an immunosuppressive therapy (mycophenolate mofetil 0.5 – 1 g every 12 hours then taper in consultation with hepatology consult). Discuss with study physician if mycophenolate is not available. **Infliximab should NOT be used.**<br>–

Specific Immune-Mediated Reactions			
**Adverse Events**	**Severity Grade of the Event**	**Dose Modifications**	**Toxicity Management**
	**Grade 4**<br>(Isolated AST or ALT >20×ULN, whether normal or elevated at baseline)	**Permanently discontinue study drug/study regimen.**	**For Grade 4:**<br>**Same as above**<br>**(except would recommend obtaining liver biopsy early)**
**If transaminase rise is not isolated but (at any time) occurs in setting of either increasing total/direct bilirubin (≥1.5×ULN, if normal at baseline; or 2×baseline, if >ULN at baseline) or signs of DILI/liver decompensation (e.g., fever, elevated INR):**<br>- **Manage dosing for Grade 1 transaminase rise as instructed for Grade 2 transaminase rise**<br>- **Manage dosing for Grade 2 transaminase rise as instructed for Grade 3 transaminase rise**<br>- **Grade 3-4: Permanently discontinue study drug/study regimen**			
**Nephritis or renal dysfunction**<br>(elevated serum creatinine)	**Any Grade**<br>**(Refer to NCI CTCAE applicable version in study protocol for defining the CTC grade/severity)**	**General Guidance**	**For Any Grade:**<br>– Consult nephrologist.<br>– Monitor for signs and symptoms that may be related to changes in renal function (e.g., routine urinalysis, elevated serum BUN and creatinine, decreased creatinine clearance, electrolyte imbalance, decrease in urine output, or proteinuria).<br>– Patients should be thoroughly evaluated to rule out any alternative etiology (e.g., disease progression. infections. Recent IV contrast, medications, fluid status).<br>– Consider using steroidsin the absence of clear alternative etiology even for low-grade events (Grade 2), in order to prevent potential progression to higher grade event.
	**Grade 1**	No dose modifications.	**For Grade 1:**<br>– Monitor serum creatinine weekly and any accompanying symptoms.

Specific Immune-Mediated Reactions			
**Adverse Events**	**Severity Grade of the Event**	**Dose Modifications**	**Toxicity Management**
			• If creatinine returns to baseline, resume its regular monitoring per study protocol.<br>• If creatinine worsens, depending on the severity, treat as Grade 2, 3, or 4.<br>– Consider symptomatic treatment, including hydration, electrolyte replacement, and diuretics.
	**Grade 2**	Hold study drug/study regimen until resolution to Grade ≤1 or baseline.<br>• If toxicity worsens, then treat as Grade 3 or 4.<br>• If toxicity improves to Grade ≤1 or baseline, then resume study drug/study regimen after completion of steroid taper.	**For Grade 2:**<br>– Consider symptomatic treatment, including hydration, electrolyte replacement, and diuretics.<br>– Carefully monitor serum creatinine every 2 to 3 days and as clinically warranted.<br>– Consult nephrologist and consider renal biopsy if clinically indicated.<br>– If event is persistent beyond 3 to 5 days or worsens, promptly start prednisone 1 to 2 mg/kg/day PO or IV equivalent.<br>– If event is not responsive within 3 to 5 days or worsens despite prednisone at 1 to 2 mg/kg/day PO or IV equivalent, consider additional workup<br>– When event returns to baseline, resume study drug/study regimen and routine serum creatinine monitoring per study protocol.
	**Grade 3 or 4**	**Permanently discontinue study drug/study regimen.**	**For Grade 3 or 4:**<br>– Carefully monitor serum creatinine<br>– Consult nephrologist and consider renal biopsy if clinically indicated.<br>– Promptly start prednisone 1 to 2 mg/kg/day PO or IV equivalent.<br>– If event is not responsive within 3 to 5 days or worsens despite prednisone at 1 to 2 mg/kg/day PO or IV equivalent, consider additional workup and prompt treatment with an immunosuppressant in consultation with a nephrologist.
**Rash or Dermatitis**<br>(including Pemphigoid)	**Any Grade**	**General Guidance**	**For Any Grade:**<br>– Monitor for signs and symptoms of dermatitis (rash and pruritus).

Specific Immune-Mediated Reactions			
**Adverse Events**	**Severity Grade of the Event**	**Dose Modifications**	**Toxicity Management**
	(Refer to NCI CTCAE applicable version in study protocol for definition of severity/grade depending on type of skin rash)		– HOLD STUDY DRUG IF STEVENS-JOHNSON SYNDROME (SJS), TOXIC EPIDERMAL NECROLYSIS (TEN), OR OTHER SEVERE CUTANEOUS ADVERSE REACTION (SCAR) IS SUSPECTED.<br>– PERMANENTLY DISCONTINUE STUDY DRUG IF SJS, TEN, OR SCAR IS CONFIRMED.<br>–
	**Grade 1**	No dose modifications.	**For Grade 1:**<br>– Consider symptomatic treatment, including oral antipruritics (e.g., diphenhydramine or hydroxyzine) and topical therapy (e.g., emolient lotion or institutional standard).
	**Grade 2**	For persistent (>1 week) Grade 2 events, hold scheduled study drug/study regimen until resolution to Grade ≤1 or baseline.<br>• If toxicity worsens, then treat as Grade 3.<br>• If toxicity improves to Grade ≤1 or baseline, then resume drug/study regimen after completion of steroid taper.	**For Grade 2:**<br>– Obtain dermatology consult.<br>– Consider symptomatic treatment, including oral antipruritics (e.g., diphenhydramine or hydroxyzine) and topical therapy.<br>– Consider moderate-strength topical steroid.<br>– If no improvement of rash/skin lesions occurs within 3 days or is worsening despite symptomatic treatment and/or use of moderate strength topical steroid, consider, discussing with study physician, as needed, and promptly start systemic steroids such as prednisone 1 to 2 mg/kg/day PO or IV equivalent.<br>– Consider skin biopsy if the event persist for >1 week or recurs.
	**Grade 3 or 4**	**For Grade 3:**<br>Hold study drug/study regimen until resolution to Grade ≤1 or baseline.<br>• If toxicity improves to Grade ≤1 or baseline, then resume drug/study regimen after completion of steroid taper.<br>• If toxicity worsens, then treat as	**For Grade 3 or 4:**<br>– Consult dermatology.<br>– Promptly initiate empiric IV methylprednisolone 1 to 2 mg/kg/day or equivalent.<br>– Consider hospitalization.<br>– Monitor extent of rash [Rule of Nines].<br>– Consider skin biopsy (preferably more than 1) as clinically feasible.<br>– Consider, as necessary, discussing with study physician.

Specific Immune-Mediated Reactions			
**Adverse Events**	**Severity Grade of the Event**	**Dose Modifications**	**Toxicity Management**
		Grade 4.<br>**For Grade 4:**<br>**Permanently discontinue study drug/study regimen.**	
**Endocrinopathy**<br>(e.g., hyperthyroidism, hypothyroidism, Type 1 diabetes mellitus, hypophysitis, hypopituitarism, and adrenal insufficiency;)	**Any Grade**<br><br>(Depending on the type of endocrinopathy, refer to NCI CTCAE applicable version in study protocol for defining the CTC grade/severity)	**General Guidance**	**For Any Grade:**<br>– Consider consulting an endocrinologist for endocrine events.<br>– Consider discussing with study physician as needed.<br>– Monitor patients for signs and symptoms of endocrinopathies. Non-specific symptoms include headache, fatigue, behavior changes, mental status changes, photophobia, visual field cuts, vertigo, abdominal pain, unusual bowel habits, polydipsia, polyuria, hypotension, and weakness.<br>– Patients should be thoroughly evaluated to rule out any alternative etiology (e.g., disease progression including brain metastases, or infections).<br>– Depending on the suspected endocrinopathy, monitor and evaluate thyroid function tests: TSH, free T3 and free T4 and other relevant endocrine and related labs (e.g., blood glucose and ketone levels, HgA1c).<br>– If a patient experiences an AE that is thought to be possibly of autoimmune nature (e.g., thyroiditis, pancreatitis, hypophysitis, or diabetes insipidus), the investigator should send a blood sample for appropriate autoimmune antibody testing.<br>– Investigators should ask subjects with endocrinopathies who may require prolonged or continued hormonal replacement, to consult their primary care physicians or endocrinologists about further monitoring and treatment after completion of the study.
	**Grade 1**	No dose modifications.	**For Grade 1**<br>– Monitor patient with appropriate endocrine function tests.<br>– For suspected hypophysitis/hypopituitarism, consider consulting of an endocrinologist to guide assessment of early-morning ACTH, cortisol, TSH and free T4; also consider gonadotropins, sex hormones, and prolactin

Specific Immune-Mediated Reactions			
**Adverse Events**	**Severity Grade of the Event**	**Dose Modifications**	**Toxicity Management**
			levels, as well as cosyntropin stimulation test (though it may not be useful in diagnosing early secondary adrenal insufficiency).<br>– If TSH < 0.5 × LLN, or TSH >2 × ULN, or consistently out of range in 2 subsequent measurements, include free T4 at subsequent cycles as clinically indicated and consider consultation of an endocrinologist.
	**Grade 2, 3 or 4**	For Grade 2-4 endocrinopathies other than hypothyroidism and Type 1 diabetes mellitus, consider holding study drug/study regimen dose until Acute symptoms resolve.<br>Study drug/study regimen can be resumed once patient stabilizes and after completion of steroid taper.<br>Patients with endocrinopathies who may require prolonged or continued steroid replacement (e.g., adrenal insufficiency) can be retreated with study drug/study regimen<br>1. patient is clinically stable as per investigator or treating physician's clinical judgement.<br>2. If toxicity worsens, then treat based on severity	**For Grade 2, 3 or 4**<br>– Consult endocrinologist to guide evaluation of endocrine function and, as indicated by suspected endocrinopathy and as clinically indicated, consider pituitary scan.<br>– For all patients with abnormal endocrine work up, except those with isolated hypothyroidism or type 1 DM, and as guided by an endocrinologist, consider short-term corticosteroids (e.g., 1 to 2 mg/kg/day methylprednisolone or IV equivalent) and prompt initiation of treatment with relevant hormone replacement (e.g., hydrocortisone, sex hormones).<br>– Isolated hypothyroidism may be treated with replacement therapy, without study drug/study regimen interruption, and without corticosteroids.<br>– Isolated type 1 diabetes mellitus (DM) may be treated with appropriate diabetic therapy, and without corticosteroids. Only hold study drug/study regimen in setting of hyperglycemia when diagnostic workup is positive for diabetic ketoacidosis.<br>– For patients with normal endocrine workup (laboratory assessment or MRI scans), repeat laboratory assessments/MRI as clinically indicated.
		1.	–
**Amylase/Lipase increased**	**Any Grade**<br>(Refer to NCI CTCAE applicable version in study protocol for defining the CTC grade/severity)	**General Guidance**	**For Any Grade:**<br>– For modest asymptomatic elevations in serum amylase and lipase, corticosteroid treatment is not indicated as long as there are no other signs or symptoms of pancreatic inflammation.<br>– Assess for signs/symptoms of pancreatitis<br>– Consider appropriate diagnostic testing (e.g., abdominal CT with contrast, MRCP if clinical suspicion of pancreatitis and no radiologic evidence on CT)
	**Grade 1**	No dose modifications.	

Specific Immune-Mediated Reactions			
**Adverse Events**	**Severity Grade of the Event**	**Dose Modifications**	**Toxicity Management**
	**Grade 2, 3 or 4**	**For Grade 2, 3, or 4:**<br>In consultation with relevant pancreatic specialist consider continuing study drug/study regimen if no clinical/radiologic evidence of pancreatitis ± improvement in amylase/lipase.	– If isolated elevation of enzymes without evidence of pancreatitis, continue immunotherapy. Consider other causes of elevated amylase/lipase<br>– If evidence of pancreatitis, manage according to pancreatitis recommendations
**Acute Pancreatitis**	**Any Grade**<br>(Refer to NCI CTCAE applicable version in study protocol for defining the CTC grade/severity)	**General Guidance**	**For Any Grade:**<br>Consider Gastroenterology referral
	**Grade 1**	No dose modifications.	**For Grade 1:**<br>- IV hydration<br>- Manage as per amylase/lipase increased (asymptomatic)
	**Grade 2, 3 or 4**	**For Grade 2 :**<br>Hold study drug/study regimen dose until resolution to Grade ≤1.<br>**For Grade 3 or 4:**<br>Permanently discontinue study drug/study regimen.	**For Grade 2, 3, or 4:**<br>– Promptly start systemic steroids prednisone 1 to 2 mg/kg/day PO or IV equivalent.<br>– IV hydration
**Neurotoxicity**<br>(to include but not be limited to non-infectious meningitis, non-infectious encephalitis and autonomic neuropathy, excluding Myasthenia Gravis and Guillain-Barre)	**Any Grade**<br>(Depending on the type of neurotoxicity, refer to CTCAE applicable version in study protocol for defining the CTC grade/severity)	**General Guidance**	**For Any Grade:**<br>– Patients should be evaluated to rule out any alternative etiology (e.g., disease progression, infections, metabolic syndromes, or medications).<br>– Monitor patient for general symptoms (headache, nausea, vertigo, behavior change, or weakness).<br>– Consider appropriate diagnostic testing (e.g., electromyogram and nerve conduction investigations).<br>– Perform symptomatic treatment with neurological consult as appropriate.FOR TRANSVERSE MYELITIS, PERMANENTLY DISCONTINUE FOR ANY GRADE.

Specific Immune-Mediated Reactions			
**Adverse Events**	**Severity Grade of the Event**	**Dose Modifications**	**Toxicity Management**
	**Grade 1**	No dose modifications.	**For Grade 1:**<br>– See "Any Grade" recommendations above.
	**Grade 2**	For acute motor neuropathies or neurotoxicity, hold study drug/study regimen dose until resolution to Grade ≤1.<br>For sensory neuropathy/neuropathic pain, consider holding study drug/study regimen dose until resolution to Grade ≤1.<br>Permanently discontinue study drug/study regimen if Grade 2 imAE does not resolve to Grade ≤1 within 30 days.<br>If toxicity worsens, then treat as Grade 3 or 4.	**For Grade 2:**<br>– Consider, as necessary, discussing with the study physician.<br>– Obtain neurology consult.<br>– Sensory neuropathy/neuropathic pain may be managed by appropriate medications (e.g., gabapentin or duloxetine).<br>– Promptly start systemic steroids prednisone 1 to 2 mg/kg/day PO or IV equivalent.<br>If no improvement within 2 to 3 days despite 1 to 2 mg/kg/day prednisone PO or IV equivalent, consider additional workup and promptly treat with an additional immunosuppressive therapy (e.g., IV IG or other immunosuppressant depending on the specific imAE).
	**Grade 3 or 4**	**For Grade 3 or 4:**<br>Permanently discontinue study drug/study regimen.	**For Grade 3 or 4:**<br>– Consider, as necessary, discussing with study physician.<br>– Obtain neurology consult.<br>– Consider hospitalization.<br>– Promptly initiate empiric IV methylprednisolone 1 to 2 mg/kg/day or equivalent.<br>– If no improvement within 2 to 3 days despite IV corticosteroids, consider additional workup and promptly treat with additional immunosuppressants (e.g., IV IG or other immunosuppressant depending on the specific imAE).<br>– Once stable, gradually taper steroids over ≥28 days.
**Peripheral neuromotor syndromes**<br>(such as Guillain-Barre and myasthenia gravis)	**Any Grade**<br>(Refer to NCI CTCAE applicable version in study protocol for defining the CTC	**General Guidance**	**For Any Grade:**<br>– The prompt diagnosis of immune-mediated peripheral neuromotor syndromes is important, since certain patients may unpredictably experience acute decompensations that can result in substantial morbidity or in the worst case, death. Special care should be taken for certain sentinel

Specific Immune-Mediated Reactions			
**Adverse Events**	**Severity Grade of the Event**	**Dose Modifications**	**Toxicity Management**
	grade/severity)		symptoms that may predict a more severe outcome, such as prominent dysphagia, rapidly progressive weakness, and signs of respiratory insufficiency or autonomic instability.<br>– Patients should be evaluated to rule out any alternative etiology (e.g., disease progression, infections, metabolic syndromes or medications). It should be noted that the diagnosis of immune-mediated peripheral neuromotor syndromes can be particularly challenging in patients with underlying cancer, due to the multiple potential confounding effects of cancer (and its treatments) throughout the neuraxis. Given the importance of prompt and accurate diagnosis, it is essential to have a low threshold to obtain a neurological consult.<br>– Neurophysiologic diagnostic testing (e.g., electromyogram and nerve conduction investigations, and “repetitive stimulation” if myasthenia is suspected) are routinely indicated upon suspicion of such conditions and may be best facilitated by means of a neurology consultation.<br>– It is important to consider that the use of steroids as the primary treatment of Guillain-Barre is not typically considered effective. Patients requiring treatment should be started with IV IG and followed by plasmapheresis if not responsive to IV IG.
	**Grade 1**	No dose modifications.	**For Grade 1:**<br>– Consider, discussing with the study physician, as needed.<br>– Care should be taken to monitor patients for sentinel symptoms of a potential decompensation as described above.<br>– Consult a neurologist.
	**Grade 2**	Hold study drug/study regimen dose until resolution to Grade ≤1.<br>Permanently discontinue study drug/study regimen if it does not resolve to Grade ≤1 within 30 days or if there are signs of respiratory insufficiency or autonomic instability.	**For Grade 2:**<br>– Considerdiscussing with the study physician, as needed.<br>– Care should be taken to monitor patients for sentinel symptoms of a potential decompensation as described above.<br>– Consulta neurologist.<br>– Sensory neuropathy/neuropathic pain may be managed by appropriate medications (e.g., gabapentin or duloxetine).

Specific Immune-Mediated Reactions			
**Adverse Events**	**Severity Grade of the Event**	**Dose Modifications**	**Toxicity Management**
			MYASTHENIA GRAVIS:<br>○ Steroids may be successfully used to treat myasthenia gravis. It is important to consider that steroid therapy (especially with high doses) may result in transient worsening of myasthenia and should typically be administered in a monitored setting under supervision of a consulting neurologist.<br>○ Patients unable to tolerate steroids may be candidates for treatment with plasmapheresis or IV IG. Such decisions are best made in consultation with a neurologist, taking into account the unique needs of each patient.<br>○ If myasthenia gravis-like neurotoxicity is present, consider starting AChE inhibitor therapy in addition to steroids. Such therapy, if successful, can also serve to reinforce the diagnosis.<br>○ Avoid medications that can worsen myasthenia gravis<br>*GUILLAIN-BARRE:*<br>○ It is important to consider here that the use of steroids as the primary treatment of Guillain-Barre is not typically considered effective.<br>○ Patients requiring treatment should be started with IV IG and followed by plasmapheresis if not responsive to IV IG.
	**Grade 3 or 4**	**For Grade 3:**<br>Hold study drug/study regimen dose until resolution to Grade ≤1.<br>Permanently discontinue study drug/study regimen if Grade 3 imAE does not resolve to Grade ≤1 within 30 days or if there are signs of respiratory insufficiency or autonomic instability.<br><br>**For Grade 4:**	**For Grade 3 or 4**<br>– Considerdiscussing with study physician, as needed.<br>– Recommend hospitalization.<br>– Monitor symptoms and obtain neurological consult.<br>*MYASTHENIA GRAVIS:*<br>○ Steroids may be successfully used to treat myasthenia gravis. They should typically be administered in a monitored setting under supervision of a consulting neurologist.<br>○ Patients unable to tolerate steroids may be candidates for treatment with plasmapheresis or IV IG.

Specific Immune-Mediated Reactions			
**Adverse Events**	**Severity Grade of the Event**	**Dose Modifications**	**Toxicity Management**
		Permanently discontinue study drug/study regimen.	○ If myasthenia gravis-like neurotoxicity present, consider starting AChE inhibitor therapy in addition to steroids. Such therapy, if successful, can also serve to reinforce the diagnosis.<br>○ Avoid medications that can worsen myasthenia gravis<br>*GUILLAIN-BARRE:*<br>○ It is important to consider here that the use of steroids as the primary treatment of Guillain-Barre is not typically considered effective.<br>○ Patients requiring treatment should be started with IV IG and followed by plasmapheresis if not responsive to IV IG.
**Myocarditis**	**Any Grade**<br>(Refer to NCI CTCAE applicable version in study protocol for defining the CTC grade/severity)	**General Guidance**<br>Discontinue drug permanently if biopsy-proven immune-mediated myocarditis.	**For Any Grade:**<br>– The prompt diagnosis of immune-mediated myocarditis is important, particularly in patients with baseline cardiopulmonary disease and reduced cardiac function.<br>– Considerdiscussing with the study physician, as needed.<br>– Monitor patients for signs and symptoms of myocarditis (new onset or worsening chest pain, arrhythmia, shortness of breath, peripheral edema). As some symptoms can overlap with lung toxicities, simultaneously evaluate for and rule out pulmonary toxicity as well as other causes (e.g., pulmonary embolism, congestive heart failure, malignant pericardial effusion). Consult a cardiologist early, to promptly assess of whether and when to complete a cardiac biopsy, including any other diagnostic procedures.<br>– Initial work-up should include clinical evaluation, BNP, cardiac enzymes, ECG, echocardiogram (ECHO), monitoring of oxygenation via pulse oximetry (resting and exertion), and additional laboratory work-up as indicated. Spiral CT or cardiac MRI can complement ECHO to assess wall motion abnormalities when needed.<br>– Patients should be thoroughly evaluated to rule out any alternative etiology (e.g., disease progression, other medications, or infections)
	**Grade 1**	No dose modifications required unless clinical suspicion is high, in which case	**For Grade 1**

Specific Immune-Mediated Reactions			
**Adverse Events**	**Severity Grade of the Event**	**Dose Modifications**	**Toxicity Management**
	(asymptomatic with laboratory (e.g., BNP) or cardiac imaging abnormalities)	hold study drug/study regimen dose during diagnostic work-up for other etiologies. If study drug/study regimen is held, resume after complete resolution to Grade 0.	- Monitor and closely follow up in 2 to 4 days for clinical symptoms, BNP, cardiac enzymes, ECG, ECHO, pulse oximetry (resting and exertion), and laboratory work-up as clinically indicated.<br>- Consider using steroids if clinical suspicion is high.
	**Grade 2, 3 or 4**<br>(Grade 2: Symptoms with mild to moderate activity or exertion)<br>(Grade 3: Severe with symptoms at rest or with minimal activity or exertion; intervention indicated)<br>(Grade 4: Life-threatening consequences; urgent intervention indicated (e.g., continuous IV therapy or mechanical hemodynamic support))	- If Grade 2 -- Hold study drug/study regimen dose until resolution to Grade 0. If toxicity rapidly improves to Grade 0, then the decision to reinitiate study drug/study regimen will be based upon treating physician's clinical judgment and after completion of steroid taper. If toxicity does not rapidly improve, permanently. discontinue study drug/study regimen.<br>If Grade 3-4, permanently discontinue study drug/study regimen.	**For Grade 2-4:**<br>– Monitor symptoms daily, hospitalize.<br>– Promptly start IV methylprednisolone 2 to 4 mg/kg/day or equivalent after Cardiology consultation has determined whether and when to complete diagnostic procedures including a cardiac biopsy.<br>– Supportive care (e.g., oxygen).If no improvement within 2 to 3 days despite IV methylprednisolone at 2 to 4 mg/kg/day, promptly start immunosuppressive therapy such as TNF inhibitors (e.g., infliximab at 5 mg/kgIV, may be repeated at 2 and 6 weeks after initial dose at the discretion of the treating provider). Caution: It is important to rule out sepsis and refer to infliximab label for general guidance before using infliximab. Infliximab is contraindicated for patients who have heart failure.<br>–
**Myositis/Polymyositis ("Poly/myositis")**	**Any Grade**<br>(Refer to NCI CTCAE applicable version in study protocol for defining the CTC grade/severity)	**General Guidance**	**For Any Grade:**<br>– Monitor patients for signs and symptoms of poly/myositis. Typically, muscle weakness/pain occurs in proximal muscles including upper arms, thighs, shoulders, hips, neck and back, but rarely affects the extremities including hands and fingers; also difficulty breathing and/or trouble swallowing can occur and progress rapidly. Increased general feelings of tiredness and fatigue may occur, and there can be new-onset falling, difficulty getting up from a fall, and trouble climbing stairs, standing up from a seated position, and/or reaching up.

Specific Immune-Mediated Reactions			
**Adverse Events**	**Severity Grade of the Event**	**Dose Modifications**	**Toxicity Management**
			– If poly/myositis is suspected, a Neurology consultation should be obtained early, with prompt guidance on diagnostic procedures. Myocarditis may co-occur with poly/myositis; refer to guidance under Myocarditis. Given breathing complications, refer to guidance under Pneumonitis/ILD. Given possibility of an existent (but previously unknown) autoimmune disorder, consider Rheumatology consultation.<br>– Consider, as necessary, discussing with the study physician.<br>– Initial work-up should include clinical evaluation, creatine kinase, aldolase, LDH, BUN/creatinine, erythrocyte sedimentation rate or C-reactive protein level, urine myoglobin, and additional laboratory work-up as indicated, including a number of possible rheumatological/antibody tests (i.e., consider whether a rheumatologist consultation is indicated and could guide need for rheumatoid factor, antinuclear antibody, anti-smooth muscle, antisynthetase [such as anti-Jo-1], and/or signal-recognition particle antibodies). Confirmatory testing may include electromyography, nerve conduction studies, MRI of the muscles, and/or a muscle biopsy. Consider Barium swallow for evaluation of dysphagia or dysphonia.<br>Patients should be thoroughly evaluated to rule out any alternative etiology (e.g., disease progression, other medications, or infections).
	**Grade 1**	- No dose modifications.	**For Grade 1:**<br>– Monitor and closely follow up in 2 to 4 days for clinical symptoms and initiate evaluation as clinically indicated.<br>– Consider Neurology consult.<br>– Consider, as necessary, discussing with the study physician.
	**Grade 2**	Hold study drug/study regimen dose until resolution to Grade ≤1.<br>- Permanently discontinue study drug/study regimen if it does not resolve to Grade ≤1 within 30 days or if there are signs of respiratory insufficiency.	**For Grade 2:**<br>– Monitor symptoms daily and consider hospitalization.<br>– Obtain Neurology consult, and initiate evaluation.<br>– Consider, as necessary, discussing with the study physician.<br>– If clinical course is rapidly progressive (particularly if difficulty breathing and/or trouble swallowing), promptly start IV methylprednisolone 2 to 4 mg/kg/day systemic steroids along with receiving input from Neurology consultant

Specific Immune-Mediated Reactions			
**Adverse Events**	**Severity Grade of the Event**	**Dose Modifications**	**Toxicity Management**
			– If clinical course is *not* rapidly progressive, start systemic steroids (e.g., prednisone 1 to 2 mg/kg/day PO or IV equivalent); if no improvement within 2 to 3 days, continue additional work up and start treatment with IV methylprednisolone 2 to 4 mg/kg/day<br>– If after start of IV methylprednisolone at 2 to 4 mg/kg/day there is no improvement within 2 to 3 days, consider starting another immunosuppressive therapy such as TNF inhibitors (e.g., infliximab at 5 mg/kg IV, may be repeated at 2 and 6 weeks after initial dose at the discretion of the treating provider). Caution: It is important to rule out sepsis and refer to infliximab label for general guidance before using infliximab.<br>–
	Grade 3 or 4	**For Grade 3:**<br>Hold study drug/study regimen dose until resolution to Grade ≤1.<br>Permanently discontinue study drug/study regimen if Grade 3 imAE does not resolve to Grade ≤1 within 30 days or if there are signs of respiratory insufficiency.<br><br>**For Grade 4:**<br>- Permanently discontinue study drug/study regimen.	**For Grade 3 or 4 (severe or life-threatening events):**<br>– Monitor symptoms closely; recommend hospitalization.<br>– Obtain Neurology consult<br>– Considerdiscussing with the study physician, as needed.<br>– Promptly start IV methylprednisolone 2 to 4 mg/kg/day systemic steroids along with receiving input from Neurology consultant.<br>– If after start of IV methylprednisolone at 2 to 4 mg/kg/day there is no improvement within 2 to 3 days, consider starting another immunosuppressive therapy such as TNF inhibitors (e.g., infliximab at 5 mg/kg IV, may be repeated at 2 and 6 weeks after initial dose at the discretion of the treating provider). Caution: It is important to rule out sepsis and refer to infliximab label for general guidance before using infliximab.<br>– Consider whether patient may require IV IG, plasmapheresis.<br>–

[a]ASCO Educational Book 2015 “Managing Immune Checkpoint Blocking Antibody Side Effects” by Michael Postow MD.
[b]FDA Liver Guidance Document 2009 Guidance for Industry: Drug Induced Liver Injury – Premarketing Clinical Evaluation.
[c]NCCN Clinical Practice Guidelines in Oncology “Management of Immunotherapy-Related Toxicities” Version 1.2020 – December 2019
AChE Acetylcholine esterase; ADL Activities of daily living; AE Adverse event; ALP Alkaline phosphatase test; ALT Alanine aminotransferase; AST Aspartate aminotransferase; BUN Blood urea nitrogen; CT Computed tomography; CTCAE Common Terminology Criteria for Adverse Events; ILD Interstitial lung disease; imAE immune-mediated adverse event; IG Immunoglobulin; IV Intravenous; GI Gastrointestinal; LFT Liver function tests; LLN Lower limit of normal; MRI Magnetic resonance imaging; NCI National Cancer Institute; NCCN National Comprehensive Cancer

Network; PJP *Pneumocystis jirovecii* pneumonia (formerly known as *Pneumocystis carinii* pneumonia); PO By mouth; T3 Triiodothyronine; T4 Thyroxine; TB Total bilirubin; TNF Tumor necrosis factor; TSH Thyroid-stimulating hormone; ULN Upper limit of normal.

Other–Immune-Mediated Reactions		
**Severity grade of the event (Refer to NCI CTCAE applicable version in study protocol for defining the CTC grade/severity)**	**Dose modifications**	**Toxicity management**
**Any Grade**	Note: It is possible that events with an inflammatory or immune mediated mechanism could occur in nearly all organs, some of them are not noted specifically in these guidelines (e.g. immune thrombocytopenia, haemolytic anaemia, uveitis, vasculitis).	– The study physician may be contacted for immune-mediated reactions not listed in the "specific immune-mediated reactions" section<br>– Thorough evaluation to rule out any alternative etiology (e.g., disease progression, concomitant medications, and infections)<br>– Consultation with relevant specialist<br>– Treat accordingly, as per institutional standard.
**Grade 1**	No dose modifications.	Monitor as clinically indicated
**Grade 2**	• Hold study drug/study regimen until resolution to ≤Grade 1 or baseline.<br>• If toxicity worsens, then treat as Grade 3 or Grade 4.<br>• Study drug/study regimen can be resumed once event stabilizes to Grade ≤1 after completion of steroid taper.<br>• Consider whether study drug/study regimen should be permanently discontinued in Grade 2 events with high likelihood for morbidity and/or mortality when they do not rapidly improve to Grade <1 upon treatment with systemic steroids and following full taper	**For Grade 2, 3 or 4:**<br>Treat accordingly, as per institutional standard, appropriate clinical practice guidelines, and other society guidelines (e.g., NCCN, ESMO)
**Grade 3**	Hold study drug/study regimen	
**Grade 4**	Permanently discontinue study drug/study regimen	

Infusion-Related Reactions		
Severity Grade of the Event (Refer to NCI CTCAE applicable version in study protocol for defining the CTC grade/severity)	**Dose Modifications**	**Toxicity Management**
**Any Grade**	General Guidance	**For Any Grade:**<br>– Manage per institutional standard at the discretion of investigator.<br>– Monitor patients for signs and symptoms of infusion-related reactions (e.g., fever and/or shaking chills, flushing and/or itching, alterations in heart rate and blood pressure, dyspnea or chest discomfort, or skin rashes) and anaphylaxis (e.g., generalized urticaria, angioedema, wheezing, hypotension, or tachycardia).
**Grade 1 or 2**	**For Grade 1:**<br>The infusion rate of study drug/study regimen may be decreased by 50% or temporarily interrupted until resolution of the event.<br>**For Grade 2:**<br>The infusion rate of study drug/study regimen may be decreased 50% or temporarily interrupted until resolution of the event.<br>Subsequent infusions may be given at 50% of the initial infusion rate.	**For Grade 1 or 2:**<br>– Acetaminophen and/or antihistamines may be administered per institutional standard at the discretion of the investigator.<br>– Consider premedication per institutional standard prior to subsequent doses.<br>– Steroids should not be used for routine premedication of Grade ≤2 infusion reactions.
**Grade 3 or 4**	**For Grade 3 or 4:**<br>Permanently discontinue study drug/study regimen.	**For Grade 3 or 4:**<br>– Manage severe infusion-related reactions per institutional standards (e.g., IM epinephrine, followed by IV diphenhydramine and famotidine, and IV glucocorticoid).

CTCAE Common Terminology Criteria for Adverse Events; IM intramuscular; IV intravenous; NCI National Cancer Institute.

Non–Immune-Mediated Reactions		
Severity Grade of the Event (Refer to NCI CTCAE applicable version in study protocol for defining **the CTC** grade/severity)	**Dose Modifications**	**Toxicity Management**
**Any Grade**	Note: Dose modifications are not required for AEs not deemed to be related to study treatment (i.e., events due to underlying disease) or for laboratory abnormalities not deemed to be clinically significant.	Treat accordingly, as per institutional standard.
**Grade 1**	No dose modifications.	Treat accordingly, as per institutional standard.
**Grade 2**	Hold study drug/study regimen until resolution to ≤Grade 1 or baseline.	Treat accordingly, as per institutional standard.
**Grade 3**	Hold study drug/study regimen until resolution to ≤Grade 1 or baseline.<br>For AEs that downgrade to ≤Grade 2 within 7 days or resolve to ≤Grade 1 or baseline within 14 days, resume study drug/study regimen administration. Otherwise, discontinue study drug/study regimen.	Treat accordingly, as per institutional standard.
**Grade 4**	Discontinue study drug/study regimen (Note: For Grade 4 labs, decision to discontinue should be based on accompanying clinical signs/symptoms, the Investigator's clinical judgment, and consultation with the Sponsor.).	Treat accordingly, as per institutional standard.

AChE=acetylcholine esterase; ADA=American Dietetic Association; ADL=Activities of daily living; ALP=alkaline phosphatase; ALT=alanine aminotransferase; AST=aspartate aminotransferase; BUN=blood urea nitrogen; CTCAE=Common Terminology Criteria for Adverse Event; GI=gastrointestinal; IDS=Infectious Disease Service; IgG=Immnoglobulin G; ILD=interstitial lung disease; IM=intramuscular; irAE=Immune-related adverse events; IV=intravenous; IVIG=intravenous immunoglobulin; LFT=liver function test; LLN=lower limit of normal; MRI=magnetic resonance imaging; PO=by mouth; TB=total bilirubin; TNF=tumor necrosis factor; TSH=thyroid stimulating hormone; ULN=upper limit of normal

Table 8: Dosing modification and toxicity management guidelines for immune-mediated, infusion-related and nonimmune- mediated reactions (durvalumab)

5.8. Management of investigational product overdose

For the purpose of the study, an overdose is defined as any dose greater than the highest dose included in the protocol. Please refer to the pharmacy manual for detailed instructions on drug preparation, storage, and administration of each product. There is currently no specific treatment in the event of overdose of Olaparib, proposed biosimilar FKB238 and Durvalumab, and possible symptoms of overdose are not established.

Any overdose or incorrect administration of study drug should be noted on the study drug administration of electronic Case Report Form (eCRF). Adverse events associated with an overdose or incorrect administration of study treatment should be recorded as an Adverse Event in eCRF.

5.9. Treatment completion

Subjects will receive Olaparib, FKB238 and Durvalumab until disease progression as per irRECIST v1.1 as assessed by the investigator, or occurrence of an unacceptable toxicity, or patient withdrawal, whichever is earlier, in a limit of 24 months.

6. CONCOMITANT AND POST-STUDY TREATMENT(S)

6.1. Permitted therapy

All medications (prescriptions or over the counter medications) continued at the start of study or started during the study or until 21 days from the end of the last protocol treatment and different from the study medication must be documented.

Any medications (with the detailed exceptions) which are considered necessary for the patient's welfare, and which it is believed will not interfere with the study medication, may be given at the discretion of the investigator, providing the medications, doses, dates and reasons for administration are recorded.

In addition, any unplanned diagnostic, therapeutic or surgical procedure performed during the study period must be recorded.

The reasons for the use, doses and dates of treatment should be recorded in the patient’s medical records and appropriate sections of the eCRF.

Patients should receive full supportive care, including transfusions of blood and blood products, antibiotics, anti-emetics, etc., when appropriate. Patients who experience infusion-associated symptoms may be treated symptomatically with acetaminophen, ibuprofen, diphenhydramine, and/or ranitidine or another H2 receptor antagonist, as per standard practice. Serious infusion-associated events manifested by dyspnea, hypotension, wheezing, bronchospasm, tachycardia, reduced oxygen saturation, or respiratory distress should be managed with supportive therapies as clinically indicated (e.g., supplemental oxygen and 2adrenergic agonists).

Patients who use oral contraceptives, hormone-replacement therapy, prophylactic or therapeutic anticoagulation therapy (such as low molecular-weight heparin or warfarin at a stable dose level), or other allowed maintenance therapy should continue their use.

6.2. Medication that may not be administered

Any concomitant therapy intended for the treatment of cancer, whether health authority-approved or experimental, is prohibited. This includes but is not limited to the following: chemotherapy, hormonal therapy, immunotherapy, radiotherapy, other targeted therapy including sunitinib, others investigational agents.

It is also not recommended to consume grapefruit juice while on olaparib therapy.

Live virus and bacterial vaccines should not be administered whilst the patient is receiving study medication and during the 30 day follow up period. An increased risk of infection by the administration of live virus and bacterial vaccines has been observed with conventional chemotherapy drugs and the effects with olaparib are unknown.

Patients should avoid concomitant use of drugs, herbal supplements and/or ingestion of foods known to modulate CYP3A4 enzyme activity from the time they enter the screening period until 30 days after the last dose of study medication.

6.3. Blood donation

Subjects should not donate blood while participating in this study and for at least 90 days following the last infusion of study drug.

6.4. Subsequent therapies for cancer

Details of first and subsequent therapies for cancer and/or details of surgery for the treatment of the cancer, after discontinuation of treatment, will be collected. Reasons for starting subsequent anti-cancer therapies will be collected and included in the exploratory assessments of OS.

7. PERMANENT DISCONTINUATION FROM STUDY TREATMENT

7.1. Permanent discontinuation of Investigational Product

Subject will receive study treatment until any of the following occur:

- Subject experiences disease progression according to irRECIST v1.1 criteria. However FKB238, Olaparib and Durvalumab treatment may be continued as long as patients are experiencing clinical benefit as assessed by the investigator.
- Subject experiences symptomatic deterioration attributed to disease progression as determined by the investigator after integrated assessment of radiographic data, biopsy results, and clinical status.
- Subject experiences unacceptable toxicity or an adverse experience that would, in the judgment of the investigator, make continued administration of the study regimen an unacceptable risk.
- Subject is considered by the investigator to be significantly non-compliant with the requirements of the protocol (e.g., subject becomes pregnant, use of another anti-cancer therapy).
- Subject is lost to follow-up.
- Investigator decision (e.g., start of second-line therapy for any reason).
- The subject could choose to discontinue all study treatments but agree to visits for follow up, or choose not to attend for visits but agree to telephone followup or agree for study team to contact their other clinicians for information on survival only. This change in level of participation should be documented in the medical records and does not require withdrawal of consent.

Patients will be permitted to continue study treatment with investigational product after irRECIST v1.1 criteria for investigator-assessed progressive disease are met, at the discretion of the investigator, if they meet all of the following criteria:

- Evidence of clinical benefit as assessed by the investigator.
- Absence of symptoms and signs (including worsening of laboratory values [e.g., new or worsening hypercalcemia]) indicating unequivocal progression of disease.
- No decline in ECOG performance status that can be attributed to disease progression.
- Absence of tumor progression at critical anatomical sites (e.g., leptomeningeal disease) that cannot be readily managed and stabilized by protocol-allowed medical interventions prior to repeat dosing.
- Patients must be provided information deferring any standard treatment options that may exist in favor of continuing IP treatment at the time of initial progression.

A patient that decides to permanently discontinue investigational product will always be asked about the reason(s) and the presence of any adverse events. If possible, they will be seen and assessed by an investigator. Adverse events will be followed up.

By discontinuing from treatment, the patient is not withdrawn from the study. Patients should be followed Unitl disease progression and OS following treatment discontinuation as per the protocol schedule.

In case of early stopping due to patient decision, it can happen that the patient will not be evaluable for the principal judgement criterion. In this case, the patient will be replaced in the study.

If a patient is withdrawn from study, see section 8.5.

Any patient discontinuing investigational product should be seen at 30 days post discontinuation for the evaluations outlined in the study schedule. The patient's tumor status should be assessed clinically and, if appropriate, disease progression should be confirmed by radiological assessment. After discontinuation of study medication, the investigator will perform the best possible observation(s), test(s) and evaluation(s) as well as give

appropriate medication and all possible measures for the safety of the patient. In addition, they will record on the eCRF the date of discontinuation, the reasons, manifestation and treatment at the time of discontinuation. If patients discontinue study treatment, the monitor must be informed immediately. Patients will be required to attend the treatment discontinuation visit.

After discontinuation of the study medication at any point in the study, all ongoing AEs or SAEs must be followed until resolution unless, in the investigator's opinion the condition is unlikely to resolve due to the patients underlying disease, or the patient is lost to follow up. All new AEs and SAEs occurring during the 30 calendar days after the last dose of study medication must be reported (if SAEs, they must be reported to ARCAGY-GINECO for safety as described in section 10.3) and followed to resolution as above. Patients should be seen at least 30 days after discontinuing study medication to collect and / or complete AE information. Any untoward event occurring subsequent to the 30-day follow-up AE reporting period that the investigator assesses as possibly related to the study medication should also be reported as an AE.

Any patient who has not yet shown objective radiological disease progression at withdrawal from IP should continue to be followed as per irRECIST v 1.1.

All patients must be followed for survival, up to the final analysis.

7.2. Treatment after discontinuation of study treatment

Once patients have been permanently discontinued from study treatment, other treatment options will be at the discretion of the investigator. The reason (s) why the investigators shifted from study treatment to a subsequent therapy will be collected.

8. STUDY CONDUCT

8.1. Patient enrolment

The Principal Investigator will:

1. Obtain signed informed consents from the potential patients before any study specific procedures are performed.
2. Assign potential patients a unique enrolment number. This number will be obtained through Interactive Voice Response System [/IWRS].
3. Determine patient's eligibility (see sections 4.1 and 4.2).
4. Obtain the inclusion code (patient number) through IWRS.

As patients are screened for the study, they must be allocated an enrolment number. This number is the patient unique identifier and is used to identify the patient on the eCRFs. If a patient withdraws from participation in the study, then her enrolment code cannot be reused.

8.2. Procedures for inclusion and initiation of investigational product

Eligibility of patients will be established before inclusion. Once the eligibility of a patient has been confirmed,
Patients will be identified to the centralized centre using date of birth (month + year).
The /IWRS Centralized Centre will inform by e-mail the Investigator and the Pharmacist of the number allocated to the patient.
It is recommended that patients begin study treatment as soon as possible after enrolment, and within 14 days.

8.3. Procedures for handling patients incorrectly enrolled or initiated on investigational product

Patients who fail to meet the inclusion/exclusion criteria should not, under any circumstance, be included or receive study medication. There can be no exception to this rule

When patients that do not meet the selection criteria are included in error or incorrectly started on treatment, or when patients subsequently fail to meet the study criteria post treatment initiation, a discussion should occur between the sponsor and the investigator regarding whether to continue or discontinue the patient from treatment. Once a decision is made, investigators need to ensure they comply with all applicable requirements for human patient protection and local ethical review.

The sponsor is to ensure all such decisions are appropriately documented. In situations when no compromise would be found, the patient will have to stop the treatment and to be withdrawn from the study.

8.4. Study treatment management

All IPs (Investigational Products) required for the completion of this study (olaparib, proposed biosimilar FKB238 and durvalumab) will be provided by the sponsor. The study drugs provided for this study will be used only as directed in the study protocol. The investigational site will acknowledge receipt of olaparib, proposed biosimilar FKB238 and durvalumab, using the IWRS to confirm the shipment condition and content. Any damaged shipments will be replaced.

The administration of all study drugs (including non-investigational products) should be recorded in the appropriate sections of the eCRF.

8.4.1. Role of the patient

Instructions should clearly be given to the patient on how and when the study treatment will be administered and how to take treatments.

Patient should follow the planned visits and perform the planned tests.

8.4.2. Role of the site study staff for the study treatment

The study site staff will have to manage the traceability of study drugs olaparib, FKB238 and durvalumab and report:

- Trial code,
- Treatment dispensations:
  - Patient study code,
  - Date of dispensation,
  - Number of Vials and batch number.
- Treatments accountability,
- Destruction of unused treatment glass vials at the end of the study after authorizatin by the sponsor. Any discrepancies must be accounted for on the appropriate forms.

8.5. Patient withdrawal from study treatment and from study

Patient may be withdrawn from the study treatment phase in the following situations:

- Patient decision to withdraw from study. The patient is at any time free to discontinue treatment, without prejudice to further treatment.
- Any medical condition that the investigator or sponsor determines may jeopardize the patient's safety if she continues in the study.
- Severe non-compliance to study protocol.
- Investigator or Sponsor determines it is in the best interest of the patient.

The primary reason for withdrawal from the study treatment should be documented on the eCRF. If a patient withdraws consent, she will be specifically asked if she is withdrawing from consent to:

- The use of their study generated data,
- The use of any collected samples (see section 12)

If a patient withdraws his consent the investigator will have to make sure that he has withdrawn his consent also for the part of the use of biological samples. For this the investigator will give him a specific form that will have to be completed and signed by the patient.
Only samples of patients who have indicated their desire not to use the biological samples will either be destroyed or returned to the investigating center.
If samples are already analyzed, ARCAGY-GINECO is not obliged to destroy the results of this research, unless the patient requires it.
The Principal Investigator has to ensure patients' withdrawal of informed consent to the use of samples is notified immediately to ARCAGY-GINECO.

The status of ongoing, lost to follow-up patients at the time of an overall survival analysis should be obtained by the site personnel by checking the patient notes, hospital records, contacting the patient's general practitioner and checking publicly available death registries.

The Steering Committee may decide to enrol up to an additional 10% of patients in order to replace withdrawn patients if the withdrawal rate is considered to be too high.

8.6. Study and site discontinuation

The sponsor has the right to terminate this study at any time. Reasons for terminating the study may include but

are not limited to the following:

- The incidence or severity of adverse events in this or other studies indicates a potential health hazard to patients,
- Patient enrolment is unsatisfactory.

The sponsor will notify to the investigator if he decides to discontinue the study.

The study will follow the FDA Risk Based Monitoring guidance. The sponsor has the right to close a site at any time. Reasons for closing a site may include but are not limited to the following:

- Excessively slow recruitment,
- Poor protocol adherence,
- Inaccurate or incomplete data recording,
- Non-compliance with the International Conference on Harmonisation (ICH) guideline for Good Clinical Practice,
- No study activity (i.e., all patients have completed and all obligations have been fulfilled).

9. COLLECTION OF STUDY VARIABLES

9.1. Recording of data

The Web Based Data Capture (WBDC) system will be used for data collection and query handling. The investigator will ensure that data are recorded on the eCRF as specified in the study protocol and in accordance with the instructions provided.

The investigator ensures the accuracy, completeness, and timeliness of the data recorded and of the provision of answers to data queries according to the Clinical Study Agreement (CSA).

9.2. Data collection at enrolment and follow-up

A study initiation visit must be conducted in each principal investigator site prior to the beginning of any study activities. A schedule for the tests and evaluations to be conducted in this study is contained in the section 3.1 of the protocol.

9.3. Enrolment / Screening procedures

The Principal Investigator/Sub-Investigator should adhere to the study plan, procedures and perform tests/observations in accordance with the protocol (see section 3.3).

9.3.1. Assessments and procedures prior to inclusion

The following assessments and procedures should be performed within 28 days before inclusion (except for the informed consent that should be signed before any study procedure).

- Informed consent,
- Date of birth (month + year),
- Medical history,
- Eligibility criteria,
- Physical examination, body weight, height, blood pressure, ECOG performance status
- Symptoms and Adverse Events: SAEs related to study procedures (e.g. blood or tumor sampling) must be reported.
- Tumor assessment: chest and abdomino-pelvic CT scan or MRI. Baseline assessments should be performed as close as possible to the start of study treatment. Scans that were performed as part of standard of care prior to signature of the informed consent form can serve as the baseline scan as long as it was performed no more than **28 days prior to start of treatment**. PET-scan cannot be used to assess disease response or progression in the absence of validated data in ovarian cancer patients treated with immunotherapy.
- Collection of archival paraffin embedded tumor tissue samples to be sent to central laboratory (mandatory. See section 12)
- Dental examination and appropriate preventive dentistry should be considered prior to starting FKB238 treatment
- Concomitant medications.
- Urinalysis
- Haematology, coagulation, serum biochemistry, hormonology
- CA-125
- Pregnancy test for women of childbearing potential (serum or urine)
- ECG
- HBV, HCV, EBV serology
- HIV test

9.3.2. Tests to be repeated prior treatment start

The following tests should be repeated **within 7 days prior the treatment start**.

- Physical examination, body weight, blood pressure, ECOG performance status (see appendix 4).
- Symptoms and Adverse Events. SAEs related to study procedures (e.g. blood or tumor sampling) must be reported.
- Concomitant medications.
- Urinalysis.
- Haematology, coagulation, serum biochemistry, hormonology
- CA-125
- Pregnancy test for women of childbearing potential (urine or serum)

9.3.3. Tests to be done prior treatment start

The following test should be done **prior treatment start**.

- Blood sample for translational research

9.4. Assessments during study treatment

9.4.1. Assessment during treatment period

The visit schedule is based on:

- **For the first cycle:** a 8 days period (1 week) for the first cycle. Patients should have a visit every week (±2 days).
- **From the second cycle:** a 21-days period (3 weeks). Patients should have a visit on day 1 every 3 weeks (±2 days).

The following assessments will be performed at time points specified in the study flowchart (see section 3.3):

- Physical examination, body weight, blood pressure, ECOG performance status (see appendix 4).
- Symptoms and Adverse Events. SAEs related to study procedures (e.g. blood or tumor sampling) must be reported.
- Concomitant medications.
- Symptom/Adverse Events.
- Urinalysis.
- Haematology, serum biochemistry, hormonology

9.4.2. Assessment every 6 weeks (each 2 cycle ±7 days) until progression or unacceptable toxicity

- Physical examination, body weight, blood pressure, ECOG performance status (see appendix 4).
- Symptoms and Adverse Events. SAEs related to study procedures (e.g. blood or tumor sampling) must be reported.
- Concomitant medications.
- Symptom/Adverse Events.
- Urinalysis.
- Pregnancy test
- Haematology, serum biochemistry, hormonology
- CA 125
- Scans of the chest, abdomen and pelvis, with other regions as clinically indicated for assessment of disease

(CT/MRI) will be performed every 6 weeks, even if treatment cycles are delayed (i.e. the scan dates are relative to the start date of study treatment regardless of the current treatment cycle). PET-scan cannot be used for disease progression assessment in this study, in absence of validated PET-scan data in ovarian cancer patients treated with chemotherapy.

9.5. Assessments for treatment discontinuation

9.5.1. Treatment discontinuation visit

For patients on study treatment, this treatment discontinuation visit has to be performed as soon as possible following the last study drug administration.

The following assessments have to be performed at this visit:

- Physical examination, body weight, blood pressure, ECOG performance status (see appendix 4).
- Symptoms and Adverse Events. SAEs related to study procedures (e.g. blood or tumor sampling) must be reported.
- Concomitant medications.
- Symptom/Adverse Events.
- Urinalysis.
- Haematology, serum biochemistry, hormonology
- CA-125

9.5.2. Safety follow-up visit (30 days after last dose administration of study treatment)

A safety follow-up visit should be conducted in addition, 30 days (± 7 days) after the last dose of study treatment, or at least before the subsequent therapy.

The following assessments will have to be performed:

- Physical examination, body weight, blood pressure, ECOG performance status (see appendix 4).
- Symptoms and Adverse Events. SAEs related to study procedures (e.g. blood or tumor sampling) must be reported.
- Concomitant medications.
- Symptom/Adverse Events.
- Urinalysis.
- Pregnancy test
- Haematology, serum biochemistry, hormonology
- CA-125
- Scans of the chest, abdomen and pelvis, with other regions as clinically indicated for assessment of disease (CT/MRI)
- ECG

9.6. Follow up visits

During the "follow-up phase", patients should have follow-up visits every 12 weeks (± 14 days), up to 12 months.

The following assessments will be performed at time points specified in the study flowchart (see section 3.3):

- Physical examination, ECOG performance status.
- Symptom/Adverse Events

- CA-125
- Pregnancy test

9.7. Survival

Follow-up after disease progression will be made according to local practice. Survival information may be obtained via telephone contact with the patient, patient's family or by contact with the patient's current physician. Survival data will be collected up to the time of the final overall survival (OS) analysis. At this point Investigators will be notified that no further data collection for the study is required.

The status of ongoing, lost to follow-up patients at the time of an overall survival analysis should be obtained by the site personnel by checking the patient notes, hospital records, contacting the patient's general practitioner and checking publicly available death registries. Data to be recorded in the eCRF.

10. STUDY ASSESSMENTS AND PROCEDURES

10.1. Tumor assessments

10.1.1. Tumor assessment scheduled

Following the baseline assessment, subsequent tumor assessments according to irRECIST v1.1 including imaging should be performed as follows:

- Every 6 weeks (2 cycles ± 7 days)

Tumor assessment will include systematically physical examination and CA-125 sample. If there is evidence of progressive disease, such as clinical progression and/or CA-125 elimination rate by Kelim model criteria i.e. CA-125 level at 8 weeks, imaging may be done for tumor assessment according to irRECIST v1.1 criteria. It is not recommended to assess CA-125 level outside of the tumor assessment visits.

NB: Clinical progression is defined as the occurence and persistance of symptoms which are considered by the investigator as undoubtfully disease-related, such as bowel occlusion, uncontrolled abdominal pain, major dyspnea induced by pleural effusion, etc. Those symptoms must have occured or have worsened despite the BOLD treatment. The decision to stop the protocol treatment will be left to investigator's discretion.

10.1.2. Imaging modalities

At baseline, the imaging modalities used for irRECIST assessment will be chest and abdomino-pelvic CT scan or MRI with other regions as clinically indicated for the assessment of disease. During follow up, the same imaging modality should be used. CT-scan of the chest will be important to perform to exclude an asymptomatic pneumonitis, in addition to chest disease progression.

PET-scan cannot be used for disease progression assessment in this study, in absence of validated PET-scan data in ovarian cancer patients treated with chemotherapy. However, PET-scan results, if available, will be collected, for exploratory comparison with those of CT scan

It is important to follow the assessment schedule as closely as possible. If scans are performed outside of scheduled visit ± 1 week window interval and the patient has not progressed, every attempt should be made to perform the subsequent scans at their scheduled time points.

Patients will be evaluated until objective radiological disease progression by irRECISTv1.1 as described in section 10.1.3)

10.1.3. Tumor evaluation

- **Immune-Related Response Criteria (irRECIST)**

Increasing clinical experience indicates that traditional response criteria may not be sufficient to fully characterize activity in this new aera of immune therapies.

IrRECIST will also be used to access the PFS within the global population and in each PD-L1 group (+VE versus –-VE). For irRECIST, only target and measurable lesions are taken into account. An additional CT scan will be required at least 4 weeks after CT scan which will detect disease progression (please refer to appendix 6). If progression is confirmed by the second scan, the date of progression should be the date of the first scan.

Following first progression (PFS1), patients should continue to be followed up for second progression and for survival as outlined in the “Patient screening and study schedule” section 3.3 and in the section 9.5. However, the management of patients will be based solely upon the results of the assessments conducted by the investigator.

10.2. Disease specific tumor marker (CA-125)

Patients will supply blood sample for CA-125 (2 mL) at the time of each tumor assessment visit (every 6 weeks) as described in study flow chart and prior to receiving study treatment (see section 3.3).

Although CA-125 is measured in this study, it will not be directly used for assessing objective response or progression and patients should be continued on treatment until objective radiological disease progression as defined by RECIST v1.1. There is insufficient data on the evolution of CA-125 levels under treatment (Olaparib, FKB 238 and Durvalumab) to make decision on disease progression based solely on CA-125 kinetics. However, in case of serological CA-125 increase as described in the Kelim guidelines (see appendix 7), CT scan/MRI may be done in order to confirm or not the disease progression, for more informations see also *You B, et al. The strong prognostic value of KELIM, a model-based parameter from CA 125 kinetics in ovarian cancer: data from CALYPSO trial (a GINECO-GCIG study) (115).*

So, it is important to follow the assessment schedule of serological CA-125 as closely as possible. If CA-125 assessment is performed outside of scheduled visit ± 1 week window interval, every attempt should be made to assess the CA-125 at the scheduled time points. Patients will be evaluated until objective disease progression (PFS1), according to irRECIST v1.1 criteria.

Further assessment of CA-125 post serological progression will be at the discretion of the investigator according to local clinical practice.

10.3. Physical examination

For timing of individual measurement refer to study flow chart (see section 3.3).

A physical examination will be performed and include an assessment of the following: general appearance, ECOG performance status, respiratory, cardiovascular, abdomen, pelvic, skin, head and neck, lymph nodes, thyroid, abdomen, musculo-skeletal (including spine and extremities) and neurological systems.

10.4. Vital signs and blood pressure

Height will be assessed at screening only. Weight will be assessed at screening only and will be repeated according to the Study Schedule if the investigator believes that it is likely to have changed significantly. Blood pressure will be measured according to the following procedures:

- In a quiet room,
- After a few minutes rest in a sitting position,
- Using preferably a semi-automatic BP recording device with an appropriate cuff size,
- At least two times 1 or 2 minutes apart.

Temperature will also be assessed.

For timings of these different assessments refer to the study flow chart (see section 3.3). The date of collection and measurement will be recorded on the appropriate eCRF. Any changes in vital signs should be recorded as an AE, if applicable.

10.5. Laboratory safety assessments

The Principal Investigator is responsible for ensuring that all staff involved in the study is familiar with the content of this section.

Blood sample for determination of haematology, coagulation, serum biochemistry, pregnancy test; urine sample for urinalysis or pregnancy test will be taken at the times indicated in the study schedule (see section 3.3).

Additional analyses may be performed if clinically indicated.

Any clinically significant abnormal laboratory values should be repeated as clinically indicated and recorded on the eCRF.

The following laboratory variables will be measured:

10.5.1. Full haematology assessment

Full haematology assessment will be done at each visit:

- Haemoglobin,
- Platelets,
- White blood cells (WBC),
- Absolute differential white cell count: neutrophils, lymphocytes, and absolute neutrophil count (ANC) should be performed at each visit and when clinically indicated. If absolute differentials not available, please provide % differentials.

10.5.2. Coagulation

Coagulation tests will be done at each visit:

- Activated partial thromboblastin time (aPTT),
- International normalized ratio (INR). Patients taking warfarin may participate in this study; however, it is recommended that prothrombin time (INR and aPTT) be monitored carefully at least once per week for the first month, then monthly if the INR is stable.

10.5.3. Biochemistry assessment

Biochemestry assessment will be done :

- Albumin and Lactate Dehydrogenase,
- Sodium, potassium, calcium, glucose
- Creatinine (at each visit),
- Total bilirubin (at each visit),
- Aspartate transaminase (AST) (at each visit), Alanine transaminase (ALT) (at each visit),
- glutamyl-transpeptidases (GGT) (at each visit),
- Alkaline phosphatase (ALP) (at each visit).

10.5.4. Serum or urine pregnancy test

Pregnancy tests on blood or urine samples will be performed for pre-menopausal women of childbearing potential (see appendix 2) at screening only, within 7 days prior to the start of study treatment. Tests will be performed by the hospital’s local laboratory. If results are positive the patient is ineligible.

In the event of a suspected pregnancy during the study, the test should be repeated. If results are positive the patient must be discontinued from the study treatment.

10.5.5. Urinalysis

Urinalysis by dipstick and proteinuria should be performed at each visit.

Microscopic analysis should be performed by the hospital’s local laboratory if required.

10.6. ECG

ECG is required within 7 days prior to starting study treatment, then should be repeated for the safety follow up visit, and more if clinically indicated. Twelve-lead ECGs will be obtained after the patient has been rested in a supine position for a few minutes. The Investigator or designated physician will review the paper copies of each of the timed 12-lead ECGs on each of the study days when they are collected.

ECGs will be recorded at 25 mm/sec. All ECGs should be assessed by the investigator as to whether they are clinically significantly abnormal / not clinically significantly abnormal. If there is a clinically significant abnormal finding, the Investigator will record it as an AE on the eCRF. The original ECG traces must be stored in the patient medical record as source data.

11. SAFETY

11.1. Definition

11.1.1. Definition of adverse events (AE)

An adverse event is the development of an undesirable medical condition or the deterioration of a pre-existing medical condition following or during exposure to a pharmaceutical product, whether or not considered causally related to the product. An undesirable medical condition can be symptoms (e.g. nausea, chest pain), signs (e.g. tachycardia, enlarged liver) or the abnormal results of an investigation (e.g. laboratory findings, electrocardiogram).

11.1.2. Definition of serious adverse events (SAE)

A serious adverse event is an AE occurring during any study phase (i.e., run-in, treatment, washout, follow-up), that fulfils one or more of the following criteria:

- Results in death (including death related to disease progression),
- Is immediately life-threatening,
- Requires in-patient hospitalization or prolongation of existing hospitalization,
- Results in persistent or significant disability/incapacity or substantial disruption of the ability to conduct normal life functions,
- Is a congenital abnormality or birth defect,
- Is an important medical event that may jeopardize the patient or may require medical intervention to prevent one of the outcomes listed above.

The expression “life threatening” is reserved to immediate vital threat, at the time of the adverse event, and regardless of consequences of a symptomatic treatment.

The term of “disability” and “incapacity” corresponds to all physical or mental disability, either temporary or permanent, clinically relevant and with physical and/or patient’s quality of life consequences.

Is considered to be “medically significant” any clinical event or laboratory result judged as serious by the investigator or/and the sponsor and not corresponding to intensity criteria defined above. Patient cannot be put at risk by it and require medical intervention to prevent from issue corresponding to one of the intensity criteria defined above (overdose, second primary cancers, intensive treatment in an emergency room can be considered as medically significant).

11.1.3. Individual case safety report (ICSR)

This refers to the format and content for the reporting of one or several suspected adverse reactions in relation to a medicinal product that occur in a single patient at a specific point of time.

If a case presents more than one adverse event, these AEs should be notified in the same SAE report to the sponsor.

11.1.4. Adverse events of specific interest

An adverse event of special interest (AESI) is one of scientific and medical interest specific to understanding of the Investigational Product and may require close monitoring and rapid communication by the investigator to the sponsor. An AESI may be serious or non-serious. The rapid reporting of AESIs allows ongoing surveillance of these events in order to characterize and understand them in association with the use of this investigational product.

All AESI should be notified to pharmacovigilance department of sponsor, regardless the causality assessment

and/or seriousness

AESIs observed with Olaparib include:

- Myelodysplastic syndrome (MDS),
- Acute myeloid leukemia (AML),
- Any other new primary cancer,
- Pneumonitis.

Note: To ensure robust safety monitoring, additional safety measures have been incorporated into this phase III protocol:

- Before inclusion into the study (eligibility criteria at section 4.1 and 4.2):
  - Patient with history of MDS/AML are excluded
  - Normal hematological values are required before inclusion into the study.
  - Patient having experienced prolonged hematotoxicity (> 2 weeks) to first-line chemotherapy are excluded.
- During treatment:

Regular blood tests are required to detect early hematological abnormality (see flow chart at section 1.6)
In case of prolonged cytopenia, patients are to be referred to an hematologist and bone marrow analysis should be considered (see section 5.6.6 dealing with the management of prolonged hematological toxicities)
If a diagnosis of MDS is confirmed, study treatment must be discontinued and the event, treatment, course and outcome must be reported as a SAE. Furthermore, MDS and/or AML is an important identified risk for Olaparib whereas pneumonitis and any other new primary cancer is an important potential risk for Olaparib. Any cases of MDS/AML will be collected all along the whole survival follow up (see section 6.4.5 dealing with the reporting of adverse event of interest).
An Independent Data Monitoring Committee (IDMC) will review the emerging safety data during the whole duration of the trial.

AESIs observed with Durvalumab include:

- Diarrhea / Colitis and intestinal perforation
- Pneumonitis / ILD
- hepatitis / transaminase increases
- Endocrinopathies (i.e. events of hypophysitis/hypopituitarism, thyroiditis, adrenal insufficiency, hyper- and hypothyroidism and type I diabetes mellitus)
- Rash / Dermatitis
- Nephritis / Blood creatinine increases
- Pancreatitis / serum lipase and amylase increases
- Myocarditis
- Myositis / Polymyositis
- Intestinal Perforations

Other inflammatory responses that are rare/less frequent with a potential immune-mediated etiology include, but are not limited to:

- Pericarditis
- Neuromuscular toxicities (such as Guillain-Barre syndrome and myasthenia gravis)
- Sarcoidosis
- Uveitis
- Other events involving the eye and skin
- Hematological events

- Rheumatological events
- Vasculitis
- Non-infectious meningitis
- Non-infectious encephalitis.

It is possible that events with an inflammatory or immune mediated mechanism could occur in nearly all organs.

- In addition, infusion-related reactions and hypersensitivity/anaphylactic reactions with a different underlying pharmacological etiology are also considered AESIs.

<u>AESIs observed with FKB238 include:</u>

- Hypertension ≥ grade 3
- Proteinuria ≥ grade 3
- GI perforation, abscesses and fistulae (any grade)
- Wound healing complications ≥ grade 3
- Haemorrhage ≥ grade 3 (any grade CNS bleeding; ≥ grade 2 haemoptysis)
- Arterial thromboembolic events (any grade)
- Venous thromboembolic events ≥ grade 3
- PRES (or RPLS; any grade)

Further information on these risks (eg. presenting symptoms) can be found in the current version of the IMP Investigator's Brochures. More specific guidelines for their evaluation and treatment are described in detail in the Dosing Modification and Toxicity Management Guidelines (see section 5.7).

11.1.5. New fact

A new fact is any new data which may lead to:

- A reassessment of the risk-benefits balance of the research or product used on the research,
- Changes in the use of the products, the conduct or documents related to the research,
- Suspend or interrupt or modify the protocol of the search or similar searches.

11.1.6. Should Not be considered as AE/SAE

The following events, in the context of this trial, <u>should not be considered as SAEs</u>. <u>No SAE form is required</u> and they are exempt from expedited reporting. They must be reported on the appropriate CRF section:

- Disease progession or death as a result of disease progression,
- Elective hospitalization and surgery for treatment of ovarian cancer, primary peritoneal cancer, fallopian tube cancer or its complications,
- Elective hospitalization to simplify treatment or procedures,
- Elective hospitalization in palliative care or respite care unit,
- Elective hospitalization for pre-existing conditions that have not been exacerbated by trial treatment and also hospitalization <24 h or not judged as experimental product related by the investigator, hospitalizations planned at the beginning of the trial and / or provided in the protocol.

For further guidance on the definition of a SAE, see appendix 8.

11.2. INVESTIGATOR RESPONSIBILITIES

- Recording of all AEs (serious or not)
- Assessment of all cases (AE's seriousness, causality assessment to IMP or non-IMP)

- Notification of serious AE, AESI and new facts to sponsor within timelines
- Follow-up of queries within timelines

11.3. Recording of adverse events

11.3.1. Time period for collection of adverse events

Adverse Events will be collected from time of signature of informed consent, throughout the treatment period and up to and including the 30-day follow-up period. All ongoing and any new AEs/SAEs identified during the 30 calendar days follow up period after last dose of study medication must be followed to resolution. After any interim analysis, any ongoing AEs/SAEs need to be unlocked and followed for resolution.
SAEs will be recorded from the time of informed consent.

After study treatment completion (i.e. after any scheduled post treatment follow-up period has ended) there is no obligation to actively report information on new AEs or SAEs occurring in study patients <u>except late adverse events of specific interest for Olaparib/FKB238 or durvalumab</u> described in section 5.9.

11.3.2. Variables

The following variables will be collect for each AE:

- AE (verbatim).
- The date when the AE started and stopped.
- Changes in CTCAE v5.0 grade.
- Whether the AE is serious or not.
- Investigator causality rating against the Investigational Product (yes or no).
- Action taken with regard to investigational product.
- Outcome.

In addition to the variables collected for each AE, the following variables will be collected for SAEs:

- Patient inclusion number
- Investigator identification
- Previous and current medical history
- Date AE met criteria for serious AE.
- Date Investigator became aware of serious AE.
- Seriousness criteria.
- Date of hospitalization.
- Date of discharge.
- Investigational treatment information
- Concomitant treatment
- Probable cause of death.
- Date of death.
- Autopsy performed.
- Causality assessment in relation to study procedure(s).
- Causality assessment in relation to other medication.
- Description of AE
- AE (verbatim),
- The date when the AE started and stopped,
- Action taken with regard to investigational product,
- Outcome,
- Whether the AE is serious or not.

All AEs spontaneously reported by the patient or reported in response to the open question from the study

personnel: 'Have you had any health problems since the previous visit/you were last asked?', or revealed by observation will be collected and recorded in the CRF.

The results from protocol mandated laboratory tests and vital signs will be summarized in the clinical study report. Deterioration as compared to baseline in protocol-mandated laboratory values, vital signs should therefore only be reported as AEs if they fulfil any of the SAE criteria or are the reason for discontinuation of treatment with the investigational product.

If deterioration in a laboratory value/vital sign/ECG is associated with clinical signs and symptoms, the sign or symptom will be reported as an AE and the associated laboratory result/vital sign/ECG will be considered as additional information. Wherever possible the reporting investigator uses the clinical, rather than the laboratory term (e.g., anaemia versus low haemoglobin value). In the absence of clinical signs or symptoms, clinically relevant deteriorations in non-mandated parameters should be reported as AE(s).

Deterioration of a laboratory value, which is unequivocally due to disease progression, should not be reported as an AE/SAE.

Any new or aggravated clinically relevant abnormal medical finding at a physical examination as compared with the baseline assessment will be reported as an AE.

11.3.3. Intensity of AEs

For each episode on an adverse event, all changes to the CTCAE version 5.0 grade attained as well as the highest attained CTC grade should be reported.

The grading scales found in the National Cancer Institute (NCI) CTCAE version 5.0 will be utilized for all events with an assigned CTCAE grading. The CTCAE reports 1 to 5 grades with specific severity clinical description of each adverse event following the general procedure:

CTCAE Grade	Equivalent to	Definition
Grade 1	Mild	• Asymptomatic or mild symptoms,<br>• Clinical or diagnostic observations only,<br>• Intervention not indicated.
Grade 2	Moderate	• Minimal, local or non-invasive intervention indicated,<br>• Limiting age-appropriate instrumental activity of daily living (ADL).
Grade 3	Severe	• Severe or medical significant but not immediately,<br>• Life-threatening hospitalization or prolongation of hospitalization indicated,<br>• Disabling,<br>• Limiting self-care ADL.
Grade 4	Life threatening	• Life-threatening consequences,<br>• Urgent intervention indicated.
Grade 5	Death	• Death related to AE.

Table 9 grade CTC and definition

For those events without assigned CTCAE grades the recommendation is that the CTCAE version 5.0 criteria that convert mild, moderate and severe events into CTCAE version 5.0 grades should be used. Only moderate and severe events will be reported.

A copy of the CTCAE version 5.0 can be downloaded from the Cancer Therapy Evaluation Program website (see

appendix 5).

11.3.4. Causality assessment

The assessment of whether there is a reasonable possibility of a causal relationship should be made by the investigator according to the following degrees of causality:

Imputability Grade	Evaluation criteria
Certain	• Clinical event or laboratory abnormalities, with plausible temporal relationship with drug administration<br>• Event that could not be explained by no illness or other drug<br>• Plausible answer to the treatment discontinuation<br>• Event identified by pharmacologic and physiopathologic point of view<br>• Positive re-challenge (where required)
Likely	• Clinical event or laboratory abnormalities, with reasonable temporal relationship with drug administration<br>• Not appear to be related to any illness or other drug<br>• Reasonable answer to the treatment discontinuation (clinical)<br>• Re-challenge information required
Possible	• Clinical event or laboratory abnormalities, with reasonable temporal relationship with drug administration<br>• Not appear to be related to any illness or other drug<br>• Not clear or lacking information concerning treatment discontinuation
Unlikely	• Clinical event or laboratory abnormalities, with improbable temporal relationship with drug administration<br>• Illness or other drug explaining plausible occurrence of the event

Table 10 imputability of SAE with experimentatl product

A guide to the interpretation of the causality question is found in appendix 8.

11.3.5. Adverse event due to lack of efficacy or to worsening disease

When there is deterioration in the ovarian cancer, for which the study treatment(s) is being used, there may be uncertainty as to whether this is lack of efficacy or an AE. In such cases, unless the Sponsor or the reporting physician considers that the study treatment contributed to the deterioration of the condition, or local regulations state to the contrary, the deterioration should be considered to be a lack of efficacy and not an AE.

Disease worsening can be considered as a worsening of a patient's condition attributable to the disease for which the investigational product is being studied. It may be an increase in the severity of the disease under study and/or increases in the signs and symptoms of the cancer. The development of new, or progression of existing metastasis to the primary cancer under study should be considered as disease worsening and not an AE. Events, which are unequivocally due to disease worsening, should not be reported as an AE during the study.

11.3.6. Overdose

Adverse reactions associated with overdose should be treated symptomatically and should be managed appropriately.

An overdose with associated AEs is recorded as the AE diagnosis/symptoms on the relevant AE modules in the CRF and on the Overdose CRF module. An overdose without associated symptoms is only reported on the Overdose CRF module. If an overdose on a study drug occurs in the course of the study, then investigators or other site personnel inform appropriate ARCAGY-GINECO representatives immediately, or no later than 24 hours of when he or she becomes aware of it. The designated ARCAGY-GINECO representative works with the investigator to ensure that all relevant information is provided.

For overdoses associated with SAE, standard reporting timelines apply. For other overdoses, reporting should be done within 30 days.

11.3.7. Pregnancy

If a patient becomes pregnant during the course of the study, the IPs should be discontinued immediately.

All cases of pregnancy (with or without AE) should be notified to the pharmacovigilance department of the sponsor.

Congenital abnormalities or birth defects and spontaneous miscarriages should be reported and handled as SAEs. The outcome of all pregnancies (spontaneous miscarriage, elective termination, ectopic pregnancy, normal birth, or congenital abnormality) should be followed up and documented even if the patient was discontinued from the study.

Note: elective abortions without complications should not be handled as AEs.

If any pregnancy occurs in the course of the study, then the Investigator or other site personnel should inform ARCAGY-GINECO immediately, but no later than 24 hours of when he or she becomes aware of it.

ARCAGY-GINECO will work with the Investigator to ensure that all relevant information is provided to the Sponsor within 1 to 5 calendar days for SAEs and within 30 days for all other pregnancies.

The same timelines apply when outcome information is available.

11.3.8. Deaths

All deaths that occur from date of signature of informed consent form, including within the protocol defined 30-days post-study follow-up period after the administration of the last dose of study treatment, must be reported as follows:

- Death which is clearly the result of disease progression should be reported to the study monitor at the next monitoring visit and should be documented in the DEATH eCRF.

Cases of death to be notified to the pharmacovigilance department of the sponsor:

- Where death is not due (or not clearly due) to progression of the disease under study, the AE causing the death must be notified as a SAE without delay. The report should contain a comment regarding the co-involvement of progression of disease, if appropriate, and should assign main and contributory causes of death. This information can be captured in the 'death eCRF'.
- Deaths with an unknown cause should always be reported as a SAE. A post mortem maybe helpful in the assessment of the cause of death, and if performed a copy of the post-mortem results should be forwarded to ARCAGY-GINECO appointed Contract Research Organization (CRO) for Safety within the usual timeframes.

11.4. Reporting of serious adverse events

11.4.1. Initial notification

From the day the informed consent form is signed and until inclusion only the SAE and AESI related to the study procedures must be reported. Non-serious AE's occurring on the same time of SAE, should be notified with the same SAE form and assessed in relation to causality assessment.

Then, from the first administration of study treatment and until 30 days after the last dose of the last treatment, or until the initiation of alternative cancer therapy, all SAEs, whether or not considered causally related to the

investigational product or to the study procedures must be reported.

All SAEs will be recorded in the e-CRF.

If any reportable SAE occurs in the course of the study, then investigators or other site personnel inform Pharmacovigilance department of ARCAGY-GINECO **immediately** when he or she becomes aware of it. An SAE form should be fulfilled, assessed and signed by the investigator and sent to the:

**ARCAGY-GINECO pharmacovigilance department**:
**By fax**: +33(0)1 84 25 40 68
Or by **e-mail**: pharmacovigilance@arcagy.org

ARCAGY-GINECO works with the investigator in order to obtain all the necessary information.

For fatal or life-threatening adverse events where important or relevant information is missing, active follow-up is undertaken immediately. Investigators or other site personnel inform ARCAGY-GINECO for Safety of any follow-up information on a previously reported SAE immediately, or no later than 24 hours of when he or she becomes aware of it. The reference document for definition of expectedness/listedness is the investigator brochure of each investigation product.

11.4.2. SAE follow up

The investigator will be assuming the appropriate medical follow-up until resolution or stabilisation of the effect or until the patient death. This could mean a prolonged follow-up after the patient study withdrawal.

The investigator has to answer to additional information requested by the ARCAGY-GINECO pharmacovigilance to document the initial notification.

The investigator forward the additional information to the pharmacovigilance department of ARCAGY-GINECO as soon as possible and no later than 48 hours after they were obtained with an SAE form (checking the box "follow up N°X" to inform it is a follow up and not an initial notification) within 48 hours after they were obtained. He also forwards the last follow up at the SAE resolution or stabilisation.

The investigator keeps the SAE documentation, in order to give more information on those previously forwarded.

12. TRANSLATIONAL RESEARCH PROGRAM

12.1. Rational and objectives

The translational research will provide informations on:

- Assessment of germline and somatic BRCA mutations and determination of HRD phenotype and mutational load by NGS
- Quantification of mutagenesis in simultaneous treatment on ctDNA
- Characterization of immune response in the tumor by Nanostring immuno-oncology panel on tumors

To realize those analysis, we will use:

FFPE tumour sample before treatment will be used to:

- A representative large panel of genes including somatic HRD phenotype (including BRCA status) will be developed to measure both TMB and HRD phenotype. We will use the idt- capture solution developed with Sophia Genetics compagny. In positive cases, a cytogentic analysis will be carried out on blood for germinal assessement. Results will be used to analyse response to olaparib.
- Characterize immune infiltrate and immune checkpoint status using Nanostring immuno-oncology panel (IO360, expression of 800 relevant genes) prior to treatment. Results will be correlated with response to durvalumab.

Blood samples will be sampled at all time points

The same representative large panel of genes including somatic HRD phenotype (including BRCA status) will be developed to measure both TMB and HRD phenotype on cfDNA. We will use the idt- capture solution developed with Sophia Genetics compagny.

- A comparison at W0 will be done between cfDNA and FFPE results for TMB burden and HRD molecular profile.
- The study of the evolution of TMB will be done according time (W0, W3 and W6).

Results will be used to analyse response to olaparib.

12.2. Samples for biomarker analysis

12.2.1. Archival tumor samples

These samples will be collected from the site pathologist. An adequately sized (minimum of 20 mm^2) tumour tissue paraffin block well representative of the tumour from resection or core biopsy of the primary tumour or metastases is mandatory for inclusion. This sample will have been collected within 3 months prior to study entry.

12.2.2. Tumor sample at disease progression

At progression (PFS1), there may be an indication for the patients to get the histologic evidence of relapse by a biopsy or an indication for a therapeutic surgical procedure at relapse. In these cases, tumor tissue could be obtained.

12.2.3. Blood samples

Blood samples will be collected at the inclusion, after 3 weeks of treatment (W3) and after six weeks of treatment (W6).

12.2.4. Samples flow shart

Time of the study	Type of sample	Optional or mandatory
Screening	• Tumor biopsy / surgical sample obtained within 3 months before inclusion	Mandatory
	• Blood sample for ctDNA, biomarker and translational research<br>• Blood sample for initial tumor mutational load	Translational study : mandatory
On treatment (on cycle 2, day 1)	• Blood sample for ctDNA, biomarker and translational research<br>• Blood sample for tumor mutational load after three weeks of treatment	Translational study : mandatory
On treatment (on cycle 3, day 1)	• Blood sample for ctDNA, biomarker and translational research<br>• Blood sample for tumor mutational load after six weeks of treatment	Translational study : mandatory
At relapse	• Tumor biopsy	Optional

Table 11 type of sample

12.3. Handling, Labelling and storage of biological samples before centralisation

The samples will be handling according to the regulatory and local rules. From collection to centralization all biological samples (blood and tumor) are under the site responsibilities'.
For Archival Tumor (FFPE), they are keeping at room temperature in the appropriate services. The identification of this sample is according to local rules. Each tumor sample has a minimum histological number.
For Tumor sample (FFPE) that has been done for the clinical study, they are keeping at room temperature in the appropriate services. The identification of this sample is according to local rules. Each tumor sample has a minimum histological number but it will be appreciate to register the patient number, site number, protocol name and sample date.
The blood samples are kept at -80°C in cryotubes with barcode in the appropriate services and conditions until centralisation. All sites must note in each cryotube the patient number, site number, protocol name and sample date.

12.4. Shipment of samples for centralisation

All tumor sample (FFPE) will be centralised in the CRB (Centre de Resource Biologique = central lab) of ARCAGY GINECO based in Institut Curie in Paris throughout the project. The Site will send the tumor sample by post mail or specific carrier. More details are available in the specific procedures of translational research which is found in investigational file.

For the blood sample, the centralisation must be done in ARCAGY GINECO central Lab (CRB) or directly in the Laboratory research.

12.5. Translational research analysis

When the sample will arrive to ARCAGY GINECO central Lab, the CRB will verify the compliance and all sample will be store in adequate condition.

The translational research laboratories will receive the samples according to the translational research plan.

The correlation between clinical data from e CRF and biological data from research laboratories can be done thank to the same identification (patient number, site number, protocol name and sample date). Some data exchange

between AG and the laboratories will take place after transfer data agreement is signed.

ARCAGY-GINECO will not provide translational research results to patients, their family members, and any insurance company.

12.6. Samples Future Use

The patient samples or the rest of the samples will be used for further translational research studies if the patient signs the consent form and agrees with this future use. This sample could be transferred to another laboratory for further research.

Biological samples for future research can be retained at ARCAGY-GINECO central lab. The results from future additional exploratory analysis should not be reported in the Clinical Study Report but separately in a Scientific Report.

13. ETHICAL AND REGULATORY REQUIREMENTS

13.1. Ethical conduct of the study

The study will be performed in accordance with ethical principles that have their origin in the Declaration of Helsinki and are consistent with International Conference on Harmonization (ICH)/Good Clinical Practice (GCP), applicable regulatory requirements.

13.2. Patient data protection

The Informed Consent Form will incorporate (or, in some cases, be accompanied by a separate document incorporating) wording that complies with relevant data protection and privacy legislation.

ARCAGY-GINECO will not provide individual genotype results to patients, their family members, any insurance company, any employer, general physician or any other third party, unless required to do so by law.

Extra precautions are taken to preserve confidentiality and prevent genetic data being linked to the identity of the patient. In exceptional circumstances, however, certain individuals might see both the genetic data and the personal identifiers of a patient. For example, in the case of a medical emergency, a sponsor Physician or an investigator might know a patient's identity and also have access to his or her genetic data. Also, Regulatory authorities may require access to the relevant files, though the patient's medical information and the genetic files would remain physically separate.

13.3. Ethical and regulatory review

An Institutional Review Board (IRB)/Ethics Committee (EC) should approve the final study protocol, including the final version of the Informed Consent Form(s) including biomarker and/or pharmacogenetic sample consents and any other written information and/or materials to be provided to the patients. The investigator will ensure the distribution of these documents to the applicable Ethics Committee, and to the study site staff.

The opinion of the IRB/Ethics Committee should be given in writing. The investigator should submit the written approval to ARCAGY-GINECO and/or CRO before enrolment of any patient into the study.

The IRB/Ethics Committee should approve all advertising used to recruit patients for the study.

ARCAGY-GINECO should approve any modifications to the Informed Consent Form that are needed to meet local requirements.

If required by local regulations, the protocol should be re-approved by the IRB/Ethics Committee annually.

Before enrolment of any patient into the study, the final study protocol, including the final version of the Informed Consent Form, is approved by the national regulatory authority or a notification to the national regulatory authority is done, according to local regulations.

ARCAGY-GINECO will handle the distribution of any of these documents to the national regulatory authorities.

ARCAGY-GINECO will provide Regulatory Authorities, Ethics Committees and Principal Investigators with safety updates/reports according to local requirements, including Suspected Unexpected Serious Adverse Reactions (SUSARs), where relevant.

Each Principal Investigator is responsible for providing the IRB/Ethics Committees with reports of any serious and unexpected adverse drug reactions from any other study conducted with the investigational product. ARCAGY-GINECO will provide this information to the Principal Investigator so that he/she can meet these reporting requirements.

13.4. Informed consent

The Principal Investigator(s) at each centre will:

- Ensure each patient is given full and adequate oral and written information about the nature, purpose, possible risk and benefit of the study, including any information on the mandatory and optional tumor biopsies.
- Ensure each patient is notified that they are free to discontinue from the study treatment at any time. If the patient has not withdrawn from the study, she will be followed according to the protocol.
- Ensure that each patient is given the opportunity to ask questions and allowed time to consider the information provided.
- Ensure each patient provides signed and dated informed consent before conducting any procedure specifically for the study.
- Ensure an original of the signed Informed Consent Form(s) is/are stored in the Investigator's Study File.
- Ensure an original of the signed Informed Consent Form is given to the patient.
- Ensure that any incentives for patients who participate in the study as well as any provisions for patients harmed as a consequence of study participation are described in the informed consent form that is approved by an Ethics Committee.

13.5. Changes to the protocol and informed consent form

Study procedures will not be changed without the mutual agreement of the Coordinating Investigator, the Scientific coordinator and ARCAGY-GINECO.

If there are any substantial changes to the study protocol, then these changes will be documented in a study protocol amendment and where required in a new version of the study protocol (Revised Clinical Study Protocol). The amendment is to be approved by the relevant Ethics Committee and if applicable, also the national regulatory authority approval, before implementation. Local requirements are to be followed for revised protocols.

ARCAGY-GINECO will distribute any subsequent amendments and new versions of the protocol to each Principal Investigator(s). If a protocol amendment requires a change to a centre's Informed Consent Form, ARCAGY-GINECO and the centre's Ethics Committee have to approve the revised Informed Consent Form before the revised form is used. If local regulations require, any administrative change will be communicated to or approved by each Ethics Committee.

13.6. Audit and inspections

Authorized representatives of ARCAGY-GINECO, a Regulatory Authority, or an Ethics Committee may perform audits or inspections at the centre, including source data verification. The purpose of an audit or inspection is to systematically and independently examine all study-related activities and documents, to determine whether these activities were conducted, and data were recorded, analyzed, and accurately reported according to the protocol, Good Clinical Practice (GCP), guidelines of the International Conference on Harmonization (ICH), and any applicable regulatory requirements. The investigator will contact ARCAGY-GINECO immediately if contacted by a regulatory agency about an inspection at the centre.

14. STUDY MANAGEMENT

14.1. Pre-study activities

Before the first patient is entered into the study, it is necessary to visit the investigational study site to:

- Determine the adequacy of the facilities.
- Determine availability of appropriate patients for the study.
- Discuss with the investigator(s) (and other personnel involved with the study) their responsibilities with regard to protocol adherence, and the responsibilities of ARCAGY-GINECO or its representatives. This will be documented in a Clinical Study Agreement (CSA) between ARCAGY-GINECO or its representatives and the investigator.

14.2. Training of study site personnel

Before the first patient is entered into the study, ARCAGY-GINECO team or its representatives or an appointed Contract Research Organization (CRO) for monitoring will review and discuss the requirements of the Clinical Study Protocol and related documents with the investigational staff and also train them in any study specific procedures and the Web Based Data Capture system(s) utilized.

The Principal Investigator will ensure that appropriate training relevant to the study is given to all of these staff, and that any new information relevant to the performance of this study is forwarded to the staff involved.

The Principal Investigator will maintain a record of all individuals involved in the study (medical, nursing and other staff).

14.3. Monitoring of the study

During the study, regular contacts with the study site, including monitoring visits, will be done to:

- Provide information and support to the investigator(s).
- Confirm that facilities remain acceptable.
- Confirm that the investigational team is adhering to the protocol, that data are being accurately and timely recorded in the eCRFs, that biological samples are handled in accordance with the Laboratory Manual and that study drug accountability checks are being performed.
- Perform source data verification (a comparison of the data in the CRFs with the patient's medical records at the hospital or practice, and other records relevant to the study) including verification of informed consent of participating patients. This will require direct access to all original records for each patient (e.g., clinic charts).
- Ensure withdrawal of informed consent to the use of the patient's biological samples is reported and biological samples are identified and disposed of/destroyed accordingly, and the action is documented, and reported to the patient.

The clinical study team or a representative for Monitoring will be available between visits if the investigator(s) or other staff at the centre needs information and advice about the study conduct.

14.4. Study agreements

The Principal Investigator of each centre should comply with all the terms, conditions, and obligations of the CSA, or equivalent, for this study. In the event of any inconsistency between this Clinical Study Protocol and the CSA, the terms of Clinical Study Protocol shall prevail with respect to the conduct of the study and the treatment of

patients and in all other respects, not relating to study conduct or treatment of patients, the terms of the CSA shall prevail.

Agreements between ARCAGY-GINECO and the Principal Investigator should be in place before any study-related procedures can take place, or patients are enrolled.

14.5. Study timetable

The study is expected to start (First patient in) in Quarter 4, 2018 and to end (Last Visite of the Last patient) by Quarter 2, 2023. The study may be terminated at individual centers if the study procedures are not being performed according to GCP, or if recruitment is slow. ARCAGY-GINECO may also terminate the entire study prematurely if concerns for safety arise within this study or in any other study with olaparib, FKB238 or durvalumab.

15. DATA MANAGEMENT

Data for this trial will be recorded via an electronic data capture BDC system using eCRF. It will be transcribed by the site from the source documents onto the eCRF.

Accurate and reliable data collection will be assured by verification and cross-checking of the eCRFs against the investigator's records by the monitoring (source document verification), and the maintenance of a drug-dispensing log.

Every investigator, study nurse, monitor or other person involved in the trial receives his or her personal login data (username and password). Access rights to the database will depend on the group affiliation. Users of the electronic data capture BDC system will receive the training materials (eCRF manual) by the data management. Every person who gets access to the system has to fill in a registration form (User-ID request) and has to confirm that they have been adequately trained. Thus, it is guaranteed that only authorized persons have access to the system to document patients in the trial.

A comprehensive validation check program utilizing front-end checks in the eCRF and backend checks in the database will verify the data, and discrepancies (queries) will be generated accordingly. These are transferred electronically to the eCRFs at the site for resolution by the investigator or designated site staff.

16. STATISTICAL METHODS AND SAMPLE SIZE DETERMINATION

A comprehensive Statistical analysis plan (SAP) for the trial will be prepared before any statistical analysis. It will include detailed information on the analysis of primary and secondary outcome measures and the definitions of major protocol deviations.

16.1. Statistical considerations

The statistical considerations are independent in the two cohorts.

16.1.1. Sample size determination

The primary objective is the rate of clinical and radiological non-progressive disease, as assessed by immune-related response criteria (irRECIST) (Wolchok et al. 2009) :

- At 3 months in the PRR cohort
- At 6 months in the PSR cohort

The sample size was calculated independently in the two cohorts. The study is based on two hypotheses for each cohort.

PRR cohort:

- The null hypothesis (p0) is 50% or lower. A rate of non-progressive disease at 3 months of 50% was considered undesirable (p0 = 50%) compared with historical control in this patient population
- The alternative hypothesis is a rate of non-progressive disease at 3 months higher than 50%.
- The positive hypothesis (p1) used for sample size calculation is 75%. A non-progressive disease rate of 75% was considered to warrant further investigations
- Sample size and cut-offs were calculated based on the exact binomial distribution. Using a one stage design and the exact binomial distribution [A'Hern, 2001], twenty three patients must be included in trial (**n=23**). This design yields a one-sided type-1 error rate of α=5% and a power of 80% when the true non progressive disease rate is 75%.

- **The total number of evaluable patients in PRR cohort will be 23. Non evaluable patients will be replaced**.

PSR cohort:

- The null hypothesis (p0) is 65%. A rate of non-progressive disease at 6 months of 65% was considered undesirable
- The alternative hypothesis is a rate of non-progressive disease at 6 months higher than 65%.
- The positive hypothesis (p1) is 84%. A non-progressive disease rate of 84% was considered to warrant further investigations.
- We again based sample size and cut-offs calculation on the exact binomial distribution. Using a one stage design and the exact binomial distribution, forty patients will be included in the trial **(n=40)**. This design yields a one-sided type-1 error rate of α=3% and a power of at least 82% when the true non progressive disease rate is 84%.

- **The total number of evaluable patients in PSR cohort will be 40. Non evaluable patients will be replaced.**

16.1.2. Decision rule

The rate of non-progressive disease (at 3 months in the PRR cohort and at 6 months in the PSR cohort) will be estimated using Kaplan-Meier estimates. Confidence intervals will be provided at the 90% 2-sided confidence level. The study conducted in the PRR cohort will be declared positive for the primary endpoint if the lower boundary of the 90% 2-sided confidence interval is higher than 50%. The study conducted in the PSR cohort will be declared positive for the primary endpoint if the lower boundary of the 90% 2-sided confidence interval is higher than 65%.

16.2. Definition of population

16.2.1. Intent-to-Treat Population

The Intent-to-treat (ITT) population is defined as all patients included in the cohort considered, regardless of whether they actually received treatment.

16.2.2. Per Protocol Population

The Per Protocol (PP) population is a subgroup of the ITT population containing all patients who do not have any major protocol violation and received study treatment Olaparib, FKB238 and durvalumab at least once. Major protocol violations will be defined in the Statistical Analysis Plan (SAP).

16.2.3. Population analysis

All efficacy analysis will be performed on the ITT population.

The safety data will be analyzed on the safety analysis set including all included patients having received at least one dose of study treatment.

16.3. Outcome measures

16.3.1. Primary outcome measure

The primary outcome measure is the rate of clinical and radiological non-progressive disease, as assessed by immune-related response criteria (irRECIST) (Wolchok et al. 2009) :

- At 3 months in the PRR cohort
- At 6 months in the PSR cohort

The rate of clinical and radiological non-progressive disease will be analysed as a time-to-event endpoint from the time of randomization to the date of progression. For this endpoint, patients who die for another cause than disease progression are censored at the time of death. Patients lost to follow-up are censored at the date of their last follow-up visit. Patients who are exempt of progression and start a next systemic therapy are censored at the date of the first administration of the next systemic therapy. Patients who are alive, exempt to progression, and exempt of next systemic therapy at their last follow-up visit are censored at that date.

16.3.2. Other secondary outcome measures

The secondary objectives of this study are to determine:

- CA 125 decline as expressed by the KELIM parameter
- Progression free survival (PFS)
- Overall survival (OS)
- Tumor response
- Toxicity as assessed by CTCAE V.5.0 scale

The translational research objectives are

- Correlate olaparib administration and durvalumab efficacy
- Correlate HRD phenotype and response to anti-PARP therapy
- Correlate tumor microenvironnement, immune check point status, and durvalumab response.

16.4. Statistical Analyses

PRR and PSR cohorts will be analysed separatetly using the same procedure at the end of the study.

16.4.1. Primary Outcome

16.4.1.1. Efficacy analyses

Population :

- Intent-to-treat population (primary analysis)
- per-protocol population (secondary analysis)

Method :

The rate of non-progressive disease (at 3 months in the PRR cohort and at 6 months in the PSR cohort) will be estimated using the Kaplan-Meier method. Confidence intervals will be provided at the 90% 2-sided confidence level. The lower limit of the 90% bilateral confidence interval being equivalent to the lower limit of the 95% unilateral confidence interval. The study conducted in the PRR cohort will be declared positive for the primary endpoint if the lower boundary of the 90% 2-sided confidence interval is higher than 50%. The study conducted in the PSR cohort will be declared positive for the primary endpoint if the lower boundary of the 90% 2-sided confidence interval is higher than 65%.

16.4.2. secondary outcomes

16.4.2.1. CA 125 decline

Population : Intent-to-treat population

Methods: The CA 125 decline wil be calculated between inclusion and end of follow-up (30 days ±7 after last treatment administration). Summarize by mean (SD) or median (25 and 75 percentils) depending if the data distribution is normal or not.

16.4.2.2. Progression free survival

Population : Intent-to-treat population

Methods: The PFS wil be calculated between the date of inclusion and the date of progression or death. The follow-up of patients still alive without progression will be censored at the date of the last follow-up visit (30 days ±7 after last treatment administration)). The PFS curves will be estimated using Kaplan-Meier methods [Kaplan, 1958].

16.4.2.3. Overall survival

Population : Intent-to-treat population

Methods: The time to death wil be calculated between the date of inclusion and the date of death. The follow-up of patients still alive will be censored at the date of the last follow-up visit (after 1 year for the two cohorts). Data on patients lost to follow-up will censored at the date of the administrative inquiry. The survival curves will be estimated using Kaplan-Meier methods [Kaplan, 1958].

16.4.2.4. Tumor response

Population : Intent-to-treat population

Methods: The tumour response will be described using the overall response categories, i.e., CR, PR, SD, NE, PD, NED. Frequency and percentage will be calculated by category.

16.4.2.5. Toxicity

Population : Safety population

Methods: Frequency and percentages of patients who had at least one AE will be calculated by category:

- Any adverse event (AE)
- Any AE related to study drug
- Any AE leading to permanent study drug discontinuation
- Any serious AE
- Any serious AE related to study drug
- Any serious AE leading to permanent study drug discontinuation

When n = 7 for PRR cohort and n = 13 for PSR cohort treatment toxicity will be analysed.
Datas will be sent to the IDMC for validation and agreement to continue the study.

16.4.2.6. IDMC

Population : Safety population
Methods: Independent Data Monitoring Committee (IDMC) will receive the treatment toxicity when n = 7 for PRR cohort and n = 13 for PSR cohort for validation and agreement to continue the study.

IDMC will be gathered upon coordinating investigator decision in case of unexpected serious adverse eventor toxic death.

16.4.3. Exploratory analyses

Analysis of the objectives and methods for the translational research

16.4.3.1. Correlation of olaparib administration and durvalumab efficacy

- **Hypothesis: Early increase of tumour mutational burden (TMB) following treatment by olaparib is predictive of a better outcome by increasing efficacy of durvalumab**

TMB is defined as the absolute number of mutations by megabase of genomic DNA. Foundation Medicine is using 5 and 20 as cut-off to stratify patients with low (<5 mut/Mb), intermediate (6-19 mut/Mb) and high (>20 mut/Mb) TMB. Efficacy of immunotherapy is increased in patients with intermediate or high TMB. Olaparib is believed to increase genomic instability by blocking base excision repair (BER) process. This should increased TMB and lead to a better priming of immune-surveillance mechanisms and response to immunotherapy.
TMB will be assessed using a large NGS panel (predicted using in house exome data on a limited set of patients) before introduction of treatment (W0), after a first cycle (W3) and a second cycle (W6) and expressed as mut/Mb. First, we will compare TMB at baseline, W3 and W6 to test whether olaparib is increasing mutational load. Patients will be classified as TMB responders if at W6 they are moving up from low to intermediate or from intermediate to high categories as defined by the FMI. For some patients, with TMB fold-change increase by at least 2- fold, (ex TMB 7 to 14, remains into the intermediate group), they will be considered also as TMB responders.
Using this stratification, we will test whether early increase in TMB is associated with a better PFS (Mann Whitney test).

16.4.3.2. Correlation between HRD phenotype and response to anti-PARPi therapy

- **Hypothesis: HRD phenotype is associated with a better response to anti-PARP therapies**

By inhibiting PARP, olaparib is blocking single-strand break DNA repair pathway. Homologous recombination (HR) is acting as a backup in case of BER defect/inhibition. HR defects are common in tumours and have been associated with predisposition to cancer (BRCA1/2 in breast and ovarian cancers for example). Besides germline alterations of BRCA genes, tumours are frequently presenting with somatic alterations of BRCA1/2 and other HR genes. HR defects were shown to sensitize tumour cells to anti-PARP/olaparib treatment. However in HR competent patients, olaparib also presented some clinical benefits.
Patients will be included in the study irrespective of their HR status. HRD phenotype will be defined using a large NGS panel and patients stratified as HR defective or competent.
We will test whether HR status is associated a better PFS using a Mann-Whitney test.

16.4.3.3. Correlation between tumor microenvironnement, immune check point status, and durvalumab response.

- **Hypothesis: Tumour microenvironment and immune checkpoint status before treatment is predictive of a better response to durvalumab.**

Cancer cells have the ability to adapt to and avoid host immune checkpoints and surveillance as a means to promote their continued survival. One of the key signaling pathways involves programmed death-1 (PD-1) and its ligands PD-L1 and PD-L2. By targeting PD-L1 durvalumab can promote an anti-tumoral immune response. Ayers

and collegues recently described a T cell-inflammed score (TIS) predictive of response to pembrolizumab in a large set of tumour types (107). TIS is based on a gene expression panel using NanoString technology (weighted sum of the housekeeping normalized values of 18 genes). Patients whose tumors had scores less than –0.3 generally showed rapid disease progression under treatment, whereas a broad spectrum of progression times was observed for higher scores. Distinct resistance mechanisms (IDO1, LAG3, MDSC or Treg infiltration, etc.) might be in play in tumors that lack T cell inflammation and in tumors showing evidence of an activated T cell infiltrate but a lack of clinical response to anti-PD1/PDL-1 therapy. TIS and alternative resistance mechanisms will be evaluated using IO360 gene expression panel (770 genes). TIS will be analyzed as a continuous quantitative variable or to stratify tumours as TIS low versus TIS high based on the defined cut-off. Expression of alternative genes will allow us to stratify patients into 2 groups: PD1/PD-L1 pathway only versus alternative escape mechanisms. Clinical benefit of durvalumab is believed to be compromised in the second category. We will test whether the TIS score is associated with a better PFS using a Cox model and a Mann-Whitney test. Association between PFS and presence of alternative escape mechanism will also be evaluated (Mann Whitney).
Exploratory biomarker data may be generated in real time during the study or retrospectively and will have unknown clinical significance. ARCAGY-GINECO will not provide exploratory biomarker results to patients, their family members, any insurance company, an employer, clinical study investigator, general physician or any other third party unless required to do so by law. The patient’s samples will not be used for any other purpose other than those dedicated to protocol related research. All this tumor material, including blood samples at progression (optional) will be collected in order to better understand the mechanism of resistance and improve the understanding of disease progression. Please refer to Investigator Laboratory Manual for further details of on study handling of blood sample, tumor tissue collection, shipping and storage.

17. STUDY COMMITTEES

17.1. Independant Data Monitoring Committee (IDMC)

The responsibilities and the schedule of the IDMC meetings will be described in a separate charter.

An Independent Data Monitoring Committee (IDMC) will be established for the BOLD trial. The IDMC will consist of designated sponsor, 2 experts in gynecology oncology and 1statistician. The IDMC will be responsible for an independent evaluation of the safety for the patients participating in the clinical trial.

No interim analysis are planned for efficacy.

The IDMC will receive the treatment toxicity when n = 7 for PRR cohort and n = 13 for PSR cohort for validation and agreement to continue the study.

The IDMC will also check the integrity and the validity of the data and the conduct of the clinical trial. The safety interim analyses and data review will be performed by the IDMC.

17.2. Steering Committee

A Steering Committee will be composed of Sponsor representatives, the Coordinating Investigator, the Biostatisticians, the Translational Research Representative, and other external participants, as needed. They will meet regularly in person or by phone to review the progress of the study within all clinical centres including recruitment, problems with protocol compliance, unexpected toxicities and need for protocol amendments.

18. PUBLICATIONS

The publication of the results of the study will be on behalf of the GINECO Group and in accordance with the ARCAGY-GINECO publication guidelines.

The Steering Committee will be in charge of the publication policy.

The authors include the Coordinating Investigator, investigators who wrote the article submitted for publication, investigators who have included the greatest number of patients, the scientific coordinator of the study, those who have made a substantial contribution to the development of the study, and statisticians who conducted the data analysis.

The participation of the IDMC/CSI will be thanked in the final publication.

For the main article and abstract, the first author is the coordinating investigator / Scientific Coordinator of the study. The following authors are the investigators by recruiting descending order.

The last author is the investigator who was involved in the design and / or obtaining the funding of the trial (if he is not the coordinating investigator / Scientific Coordinator).

The statistician is in 4th position.

The fundamental principle in ARCAGY-GINECO is the author rotation. So, a maximum of investigators can be authors, especially in conference abstracts or in sub-studies.

An exception: if the coordinator does not write the article, then it will be the one who will have written who will be the first author.

19. MEDICAL EMERGENCIES AND CONTACTS

The Principal Investigator is responsible for ensuring that procedures and expertise are available to handle medical emergencies during the study. A medical emergency usually constitutes an SAE and has to be reported as such, see section 11.

In the case of a medical emergency, the investigator may contact the Study Team members whose contacts are reported in the following table.

NAME	ROLE IN THE STUDY	ADDRESS & TELEPHONE NUMBER
Pr Léa PAYEN / Dr Jonathan LOPEZ	Scientific coordinator	Institut de Cancérologie des Hospices Civils de Lyon<br>165 Chemin du Grand Revoyet,<br>69495 PIERRE BENITE<br>Phone: + 33 (0)4 78 86 16 07
Pr Gilles FREYER	Coordinating Investigator	Institut de Cancérologie des Hospices Civils de Lyon<br>165 Chemin du Grand Revoyet,<br>69495 PIERRE BENITE<br>Phone: + 33 (0)4 78 86 43 18
Bénédicte VOTAN	General Manager of ARCAGY-GINECO	ARCAGY-GINECO<br>Hôtel-Dieu Hôpital<br>Parvis de Notre Dame<br>Place Jean-Paul II<br>75181 PARIS Cedex 04<br>France<br>Phone: + 33 (0)1 42 34 83 23
Sébastien ARMANET	Global Clinical Operation Manager	
Michèle TORRES-MACQUE	Clinical Project Manager	
Edna MORA	Pharmacovigilance Manager	

20. LIST OF REFERENCES

1. Reid BM, Permuth JB, Sellers TA. Epidemiology of ovarian cancer: a review. Cancer Biol Med. 2017 Feb;14(1):9-32.
2. Jayson GC, Kohn EC, Kitchener HC, Ledermann JA. Ovarian cancer. Lancet. 2014. Oct 11;384(9951):1376-88.
3. 2bis.Reid BM, Permuth JB, Sellers TA. Epidemiology of ovarian cancer: a review. Cancer Biol Med. 2017 Feb;14(1):9-32.
4. Jayson GC, Kohn EC, Kitchener HC, Ledermann JA. Ovarian cancer. Lancet. 2014. Oct 11;384(9951):1376-88.
5. Pujade-Lauraine E, Hilpert F, Weber B, Reuss A, Poveda A, Kristensen G, Sorio R, Vergote I, Witteveen P, Bamias A, Pereira D, Wimberger P, Oaknin A, Mirza MR, Follana P, Bollag D, Ray-Coquard I. Bevacizumab combined with chemotherapy for platinum-resistant recurrent ovarian cancer: The AURELIA open-label randomized phase III trial. J Clin Oncol. 2014 May 1;32(13):1302-1308.
6. Ledermann JA, Harter P, Gourley C, Friedlander M, Vergote I, Rustin G, Scott CL, Meier W, Shapira-Frommer R, Safra T, Matei D, Fielding A, Spencer S, Dougherty B, Orr M, Hodgson D, Barrett JC, Matulonis U. Olaparib maintenance therapy in patients with platinum-sensitive relapsed serous ovarian cancer: a preplanned retrospective analysis of outcomes by BRCA status in a randomised phase 2 trial. Lancet Oncol. 2014; 15(8):852-861.
7. Stone B, et al. Serologic analysis of ovarian tumor antigens reveals a bias toward anti-gens encoded on 17q. Int J Cancer 2003 ;104 :73-84.
8. Schlienger K, Chu CS, Woo EY, et al. TRANCE and CD40 ligand-matured dendritic cells reveal MHC class I-restricted T cells specific for autologous tumor in late-stage ovarian cancer patients. Clin Cancer Res 2003;9:1517-1527.
9. Goodell V, Salazar LG, Urban N, et al. Antibody immunity to the p53 oncogenic protein is a prognostic indicator in ovarian cancer. J Clin Oncol 2006;24:762-768.
10. Reuschenbach M, von Knebel Doeberitz M, Wentzensen N. A systematic review of humoral immune responses against tumor antigens. Cancer Immunol Immunother 2009;58:1535-1544
11. Zhang L, Conejo-Garcia JR, Katsaros D, Gimotty PA, Massobrio M, Regnani G, Makrigiannakis A, Gray H, Schlienger K, Liebman MN, Rubin SC, Coukos G. Intratumoral T cells, recurrence, and survival in epithelial ovarian cancer. N Engl J Med 2003;348:203-213.
12. Hwang WT, Adams SF, Tahirovic E, et al. Prognostic significance of tumor-infiltrating T cells in ovarian cancer:a meta-analysis. Gynecol Oncol 2012;124:192-198.
13. Curiel TJ, Coukos G, Zou L, Alvarez X, Cheng P, Mottram P, Evdemon-Hogan M, Conejo-Garcia JR, Zhang L, Burow M, Zhu Y, Wei S, Kryczek I, Daniel B, Gordon A, Myers L, Lackner A, Disis ML, Knutson KL, Chen L, Zou W. Specific recruitment of regulatory T cells in ovarian carcinoma fosters immune privilege and predicts reduced survival. Nat Med 2004;10:942-949.
14. Sato E, Olson SH, Ahn J, Bundy B, Nishikawa H, Qian F, Jungbluth AA, Frosina D, Gnjatic S, Ambrosone C, Kepner J, Odunsi T, Ritter G, Lele S, Chen YT, Ohtani H, Old LJ, Odunsi K. Intraepithelial CD8+ tumor-infiltrating lymphocytes and a high CD8+/regulatory T cell ratio are associated with favorable prognosis in ovarian cancer. Proc Natl Acad Sci U S A 2005;102:18538-18543.
15. Zhang QW, Liu L, Gong CY, Shi HS, Zeng YH, Wang XZ, Zhao YW, Wei YQ. Prognostic significance of tumor-associated macrophages in solid tumor: a meta-analysis of the literature.PLoS One. 2012;7(12): e50946.
16. Blank C, Gajewski TF, Mackensen A. Interaction of PD-L1 on tumor cells with PD-1 on tumor-specific T cells as a mechanism of immune evasion: implications for tumor immunotherapy. Cancer Immunol Immunother 2005;54:307-314.
17. Keir ME, Butte MJ, Freeman GJ, Sharpe AH. PD-1 and its ligands in tolerance and immunity. Annual Rev Immunol 2008;26:677-704.
18. Butte MJ, Keir ME, Phamduy TB, Sharpe AH, Freeman GJ. Programmed death-1 ligand 1 interacts specifically with the B7-1 costimulatory molecule to inhibit T cell responses. Immunity 2007;27:111-122.
19. Yang J, Riella LV, Chock S, Liu T, Zhao X, Yuan X, Paterson AM, Watanabe T, Vanguri V, Yagita H, Azuma M, Blazar BR, Freeman GJ, Rodig SJ, Sharpe AH, Chandraker A, Sayegh MH. The novel costimulatory programmed death ligand1/B7.1 pathway is functional in inhibiting alloimmune responses in vivo. J Immunol 2011;187:1113-1119.
20. Chen DS, Irving BA, and Hodi FS. Molecular pathways: next-generation immunotherapy-inhibiting programmed death-ligand 1 and programmed death-1. Clin Cancer Res 2012;18:6580-6587.
21. Blank C, Mackensen A. Contribution of the PD-L1/PD-1 pathway to T-cell exhaustion: an update on implications for

chronic infections and tumor evasion. Cancer Immunol Immunother 2007;56:739-745.

22. Hamanishi J, Mandai M, Iwasaki M, Okazaki T, Tanaka Y, Yamaguchi K, Higuchi T, Yagi H, Takakura K, Minato N, Honjo T, Fujii S. Programmed cell death 1 ligand 1 and tumor-infiltrating CD8+ T lymphocytes are prognostic factors of human ovarian cancer. Proc Natl Acad Sci U S A 2007;104:3360-3365.
23. Kryczek I, Liu R, Wang G, Wu K, Shu X, Szeliga W, Vatan L, Finlayson E, Huang E, Simeone D, Redman B, Welling TH, Chang A, Zou W. FOXP3 defines regulatory T cells in human tumor and autoimmune disease. Cancer Res. 2009 May 1;69(9):3995-4000.
24. Curiel TJ, Wei S, Dong H, Alvarez X, Cheng P, Mottram P, Krzysiek R, Knutson KL, Daniel B, Zimmermann MC, David O, Burow M, Gordon A, Dhurandhar N, Myers L, Berggren R, Hemminki A, Alvarez RD, Emilie D, Curiel DT, Chen L, Zou W. Blockade of B7 -H1 improves myeloid dendritic cell-mediated antitumor immunity. Nat Med 2003;9:562-567.
25. Hamid O, Robert C, Daud A, Hodi FS, Hwu WJ, Kefford R, Wolchok JD, Hersey P, Joseph RW, Weber JS, Dronca R, Gangadhar TC, Patnaik A, Zarour H, Joshua AM, Gergich K, Elassaiss-Schaap J, Algazi A, Mateus C, Boasberg P, Tumeh PC, Chmielowski B, Ebbinghaus SW, Li XN, Kang SP, Ribas A. Safety and tumor responses with lambrolizumab (anti-PD-1) in melanoma. N Engl J Med 2013 ;369(2):134-144
26. Hodi FS, O'Day SJ, McDermott DF, Weber RW, Sosman JA, Haanen JB, Gonzalez R, Robert C, Schadendorf D, Hassel JC, Akerley W, van den Eertwegh AJ, Lutzky J, Lorigan P, Vaubel JM, Linette GP, Hogg D, Ottensmeier CH, Lebbé C, Peschel C, Quirt I, Clark JI, Wolchok JD, Weber JS, Tian J, Yellin MJ, Nichol GM, Hoos A, Urba WJ. Improved survival with ipilimumab in patients with metastatic melanoma. N Engl J Med. 2010 Aug 19;363(8):711-723
27. Robert C, Thomas L, Bondarenko I, O'Day S, Weber J, Garbe C, Lebbe C, Baurain JF, Testori A, Grob JJ, Davidson N, Richards J, Maio M, Hauschild A, Miller WH Jr, Gascon P, Lotem M, Harmankaya K, Ibrahim R, Francis S, Chen TT, Humphrey R, Hoos A, Wolchok JD. Ipilimumab plus dacarbazine for previously untreated metastatic melanoma. N Engl J Med. 2011 ;364(26):2517-2526
28. Topalian SL, Hodi FS, Brahmer JR, Gettinger SN, Smith DC, McDermott DF, Powderly JD, Carvajal RD, Sosman JA, Atkins MB, Leming PD, Spigel DR, Antonia SJ, Horn L, Drake CG, Pardoll DM, Chen L, Sharfman WH, Anders RA, Taube JM, McMiller TL, Xu H, Korman AJ, Jure-Kunkel M, Agrawal S, McDonald D, Kollia GD, Gupta A, Wigginton JM, Sznol M. Safety, activity, and immune correlates of anti-PD-1 antibody in cancer. N Engl J Med. 2012 ;366(26):2443-54
29. Brahmer JR, Drake CG, Wollner I, Powderly JD, Picus J, Sharfman WH, Stankevich E, Pons A, Salay TM, McMiller TL, Gilson MM, Wang C, Selby M, Taube JM, Anders R, Chen L, Korman AJ, Pardoll DM, Lowy I, Topalian SL. Phase I study of single-agent anti-programmed death-1 (MDX-1106) in refractory solid tumors: safety, clinical activity, pharmacodynamics, and immunologic correlates. J Clin Oncol. 2010 Jul 1;28(19):3167-75
30. Motzer RJ, Rini BI, McDermott DF, et al: Nivolumab for metastatic renal cell carcinoma: Results of a randomized, dose-ranging phase II trial. J Clin Oncol 32:5s, 2014 (suppl; abstr 5009).
31. Petrylak DP, et al. A phase Ia study of atezolimumab (anti-PDL1): updated response and survival data in urothelial bladder cancer (UBC); J Clin Oncol 33, 2015 (suppl; abstr 4501)
32. 30bis. Junzo Hamanishi, Masaki Mandai, Takafumi Ikeda, Manabu Minami, Atsushi Kawaguchi, Toshinori Murayama, Masashi Kanai, Yukiko Mori, Shigemi Matsumoto, Shunsuke Chikuma, Noriomi Matsumura, Kaoru Abiko, Tsukasa Baba, Ken Yamaguchi, Akihiko Ueda, Yuko Hosoe, Satoshi Morita, Masayuki Yokode, Akira Shimizu, Tasuku Honjo, Ikuo Konishi. Safety and Antitumor Activity of Anti–PD-1 Antibody, Nivolumab, in Patients With Platinum-Resistant Ovarian Cancer. J Clin Oncol. 2015 Dec 1;33(34):4015-22. doi: 10.1200/JCO.2015.62.3397. Epub 2015 Sep 8.
33. Disis M, Patel MR, Pant S, Infante JR, Lockhart AC, Kelly K, Beck JT, Gordon MS, Weiss GJ, Ejadi S, Taylor, Anja von Heydebreck MH, Chin KM, Cuillerot JM, Gulley JL. Avelumab (MSB0010718C), an anti-PD-L1 antibody, in patients with previously treated, recurrent or refractory ovarian cancer: A phase Ib, open-label expansion trial. J Clin Oncol 33, 2015 (suppl, abstract 5509)
34. Duncan TJ, Al-Attar A, Rolland P, Scott IV, Deen S, Liu DT, Spendlove I, Durrant LG. Vascular endothelial growth factor expression in ovarian cancer: a model for targeted use of novel therapies? Clin Cancer Res. 2008 May 15;14(10):3030-5.
35. Bamias A, Koutsoukou V, Terpos E, Tsiatas ML, Liakos C, Tsitsilonis O, Rodolakis A, Voulgaris Z, Vlahos G, Papageorgiou T, Papatheodoridis G, Archimandritis A, Antsaklis A, Dimopoulos MA. Correlation of NK T-like CD3+CD56+ cells and CD4+CD25+(hi) regulatory T cells with VEGF and TNFalpha in ascites from advanced ovarian cancer: Association with platinum resistance and prognosis in patients receiving first-line, platinum-based

chemotherapy. Gynecol Oncol. 2008 Feb;108(2):421-7.

36. Burger RA, Brady MF, Bookman MA, Fleming GF, Monk BJ, Huang H, Mannel RS, Homesley HD, Fowler J, Greer BE, Boente M, Birrer MJ, and Liang SX. Incorporation of bevacizumab in the primary treatment of ovarian cancer. N. Engl. J. Med. 2011; 365(26):2473-2483.

37. Perren TJ, Swart AM, Pfisterer J, Ledermann JA, Pujade-Lauraine E, Kristensen G, Carey MS, Beale P, Cervantes A, Kurzeder C, du Bois A, Sehouli J, Kimmig R, Stahle A, Collinson F , Essapen S, Gourley C , Lortholary A, Selle F, Mirza,MR, Leminen A, Plante M, Stark D, Qian W, Parmar MK, and Oza AM. A phase 3 trial of bevacizumab in ovarian cancer. N. Engl. J. Med. 2011; 365(26), 2484-2496.

38. K.J. Kim, B. Li, J. Winer, M. Armanini, N. Gillett, H.S. Phillips, N. Ferrara Inhibition of vascular endothelial growth factor-induced angiogenesis suppresses tumor growth in vivo Nature, 362 (1993), pp. 841–844

39. R.S. Warren, H. Yuan, M.R. Matli, N.A. Gillett, N. Ferrara. Regulation by vascular endothelial growth factor of human colon cancer tumorigenesis in a mouse model of experimental liver metastasis. J. Clin. Invest., 95 (1995), pp. 1789–1797

40. O. Melnyk, M.A. Shuman, K.J. Kim. Vascular endothelial growth factor promotes tumor dissemination by a mechanism distinct from its effect on primary tumor growth P. Borgstrom, K.J. Hillan, P. Sriramarao, N. Ferrara Complete inhibition of angiogenesis and growth of microtumors by anti-vascular endothelial growth factor neutralizing antibody: novel concepts of angiostatic therapy from intravital videomicroscopy Cancer Res., 56 (1996), pp. 4032–4039

41. P. Borgstrom, K.J. Hillan, P. Sriramarao, N. Ferrara. Complete inhibition of angiogenesis and growth of icrotumors by anti-vascular endothelial growth factor neutralizing antibody: novel concepts of angiostatic therapy from intravital videomicroscopy Cancer Res., 56 (1996), pp. 4032–4039

42. P. Borgstrom, M.A. Bourdon, K.J. Hillan, P. Sriramarao, N. Ferrara. Neutralizing anti-vascular endothelial growth factor antibody completely inhibits angiogenesis and growth of human prostate carcinoma micro tumors in vivo Prostate, 35 (1998), pp. 1–10 B. Millauer, L.K. Shawver, K.H. Plate, W. Risau, A. Ullrich. Glioblastoma growth inhibited in vivo by a dominant-negative Flk-1 mutant Nature, 367 (1994), pp. 576–579

43. L.M. Strawn, G. McMahon, H. App, R. Schreck, W.R. Kuchler, M.P. Longhi, T.H. Hui, C. Tang, A. Levitzki, A. Gazit, I. Chen, G. Keri, L. Orfi, W. Risau, I. Flamme, A. Ullrich, K.P. Hirth, L.K. Shawver. Flk-1 as a target for tumor growth inhibition Cancer Res., 56 (1996), pp. 3540–3545

44. J.M. Wood, G. Bold, E. Buchdunger, R. Cozens, S. Ferrari, J. Frei, F. Hofmann, J. Mestan, H. Mett, T. O'Reilly, E. Persohn, J. Rosel, C. Schnell, D. Stover, A. Theuer, H. Towbin, F. Wenger, K. Woods-Cook, A. Menrad, G. Siemeister, M. Schirner, K.H. Thierauch, M.R. Schneider, J. Drevs, G. Martiny-Baron, F. Totzke PTK787/ZK 222584, a novel and potent inhibitor of vascular endothelial growth factor receptor tyrosine kinases, impairs vascular endothelial growth factor-induced responses and tumor growth after oral administration

45. Oku, J.G. Tjuvajev, T. Miyagawa, T. Sasajima, A. Joshi, R. Joshi, R. Finn, K.P. Claffey, R.G. Blasberg. Tumor growth modulation by sense and antisense vascular endothelial growth factor gene expression: effects on angiogenesis, vascular permeability, blood volume, blood flow, fluorodeoxyglucose uptake, and proliferation of human melanoma intracerebral xenografts Cancer Res., 58 (1998), pp. 4185–4192

46. J. Holash, S. Davis, N. Papadopoulos, S.D. Croll, L. Ho, M. Russell, P. Boland, R. Leidich, D. Hylton, E. Burova, E. Ioffe, T. Huang, C. Radziejewski, K. Bailey, J.P. Fandl, T. Daly, S.J. Wiegand, G.D. Yancopoulos, J.S. Rudge. VEGF-trap: a VEGF blocker with potent antitumor effects Proc. Natl. Acad. Sci. USA, 99 (2002), pp. 11393–11398

47. L.G. Presta, H. Chen, S.J. O'Connor, V. Chisholm, Y.G. Meng, L. Krummen, M. Winkler, N. Ferrara.Humanization of an anti-VEGF monoclonal antibody for the therapy of solid tumors and other disorders Cancer Res., 57 (1997), pp. 4593–4599

48. H.P. Gerber, J. Kowalski, D. Sherman, D.A. Eberhard, N. Ferrara. Complete inhibition of rhabdomyosarcoma xenograft growth and neovascularization requires blockade of both tumor and host vascular endothelial growth factor Cancer Res., 60 (2000), pp. 6253–6258

49. A.M. Ryan, D.B. Eppler, K.E. Hagler, R.H. Bruner, P.J. Thomford, R.L. Hall, G.M. Shopp, C.A. O'Neill. Preclinical safety evaluation of rhuMAbVEGF, an antiangiogenic humanized monoclonal antibody. Toxicol. Pathol., 27 (1999), pp. 78–86

50. D.T. Shima, A. Gougos, J.W. Miller, M. Tolentino, G. Robinson, A.P. Adamis, P.A. D'Amore. Cloning and mRNA expression of vascular endothelial growth factor in ischemic retinas of Macaca fascicularis. Invest. Ophthalmol. Vis. Sci., 37 (1996), pp. 1334–1340

51.H.P. Gerber, T.H. Vu, A.M. Ryan, J. Kowalski, Z. Werb, N. Ferrara.VEGF couples hypertrophic cartilage remodeling, ossification and angiogenesis during endochondral bone formation. Nat. Med., 5 (1999), pp. 623–628

52.Ryan AM1, Eppler DB, Hagler KE, Bruner RH, Thomford PJ, Hall RL, Shopp GM, O'Neill CA. Preclinical safety evaluation of rhuMAbVEGF, an antiangiogenic humanized monoclonal antibody. Toxicol Pathol. 1999 Jan-Feb;27(1):78-86.

53.Burger RA, Brady MF, Bookman MA, Fleming GF, Monk BJ, Huang H, Mannel RS, Homesley HD, Fowler J, Greer BE, Boente M, Birrer MJ, and Liang SX. Incorporation of bevacizumab in the primary treatment of ovarian cancer. N. Engl. J. Med. 2011; 365(26):2473-2483.

54.Perren TJ, Swart AM, Pfisterer J, Ledermann JA, Pujade-Lauraine E, Kristensen G, Carey MS, Beale P, Cervantes A, Kurzeder C, du Bois A, Sehouli J, Kimmig R, Stahle A, Collinson F , Essapen S, Gourley C , Lortholary A, Selle F, Mirza,MR, Leminen A, Plante M, Stark D, Qian W, Parmar MK, and Oza AM. A phase 3 trial of bevacizumab in ovarian cancer. N. Engl. J. Med. 2011; 365(26), 2484-2496

55.Pujade-Lauraine E, Hilpert F, Weber B, Reuss A, Poveda A, Kristensen G, Sorio R, Vergote I, Witteveen P, Bamias A, Pereira D, Wimberger P, Oaknin A, Mirza MR, Follana P, Bollag D, Ray-Coquard I. Bevacizumab combined with chemotherapy for platinum-resistant recurrent ovarian cancer: The AURELIA open-label randomized phase III trial. J Clin Oncol. 2014 May 1;32(13):1302-1308.

56.Poveda AM, Selle F, Hilpert F, Reuss A, Savarese A, Vergote I, Witteveen P, Bamias A, Scotto N, Mitchell L, Pujade-Lauraine E. Bevacizumab Combined With Weekly Paclitaxel, Pegylated Liposomal Doxorubicin, or opotecan in Platinum-Resistant Recurrent Ovarian Cancer: Analysis by Chemotherapy Cohort of the Randomized Phase III AURELIA Trial. J Clin Oncol. 2015 Nov 10;33(32):3836-8.

57.Aghajanian C, Blank SV, Goff BA, Judson PL, Teneriello MG, Husain A, Sovak MA, Yi J, Nycum LR. OCEANS: a randomized, double-blind, placebo-controlled phase III trial of chemotherapy with or without bevacizumab in patients with platinum-sensitive recurrent epithelial ovarian, primary peritoneal, or fallopian tube cancer. J Clin Oncol. 2012 ;30(17):2039-2045

58.Helleday T. The underlying mechanism for the PARP and BRCA synthetic lethality: clearing up the misunderstandings. Mol. Oncol. 2011; 5(4):387-393

59.Murai J, Huang SY, Das BB, Renaud A, Zhang Y, Doroshow JH, Ji J, Takeda S, Pommier Y. Trapping of PARP1 and PARP2 by Clinical PARP Inhibitors. Cancer Res. 2012; 72(21):5588-5599.

60.Ledermann J, Harter P, Gourley C, Friedlander M, Vergote I, Rustin G, Scott C, Meier W, Shapira-Frommer R, Safra T, Matei D, Macpherson E, Watkins C, Carmichael J, and Matulonis U. Olaparib maintenance therapy in platinum-sensitive relapsed ovarian cancer. N. Engl. J. Med. 2012; 366(15), 1382-1392.

61.Oza AM, Cibula D, Oaknin A, Poole CJ, Mathijssen R, Sonke G, Colombo N, Špacek J, Vuylsteke P, Hirte HW, Mahner S, Plante M, Schmalfeldt B, Mackay H, Rowbottom J, Tchakov I, Friedlander M. Olaparib plus paclitaxel plus carboplatin (P/C) followed by olaparib maintenance treatment in patients (pts) with platinum-sensitive recurrent serous ovarian cancer (PSR SOC): A randomized, open-label phase II study. Meeting: 2012 ASCO Annual Meeting Citation: J Clin Oncol 30, 2012 (suppl; abstr 5001)

62.Vay A, Kumar S, Seward S, Semaan A, Schiffer CA, Munkarah AR, Morris RT. Therapy-related myeloid leukemia after treatment for epithelian ovarian carcinoma: an epidemiological analysis. Gynecol Oncol. 2011; 123(3), 456-460.

63.Morton LM, Dores GM, Tucker MA, Kim CJ, Onel K, Gilbert ES, Fraumeni JF Jr, Curtis RE. Evolving risk of therapy-related acute myeloid leukemia following cancer chemotherapy among adults in the United States, 1975-2008. Blood. 2013; 121(15):2996-3004.

64.Gaymes TJ, Shall S, Farzaneh F, Mufti GJ. Chromosomal instability syndromes are sensitive to poly-ADP-ribose polymerase inhibitors. Haematologica 2008; 93(12):1886-1889

65.Ledermann J, Harter P, Gourley C, Friedlander M, Vergote I, Rustin G, Scott CL, Meier W, Shapira-Frommer R, Safra T, Matei D, Fielding A, Spencer S, Dougherty B, Orr M, Hodgson D, Barrett JC, Matulonis U. Olaparib maintenance therapy in patients with platinum-sensitive relapsed serous ovarian cancer: a preplanned retrospective analysis of outcomes by BRCA status in a randomised phase 2 trial. Lancet Oncol. 2014; 15(8):852-861.

66.Mu CY, Huang JA, Chen Y, Chen C, Zhang XG. High expression of PD-L1 in lung cancer may contribute to poor prognosis and tumor cells immune escape through suppressing tumor infiltrating endritic cells maturation. Med Oncol. 2011 Sep;28(3):682-8.

67.Krambeck AE, Dong H, Thompson RH, Kuntz SM, Lohse CM, Leibovich BC, et al. Survivin and B7-H1 are collaborative predictors of survival and represent potential therapeutic targets for patients with renal cell carcinoma. Clin Cancer

Res. 2007;13(6):1749-56.

68. Thompson RH, Gillett MD, Chevillem JC, Lohsem CM, Dongm H, Webster WS, et al. Costimulatory molecule B7-H1 in primary and metastatic clear cell renal cell carcinoma. Cancer. 2005;104(10):2084-91.
69. Thompson RH, Kuntz SM, Leibovich BC, Dong H, Lohse CM, Webster WS, et al. Tumor B7- H1 is associated with poor prognosis in renal cell carcinoma patients with long-term followup. Cancer Res. 2006;66(7):3381-5.
70. Loos M, Giese NA, Kleeff J, Giese T, Gaida MM, Bergmann F, et al. Clinical significance and regulation of the costimulatory molecule B7-H1 in pancreatic cancer. Cancer Lett. 2008;268(1):98-109.
71. Nomi T, Sho M, Akahori T, Hamada K, Kubo A, Kanehiro H, et al. Clinical significance and therapeutic potential of the programmed death-1 ligand/programmed death-1 pathway in human pancreatic cancer. Clin Cancer Res. 2007 Apr 1;13(7):2151-7.
72. Wang L, Ma Q, Chen X, Guo K, Li J, Zhang M. Clinical significance of B7-H1 and B7-1 expressions in pancreatic arcinoma. World J Surg. 2010;34(5):1059-65.
73. Hamanishi J, Mandai M, Iwasaki M, Okazaki T, Tanaka Y, Yamaguchi K, et al. Programmed cell death 1 ligand 1 and tumor-infiltrating CD8+ T lymphocytes are prognostic factors of human ovarian cancer. Proc Nat Acad Sci U S A. 2007;104(9):3360-5.
74. Keir ME, Butte MJ, Freeman GJ, Sharpe AH. PD-1 and its ligands in tolerance and immunity. Annu Rev Immunol. 2008;26:677-704.
75. Park J, Omiya R, Matsumura Y, Sakoda Y, Kuramasu A, Augustine MM, et al. B7-H1/CD80 interaction is required for the induction and maintenance of peripheral T-cell tolerance. Blood. 2010;116(8):1291-8.
76. Zou W, Chen L. Inhibitory B7-family molecules in the tumour microenvironment. Nat Rev Immunol. 2008;8(6):467-77.
77. Keir ME, Butte MJ, Freeman GJ, Sharpe AH. PD-1 and its ligands in tolerance and immunity. Annu Rev Immunol. 2008;26:677-704.
78. Park J, Omiya R, Matsumura Y, Sakoda Y, Kuramasu A, Augustine MM, et al. B7-H1/CD80 interaction is required for the induction and maintenance of peripheral T-cell tolerance. Blood. 2010;116(8):1291-8.
79. Dean, E. et al. Phase I study to assess the safety and tolerability of olaparib in combination with bevacizumab in patients with advanced solid tumors. Br. J. Cancer 106, 468–474 (2012).
80. Liu, J. F. et al. Combination cediranib and olaparib versus olaparib alone for women with recurrent platinum-sensitive ovarian cancer: a randomised phase 2 study. Lancet Oncol. 15, 1207–1214 (2014).
81. Wolchok, J. D. et al. Guidelines for the evaluation of immune therapy activity in solid tumors: immune-related response criteria. Clin. Cancer Res. Off. J. Am. Assoc. Cancer Res. 15, 7412–7420 (2009).
82. Lee JM, Cimino-Mathews A, Peer CJ, Zimmer A, Lipkowitz S, Annunziata CM, Cao
83. L, Harrell MI, Swisher EM, Houston N, Botesteanu DA, Taube JM, Thompson E,
84. Ogurtsova A, Xu H, Nguyen J, Ho TW, Figg WD, Kohn EC. Safety and Clinical
85. Activity of the Programmed Death-Ligand 1 Inhibitor Durvalumab in Combination
86. With Poly (ADP-Ribose) Polymerase Inhibitor Olaparib or Vascular Endothelial
87. Growth Factor Receptor 1-3 Inhibitor Cediranib in Women's Cancers: A
88. Dose-Escalation, Phase I Study. J Clin Oncol. 2017 Jul 1;35(19):2193-2202
89. Chan N, Pires IM, Bencokova Z, Coackley C, Luoto KR, Bhogal N, Lakshman M, Gottipati P, Oliver FJ, Helleday T, Hammond EM, Bristow RG. Contextual synthetic lethality of cancer cell kill based on the tumor microenvironment. Cancer Res. 2010; 70(20):8045-8054.
90. Hegan DC, Lu Y, Stachelek GC, Crosby ME, Bindra RS, and Glazer PM. Inhibition of poly(ADP-ribose) polymerase down-regulates BRCA1 and RAD51 in a pathway mediated by E2F4 and p130. Proc. Natl. Acad. Sci. U. S.A 2010; 107(5), 2201-2206.
91. Dean E, Middleton MR, Pwint T, Swaisland H, Carmichael J, Goodege-Kunwar P, and Ranson M. Phase I study to assess the safety and tolerability of olaparib in combination with bevacizumab in patients with advanced solid tumours. Br. J. Cancer 2012; 106(3), 468-474.
92. Farmer H, McCabe N, Lord CJ, Tutt AN, Johnson DA, Richardson TB, et al. Targeting the DNA repair defect in BRCA mutant cells as a therapeutic strategy. Nature. 2005 Apr 14;434(7035):917-21.
93. Chatzinikolaou G, Karakasilioti I, Garinis GA. DNA damage and innate immunity: links and trade-offs. Trends

Immunol. 2014;35(9):429-35.

94. Tang ML, Khan MK, Croxford JL, Tan KW, Angeli V, Gasser S. The DNA damage response induces antigen presenting cell-like functions in fibroblasts. Eur J Immunol. 2014 Apr;44(4):1108-18. doi: 10.1002/eji.201343781. Epub 2014 Feb 16.

95. Kroemer G, Galluzzi L, Kepp O, Zitvogel L. Immunogenic cell death in cancer therapy. Annu Rev Immunol. 2013;31:51-72.

96. Ledermann J, Harter P, Gourley C, Friedlander M, Vergote I, Rustin G, et al. Olaparib maintenance therapy in patients with platinum-sensitive relapsed serous ovarian cancer: a preplanned retrospective analysis of outcomes by BRCA status in a randomised phase 2 trial. Lancet Oncol. 2014 Jul;15(8):852-61.

97. Ledermann JA, Harter P, Gourley C, Friedlander M, Vergote I, Rustin G, et al. Overall survival (OS) in patients (pts) with platinum-sensitive relapsed serous ovarian cancer (PSR SOC) receiving olaparib maintenance monotherapy: An interim analysis. J Clin Oncol 2016;34 (suppl; abstr 5501).

98. Mulligan JM, Hill LA, Deharo S, Irwin G, Boyle D, Keating KE, et al. Identification and validation of an anthracycline/cyclophosphamide-based chemotherapy response assay in breast cancer. J Natl Cancer Inst. 2014 Jan;106(1)

99. Higuchi T, Flies DB, Marjon NA, Mantia-Smaldone G, Ronner L, Gimotty PA, et al. CTLA-4 Blockade Synergizes Therapeutically with PARP Inhibition in BRCA1-Deficient Ovarian Cancer. Cancer Immunol Res. 2015;3(11):1257-68.

100. Huang J, Wang L, Cong Z, Amoozgar Z, Kiner E, Xing D, et al.. The PARP1 inhibitor BMN 673 exhibits immunoregulatory effects in a Brca1(-/-) murine model of ovarian cancer. Biochem Biophys Res Commun. 2015;463(4):551-6.

101. Wolchok, J. D. et al. Guidelines for the evaluation of immune therapy activity in solid tumors: immune-related response criteria. Clin. Cancer Res. Off. J. Am. Assoc. Cancer Res. 15, 7412–7420 (2009).

102. Mirza MR1, Monk BJ1, Herrstedt J1, Oza AM1, Mahner S1, Redondo A1, Fabbro M1, Ledermann JA1, Lorusso D1, Vergote I1, Ben-Baruch NE1, Marth C1, Mądry R1, Christensen RD1, Berek JS1, Dørum A1, Tinker AV1, du Bois A1, González-Martín A1, Follana P1, Benigno B1, Rosenberg P1, Gilbert L1, Rimel BJ1, Buscema J1, Balser JP1, Agarwal S1, Matulonis UA1; ENGOT-OV16/NOVA Investigators. Niraparib Maintenance Therapy in Platinum-Sensitive, Recurrent Ovarian Cancer. N Engl J Med. 2016 Dec 1;375(22):2154-2164. Epub 2016 Oct 7.

103. Disis M, Patel MR, Pant S, Infante JR, Lockhart AC, Kelly K, Beck JT, Gordon MS, Weiss GJ, Ejadi S, Taylor, Anja von Heydebreck MH, Chin KM, Cuillerot JM, Gulley JL. Avelumab (MSB0010718C), an anti-PD-L1 antibody, in patients with previously treated, recurrent or refractory ovarian cancer: A phase Ib, open-label expansion trial. J Clin Oncol 33, 2015 (suppl, abstract 5509)

104. Ledermann JA, Harter P, Gourley C, Friedlander M, Vergote I, Rustin G, Scott CL, Meier W, Shapira-Frommer R, Safra T, Matei D, Fielding A, Spencer S, Dougherty B, Orr M, Hodgson D, Barrett JC, Matulonis U. Olaparib maintenance therapy in patients with platinum-sensitive relapsed serous ovarian cancer: a preplanned retrospective analysis of outcomes by BRCA status in a randomised phase 2 trial. Lancet Oncol. 2014; 15(8):852-861.

105. Antonia SJ1, Villegas A1, Daniel D1, Vicente D1, Murakami S1, Hui R1, Yokoi T1, Chiappori A1, Lee KH1, de Wit M1, Cho BC1, Bourhaba M1, Quantin X1, Tokito T1, Mekhail T1, Planchard D1, Kim YC1, Karapetis CS1, Hiret S1, Ostoros G1, Kubota K1, Gray JE1, Paz-Ares L1, de Castro Carpeño J1, Wadsworth C1, Melillo G1, Jiang H1, Huang Y1, Dennis PA1, Özgüroğlu M1; PACIFIC Investigators. Durvalumab after Chemoradiotherapy in Stage III Non-Small-Cell Lung Cancer. N Engl J Med. 2017 Nov 16;377(20):1919-1929. doi: 10.1056/NEJMoa1709937. Epub 2017 Sep 8.

106. Mantia-Smaldone G, Ronner L, Blair A, Gamerman V, Morse C, Orsulic S, Rubin S, Gimotty P, Adams S. The immunomodulatory effects of pegylated liposomal doxorubicin are amplified in BRCA1--deficient ovarian tumors and can be exploited to improve treatment response in a mouse model. Gynecol Oncol. 2014;133(3):584-90

107. Mantia-Smaldone G, Ronner L, Blair A, Gamerman V, Morse C, Orsulic S, Rubin S, Gimotty P, Adams S. The immunomodulatory effects of pegylated liposomal doxorubicin are amplified in BRCA1--deficient ovarian tumors and can be exploited to improve treatment response in a mouse model. Gynecol Oncol. 2014;133(3):584-90

108. Narwal R, Roskos LK, Robbie GJ. Population pharmacokinetics of sifalimumab, an investigational anti-interferon-alpha monoclonal antibody, in systemic lupus erythematosus. Clin Pharmacokinet. 2013 Nov;52(11):1017–27.

109. Ng CM, Lum BL, Gimenez V, Kelsey S, Allison D. Rationale for fixed dosing of pertuzuma in cancer patients based on population pharmacokinetic analysis. Pharm Res. 2006;23(6):1275–84.

110. Wang DD, Zhang S, Zhao H, Men AY, Parivar K. Fixed dosing versus body size-based dosing of monoclonal antibodies in adult clinical trials. J Clin Pharmacol. 2009;49(9):1012–24.

111. Zhang S, Shi R, Li C, Parivar K, Wand DD. Fixed dosing versus body size-based dosing of therapeutic peptides and proteins in adults. J Clin Pharmacol. 2012;52(1):18–28.

112. Zhang S, Shi R, Li C, Parivar K, Wand DD. Fixed dosing versus body size-based dosing of therapeutic peptides and proteins in adults. J Clin Pharmacol. 2012;52(1):18–28.

113. Bang YJ, Im SA, Lee KW, Cho JY, Song EK, Lee KH, et al. Randomized, double-blind Phase II trial with prospective classification by ATM protein level to evaluate the efficacy and tolerability of olaparib plus paclitaxel in patients with recurrent or metastatic gastric cancer. J Clin Oncol. 2015 Aug 17.

114. Wolchok, J. D. et al. Guidelines for the evaluation of immune therapy activity in solid tumors: immune-related response criteria. Clin. Cancer Res. Off. J. Am. Assoc. Cancer Res. 15, 7412–7420 (2009).

115. Ayers M, Lunceford J, Nebozhyn M, Murphy E, Loboda A, Kaufman DR, Albright A, Cheng JD, Kang SP, Shankaran V, Piha-Paul SA, Yearley J, Seiwert TY, Ribas A, McClanahan TK. IFN-γ-related mRNA profile predicts clinical response to PD-1 blockade. J Clin Invest. 2017 Aug 1;127(8):2930-2940

116. You B, Colomban O, Heywood M, Lee C, Davy M, Reed N, Pignata S, Varsellona N, Emons G, Rehman K, Steffensen KD, Reinthaller A, Pujade-Lauraine E, Oza A. The strong prognostic value of KELIM, a model-based parameter from CA 125 kinetics in ovarian cancer: data from CALYPSO trial (a GINECO-GCIG study). Gynecol Oncol. 2013 Aug;130(2):289-94. doi: 10.1016/j.ygyno.2013.05.013. Epub 2013 May 18.

.

21. APPENDICES

Appendix 1: CHMP Recommandation on Olaparib

Appendix 2: Definition of non-childbearing potential and Acceptable birth control methods

Appendix 3: FIGO staging (2014) Ovary, Fallopian tube, Peritoneum Cancer

Appendix 4: ECOG Performance Status

Appendix 5: Common Terminology Criteria for Adverse Events V 5.0 (CTCAE)

Appendix 6: Radiological disease assessment according to RECIST v. 1.1

Appendix 7: Progression based on serum CA-125 levels according to Kelim, a model-based parameter from CA-125 kinetics

Appendix 8: Additional safety information

EUROPEAN MEDICINES AGENCY
SCIENCE MEDICINES HEALTH

22 February 2018
EMA/CHMP/86143/2018
Committee for Medicinal Products for Human Use (CHMP)

Summary of opinion[1] (post authorisation)

Lynparza

olaparib

On 22 February 2018, the Committee for Medicinal Products for Human Use (CHMP) adopted a positive opinion recommending a change to the terms of the marketing authorisation for the medicinal product Lynparza. The marketing authorisation holder for this medicinal product is AstraZeneca AB.

The CHMP recommended the approval of a new presentation and new strengths: Lynparza 100 mg and 150 mg tablets, which will allow for fewer dose units. The dose of the currently authorised form (50 mg capsules) is 8 capsules taken twice a day, whereas the dose for the new form will be 2 tablets twice a day.

The indication of Lynparza 100 mg and 150 mg tablet will be as follows:

> "Lynparza is indicated as monotherapy for the maintenance treatment of adult patients with platinum-sensitive relapsed high-grade epithelial ovarian, fallopian tube, or primary peritoneal cancer who are in response (complete or partial) to platinum-based chemotherapy."

For information, the indication for Lynparza capsules will remain as follows:

> "Lynparza is indicated as monotherapy for the maintenance treatment of adult patients with platinum-sensitive relapsed *BRCA*-mutated (germline and/or somatic) high grade serous epithelial ovarian, fallopian tube, or primary peritoneal cancer who are in response (complete response or partial response) to platinum-based chemotherapy."

Detailed recommendations for the use of this product will be described in the updated summary of product characteristics (SmPC), which will be published in the revised European public assessment report (EPAR), and will be available in all official European Union languages after a decision on this change to the marketing authorisation has been granted by the European Commission.

[1] Summaries of positive opinion are published without prejudice to the Commission decision, which will normally be issued 67 days from adoption of the opinion

30 Churchill Place • Canary Wharf • London E14 5EU • United Kingdom
Telephone +44 (0)20 3660 6000 **Facsimile** +44 (0)20 3660 5520
Send a question via our website www.ema.europa.eu/contact

An agency of the European Union

© European Medicines Agency, 2018. Reproduction is authorised provided the source is acknowledged.

Appendix 2 – Definition of non-childbearing potential and Acceptable birth control methods

The following restrictions apply while the patient is receiving study treatment and for the specified times before and after:

1. Female subjects of childbearing potential who are sexually active with a non-sterilized male partner must use at least one highly effective method of contraception from the time of screening and must agree to continue using such precautions for 180 days after the last dose of investigational product. Male partners of a female subject must use male condom plus spermicide throughout this period. Cessation of birth control after this point should be discussed with a responsible physician. Not engaging in sexual activity for the total duration of the trial and the drug washout period is an acceptable practice; however, occasional abstinence, the rhythm method, and the withdrawal method are not acceptable methods of contraception. Female patients should refrain from breastfeeding throughout this period.
2. Non-sterilized male subjects who are sexually active with a female partner of childbearing potential must use male condom plus spermicide from screening through 180 days after the last dose of investigational product. Not engaging in sexual activity for the total duration of the trial and the drug washout period is an acceptable practice; however, occasional abstinence, the rhythm method, and the withdrawal method are not acceptable methods of contraception. Male patients should refrain from sperm donation throughout this period. Female partners of a male subject must use a highly effective method of contraception throughout this period.
3. Females of childbearing potential are defined as those who are not surgically sterile (ie, bilateral tubal ligation, bilateral oophorectomy, or complete hysterectomy) or post-menopausal (defined as 12 months with no menses without an alternative medical cause).

   Women <50 years of age would be considered post-menopausal if they have been amenorrheic for 12 months or more following cessation of exogenous hormonal treatments and if they have luteinizing hormone and follicle-stimulating hormone levels in the post-menopausal range for the institution or underwent surgical sterilization (bilateral oophorectomy or hysterectomy).

   Women ≥50 years of age would be considered post-menopausal if they have been amenorrheic for 12 months or more following cessation of all exogenous hormonal treatments, had radiation-induced menopause with last menses >1 year ago, had chemotherapy-induced menopause with last menses >1 year ago, or underwent surgical sterilization (bilateral oophorectomy, bilateral salpingectomy or hysterectomy).

4. Highly effective methods of contraception are described in Table XX. A highly effective method of contraception is defined as one that results in a low failure rate (i.e. less than 1% per year) when used consistently and correctly. Note that some contraception methods are not considered highly effective (e.g. male or female condom with or without spermicide; female cap, diaphragm, or sponge with or without spermicide; non-copper containing intrauterine device; progestogen-only oral hormonal contraceptive pills where inhibition of ovulation is not the primary mode of action [excluding Cerazette/desogestrel which is considered highly effective]; and triphasic combined oral contraceptive pills).

Table XX Highly Effective[a] Methods of Contraception

BARRIER/INTRAUTERINE METHODS	HORMONAL METHODS
• Copper T intrauterine device<br>• Levonorgesterel-releasing intrauterine system (eg, Mirena®)[b]	• "Implants": Etonogestrel-releasing implants: e.g. Implanon® or Norplan®<br>• "Intravaginal Devices": Ethinylestradiol/etonogestrel-releasing intravaginal devices: e.g. NuvaRing®<br>• "Injection": Medroxyprogesterone injection: e.g. Depo-Provera®<br>• "Combined Pill": Normal and low dose combined oral contraceptive pill<br>• "Patch": Norelgestromin/ethinylestradiol-releasing transdermal system: e.g. Ortho Evra®<br>• "Minipill[c]": Progesterone based oral contraceptive pill using desogestrel e.g. Cerazette®

[a] Highly effective (i.e. failure rate of <1% per year)

[b] This is also considered a hormonal method

[c] Cerazette® is currently the only highly effective progesterone based pill

APPENDIX 3 - ECOG PERFORMANCE STATUS

SCALE	DESCRIPTION
0	Fully active, able to carry on all pre-disease performance without restriction.
1	Restricted in physically strenuous activity but ambulatory and able to carry out work of a light or sedentary nature, e.g., light house work, office work.
2	Ambulatory and capable of all selfcare but unable to carry out any work activities. Up and about more than 50% of waking hours.
3	Capable of only limited selfcare, confined to bed or chair more than 50% of waking hours.
4	Completely disabled. Cannot carry on any selfcare. Totally confined to bed or chair.

Oken MM, Creech RH, Tormey DC, et al. Toxicity And Response Criteria Of The Eastern Cooperative Oncology Group. Am J Clin Oncol 1982; 5(6):649-655

APPENDIX 4 – FIGO STAGING (2014) OVARY, FALLOPIAN TUBE, PERITONEUM CANCER

FIGO Guidelines: Staging classification for cancer of the ovary, fallopian tube, and peritoneum. Jaime Prat for the FIGO Committee on Gynecologic Oncology. International Journal of Gynecology & Obstetrics. Vol 124, (1), January 2014, 1–5

DESCRIPTOR	STAGE	DESCRIPTOR	STAGE
I: Tumor confined to ovaries or fallopian tube(s)	I	III: Tumor involves 1 or both ovaries or fallopian tubes, or primary peritoneal cancer, with cytologically or histologically confirmed spread to the peritoneum outside the pelvis and/or metastasis to the retroperitoneal lymph nodes	III
IA: Tumor limited to 1 ovary (capsule intact) or fallopian tube; no tumor on ovarian or fallopian tube surface; nomalignant cells in the ascites or peritoneal washings	IA	IIIA1: Positive retroperitoneal lymph nodes only (cytologically or histologically proven)	IIIA1
IB: Tumor limited to both ovaries (capsules intact) or fallopian tubes; no tumor on ovarian or fallopian tube surface; no malignant cells in the ascites or peritoneal washings	IB	IIIA1(i) Metastasis up to 10 mm in greatest dimension	IIIA1(i)
IC: Tumor limited to 1 or both ovaries or fallopian tubes, with any of the following	IC	IIIA1(ii) Metastasis more than 10 mm in greatest dimension	IIIA1(ii)
IC1: Surgical spill	IC1	IIIA2: Microscopic extrapelvic (above the pelvic brim) peritoneal involvement with or without positive retroperitoneal lymph nodes	IIIA2
IC2: Capsule ruptured before surgery or tumor on ovarian or fallopian tube surface	IC2	IIIB: Macroscopic peritoneal metastasis beyond the pelvis up to 2 cm in greatest dimension, with or without metastasis to the retroperitoneal lymph nodes	IIIB
IC3: Malignant cells in the ascites or peritoneal washings	IC3	IIIC: Macroscopic peritoneal metastasis beyond the pelvis more than 2 cm in greatest dimension, with or without metastasis to the retro-peritoneal lymph nodes (includes extension oftumour to capsule of liver and spleen without parenchymal involvement of either organ)	IIIC
II: Tumor involves 1 or both ovaries or fallopian tubes with pelvic extension (below pelvic brim) or primary peritoneal cancer	II	IV: Distant metastasis excluding peritoneal metastases	IV
IIA: Extension and/or implants on uterus and/or fallopiantubes and/ or ovaries	IIA	IVA: Pleural effusion with positive cytology	IVA
IIB: Extension to other pelvic intraperitoneal tissues	IIB	IVB: Parenchymal metastases and metastases to extra-abdominal organs (including inguinal lymphnodes and lymph nodes outside of the abdominal cavity)	IVB

APPENDIX 5 – COMMON TERMINOLOGY CRITERIA FOR ADVERSE EVENTS V.5.0 (CTCAE)

Refer to NCI CTCAE v.5.0 online at the following NCI website:

https://ctep.cancer.gov/protocolDevelopment/electronic_applications/docs/CTCAE_v5_Quick_Reference_8.5x11.pdf

Cancer Therapy Evaluation Program

Common Terminology Criteria for Adverse Events v.5.0
(Publish Date November 27, 2017)

APPENDIX 6 – IMMUNE-RELATED RESPONSE CRITERIA DERIVED FROM RECIST V.1.1 (IRRECIST)

Increasing clinical experience indicates that traditional response criteria may not be sufficient to fully characterize activity in this new era of targeted therapies and/or biologics.

This is particularly true for immunotherapeutic agents such as anti-CTLA4 and anti-PD1\anti PDL1 which exert the antitumor activity by augmenting activation and proliferation of T cells, thus leading to tumor infiltration by T cells and tumor regression rather than direct cytotoxic effects (Hoos et al, 2010; Hodi et al Proc Natl Acad Sci U S A 2008). Clinical observations of patients with advanced melanoma treated with ipilimumab, for example, suggested that conventional response assessment criteria such as Response Evaluation Criteria in Solid Tumors (RECIST) and WHO criteria are not sufficient to fully characterize patterns of tumor response to immunotherapy because tumors treated with immunotherapeutic agents may show additional response patterns that are not described in these conventional criteria.

Furthermore, the conventional tumor assessment criteria (RECIST and WHO criteria) have been reported as not capturing the existence of a subset of patients who have an OS similar to those who have experienced CR or PR but were flagged as PD by WHO criteria.

On these grounds, a tumor assessment system has been developed that incorporates these delayed or flare-type responses into the RECIST 1.1 criteria (irRECIST).

For irRECIST, only target and measurable lesions are taken into account. In contrast to the RECIST 1.1, the irRECIST criteria

- require confirmation of both progression and response by imaging at least 4 weeks from the date first documented, and
- do not necessarily score the appearance of new lesions as progressive disease if the sum of lesion diameters of target lesions (minimum of 10 mm per lesion, maximum of 5 target lesions, maximum of 2 per organ) and measurable new lesions does not increase by ≥20%.

The same method of assessment and the same technique should be used to characterize each identified and reported target lesion(s) at baseline and throughout the trial.

irRECIST criteria are defined as follows:

- Overall immune-related complete response (irCR): Complete disappearance of all lesions (whether measurable or not) and no new lesions. All measurable lymph nodes also must have a reduction in short axis to <10 mm.
- Overall immune-related partial response (irPR): Sum of the longest diameters of target and new measurable lesions decreases ≥30%.
- Overall immune-related stable disease (irSD): Sum of the longest diameters of target and new measurable lesions neither irCR, irPR, (compared to baseline) or immune-related progressive disease (irPD, compared to nadir).
- Overall immune-related progressive disease (irPD): Sum of the longest diameters of target and new measurable lesions increases ≥ 20% (compared to nadir), confirmed by a repeat, consecutive observation at least 4 weeks from the date first documented.

New measurable lesions: Incorporated into tumor burden.

New non-measurable lesions: Do not define progression but preclude irCR.

Overall responses derived from changes in index, non-index, and new lesions are outlines in Table below

Table 12 Overall Response Derived from Changes in Index, Non-index and New Lesions

Measurable response	Non-measurable response		Overall response using irRECIST[b]
Index and New Measurable Lesions (Tumor Burden)[a]	Non-Index Lesions	Measurable Lesions	
Decrease 100%	Absent	Absent	irCR
Decrease 100%	Stable	Any	irPR
Decrease 100%	Unequivocal progression	Any	irPR
Decrease ≥30%	Absent/stable	Any	irPR
Decrease ≥30%	Unequivocal progression	Any	irPR
Decrease <30% and increase <20%	Absent/stable	Any	irSD
Decrease <30% and increase <20%	Unequivocal progression	Any	irSD
Increase ≥20%	Any	Any	irPD

a. Decrease assessed relative to baseline.

b. Response (irCR and irPR) and progression (irPD) must be confirmed by a second, consecutive assessment at least 4 weeks apart.

APPENDIX 7 – PROGRESSION BASED ON SERUM CA-125 LEVELS ACCORDING TO KELIM, A MODEL-BASED PARAMETER FROM CA-125 KINETICS

Progression or recurrence based on serum CA-125 levels will be defined on the basis of a progressive serial elevation of serum CA-125, according to Kelim.

You B, Colomban O, Heywood M, Lee C, Davy M, Reed N, Pignata S, Varsellona N, Emons G, Rehman K, Steffensen KD, Reinthaller A, Pujade-Lauraine E, Oza A. The strong prognostic value of KELIM, a model-based parameter from CA 125 kinetics in ovarian cancer: data from CALYPSO trial (a GINECO-GCIG study). Gynecol Oncol. 2013 Aug;130(2):289-94.

Parallel evolution of serum tumor marker titers and cancer growth has been reported in many cancers . The kinetics of CA-125 titers during treatment, considered as a reflection of treatment efficacy, has been largely investigated. The lack of utility of RECIST criteria in many patients with ovarian cancer has amplified the interest for CA-125 kinetic studies. Different approaches meant to analyze serum CA-125 kinetics and their prognostic values have been reported in the literature. Heterogeneities and simplifications in methodologies used to assess kinetic parameters may explain the inconsistencies in results and their limited ability to produce clinically and prognostically relevant kinetic investigations. Only CA-125 response definition based on CA-125 percentage decrease was adopted by GCIG for assessment of treatment efficacy in clinical studies. However based on the CALYPSO data, Lee et al., showed unexpected results about the earlier decline of CA-125 with CP as compared with CD. They concluded that early CA-125 decrease and early response were poor predictors of treatment benefit. Consistent with their report, we found that more patients presented favorable CA-125 response according to GCIG criteria in the CP arm. Lee et al., provided potential explanations for the poor surrogacy of CA-125 decline slope/percentage in the CALYPSO trial. In particular, more frequent dosing of paclitaxel (every 3 weeks) than PLD (every 4 weeks) might explain earlier CA-125 decline.

Mathematical modeling of CA-125 kinetics during the first 50 treatment days was feasible on an individual basis. Based on the structure of our semi-mechanistic model, it was possible to separate information about CA-125 production, elimination, and indirect treatment effect. Higher values of treatment kinetic parameter K had favorable predictive value regarding PFS using univariate analyses. However, although it was not possible to compare K and other modeled kinetic parameters in both arms using statistical tests due to data shrinkage , we found a trend for higher values of K in the paclitaxel arm. It might be concordant with the assumption made by Lee et al., because K relates to lag-time required to observe CA-125 decline after treatment administration. Paclitaxel dosed more frequently may have induced quicker decrease of CA-125, which was a poor predictor of treatment efficacy. Another kinetic parameter linked to tumor marker elimination, KELIM, that can be assimilated to CA-125 clearance, harbored a strong independent predictive value. When compared to previous publications, incorporation of KELIM in multivariate Cox model led to elimination of tumor bulk-related covariates, such as metastatic site number and tumor size. It suggests that prognostic information linked to cancer size might also be contained in the value of this modeled kinetic parameter. Given that KELIM is determined dynamically based on mathematical equations describing CA-125 kinetic profiles, it is independent on selected time points used to calculate it. KELIM may be a novel relevant predictive factor of treatment efficacy. Confirmation of these results in independent cohorts of patients is warranted.

Model-based strategies may also be used in drug development, as there is a need for identification of strategies to facilitate early identification of effective dose and dosing schedule of new targeted anti-cancer agents during early phase trials. Mathematical modeling of CA-125 kinetics in ROC patients enables understanding of the time-change components during chemotherapy. The modeled CA-125 elimination rate KELIM, potentially assessable in routine, may have promising predictive value regarding PFS.

APPENDIX 8 – ADDITIONAL SAFETY INFORMATION

Definitions

Term	Definition
**Adverse event (AE)**	Any untoward medical occurrence in a patient or clinical investigation subject, administered a pharmaceutical product and which does not necessarily have to have a causal relationship with this treatment.<br>An AE can therefore be any unfavorable and unintended sign (including an abnormal laboratory finding, for example), symptom, or disease temporally associated with the use of a medicinal product, whether or not considered related to the medicinal product.
**Adverse Reaction (AR)**	Any noxious and unintended responses to a medicinal product related to any dose.
**Serious AE (SAE) or**<br>**Serious AR (SAR)**	Results in death *(NOTE: death is an outcome, not an event)*<br>Is life-threatening *(NOTE: the term "life-threatening" refers to an event in which the patient was at risk of death at the time of the event; it does not refer to an event which that hypothetically might have caused a death if it were more severe.)*<br>Requires in-patient hospitalization or prolongation of existing hospitalization<br>Results in persistent or significant disability/incapacity or<br>Is a congenital anomaly/birth defect<br>Is an important medical event (an event that jeopardizes the patient or may require intervention to prevent one of the other outcomes listed above
**Expected SAE**	Serious adverse event is an event that is mentioned in the Investigator Brochure or Summary of Product Characteristics latest version for product that granted marketing authorization, even if it is not in the same studied population.
**Suspected Unexpected Serious Adverse Reaction (SUSAR)**	Serious adverse reaction, the nature, or severity of which is not consistent with the applicable product information.
**Adverse Events of Special Interest (AESI)**	Adverse events that have been identified as AESI from previous study drug clinical data and require prompt SAE reporting to Pharmacovigilance if the events can be considered non-serious according to the usual regulatory criteria as they may be subject to expedited submission to regulatory authorities. The AESI requiring expedited report are the following:
**New fact**	All new safety data which may lead to the reevaluation of the risk-benefit balance of the research or of the experimental product, or that could be sufficient to consider modifications in the experimental product administration, in the conduct of the trial, or research documents

Further Guidance on the definition of serious adverse event (SAE)

Life threatening

'Life-threatening' means that the subject was at immediate risk of death from the AE as it occurred or it is suspected that use or continued use of the product would result in the subject's death. 'Life-threatening' does not mean that had an AE occurred in a more severe form it might have caused death (e.g., hepatitis that resolved without hepatic failure).

Hospitalization

Outpatient treatment in an emergency room is not in itself a serious AE, although the reasons for it may be (e.g. bronchospasm, laryngeal edema). Hospital admissions and/or surgical operations planned before or during a study are not considered AEs if the illness or disease existed before the subject was enrolled in the study, provided that it did not deteriorate in an unexpected way during the study.

Important medical event or medical intervention

Medical and scientific judgment should be exercised in deciding whether a case is serious in situations where important medical events may not be immediately life threatening or result in death, hospitalization, disability or incapacity but may jeopardize the subject or may require medical intervention to prevent one or more outcomes listed in the definition of serious.
These should usually be considered as serious.
Simply stopping the suspect drug does not mean that it is an important medical event; medical judgment must be used.
Examples of such events are:

- Angioedema not severe enough to require intubation but requiring iv hydrocortisone treatment
- Hepatotoxicity caused by paracetamol (acetaminophen) overdose requiring treatment with N-acetylcysteine
- Intensive treatment in an emergency room or at home for allergic bronchospasm
- Blood dyscrasias (e.g., neutropenia or anaemia requiring blood transfusion, etc) or convulsions that do not result in hospitalization
- Development of drug dependency or drug abuse.

A guide to interpreting the causality question

The following factors should be considered when deciding if there is a "reasonable possibility" that an AE may have been caused by the drug.

- Time Course. Exposure to suspect drug. Has the subject actually received the suspect drug? Did the AE occur in a reasonable temporal relationship to the administration of the suspect drug?
- Consistency with known drug profile. Was the AE consistent with the previous knowledge of the suspect drug (pharmacology and toxicology) or drugs of the same pharmacological class? OR could the AE be anticipated from its pharmacological properties?
- Re challenge experience. Did the AE resolve or improve on stopping or reducing the dose of the suspect drug?
- No alternative cause. The AE cannot be reasonably explained by another etiology such as the underlying disease, other drugs, other host or environmental factors.
- Re challenge experience. Did the AE reoccur if the suspected drug was reintroduced after having been stopped? AstraZeneca would not normally recommend or support a re challenge.

- Laboratory tests. A specific laboratory investigation (if performed) has confirmed the relationship?

A “reasonable possibility” could be considered to exist for an AE where one or more of these factors exist.

In contrast, there would not be a “reasonable possibility” of causality if none of the above criteria apply or where there is evidence of exposure and a reasonable time course but any dechallenge (if performed) is negative or ambiguous or there is another more likely cause of the AE.

In difficult cases, other factors could be considered such as:

- Is this a recognized feature of overdose of the drug?
- Is there a known mechanism?

Ambiguous cases should be considered as being a “reasonable possibility” of a causal relationship unless further evidence becomes available to refute this. Causal relationship in cases where the disease under study has deteriorated due to lack of effect should be classified.

SUPPLEMENTARY NOTE 1 - BOLD STATISTICAL ANALYSIS PLAN

A GINECO PHASE II TRIAL ASSESSING THE SAFETY AND EFFICACY OF THE BEVACIZUMAB (FKB238), OLAPARIB AND DURVALUMAB (MEDI 4736) COMBINATION IN PATIENTS WITH ADVANCED EPITHELIAL OVARIAN CANCER IN RELAPSE

BOLD

(Bevacizumab (FKB238), Olaparib, and Durvalumab (MEDI 4736) in relapsing high grade carcinoma of the ovary)

Statistical Analysis Plan

Version: 4

Date version: 2021-04-12

Written by: Dr Catherine MERCIER (PhD) and Carole Langlois-Jacques

Approved by:

Institut de Cancérologie des Hospices Civils de Lyon : Dr Julien Péron (MD, PhD), Pr Gilles Freyer

ARCAGY-GINECO : Laure Jerber

Biostatisticians:

Catherine Mercier and Carole Langlois-Jacques

Hospices Civils de Lyon

Pôle de santé publique

Service de Biostatistique et Bioinformatique

162 avenue Lacassagne

69424 Lyon Cedex 03

FRANCE

Table of contents

List of abbreviations and definition of terms **4**
1 Study objectives **5**
1.1 Primary objective 5
1.2 Secondary objectives 5
2 Experimental Design **5**
2.1 Trial design 5
2.2 Randomization 5
2.3 Patient screening and study schedule 5
3 Study Evaluation Criteria **8**
3.1 Primary efficacy endpoint 8
3.2 Secondary efficacy endpoints 8
3.3 Safety endpoints 8
• Adverse events (AE) **8**
4 Statistical methods **8**
4.1 Sample size determination 8
4.2 Interim analysis 9
4.3 General issues for statistical analyses 9
4.3.1 Statistical software 9
4.3.2 Presentation of results 9
4.3.3 Usual statistical tests 10
4.3.4 Date and duration conventions 10
4.3.5 Missing data 10
4.4 Definition of population 10
4.4.1 Selection criteria of the Study Population 10
4.4.1.1 Inclusion criteria 10
4.4.1.2 Exclusion criteria 13
4.4.1.3 Protocol violation 16
4.4.2 Analysis populations 16
4.4.2.1 Intent-to-Treat Population 16
4.4.2.2 Per Protocol Population 16
4.4.2.3 Population analysis 16
4.5 Definition of outcomes 17

4.5.1 Primary outcome measure ... *17*
4.5.2 Secondary outcome measures ... *18*
4.5.3 Derived and computed variables ... *18*
4.6 Statistical Analyses ... 20
4.6.1 Primary efficacy Outcome ... *20*
4.6.2 Secondary efficacy outcomes ... *20*
4.6.3 Secondary outcomes for toxicity and safety ... *21*
4.6.4 Trial Population description ... *22*
4.6.5 Adherence and protocol deviations ... *22*
4.6.6 Baseline and at diagnosis patient characteristics ... *23*
4.6.7 Exposition to treatments: ... *23*
5 References ... 23
6 Appendix ... 24
6.1 Data variables ... 24
6.2 Progression determination algorithm ... 26
6.3 Clinical – radiological primary outcome ... 30
6.4 Not Evaluable patients ... 30
6.5 Overall response ... 31

List of abbreviations and definition of terms

List of abbreviation or special term	Explanation
AE	Adverse Event
AESI	Adverse Event of Specific Interest
Baseline	Refers to the most recent assessment of any variable prior to dosing with study treatment
CA-125	Cancer Antigen – 125
CI	Confidence interval
CR	Complete response
CT	Computed Tomography
CTC / CTCAE	Common Terminology Criteria for Adverse Event
IDMC	Independent Data Monitoring Committee
ITT	Intent To Treat
Ir RECIST	Immune-related Response Criteria Derived from RECIST v.1.1
MedDRA	Medical Dictionary for Regulatory Activities
MRI	Magnetic Resonance Imaging
NE	Not Evaluable
NED	No Evidence of Disease
NTL	Non-Target Lesions
OC	Ovarian Cancer
ORR	Objective Response Rates
OS	Overall Survival
PD	Progressive Disease
PFS	Progression Free Survival
PI	Principal Investigator
PP	Per Protocol
PR	Partial response
PRR	Platinum resistant relapse
PSR	Platinum sensitive relapse
QoL	Quality of Life
SAP	Statistical Analysis Plan
SD	Stable Disease

1 Study objectives

1.1 Primary objective

Efficacy and tolerance of the following combination:

- Durvalumab 1.12 g IV Q3W
- Bevacizumab (FKB238) 15 mg/kg Day 1 Q3W
- Olaparib 300 mg twice daily po (150 mg and 100mg tablets), continuously.

The primary objective is the rate of clinical and radiological non-progressive disease, as assessed by immune-related response criteria (irRC) (Wolchok et al. 2009) in two cohorts, Platinum resistant relapse (PRR) or Platinum sensitive relapse (PSR):

- At 3 months in the PRR cohort
- At 6 months in the PSR cohort

1.2 Secondary objectives

The secondary objectives are

- CA 125 decline as expressed by the KELIM parameter
- Progression free survival (PFS)
- Overall survival (OS)
- Tumor response
- Toxicity as assessed by CTCAE V.5.0 scale

2 Experimental Design

2.1 Trial design

This is a multi-centre, open, non-randomized Phase II trial assessing the safety of the Bevacizumab, Olaparib and Durvalumab combination in patients with advanced epithelial ovarian cancer in relapse (PRR or PSR).

2.2 Randomization

No randomization is performed (mono arm cohorts).

2.3 Patient screening and study schedule

GINECO

	Visit									
**Study period**	**Inclusion**	**C1**	**C1**	**C1**	**C2**	**Cn**	**Assessment visit**	**End of treatment (EoT) visit**	**Safety follow-up visit**	**Follow up after progression or toxicity**
**Timelines**		**D1**	**D8**	**D15**	**D1**	**D1**	**Each 6 weeks ±1 week (2 cycles) until progression**	**Just after last TTT administration**	**30 days ±7 after last TTT administration**	**Each 3 months during 12 months**
**Informed consent**[1]	☑									
**Inclusion / Exclusion criteria**	☑									
**Medical history**	☑									
**Physical examination, Vital signs, Performance status**	☑	☑	☑	☑	☑	☑	☑	☑	☑	☑
**Haematology**[a]	☑[5]	☑	☑	☑	☑	☑	☑	☑	☑	
**Coagulation**[b]	☑	☑								
**Biochemistry**[c]	☑[5]	☑	☑	☑	☑	☑	☑	☑	☑	
**Hormonology**[d]	☑[5]	☑	☑	☑	☑	☑	☑	☑	☑	
**Urinalysis (dipstick)**	☑[5]	☑	☑	☑	☑	☑	☑	☑	☑	
**Pregnancy test**[2]	☑[5]						☑		☑	☑
**ECG**	☑								☑	
**CT scan (Thoracic-Abdominal-Pelvic)**[3]	☑[4]						☑		☑	
**CA-125**	☑[5]						☑	☑	☑	
**Study treatment : Bevacizumab, 15mg/Kg IV**[9]		☑			☑	☑				
**Study treatment : Olaparib, 300mg bid po**[10]				☑						
**Study treatment : Durvalumab, 1.12g IV**[9]		☑			☑	☑				
**Adverse events/concomitant treatments**	☑	☑	☑	☑	☑	☑	☑	☑	☑	☑
**Translational Research: biopsy**	☑[6]							☑[7]		
**Translational Research: blood sample**	☑				☑	☑[8]				

1: Informed consent has to be signed any time before any study procedure.

2: Except for women without female genital tract.

3: MRI of the abdomen and pelvis and a chest X-ray can be performed instead of CT scan. The same imaging modality (CT or MRI) has to be used throughout the trial. If an unscheduled CT-scan or MRI of the abdomen and pelvis has been performed within six weeks of a scheduled image, it does not need to be repeated.

4: Imaging has to be done within 28 days of first dose

5:Test to be repeated within 7 days prior the treatment start.

6: Within 3 months before inclusion and performed after the last previous line chemotherapy administration

7: Tumor biopsy at relapse optional

8: blood sample at C3D1

9: Every 3 weeks (D21Cx = D1Cx+1)

10: Continuously

a:Hemoglobin, platelets, white blood cell, neutrophil, lymphcyts, absolute neutrophils count

b:Coagulation: aPTT, INR

c: Biochemistry: Albumin, Total bilirubin, Lactic dehydrogenase [LDH], Aspartate [AST] and Alanine transaminase [ALT], Alkaline Phosphatase [ALP], Sodium, Potassium, Calcium, Gamma Glutamyl transferase (GGT), Creatinin, Creatinin clearance, Glucose

d: Thyroid stimulating hormone (TSH), Free thyroxine (FT4)

3 Study Evaluation Criteria

3.1 Primary efficacy endpoint

The primary outcome measure is the rate of clinical and radiological non-progressive disease, as assessed by immune-related response criteria (irRECIST) (Wolchok et al. 2009):

- At 3 months in the PRR cohort
- At 6 months in the PSR cohort

3.2 Secondary efficacy endpoints

- CA 125 decline as expressed by the KELIM parameter
- Progression free survival (PFS)
- Overall survival (OS)
- Tumor response
- Toxicity as assessed by CTCAE V.5.0 scale

3.3 Safety endpoints

- Adverse events (AE)
- Serious adverse events (SAE)
- Adverse event of special interest (AESI)

4 Statistical methods

4.1 Sample size determination

The primary objective is the rate of clinical and radiological non-progressive disease, as assessed by immune-related response criteria (irRECIST) (Wolchok et al. 2009):

- At 3 months in the PRR cohort
- At 6 months in the PSR cohort

The sample size was calculated independently in the two cohorts. The study is based on two hypotheses for each cohort.

PRR cohort:

- The null hypothesis (p0) is 50% or lower. A rate of non-progressive disease at 3 months of 50% was considered undesirable (p0 = 50%) compared with historical control in this patient population
- The alternative hypothesis is a rate of non-progressive disease at 3 months higher than 50%.
- The positive hypothesis (p1) used for sample size calculation is 75%. A non-progressive disease rate of 75% was considered to warrant further investigations

- Sample size and cut-offs were calculated based on the exact binomial distribution. Using a one stage design and the exact binomial distribution [A'Hern, 2001], twenty three patients must be included in trial (**n=23**). This design yields a one-sided type-1 error rate of α=5% and a power of 80% when the true non progressive disease rate is 75%.

The total number of evaluable patients in PRR cohort will be 23. Non evaluable patients will be replaced.

PSR cohort:

- The null hypothesis (p0) is 65%. A rate of non-progressive disease at 6 months of 65% was considered undesirable
- The alternative hypothesis is a rate of non-progressive disease at 6 months higher than 65%.

- The positive hypothesis (p1) is 84%. A non-progressive disease rate of 84% was considered to warrant further investigations.
- We again based sample size and cut-offs calculation on the exact binomial distribution. Using a one stage design and the exact binomial distribution, forty patients will be included in the trial **(n=40)**. This design yields a one-sided type-1 error rate of α=3% and a power of at least 82% when the true non progressive disease rate is 84%.

  The total number of evaluable patients in PSR cohort will be 40. Non evaluable patients will be replaced.

4.2 Interim analysis

No interim analysis was planned for efficacy. An interim safety analysis was planned and performed for both cohorts (safety report in 2020/04/25).

4.3 General issues for statistical analyses

4.3.1 Statistical software

All statistical analyses will be performed using SAS® Software version 9.4 in a Windows environment, and R software version 4.0.2.

4.3.2 Presentation of results

The quantitative variable will be described by the following parameters: Number of patients, Number of missing value, Mean, Standard deviation (SD), Median, First and third quartiles (Q1 and Q3), Minimum and maximum.

The qualitative variable will be described by the following parameters: Number of patient, Number of missing value, Frequency and percentage of each modality (missing values will not be included in the denominator used for frequency computation).

4.3.3 Usual statistical tests

No comparisons are planned between the two cohorts.

Within each cohort, the significance threshold of 5% will be adopted for all hypotheses testing unless otherwise specified. If necessary, adjustment for multiple tests can be adopted.

Confidence interval (CI) provided will be a bilateral 95% confidence interval unless otherwise specified.

4.3.4 Date and duration conventions

The time since inclusion will be defined as the time elapsed since the day of inclusion visit, the day of inclusion being considered as day 1.

Thereby, calculation rule of duration can be defined as, for example, the computation of the time elapsed between death and inclusion by: day of death – day of inclusion +1.

Convention rules for computing the conversion of the number of days to the number of months or years are: 1 month = 30.5 days ; 1 year = 365.25 days.

4.3.5 Missing data

All missing/invalidated data on efficacy endpoints will be reviewed by the Coordinator Investigator and the statistician before their potential consideration in the analysis.

4.4 Definition of population

4.4.1 Selection criteria of the Study Population

4.4.1.1 Inclusion criteria

Patients will be eligible for study participation if they respect all the following criteria:

I-1 Female Patient must be ≥18 years of age.

I-2 Signed informed consent and ability to comply with treatment and follow-up.

I-3 Patient with :

Ovarian cancer, primary peritoneal cancer and/or fallopian-tube cancer, histologically confirmed (based on local histopathological findings): high grade serous or high grade endometrioid or other high grade epithelial non mucinous ovarian tumor.

I-4 Patient who has completed at least one line of platinum-taxane chemotherapy, and presents with platinum resistant relapse (resistant disease defined by a tumor progression less than six months after the last dose of platinum) [Note: the patient may have received one or even more line of platinum based chemotherapy].

I-5 Patient who didn't receive any of the tested drugs, or previously received either bevacizumab or olaparib BUT NOT the combination of both drugs.

I-6 At least one measurable or evaluable lesion that can be accurately assessed at baseline by computed tomography (CT) (or magnetic resonance imaging [MRI] where CT is contraindicated) and is suitable for repeated assessment as per irRECIST. The baseline scan must be obtained within 28 days of first dose.

I-7 Availability of a pre-treatment tumor sample (archival FFPE block or fresh biopsy if feasible) lasting of less than 3 months before inclusion into the study and performed AFTER the last chemotherapy administration.

I-8 Patient not amenable to cytoreductive surgery at the time of relapse (surgery is not allowed during the protocole treatment).

I-9 Patient must have normal organ and bone marrow function:

a. Hemoglobin ≥ 9.0 g/dL. (Transfusions is not allowed within 28 days before randomization)

b. Absolute neutrophil count (ANC) ≥ 1.5 x 10^9/L.

c. Platelet count ≥ 100 x 10^9/L. (Platelet transfusion or G-CSF administration is not allowed within 28 days before randomization)

d. Total bilirubin ≤ 1.5 x institutional upper limit of normal (ULN).

e. Aspartate aminotransferase / Serum Glutamic Oxaloacetic Transaminase (ASAT/SGOT)) and Alanine aminotransferase / Serum Glutamic Pyruvate Transaminase (ALAT/SGPT)) ≤ 2.5 x ULN, unless liver metastases are present in which case they must be ≤ 5 x ULN.

f. Creatinine clearance ≥ 60 mL/min by Cockcroft and Gault formula.

g. Patient not receiving anticoagulant medication who has an International Normalized Ratio (INR) ≤ 1.5 and an Activated ProThrombin Time (aPTT) ≤ 1.5 x ULN. The use of full-dose oral or parenteral anticoagulants is permitted as long as the INR or APTT is within therapeutic limits (according to site medical standard). If the patient is on oral anticoagulants, dose has to be stable for at least two weeks at the time of inclusion.

h. Urine dipstick for proteinuria < 2+. If urine dipstick is ≥ 2+, 24-hour urine must demonstrate < 1 g of protein in 24 hours.

i. Normal blood pressure or adequately treated and controlled hypertension (systolic BP ≤ 150 mmHg and/or diastolic BP ≤ 90 mmHg).

I-10 Expectancy of at least 12 weeks

I-11 Eastern Cooperative Oncology Group (ECOG) performance status 0-1.

I-12 Postmenopausal or evidence of non-childbearing status for women of childbearing potential prior to the first dose of study treatment (see protocol 2).

I-13 As this study will include patients in France, a subject will be eligible for inclusion in this study only if either affiliated to, or a beneficiary of, a social category.

Patients with platinum sensitive relapse

I-1 Female Patient must be ≥18 years of age.

I-2 Signed informed consent and ability to comply with treatment and follow-up.

I-3 Patient with :

Ovarian cancer, primary peritoneal cancer and/or fallopian-tube cancer, histologically confirmed (based on local histopathological findings): high grade serous or high grade endometrioid or other high grade epithelial non mucinous ovarian tumor.

I-4 Patient who is in platinum-sensitive relapse, whatever the line of chemotherapy given at relapse [Note: any chemotherapy previously administered must have contained a platinum compound]. The platinum sensitive relapse is defined by a tumor progression occurring more than six months after the last dose of platinum chemotherapy.

I-5 Patient who didn't receive any of the tested drugs, or previously received either bevacizumab or olaparib BUT NOT the combination of both drugs.

I-6 At least one measurable or evaluable lesion that can be accurately assessed at baseline by computed tomography (CT) (or magnetic resonance imaging [MRI] where CT is contraindicated) and is suitable for repeated assessment as per irRECIST. The baseline scan must be obtained within 28 days of first dose.

I-7 Availability of a pre-treatment tumor sample (archival FFPE block or fresh biopsy if feasible) lasting of less than 3 months before inclusion into the study and performed AFTER the last chemotherapy administration.

I-8 Patient not amenable to cytoreductive surgery at the time of relapse (surgery is not allowed during the protocole treatment).

I-9 Patient must have normal organ and bone marrow function:

a. Hemoglobin ≥ 10.0 g/dL. (Transfusions is not allowed within 28 before randomization)

b. Absolute neutrophil count (ANC) ≥ 1.5 x 10^9/L.

c. Platelet count ≥ 100 x 10^9/L. (Platelet transfusion or G-CSF administration is not allowed within 28 days before randomization)

d. Total bilirubin ≤ 1.5 x institutional upper limit of normal (ULN).

e. Aspartate aminotransferase / Serum Glutamic Oxaloacetic Transaminase (ASAT/SGOT)) and Alanine aminotransferase / Serum Glutamic Pyruvate Transaminase (ALAT/SGPT)) ≤ 2.5 x ULN, unless liver metastases are present in which case they must be ≤ 5 x ULN.

f. Creatinine clearance ≥ 60 mL/min by Cockcroft and Gault formula.

g. Patient not receiving anticoagulant medication who has an International Normalized Ratio (INR) ≤ 1.5 and an Activated ProThrombin Time (aPTT) ≤ 1.5 x ULN. The use of full-dose oral or parenteral anticoagulants is permitted as long as the INR or APTT is within therapeutic limits (according to site medical standard). If the patient is on oral anticoagulants, dose has to be stable for at least two weeks at the time of incusion.

h. Urine dipstick for proteinuria < 2+. If urine dipstick is ≥ 2+, 24-hour urine must demonstrate < 1 g of protein in 24 hours.

i. Normal blood pressure or adequately treated and controlled hypertension (systolic BP ≤ 150 mmHg and/or diastolic BP ≤ 90 mmHg).

I-10 Expectancy of at least 12 weeks

I-11 Eastern Cooperative Oncology Group (ECOG) performance status 0-1.

I-12 Postmenopausal or evidence of non-childbearing status for women of childbearing potential prior to the first dose of study treatment.

I-13 As this study will include patients in France, a subject will be eligible for inclusion in this study only if either affiliated to, or a beneficiary of, a social category.

4.4.1.2 Exclusion criteria

Patient must not enter the study if any of the following exclusion criteria are fulfilled

E-1 Non-epithelial origin of the tumor (i.e. germ cell tumor).

E-2 Ovarian tumors of low malignant potential (e.g. borderline tumors), or mucinous carcinoma.

E-3 Carcinosarcoma (Mixed Mullerian Tumor)

E-4 Patient with synchronous primary endometrial cancer unless both of the following criteria are met:

- Stage < II,
- Less than 60 years old at the time of diagnosis of endometrial cancer with stage IA or IB grade 1 or 2, or stage IA grade III endometrial carcinoma,OR ≥ 60 years old at the time of diagnosis of endometrial cancer with stage IA grade 1 or 2 endometrioid adenocarcinoma.

Patient with serous or clear cell adenocarcinoma or carcinosarcoma of the endometrium is not eligible.

E-5 Other malignancy within the last 5 years except: adequately treated non-melanoma skin cancer, curatively treated in situ cancer of the cervix, ductal carcinoma in situ (DCIS). Patient with a history of localized malignancy diagnosed over 5 years ago may be eligible provided she completed her adjuvant systemic therapy and remains free of recurrent or metastatic disease. Patient with history of primary triple negative breast cancer may be eligible provided she completed her definitive anticancer treatment more than 3 years ago and she remains breast cancer disease free prior to start of study treatment.

E-6 Patient with myelodysplastic syndrome/acute myeloid leukemia history.

E-7 Current or prior use of immunosuppressive medication within 14 days (use 28 days if combining durvalumab with a novel agent) before the first dose of durvalumab, with the exceptions of intranasal and inhaled corticosteroids or systemic corticosteroids at physiological doses, which are not to exceed 10 mg/day of prednisone, or an equivalent corticosteroid. The following are exceptions to this criterion:

- Intranasal, inhaled, topical steroids, or local steroid injections (e.g., intra articular injection)
- Systemic corticosteroids at physiologic doses not to exceed <<10 mg/day>> of prednisone or its equivalent
- Steroids as premedication for hypersensitivity reactions (e.g., CT scan premedication)

E-8 Any unresolved toxicity NCI CTCAE Grade ≥2 from previous anticancer therapy with the exception of alopecia, vitiligo, and the laboratory values defined in the inclusion criteria

- Patients with Grade ≥2 neuropathy will be evaluated on a case-by-case basis after consultation with the Study Physician.
- Patients with irreversible toxicity not reasonably expected to be exacerbated by treatment with durvalumab may be included only after consultation with the Study Physician.

E-9 Active or prior documented autoimmune or inflammatory disorders (including inflammatory bowel disease [e.g., colitis or Crohn's disease], diverticulitis [with the exception of diverticulosis], systemic lupus erythematosus, Sarcoidosis syndrome, or Wegener syndrome [granulomatosis with polyangiitis, Graves' disease, rheumatoid arthritis, hypophysitis, uveitis, etc]). The following are exceptions to this criterion:

- Patients with vitiligo or alopecia
- Patients with hypothyroidism (e.g., following Hashimoto syndrome) stable on hormone replacement
- Any chronic skin condition that does not require systemic therapy
- Patients without active disease in the last 5 years may be included but only after consultation with the study physician
- Uncontrolled intercurrent illness, including but not limited to, ongoing or active infection, symptomatic congestive heart failure, uncontrolled hypertension, unstable angina pectoris, cardiac arrhythmia, interstitial lung disease, serious chronic gastrointestinal conditions associated with diarrhea, or psychiatric illness/social situations that would limit compliance with study requirement, substantially increase risk of incurring AEs or compromise the ability of the patient to give written informed consent
- History of active primary immunodeficiency
- Active infection including tuberculosis (clinical evaluation that includes clinical history, physical examination and radiographic findings, and TB testing in line with local practice), hepatitis B (known positive HBV surface antigen (HBsAg) result), hepatitis C, or human immunodeficiency virus (positive HIV 1/2 antibodies). Patients with a past or resolved HBV infection (defined as the presence of hepatitis B core antibody [anti-HBc] and absence of HBsAg) are eligible. Patients positive for hepatitis C (HCV) antibody are eligible only if polymerase chain reaction is negative for HCV RNA.

E-10 Patient receiving radiotherapy within 6 weeks prior to study treatment.

E-11 Major surgery within 4 weeks of starting study treatment and patient must have recovered from any effects of any major surgery.

E-12 Previous allogenic bone marrow transplant.

E-13 Any previous treatment with Anti PD(L)-1 immunotherapy, including durvalumab

E-14 Any previous treatment with a PARP inhibitor in combination with an anti-VEGF (previous treatment with PARP inhibitor alone or anti-VEGF alone is allowed).

E-15 Past medical history of interstitial lung disease, drug-induced pneumonitis, radiation pneumonitis that required steroid treatment, or any evidence of clinically active interstitial lung disease

E-16 Administration of other simultaneous chemotherapy drugs, any other anticancer therapy or anti-neoplastic hormonal therapy, or simultaneous radiotherapy during the trial treatment period (hormonal replacement therapy is permitted as are steroidal antiemetics).

E-17 Current or recent (within 10 days prior to inclusion) chronic use of aspirin > 325 mg/day.

E-18 Concomitant use of known potent CYP3A4 inhibitors such as ketoconazole, itraconazole, ritonavir, indinavir, saquinavir, telithromycin, clarithromycin and nelfinavir. The required washout period prior to starting study treatment is 2 weeks.

E-19 Concomitant use of known strong (e.g. phenobarbital, enzalutamide, phenytoin, rifampicin, rifabutin, rifapentine, carbamazepine, nevirapine and St John's Wort) or moderate CYP3A inducers (e.g. bosentan, efavirenz, modafinil). The required washout period prior to starting study treatment is 5 weeks for enzalutamide or phenobarbital and 3 weeks for other agents. Patients should stop using herbal remedies 7 days prior to the first dose of study medication and for the duration of the trial.

E-20 Prior history of hypertensive crisis (CTCAE grade 4) or hypertensive encephalopathy.

E-21 Clinically significant (e.g. active) cardiovascular disease, Previous Cerebro-Vascular Accident (CVA), Transient Ischemic Attack (TIA) or Sub- Arachnoids Hemorrhage (SAH) within 6 months prior to inclusion.

E-22 History Clinically significant (e.g. active) cardiovascular disease, including:

- Myocardial infarction or unstable angina within ≤ 6 months of inclusion,
- New York Heart Association (NYHA) ≥ grade 2 congestive heart failure (CHF),
- Poorly controlled cardiac arrhythmia despite medication (patient with rate controlled atrial fibrillation are eligible), or any clinically significant abnormal finding on resting ECG,
- Peripheral vascular disease grade ≥ 3 (e.g. symptomatic and interfering with activities of daily living [ADL] requiring repair or revision) or evidence of hemorrhagic disorders within 6 months prior to treatment administration.

E-23 Evidence of bleeding diathesis or significant coagulopathy (in the absence of coagulation).

E-24 History or clinical suspicion of brain metastases or spinal cord compression. CT/MRI of the brain is mandatory (within 4 weeks prior to inclusion) in case of suspected brain metastases. Spinal MRI is mandatory (within 4 weeks prior to inclusion) in case of suspected spinal cord compression.

E-25 Significant traumatic injury during 4 weeks prior to inclusion.

E-26 Non-healing wound, active ulcer or bone fracture. Patient with granulating incisions healing by secondary intention with no evidence of facial dehiscence or infection is eligible but require 3 weekly wound examinations.

E-27 History of VEGF therapy related abdominal fistula or gastrointestinal perforation or active gastrointestinal bleeding within 6 months prior to the first study treatment.

E-28 Current, clinically relevant bowel obstruction, including sub-occlusive and occlusive disease.

E-29 Patient with evidence of abdominal free air not explained by paracentesis or recent surgical procedure.

E-30 Evidence of any other disease, metabolic dysfunction, physical examination finding or laboratory finding giving reasonable suspicion of a disease or condition that contraindicates the use of an investigational drug or puts the patient at high risk for treatment related complications.

E-31 Pregnant or lactating women.

E-32 Participation in another clinical study with an investigational product during her chemotherapy course immediately prior to inclusion.

E-33 Patient unable to swallow orally administered medication and patient with gastrointestinal disorders likely to interfere with absorption of the study medication.

E-34 Patient with a known hypersensitivity to olaparib, durvalumab or bevacizumab or any of the recipients of those products.

Immunocompromised patient, e.g., with known active hepatitis (i.e. Hepatitis B or C) due to risk of transmitting the infection through blood or other body fluids or patient who is known to be serologically positive for human immunodeficiency virus (HIV).

4.4.1.3 Protocol violation

2 major protocol violations will be considered concerning inclusion criteria:

- Inclusion criteria : Major deviation when a creatinine clairance less than < 45ml/min
- Inclusion criteria : Major deviation when a patient was included with a Current or prior use of immunosuppressive medication within 14 days (use 28 days if combining durvalumab with a novel agent) before the first dose of durvalumab

4.4.2 Analysis populations

4.4.2.1 Intent-to-Treat Population

The Intent-to-treat (ITT) population is defined as all patients included in the cohort considered, regardless of whether they actually received treatment.

4.4.2.2 Per Protocol Population

The Per Protocol (PP) population is a subgroup of the ITT population containing all patients who do not have any major protocol violation and received study treatment Olaparib, bevacizumab and durvalumab at least once. Major protocol violations will be defined in the Statistical Analysis Plan (SAP) before the data lock.

4.4.2.3 Population analysis

Primary outcome of efficacy analyses

Population:

- Intent-to-treat population (primary analysis)
- per-protocol population (secondary analysis)

Secondary outcomes of efficacy analyses: CA125 decline, Progression free survival, Overall survival, Tumor response.

Population:

- Intent-to-treat population

Safety analysis

The safety data will be analyzed on the safety analysis set including all included patients having received at least one dose of study treatment.

4.5 Definition of outcomes

4.5.1 Primary outcome measure

The primary outcome measure is the rate of clinical and radiological non-progressive disease, as assessed by immune-related response criteria (irRECIST) (Wolchok et al. 2009):

- At 3 months in the PRR cohort
- At 6 months in the PSR cohort

The rate of clinical and radiological non-progressive disease is based on the best overall response to the treatment according to the irRECIST criteria.

As recommended for trials where the response rate is the primary endpoint, the best tumor response needs to be confirmed according to RECIST and all responses will be reviewed by an expert(s) independent of the study at the study's conclusion.

- irRECIST criteria will be used as recorded in the CRF to compute the overall tumor response,
- RECIST criteria will be used in addition to compute separately the overall tumor response (details in 2.5.3 Derived and computed variables).

The differences between RECIST and irRECIST criteria occur in cases of progression (Tazdait et al 2017). Unlike the RECIST 1.1 guidelines:

- irRECIST require a confirmatory CT scan at 4-8 weeks , if a progression is confirmed, the date of progression is the date of the first scan
- Death or immunotherapy discontinuation due to clinical progression is considered as confirmation of progression.
- For appearance of one or more new lesion(s), new measurable lesions are added to the sum of target lesions.
- To confirm progression, the irRECIST guidelines require an increase of target, non-target or new lesions compared with nadir in the subsequent CT with the same cut-off than RECIST 1.1 guidelines.

The rate of clinical and radiological non-progressive disease will be analyzed according to both set of criteria (RECIST an irRECIST).

All data on the primary endpoint will be used for statistical analysis. Reasons for treatment stopping will be documented. If the decision (of the patient or the investigator) is related to inefficacy or treatment safety it will be considered as a failure.

4.5.2 Secondary outcome measures

- CA 125 decline as expressed by the KELIM parameter
  CA-125 ELIMination Rate Constant K (KELIM) Is a Marker of Chemosensitivity in Patients with Ovarian Cancer. It will not be registered in the database and it will be calculated by Pr B.You and sent to the statistical team after the database lock. .
- Progression free survival (PFS) will be calculated between the date of inclusion and the date of progression or death or censored at the date of the first administration of the next systemic therapy or at their last follow-up visit (details in 4.6.2).
- Overall survival (OS) will be calculated between the date of inclusion and the date of death or censored at the date of the last follow-up visit (after 1 year for the two cohorts) or at the date of the administrative inquiry (details in 4.6.2).
- Tumor response (RECIST v1.1 and irRECIST)
- Toxicity as assessed by CTCAE V.5.0 scale

4.5.3 Derived and computed variables

The overall tumor response for each patient at certain time point will depend on the findings of both target and no-target lesions, and also will include the occurrence of any new lesion. The best overall response is the best response recorded from the start of the treatment until disease progression/recurrence.

Tumor response (RECIST v1.1)

- **Target lesions**

• Complete Response (CR) –all target lesions have disappeared during the treatment. Lymph nodes selected must return to normal size (<10mm).

• Partial Response (PR) –at least 30% decrease from baseline sum of the longest diameter.

• Stable Disease (SD) –no significant decrease or increase in the size of target lesions.

• Progressive Disease (PD) – >=20% increase over the smallest sum of the longest diameter and with at least 5mm increase, or appearance of any new lesion.

- **Non-target lesions**

• Complete Response (CR): complete disappearance of all non-target lesions and normalization of lymph node.

• Stable Disease (SD): persistence of one or more non-target lesions.

• Progressive Disease (PD): Appearance of any new lesion and/or unequivocal progression of existing non-target lesions.

Time point overall response The below table is the summary of overall response at certain time point per RECIST 1.1 guidelines.

Target Lesion	Non-Target	New Lesion(s)	Overall Response

**CR**	**CR**	**No**	**CR**
**CR or PR**	**SD or NE**	**No**	**PR**
**SD**	**No PD**	**No**	**SD**
**NE**	**No PD**	**No**	**NE**
**PD**	**Any**	**Yes or No**	**PD**
**Any**	**PD**	**Yes or No**	**PD**
**Any**	**Any**	**Yes**	**PD**

NE – Not Evaluable

Best overall response

The best overall response is the best response recorded from the start of the treatment until disease progression/recurrence (taking as reference for PD the smallest measurements recorded since the treatment started). Note, tumor assessments performed after initiation of new anticancer treatment will be excluded from evaluating the best overall response. In general, the patient's best response assignment will depend on the achievement of both measurement and confirmation criteria.

Exposition to treatment

By cycle:

- Durvalumab:
  - administred dose by cycle (variable DURVADOSE in the database).
  - theoric dose= 1120mg per cycle.
  - If administred dose is missing : 0 if not administred, theoric dose if administred.

- Bevacizumab (Fkb238)
  - administred dose by cycle (variable BEVADOSE in the database).
  - theoric dose= 15mg/kg per cycle according to weight at the same visit.
  - If the weight is missing : weight at screening and if missing mean weight for the patient (in table clin)
  - If administred dose is missing : 0 if not administred, theoric dose if administred.

- Olaparib:
  - administred dose by cycle (variable OLATDOS in the database).
  - if discontinuation sum of administred doses in the cycle.
  - theoric dose= 600 mg/day
  - number of days : 21 (theoric days) or (administred days =date last dose- date first dose +1)

By patient for each drug:

- administred dose by patient = sum of administred doses on all cycles
- theoric dose by patient = sum of theoric doses on all cycles
- exposition (%) = administred dose by patient *100 / theoric dose by patient

4.6 Statistical Analyses

PRR and PSR cohorts will be analysed separately using the same procedure at the end of the study.

4.6.1 Primary efficacy Outcome

Population :

- Intent-to-treat population (primary analysis)
- per-protocol population (secondary analysis)

Method :

The rate of clinical and radiological non-progressive disease will be analysed as a time-to-event endpoint from the time of inclusion to the date of progression. For this endpoint, patients who die for another cause than disease progression are censored at the time of death. Patients lost to follow-up are censored at the date of their last follow-up visit. Patients who are exempt of progression and start a next systemic therapy are censored at the date of the first administration of the next systemic therapy. Patients who are alive, exempt to progression, and exempt of next systemic therapy at their last follow-up visit are censored at that date.

The rate of clinical and radiological non-progressive disease will be estimated using the Kaplan-Meier method. Confidence intervals will be provided at the 90% 2-sided confidence level. The lower limit of the 90% bilateral confidence interval being equivalent to the lower limit of the 95% unilateral confidence interval. The study conducted in the PRR cohort will be declared positive for the primary endpoint if the lower boundary of the 90% 2-sided confidence interval is higher than 50%. The study conducted in the PSR cohort will be declared positive for the primary endpoint if the lower boundary of the 90% 2-sided confidence interval is higher than 65%.

The rate of clinical and radiological non-progressive disease will be analyzed according to both set of criteria (RECIST an irRECIST).

4.6.2 Secondary efficacy outcomes

Secondary efficacy outcomes will be analysed in the ITT population.

CA 125 decline

The CA 125 decline will be calculated between inclusion and end of follow-up (30 days ±7 after last treatment administration). Summarize by mean (SD) or median (25 and 75 percentiles) depending of the data distribution.

Progression free survival

The PFS will be calculated between the date of inclusion and the date of progression or death. The follow-up of patients still alive without progression will be censored at the date of the last follow-up visit. Patients who die for another cause than disease progression will be censored at the time of death. Patients lost to follow-up will be censored at the date of their last follow-up visit (30 days ±7 after last treatment administration). Patients who are exempt of progression and start a next systemic therapy will be censored at the date of the first administration of the next systemic therapy. Patients who are alive, exempt to progression, and exempt of next systemic therapy at their last follow-up visit will be censored at the date of that visit.

The PFS curves will be estimated using Kaplan-Meier methods [Kaplan, 1958]. The median PFS will be calculated by cohort with their 95% confidence interval.

The PFS will be analyzed according to both set of criteria (RECIST and irRECIST).

Overall survival

The time to death will be calculated between the date of inclusion and the date of death. The follow-up of patients still alive will be censored at the date of the last follow-up visit (after 1 year for the two cohorts). Data on patients lost to follow-up will censored at the date of the administrative inquiry. The survival curves will be estimated using Kaplan-Meier methods [Kaplan, 1958]. The median survival will be calculated by cohort with their 95% confidence interval.

Tumor response

The tumor response will be described using the overall response categories, i.e., CR, PR, SD, NE, PD. Frequency and percentage will be calculated by category.

The tumor response will be analyzed according to both set of criteria (RECIST and irRECIST).

4.6.3 Secondary outcomes for toxicity and safety

Population : Safety population by cohort and all confounded cohorts.

Methods: Frequency and percentages of patients who had at least one AE will be calculated by category:

For adverse events (AE):

- Any AE
- Any AE related to study drug
- Any AE leading to permanent study drug discontinuation
- Any serious AE (SAE)
- Any SAE related to study drug
- Any SAE leading to permanent study drug discontinuation

For adverse event of special interest (AESI)

- Any AESIs observed with Olaparib, including:
    - Myelodysplastic syndrome,
    - Acute leukemia,
    - Any other new primary cancer,
    - Pneumonitis.

- AESIs observed with Durvalumab including:
    - Infusion-related reactions
    - Pneumonitis
    - Colitis
    - Intestinal perforation
    - Endocrinopathies
    - Nephritis
    - Rash/dermatitis
    - Myocarditis
    - Myositis/polymyositis
    - Pancréatitis

- AESIs observed with Bevacizumab including:
    - Hypertension ≥ grade 3
    - Proteinuria ≥ grade 3
    - GI perforation, abscesses and fistulae (any grade)
    - Wound healing complications ≥ grade 3
    - Haemorrhage ≥ grade 3 (any grade CNS bleeding; ≥ grade 2 haemoptysis)
    - Arterial thromboembolic events (any grade)
    - Venous thromboembolic events ≥ grade 3
    - PRES (or RPLS; any grade)

4.6.4 Trial Population description

Number of screening patients and number of patients enrolled (all inclusion criteria and no exclusion criteria). The number of patients with withdrawal from intervention and from follow-up will be calculated. The duration of participation will be calculated between the date of inclusion and the date of withdrawal or the date of last news for the lost-of follow-up patients. Reasons and details of how withdrawal/lost to follow-up data will be presented.

4.6.5 Adherence and protocol deviations

Adherence to the intervention and extent of exposure will be assessed by the number of cycles received (median and range) and by the proportion of patients with discontinuation.

Major protocol deviations for each cohort will be defined at the data review and will be summarized (frequency and percentages of patients).

A listing of patients for the following reasons will be done:

- Criteria of eligibility not respected
- Reason for not receiving treatment
- Reason for loss to follow-up
- Reason for discontinued intervention
- Exclusion from analysis

4.6.6 Baseline and at diagnosis patient characteristics

Description of patients at baseline (inclusion) will be done in the ITT population

At inclusion:

- Demographic data :
  - Age (in years)
  - Sex (F)
  - Performance status (0/1/2/3)

At diagnosis:

- Stade Figo (II/III/IV)

4.6.7 Exposition to treatments:

For each drug (Durvalumab, Bevacizumab and Olaparib), the percentage of Administred dose to theoric dose by patient will be summarized by cohort.

5 References

Guidelines for the Content of Statistical Analysis Plans in Clinical Trials (2017_Gamble)

6 Appendix

6.1 Data variables

For the Primary outcome	Data table	Variable name
time of inclusion	INCLU	INCDTC
Disease progression according to irRECIST	PFS1	P1RECIST
the date of first progression<br>IR progression confirmation	PFS1	P1RECISTDTC<br>P1IMAG
time of death	DEATH	DTHDTC
date of their last follow-up visit	CLIN	VISITDTC
date of the first administration of the next systemic therapy (subsequent line)	SUBST	Minimum of the following Start Dates :<br>STCARSTDT (Carboplatin)<br>STOTHPLSTDT (Other platinium)<br>STPACSTDT (Paclitaxel)<br>STPLDSTDT (PLD)<br>STGEMSTDT (Gemcitabine)<br>STTOPSTDT (Topotecan)<br>STOTH1STDT (Other chemotherapy 1)<br>STOTH2STDT (Other chemotherapy 2)<br>STBEVSTDT (Bevacizumab)<br>STOLASTDT (Olaparib)<br>STOTHTSTDT (Other targeted therapy)

Secondary outcomes	Data table	Variable label and Variable name
CA125 decline as expressed by the KELIM parameter	CA125	Value (kU/L) : CA125VNUM<br>KELIM parameter (not in the database but calculated separately by Pr.B.You)
PFS	INCLU<br>PFS1<br>DEATH<br>CLIN	Inclusion date INCDTC<br>Date of 1ère progression : P1RECISTDTC<br>Date of death : DTHDTC<br>Date de dernière visite : VISITDTC
OS	INCLU<br>DEATH<br>CLIN	Inclusion date : INCDTC<br>Death date: DTHDTC and DTHDT<br>Date de dernière visite : VISITDTC

Tumor response (irRECIST)<br><br>Tumor response (RECIST) to compute	EVAL_RESP<br><br>**EVAL_TL (target lesions)**<br><br>**EVAL_NTL (non target lesions)**	Radiological response:<br>Response for solid tumors - Overall response : variable TUMRESP<br>Clinical response:<br>Overall disease response - Overall response : variable OVRESP<br>Target lesions - Sum of diameter: TLSUM<br>Target lesions – Response: TLRESP<br>New target lesions: TLNYN<br>Non target lesion – Response: NTLRESP<br>New Non target lesion : NTLNYN
Toxicity as assessed by CTCAE V.5.0 scale	AE	« Adverse Event - Grade (CTCAE v5.0) » (variable AESEV)
Safety	AE (terms : LLT/PT/SOC)	AE :<br>- SOC : AESOCCODE<br>- LLT : AELLTCODE<br>- PT : AEPTCODE<br>AE related to drug study :<br>- AERELOLA : O/N<br>- AERELBEVA : O/N<br>- AERELDURVA : O/N<br><br>SAE : AESER<br>AESI (listing From external safety data)
	AE + EOT	AE leading to permanent study drug discontinuation<br>Table AE (Action taken):<br>- AEACNDURVA<br>- AEACNBEVA<br>- AEACNOLA<br>Table EOT : Variable EOTREAS

Other variables to analyse

CRF data	Data table	Variable label	Variable name
Registration		Age, sex	Age, sex
screening	INCLU	Screening failure, main reason, Inclusion - Cohort	FAIl, FAILR, FAILRP<br>INCCOHORT
visits during chemotherapy C1 à 5	DELAY	Has the treatment planned at day 1 been delayed >7 days? If yes Olaparib, Durvalumab, FKB238, reason	VISIT, CDLOLA, CDLDURV, CDLBEVA, CLB
EOT, if applicable	EOT	Nb of cycles, cause	EOTNBCYCL, EOTREAS

concomitant medication	CM	Drug name	CMTRT
End of study, if applicable	EOS	Date of study end	EOSDT, EOSREAS
death report, if applicable	DEATH	Date of death, cause	DTHDT, DTHREAS

6.2 Progression determination algorithm

Progression Disease (PD) definition according to 3 criteria:

- ✓ **Radiological:** RECIST
- ✓ **Radiological:** irRECIST with confirmation at 4 weeks or subsequent therapy
- ✓ **Clinical:** symptom of deterioration

a. Radiological: RECIST

This criterion is not collected in the e-CRF therefore an implementation of an algorithm is necessary.
Use the **% resp SAS macro** of the article "Objective tumor response and RECIST criteria in cancer clinical trials", Jian Yu, I3, Indianapolis, Indiana, in which we will add the variable **New Non target lesion.**
Preparing the data to obtain the table below:

Obs	Subject Identifier for the Study	Visit Number	Tumor assessment - Date of evaluation	Target lesions - Sum of diameter	New Target lesion - Have new lesions been observed?	Non target lesion - Response	New Non target lesion - Have new lesions been observed?
**1**	004-01	Screening	28/08/2019	32	Missing	.	Missing
**2**	004-01	Cycle 3	15/10/2019	8	No	Non-CR/Non-PD	No
**3**	004-01	Cycle 4	.		Missing	.	Missing
**4**	004-01	Cycle 5	26/11/2019	0	No	Non-CR/Non-PD	No
**5**	004-01	Cycle 7	02/01/2020	0	No	Non-CR/Non-PD	No
**6**	004-01	Cycle 9	18/02/2020	0	No	Non-CR/Non-PD	No
**7**	004-01	End of Treatment	19/03/2020	0	Yes	Unequivocal progression	No
**8**	004-01	Safety Follow up	19/03/2020	0	Yes	Unequivocal progression	No

- **3rd column**: **DATE of PD:** Date of tumor measurement: variable **EVALDT** (TABLE **EVAL_TL**) corresponds at the date of visit = date of imaging.

- **4th column** : Disease evaluation – **Sum of the longest diameters for Target lesions** (TABLE **EVAL_TL**);

    Variable **TLSUM:** Target lesions - Sum of diameter. This variable will be transformed into numeric.

If the variable TLSUM is missing (TLSUM=.) and at least 1 longest diameter is measured and the other target lesion diameters are "ticked" as NE, The sum will be calculated on the available longest diameters.

- **5th column** : Disease evaluation - **Non Target lesions response** (TABLE **EVAL_NTL**);

    Variable **NTLRESP**: Non target lesion - Response;*CR, Non-CR/Non-PD, Unequivocal progression, Not Evaluable; This variable will be transformed: CR=>CR, No-CR/No-PD=>SD, Unequivocal progression=>PD, Not Evaluable=>NE.

- **6th column** : Disease evaluation – **New Target lesions** (TABLE **EVAL_TL**);

    Variable **TLNYN**: New Target lesion - Have new lesions been observed? No/Yes

- **7th column** : Disease evaluation – **New Non-Target lesions** (TABLE **EVAL_NTL**);

    Variable **NTLNYN**: New Non-Target lesion - Have new lesions been observed? No/Yes

(Whether the new lesion is target or not it must be taken into account to determine the RECIST progression)

Use the SAS macro **%RESP_BOLD** to create table **CYCLE_RESP** with variables:

    - Variable **RESP**: time point (cycle) tumor overall response (determine order PD->NE->CR->PR->SD).

We will obtain a response for each of the cycles and the earliest date will be retained for the date of 1st progression.

Use the variable « Tumor assessment – Not done » (variable EVALND table EVAL_TL) =ticked to determine a cycle not done.

- If the cycle is not done (EVALND =ticked) then the variables New lesion target (variable TLNYN table EVAL_TL) and New lesion no target (variable NTLNYN table EVAL_NTL) are not informed.

The cycle in the prepared table will be removed.

- If the cycle is done (EVALND=missing) (will be modified directly in the macro_resp_BOLD):
    - Look the variable Target lesions – None (variable TLNONE table EVAL_TL) :

- If it is ticked (TLNONE =Ticked), there is not target lesion on the cycle ie **TLSUM=.→ resp=SD.**

However, there may be New lesions target (variable TLNYN table EVAL_TL).

- Look the variable No Target lesions – None (variable NTLNONE table EVAL_NTL) :
  - If it is ticked (NTLNONE =Ticked), there is not no target lesion on the cycle.

However, there may be New no target lesions (variable NTLNYN table EVAL_NTL).

b. Radiological: Criteria irRECIST with confirmation at 4 weeks or subsequent therapy

This criterion will be determined as below:

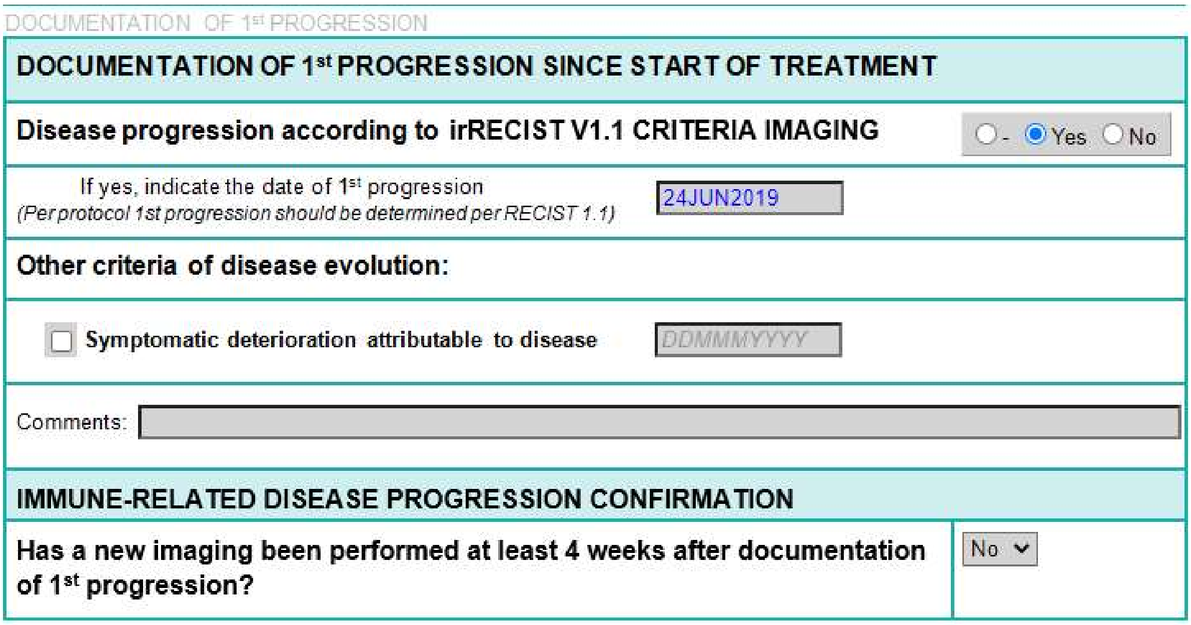
DOCUMENTATION OF 1st PROGRESSION

DOCUMENTATION OF 1st PROGRESSION SINCE START OF TREATMENT

Disease progression according to irRECIST V1.1 CRITERIA IMAGING	○ - ◉ Yes ○ No
If yes, indicate the date of 1st progression (Per protocol 1st progression should be determined per RECIST 1.1)	24JUN2019
Other criteria of disease evolution:	
☐ Symptomatic deterioration attributable to disease	DDMMMYYYY
Comments:	

IMMUNE-RELATED DISEASE PROGRESSION CONFIRMATION

Has a new imaging been performed at least 4 weeks after documentation of 1st progression?	No

Use the variables as below:

- Table **PFS1**: variable **P1RECIST** yes/no and **P1RECISTDT** for the date
- Table **PFS1**: variable **P1IMAG** yes/no.
- Table **SUBSTR**: if a patient has a new treatment, he will be in this table.

The progression according irRECIST will be defined as below:

**ALGORITHM**			**PROGRESSION**	
**Variable: P1RECIST**	**Variable: P1IMAG**	**Table SUBST**	**Progression according ir RECIST**	**Date of progression**
Yes	Yes	-	Progression=yes	P1RECISTDT
Yes	No	Yes	Progression=yes	P1RECISTDT

	The scan of confirmation will not be obliged if the patient received a new treatment to determine the progression among irRECIST criteria.	(If the patient is present in this table)		
Yes	No	No<br>(If the patient is NOT present in this table)	Progression=no<br>This should not happen. Check with the centers. The patient's folder will have to be reviewed to validate the progression according irRECIST criteria.	-

If there is progression with RECIST and irRECIST criteria, it will be necessary to check the date to be sure it will be the same.

c. Clinical criterion: symptom of deterioration

Table **PFS1**: variable **P1SYMPD** yes/no and **P1SYMPDDT** for the date.

If progression, it will be necessary to:

- ⇨ Check if this date will be the same as the date in the table CYCLES.
- ⇨ Take the earliest date of symptomatic deterioration
- ⇨ Check if the patient has progressed clinically but continued treatment: require to review the folder to ensure that this is justified.

d. Investigator criterion:

Among the reason of new subsequent therapy (table SUBST), a progression according the investigator will be considered. The date of progression will be the date of new subsequent therapy.

e. Common to the 4 progressions

- **7th column** : **Date of started treatment** by taking the minimum of the started date of Olaparib, Fkb238, Durvalumab **FIRSTDT=min(DURVAADT,OLASTDT,BEVAADT);**
    - TABLE **DURVA** (at 1rst visit):

        - Variable **DURVADT**= Durvalumab - Date of infusion
  - TABLE :
    - **OLAPARIB** (at 1rst visit) :
      - Variable **OLASTDT**= Olaparib - Start date of administration
  - TABLE **Fkb238** (at 1rst visit) :
      - Variable **BEVADT**= Bevacizumab (FKB238) - Date of infusion

- Cohort PRR (table **INCLU**: variable **INCCOHORT**): The time between the start of treatment and progression or 3 months will be calculated (**delai_eval_first=date -FIRSTDT**) and the survival to no-progression will be given.
- Cohort PSR (table **INCLU**: variable **INCCOHORT**): The time between the start of treatment and progression or 6 months will be calculated (**delai_eval_first=date -FIRSTDT**) and the survival to no-progression will be given.

If there is a progression exactly at 3 months for the PRR cohort or at 6 months for PSR cohort, it wouldn't be considered.

6.3 Clinical – radiological primary outcome

The primary outcome is the rate of clinical or radiological non-progression, the date of the first progression (clinical or radiological) must be taken. Nevertheless, it seems important to carry out a quality control of the data and to check the possible files or there would be a discrepancy (example: clinical progression at 3 months, response / radiological stability at 3 and a half months and continued treatment, then clinical response.) In this case, the reality of the clinical progression is questionable and should be verified with the centers.

6.4 Not Evaluable patients

There shouldn't be «Not Evaluable» response.
If there is:

- If it is only 1 evaluation between others (« stables » or « response ») - > need to check with centers.
- If patients are classified «Not evaluable» on all the follow-up to the progression-> need to classify «stable» and probably check with centers.

6.5 Overall response

There are two responses:

- Radiological response
- Clinical response

The 2 variables are in the table EVAL_RESP:

- Radiological response: variable TUMRESP: Response for solid tumors - Overall response (at the top part). If the variable TUMRESPND=Ticked then TUMRESP will not be considered.

- Clinical response: variable OVRESP : Overall disease response - Overall response (at the bottom part)

  If the variable TUMRESPND=Ticked then TUMRESP will not be considered.

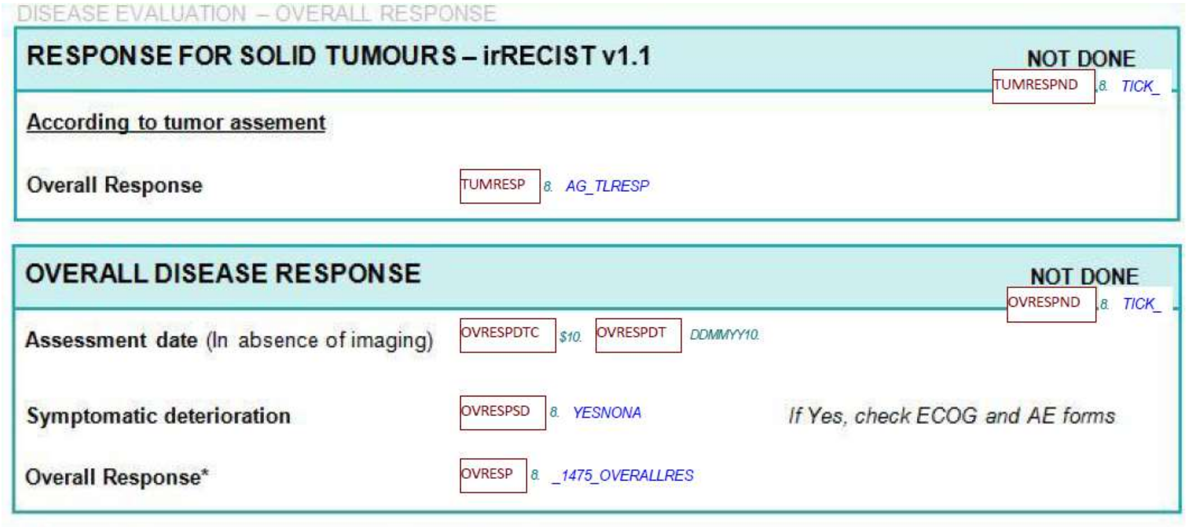

DISEASE EVALUATION – OVERALL RESPONSE

RESPONSE FOR SOLID TUMOURS – irRECIST v1.1

NOT DONE TUMRESPND 8. TICK_

According to tumor assement

Overall Response TUMRESP 8. AG_TLRESP

OVERALL DISEASE RESPONSE

NOT DONE OVRESPND 8. TICK_

Assessment date (In absence of imaging) OVRESPDTC $10. OVRESPDT DDMMYY10.

Symptomatic deterioration OVRESPSD 8. YESNONA If Yes, check ECOG and AE forms

Overall Response* OVRESP 8. _1475_OVERALLRES
