# Supplementary material for: Bevacizumab, olaparib, and durvalumab in patients with relapsed ovarian cancer: a phase II clinical trial from the GINECO group
Source: Nat Commun. 2024 Mar 5;15:1985. doi: 10.1038/s41467-024-45974-w (PMC10914754; doi:10.1038/s41467-024-45974-w)
Supplement: Supplementary file 3 — Description of Additional Supplementary Files [file 41467_2024_45974_MOESM3_ESM.pdf]

Description of Additional Supplementary Files

File Name: Supplementary Data 1
Description: **Raw counts:** calculation for all genes included in the Nanostring signatures

File Name: Supplementary Data 2
Description: **360 reports:** Nanostring signatures for all patients included
