# Supplementary material for: Bevacizumab, olaparib, and durvalumab in patients with relapsed ovarian cancer: a phase II clinical trial from the GINECO group
Source: Nat Commun. 2024 Mar 5;15:1985. doi: 10.1038/s41467-024-45974-w (PMC10914754; doi:10.1038/s41467-024-45974-w)
Supplement: Supplementary file 6 — Reporting Summary [file 41467_2024_45974_MOESM6_ESM.pdf]

nature portfolio

Corresponding author(s): Gilles Freyer

Last updated by author(s): Feb 8, 2024

Reporting Summary

Nature Portfolio wishes to improve the reproducibility of the work that we publish. This form provides structure for consistency and transparency in reporting. For further information on Nature Portfolio policies, see our Editorial Policies and the Editorial Policy Checklist.

Statistics

For all statistical analyses, confirm that the following items are present in the figure legend, table legend, main text, or Methods section.

n/a	Confirmed	
☐	☒	The exact sample size (*n*) for each experimental group/condition, given as a discrete number and unit of measurement
☒	☐	A statement on whether measurements were taken from distinct samples or whether the same sample was measured repeatedly
☐	☒	The statistical test(s) used AND whether they are one- or two-sided<br>*Only common tests should be described solely by name; describe more complex techniques in the Methods section.*
☐	☒	A description of all covariates tested
☐	☒	A description of any assumptions or corrections, such as tests of normality and adjustment for multiple comparisons
☐	☒	A full description of the statistical parameters including central tendency (e.g. means) or other basic estimates (e.g. regression coefficient) AND variation (e.g. standard deviation) or associated estimates of uncertainty (e.g. confidence intervals)
☐	☒	For null hypothesis testing, the test statistic (e.g. *F*, *t*, *r*) with confidence intervals, effect sizes, degrees of freedom and *P* value noted<br>*Give P values as exact values whenever suitable.*
☒	☐	For Bayesian analysis, information on the choice of priors and Markov chain Monte Carlo settings
☒	☐	For hierarchical and complex designs, identification of the appropriate level for tests and full reporting of outcomes
☒	☐	Estimates of effect sizes (e.g. Cohen's *d*, Pearson's *r*), indicating how they were calculated

Our web collection on statistics for biologists contains articles on many of the points above.

Software and code

Policy information about availability of computer code

Data collection	EDC used was Ennov Clinical
Data analysis	SAS version 9.4, R software v4.1.1, NONMEM 7.5.0 software

For manuscripts utilizing custom algorithms or software that are central to the research but not yet described in published literature, software must be made available to editors and reviewers. We strongly encourage code deposition in a community repository (e.g. GitHub). See the Nature Portfolio guidelines for submitting code & software for further information.

Data

Policy information about availability of data

All manuscripts must include a data availability statement. This statement should provide the following information, where applicable:

- Accession codes, unique identifiers, or web links for publicly available datasets
- A description of any restrictions on data availability
- For clinical datasets or third party data, please ensure that the statement adheres to our policy

Data sharing in a public repository was not planned at the start of the study. Per European and French regulations for personal data privacy, this is not permitted without having informed the study participants which was not done. This is also linked to a confidentiality agreement with AstraZeneca who provided the drug and funding. This agreement aims to guarantee protection for the company about potential sub-licensable or patentable information/discovery. Requests to access the deidentified data for further scientific use can be sent to ARCAGY-GINECO (Sébastien Armanet sarmanet@arcagy.org) and will be considered on a case-by-case basis in a timely manner beginning 3 months and ending 5 years after this article publication. Request must contain a proposal with scientific and methodologically

justified objectives. A Data Transfer Agreement will be established to provide a formal framework regarding the use of the data. The deidentified data underlying the results generated in this article are provided in the Source Data, Supplementary Data 1 and Supplementary Data 2 files. The study protocol and statistical analysis plan are available (Supplementary Information Note 1).

Research involving human participants, their data, or biological material

Policy information about studies with human participants or human data. See also policy information about sex, gender (identity/presentation), and sexual orientation and race, ethnicity and racism.

Reporting on sex and gender	Only females were included in this study of ovarian cancer
Reporting on race, ethnicity, or other socially relevant groupings	Race, ethnicity, and socially relevant grouping not reported
Population characteristics	N=74; Median age 65.5 years (38-89), 66 patients (89%) ovarian primary / 8 patients (11%) peritoneal primary, 15 patients (20%) BRCA1/2 mutant, 41 patients platinum-resistant / 33 patients platinum-sensitive
Recruitment	Patients were recruited in 9 oncology hospitals/sites in France by the treating physician from on the basis of eligibility criteria stipulated in the protocol. There were no potential self-selection bias or other biases present that we are aware of.
Ethics oversight	The study was approved by the relevant local institutional ethics committees (CPP SUD-EST 1) and the national health authorities.

Note that full information on the approval of the study protocol must also be provided in the manuscript.

Field-specific reporting

Please select the one below that is the best fit for your research. If you are not sure, read the appropriate sections before making your selection.

☒ Life sciences ☐ Behavioural & social sciences ☐ Ecological, evolutionary & environmental sciences

For a reference copy of the document with all sections, see nature.com/documents/nr-reporting-summary-flat.pdf

Life sciences study design

All studies must disclose on these points even when the disclosure is negative.

Sample size	A one-stage design and the exact binomial distribution was used, and the sample size was calculated independently in the two cohorts. For the platinum-resistant cohort, the objective was to exclude a 3-month non-progressive disease rate of ≤50%, with a positive hypothesis of 75%. A total of 23 evaluable patients yields a maximum one-sided type-1 error rate of α=5% and a power of ≥80% when the true non-progressive disease rate is 75%. For the platinum-sensitive cohort, the objective was to exclude a 6-month non-progressive disease rate of ≤65%, with a positive hypothesis of 84%. A total of 40 evaluable patients yields a one-sided type-1 error rate of α=3% maximum and a power of ≥82% when the true non-progressive disease rate is 84%.
Data exclusions	No data were excluded
Replication	The assessment of the prognostic role of the TIS was not done with replicative analysis, since the cut-off were already kown. It was a univariate analysis. Regarding KELIM, this parameter derives from CA125 modelling and the parameters of the model are specific to each situation (i.e. first line, relapse, more advanced lines). The cut-off for Kelim therefore has to be reassessed for every new situation. In the BOLD study, it was used for the first time in a trial with no cytotoxic chemoterapy. Since the calculation required replicative analyses, our findings with Kelim are therefore mainly a "proof of concept" and would need further validation in a prospective manner.
Randomization	This is not a randomized study because there is a single treatment regimen evaluated.<br>Note that it was evaluated in 2 study populations selected on the basis of their platinum resistance status - this is not a factor for randomization
Blinding	Blinding is irrelevant when there is a single treatment possible.

Reporting for specific materials, systems and methods

We require information from authors about some types of materials, experimental systems and methods used in many studies. Here, indicate whether each material, system or method listed is relevant to your study. If you are not sure if a list item applies to your research, read the appropriate section before selecting a response.

Materials & experimental systems

n/a	Involved in the study
☒	☐ Antibodies
☒	☐ Eukaryotic cell lines
☒	☐ Palaeontology and archaeology
☒	☐ Animals and other organisms
☐	☒ Clinical data
☒	☐ Dual use research of concern
☒	☐ Plants

Methods

n/a	Involved in the study
☒	☐ ChIP-seq
☒	☐ Flow cytometry
☒	☐ MRI-based neuroimaging

Clinical data

Policy information about clinical studies

All manuscripts should comply with the ICMJEguidelines for publication of clinical research and a completedCONSORT checklist must be included with all submissions.

Clinical trial registration	NCT04015739
Study protocol	Copy of the protocol provided to the journal editor and made available as a supplementary file (Note 1)
Data collection	An open-label, parallel cohort, single arm phase II study conducted in nine French centers with a total of 74 patients enrolled and treated between 01 March 2019 and 29 January 2020. Patients were invited to participate as part of their routine care at one of he following institutions: Centre Léon Bérard, Lyon; Centre Oscar Lambret, Lille; Groupe Hospitalier Diaconesses Croix Saint-Simon, Paris; Institut Gustave Roussy, Villejuif; Institut de Cancérologie des HCL, Lyon; Institut du Cancer de Montpellier, Montpellier; IInstitut Bergonié, Bordeaux; Institut Curie Saint Cloud, Paris; Institut Claudius Regaud IUCT-Oncopole, Toulouse
Outcomes	Endpoints were planned prospectively in the protocol. The primary endpoint was the non-progression rate at 3 months for platinum-resistant relapse or 6 months for platinum-sensitive relapse which was assessed by radiological imaging per RECIST 1.1 and irRECIST. It was selected because it is a standard oncology endpoint in advanced ovarian cancer. Secondary endpoints were CA-125 decline with the modeled CA-125 ELIMination rate constant K (KELIM-B) per CA-125 longitudinal kinetics over 100 days. Serum CA-125 levels were determined every 6 weeks. CA-125 is a standard biomarker measured in ovarian cancer. The KELIM-B is an accepted measure as referenced in the article. Full details of the methodology are provided in supplementary methods and are too long to detail here. the following endpoints are also standard oncology endpoints in ovarian cancer. Survival was measured by if the patient had died or not. Survival status was followed-up every 3 months for up to 1 year. Safety was analyzed by adverse events and clinical laboratory tests (hematology chemistry etc – please see details in the protocol – too long to elaborate here) evaluated throughout treatment per National Cancer Institute Common Terminology Criteria for Adverse Events (NCI-CTCAE), v5.0. Tumor response was evaluated by radiological imaging per RECIST 1.1 and irRECIST, at baseline and every 6 weeks until progression, per the investigator. Clinical progression was defined as symptoms considered by the investigator as disease-related. The protocol planned to assess tumor response and tumor progression according to both RECIST and irRECIST for the primary objective. However, irRECIST was subsequently considered less appropriate for reporting the final results due to due to a lower level of evidence. This is explained fully in the manuscript including a reference. Biomarkers are a standard oncology endpoint. Tumor inflammation signature (TIS) was evaluated with the Nanostring IO360 immuno-oncology panel (770 genes) using RNA from formalin-fixed paraffin-embedded baseline tumor samples (archival or fresh obtained <3 months prior to treatment start and after prior chemotherapy) based on 18 genes, as described and referenced in the manuscript. Please read the manuscript if further details are required.

Plants

Seed stocks	this study is not about plants
Novel plant genotypes	this study is not about plants
Authentication	this study is not about plants
